# Structure–Activity Relationship Studies Resulting in the Identification of Potent and Selective Salt-Inducible Kinase Inhibitors

**DOI:** 10.1021/acsmedchemlett.6c00199

**Published:** 2026-07-01

**Authors:** Daniel T. Webb, Katherine L. Jones, Gerald Keil, Matthew Holt, Nicholas Richards, Yvonne Walker, Andrea Taylor, Maryam Ahmadi, Lampros Milanos, Natsuko Macabuag, Eilidh Bush, Matthew Gill, Rebecca Penrose, David M. Lindsay, William J. Kerr, William Esmieu

**Affiliations:** £ Department of Pure and Applied Chemistry, 3527 University of Strathclyde, Glasgow G1 1XL, United Kingdom; $ IQVIA Laboratories, Chesterford Research Park, Saffron Walden CB10 1XL, United Kingdom; # 4752 Discovery from Charles River, Charles River, Chesterford Research Park, Saffron Walden CB10 1XL, United Kingdom

## Abstract

Salt-inducible kinases (SIK1/2/3) belong to the AMPK family of serine/threonine kinases, and SIK inhibition has been identified as a promising strategy for therapeutic intervention in oncology and immunology. Herein, we describe the use of parallel chemistry approaches to facilitate the development of novel SIK inhibitors. From these efforts, potent pan-SIK inhibitors were identified, as well as compounds which demonstrated biased isoform selectivity toward SIK3, and possessed attractive *in vitro* ADME properties. To validate the most promising compounds as high-quality chemical tools, cellular target engagement was assessed using NanoBRET assays, and selectivity was investigated across the wider kinome. Finally, the pharmacological effects of several compounds were studied using cellular models of acute myeloid leukemia, resulting in the identification of potent antiproliferative agents. The compounds reported represent high-quality chemical tools of potential value for target validation studies and could serve as effective starting points for a lead optimization campaign.

## Graphical abstract

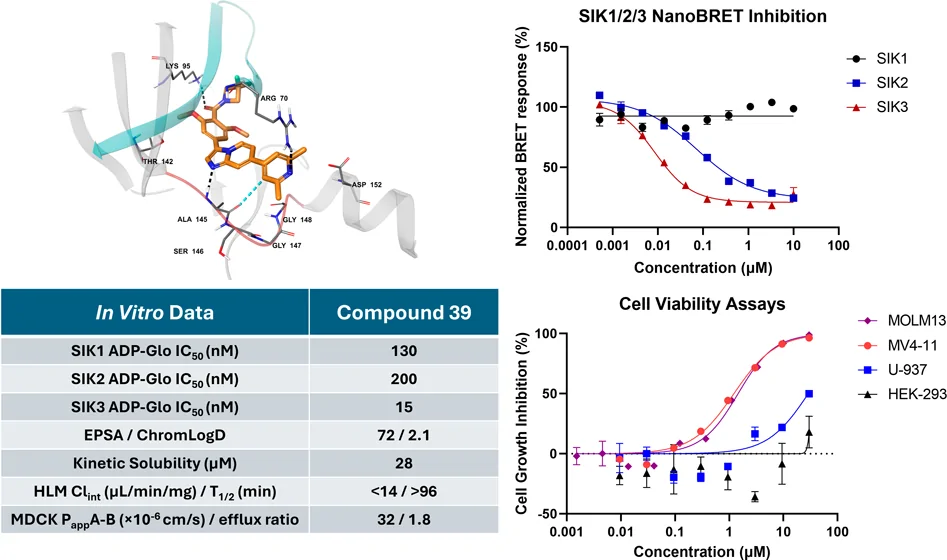

Salt-inducible kinases (SIKs) belong to the adenosine monophosphate-activated protein kinase (AMPK) family of serine/threonine kinases. Three SIK family proteins are known (SIK1, SIK2, and SIK3) and amino acid conservation within the kinase domain is high across all three isoforms (55–70% identity).^1–3^ SIK activity is regulated by phosphorylation by liver kinase B1 (LKB1) and is disrupted by cyclic adenosine monophosphate (cAMP)/protein kinase A (PKA)-dependent phosphorylation, which results in SIK binding to 14–3–3 proteins.^4^ The downstream targets of SIKs are class 2a HDACs and CRTC1/2/3 proteins. Phosphorylation of these targets by SIKs results in their recruitment by 14–3–3 proteins, and retention in the cytosol, preventing their translocation into the nucleus and their regulation of gene expression.^5^ The downstream targets of SIKs have diverse roles in physiology. CRTC proteins have reported roles in maintaining metabolic homeostasis, normal brain function, and regulating inflammatory response.^6–8^ Class 2a HDACs act as transcriptional corepressors, are expressed in a tissue-specific manner, and are involved in differentiation and development.^9,10^ Their ability to act as transcriptional corepressors is governed by signal-dependent phosphorylation, which determines whether they are localized to the cytoplasm or nucleus.^10^

Inhibition of SIK activity has been shown to drive macrophages into a pro-resolution phenotypic state (M2), and inhibition of SIKs using small molecule inhibitors has garnered much attention as a therapeutic strategy for inflammatory disorders.^11–14^ Galapagos researchers have published extensively on the subject of SIK inhibition, reporting compounds such as **GLPG3312** (**1**) (pan-SIK inhibitor), **GLPG3970** (**2**), **GLPG4876** (**3**), and **GLPG4970** (**4**) (SIK2/3 selective inhibitors) (Figure 1).^15–17^ **GLPG3312** (**1**) completed a phase 1 clinical trial (NCT03800472) in 2020. After identifying that SIK1 inhibition was dispensable for the immunoregulatory pharmacology of SIK inhibitors, the development of this compound was superseded by **GLPG3970** (**2**), which was evaluated in multiple phase 2 clinical trials in 2021 for conditions including ulcerative colitis, primary Sjögren’s Syndrome, and systemic lupus erythematosus (NCT04577794, NCT04700280, and NCT04700267, respectively). **GLPG4876** (**3**) was not advanced into the clinic as a result of genotoxicity issues in rats.^17^ No clinical update has been provided on **GLPG4970** (**4**) and we note that, while being an exceptionally potent SIK2/3 inhibitor, this compound did inhibit a significant number of other kinases when tested in a kinase panel at a concentration of 1 μM.^17^ Galapagos has also reported **GLPG4399**, a selective SIK3 inhibitor which completed a phase 1 clinical trial in healthy adults in 2022 (NCT04653467). The structure of **GLPG4399** has not been disclosed, however, an example SIK3-selective inhibitor from Galapagos, **Cmpd-219** (**5**), divulged in patent WO2020239658, is displayed in Figure 1.^18^ Furthermore, Pfizer recently progressed a colon-targeted pan-SIK inhibitor, **PF-07899895** (**6**), into clinical trials for the treatment of inflammatory bowel disease (IBD) (NCT06137729).^19^ Structurally related SIK2/3 inhibitors have also been reported, such as **SK-124** (**7**), which stimulate bone formation in mice.^20^

**Figure 1:**
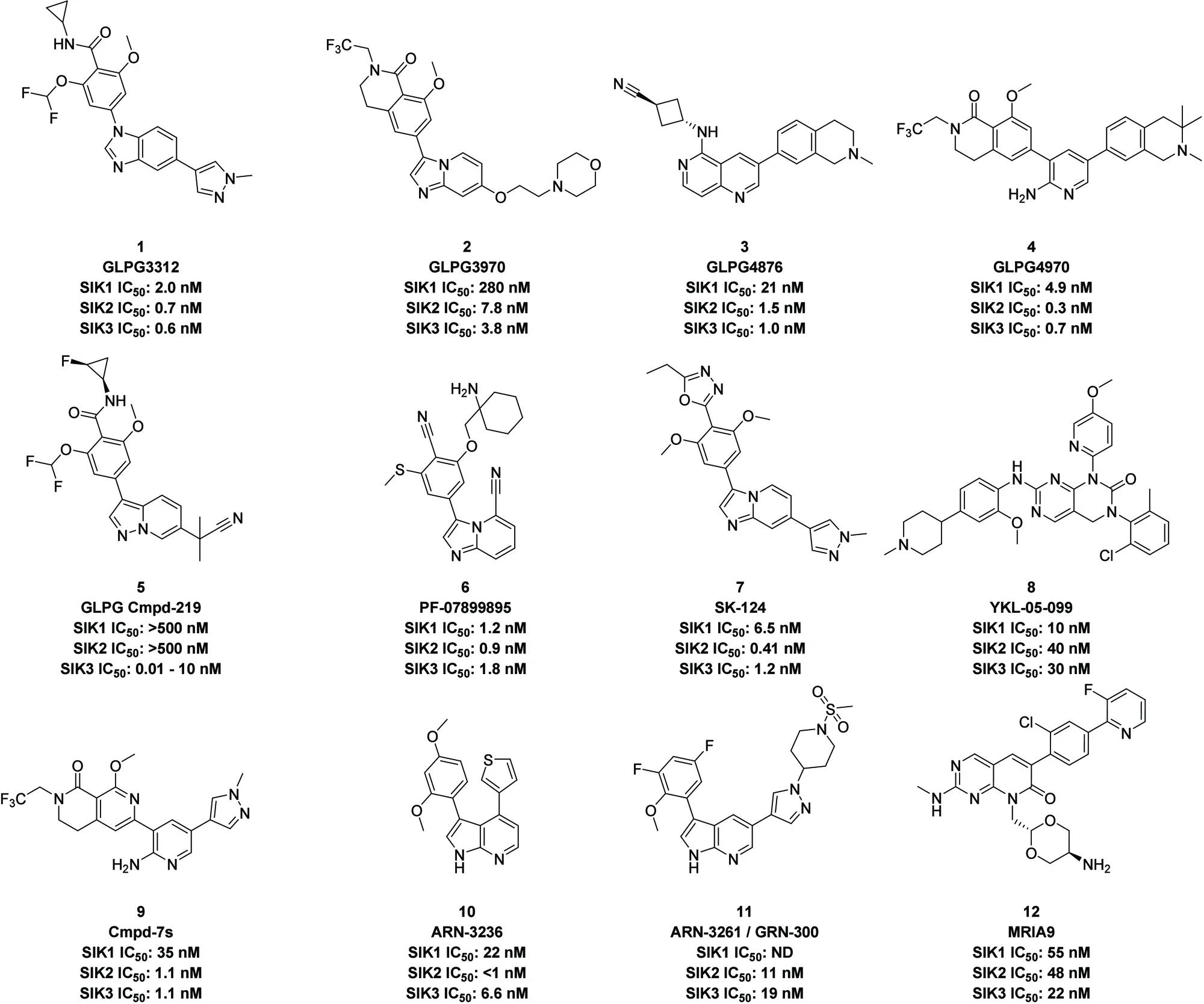
Structures and reported SIK1/2/3 IC_50_ values for selected SIK inhibitors.

SIK2/3 inhibition has also been identified as a potential therapeutic strategy for some forms of acute myeloid leukemia (AML), where myocyte enhancer factor-2C (MEF2C) is overexpressed. MEF2C signaling promotes leukemic cell self-renewal, thereby resulting in leukemogenesis.^21,22^ HDAC4 is a repressive cofactor of MEF2C, and SIK2/3 phosphorylate HDAC4, sequestering it in the cytoplasm and preventing it from repressing MEF2C activity in the nucleus.^22,23^ As such, SIK2/3 inhibition presents an attractive therapeutic strategy for MEF2C-expressing AML, and a reported pan-SIK inhibitor, **YKL-05–099** (**8**) has been shown to suppress AML progression *in vivo*.^24,25^ More recently, Insilico Medicine reported on the development of SIK2/3 inhibitors, including **Cmpd-7s** (**9**), which demonstrated strong antitumor activity in MEF2C-expressing AML xenograft models (Figure 1).^26^ SIK2 inhibition has also been identified as a promising therapeutic strategy for the treatment of ovarian cancer, and a reported SIK inhibitor, **ARN-3236** (**10**) has been shown to inhibit the growth of ovarian cancer cells and sensitizes ovarian cancer xenograft models to taxane treatment.^27^ Moreover, a related compound, ARN-3261/GRN-300 (**11**) was advanced into clinical trials for the treatment of recurrent ovarian, primary peritoneal, and fallopian tube cancers (NCT04711161).^28^

The salt-inducible kinases represent attractive therapeutic targets for indications in oncology and immunology, and a small number of chemical tools have been developed in order to facilitate drug development, such as **MRIA9** (**12**), a high-quality pan-SIK inhibitor.^29^ There are also examples of compounds which inhibit SIK1 and/or SIK2 with good selectivity over the other isoforms.^30–32^ Nevertheless, there is a lack of available high-quality chemical tools that selectively inhibit SIK3 over the other isoforms. As such, it is challenging to conduct target validation studies using small molecule inhibitors to distinguish between the roles of the different isoforms in various diseases. Furthermore, **GLPG3970** (**2**) has been proposed to exhibit toxicity associated with SIK2 inhibition and, therefore, a selective SIK3 inhibitor may present a more promising candidate for therapeutic development in certain conditions.^33^ In this work, we report on our efforts to develop novel SIK inhibitors with biased isoform selectivity toward SIK3. The compounds described herein represent high-quality chemical tools that can be used to study the role of SIKs in cellular disease models.

With a view to designing novel SIK3 inhibitors, available crystal structures of SIK3 were compared with the AlphaFold models of SIK1 and SIK2.^34–36^ In proximity of the active site, there are only 7 amino acid residues that display differences across the isoforms, and several of these are conservative replacements. Nevertheless, it was identified that three of these nonconserved residues were in the solvent exposed region of the SIK3 ATP-binding pocket. Specifically, Arg70 in SIK3 is replaced by a glycine in SIK2; Ser146 in SIK3 is replaced by a lysine in SIK1 and SIK2; and Gly147 in SIK3 is replaced by an asparagine in SIK1 and SIK2. It was hypothesized that by conducting SAR exploration around this region, it would be possible to develop compounds with biased isoform selectivity toward SIK3. An additional aim of this research was to develop SIK inhibitors with good selectivity across the wider-kinome and, with this in mind, a monodentate imidazo­[1,2-*a*]­pyridine hinge-binding motif was selected as the core scaffold, noting that this privileged structure was utilized in **GLPG3970** (**2**), **PF-07899895** (**6**), and **SK-124** (**7**) (Figure 1).

To rapidly generate SAR data, a parallel chemistry approach was employed, where an advanced imidazo­[1,2-*a*]­pyridine-containing intermediate (**19**), that was previously reported by Desroy et al.,^16^ was reacted with 24 different aryl boronates using a plate-based Suzuki-Miyaura cross-coupling protocol. The library of aryl boronates was selected with a view to yielding novel compounds covering a range of physicochemical property space, as well as varied steric and electronic properties. The key intermediate **19** was prepared according to previously reported methods.^16^ 7-Bromoimidazo­[1,2-*a*]­pyridine **13** was iodinated then reacted in a Suzuki coupling with boronate **16** (which was prepared via an iridium-catalyzed C–H borylation of arene **15**) to deliver ester **17**. Saponification of the methyl ester preceded an amide coupling to prepare intermediate **19**. For library synthesis, vials were charged with the corresponding boronic acid, cesium carbonate, and Pd­(dppf)­Cl_2_ (10 mol %). A stock solution of intermediate **19** was made up in DMF, and aliquots were added to each vial such that the reactions were carried out using approximately 0.02 mmol of the limiting reagent. Water was then added to each vial, and the plates were heated at 90 °C, with continuous stirring, for 23 h (Scheme 1). DMF was chosen as the solvent as this medium is compatible with the subsequent SFC/HPLC purification; furthermore, the reactions were not carried out under inert or anhydrous conditions. Upon reaction completion, the mixtures were filtered and purified directly by supercritical fluid chromatography (SFC), using similar purification conditions for each sample. Typically, achiral final compounds are purified by reverse phase HPLC and, under our protocols, each sample would take approximately 18 min to purify. In contrast, SFC purification takes approximately 6 min per sample. As such, employing SFC purification for compounds made via library synthesis saves a significant amount of time. Following purification, product purity was assessed by LCMS, and all compounds that showed >90% UV purity were tested in a SIK3 ADP-Glo assay, and profiled in kinetic solubility, MDCK permeability, and human liver microsomal stability assays.

**Scheme 1:**
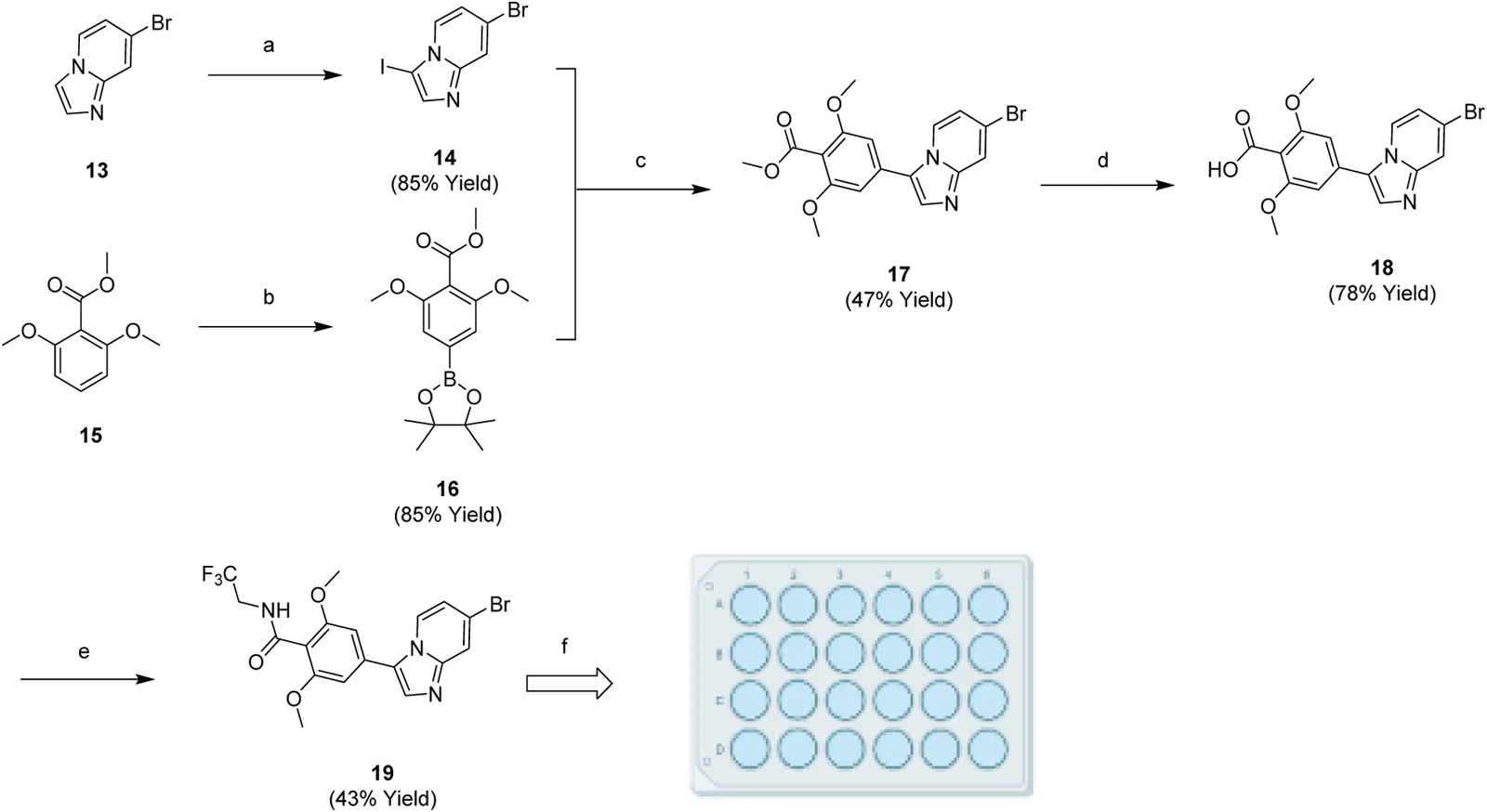
Synthesis of SIK Inhibitor Library^a^

a Reagents and conditions: (a) NIS, DMF, 100 °C, 1 h; (b) B_2_Pin_2_, (1,5-cyclooctadiene)­(methoxy)­iridium­(I) dimer, 3,4,7,8-tetramethyl-1,10-phenanthroline, THF, 65 °C, 1 h; (c) Pd­(dppf)­Cl_2_, (10 mol %), Cs_2_CO_3_, CPME/water (4:1), 90 °C, 1 h; (d) 4 M NaOH (aq.), THF/MeOH (1:1), 70 °C, 18 h; (e) 2,2,2-trifluoroethylamine, Et_3_N, HATU, DMF, r.t., 1 h; (f) aryl boronate, Pd­(dppf)­Cl_2_ (10 mol %), Cs_2_CO_3_, DMF/water (4:1), 90 °C, 23 h.

Of the 24 possible target compounds, 17 were isolated with purity >90%, translating to a 71% reaction success rate for this array. The structures of all isolated compounds are displayed in Figure 2, with SAR data presented in Table 1.

**Figure 2:**
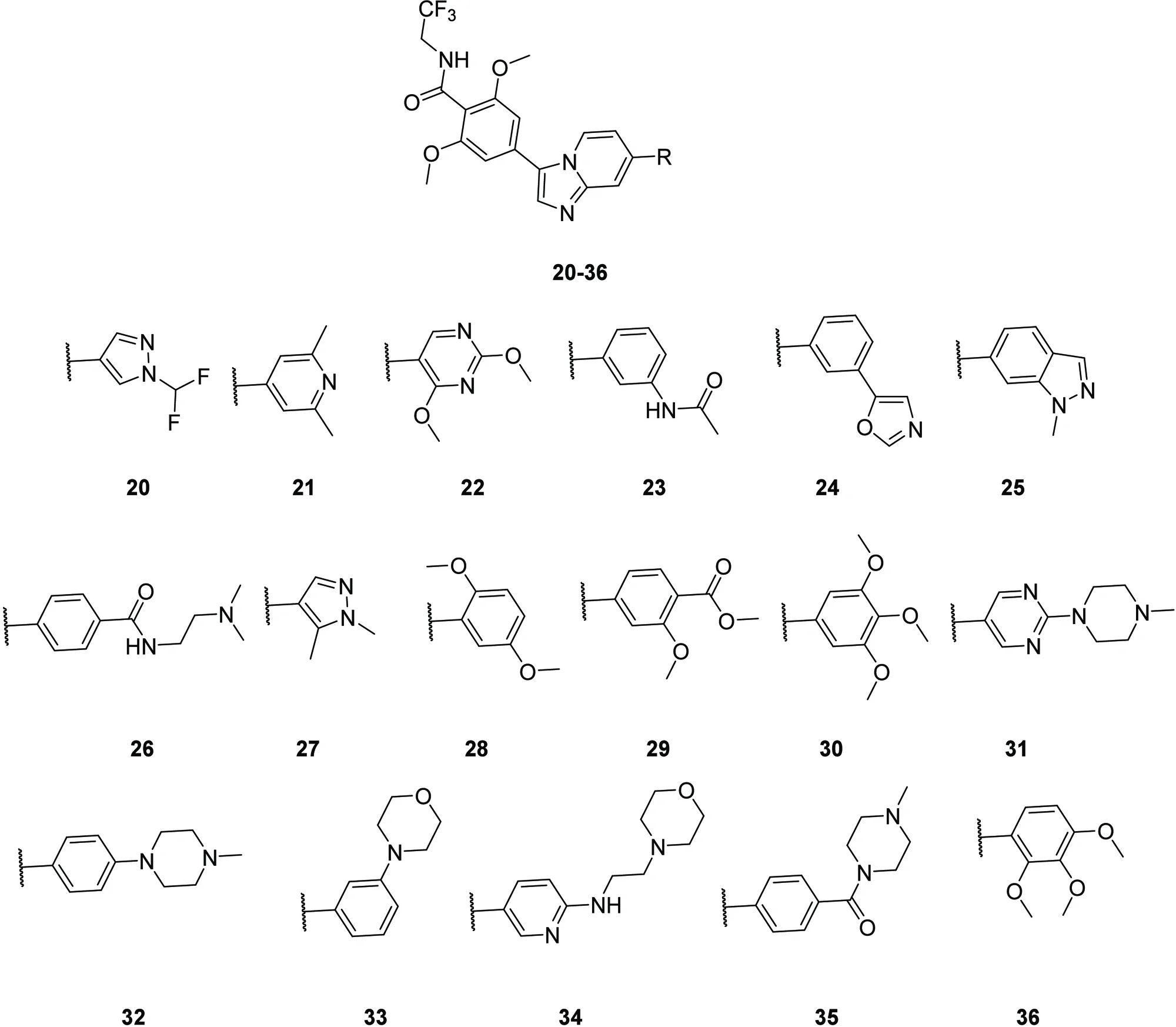
Structures of compounds successfully isolated from the Suzuki coupling array as detailed in Scheme 1.

**Table 1:** *In Vitro* Properties of SIK Inhibitors

CMPD:	SIK3 pIC_50_ ^a^	Kin. Sol. (μM)^b^	HLM Cl_int_ (μL/min/mg)^c^	MDCK P_app_A-B (×10^–6^ cm/s)/Efflux ratio^d^	cLogD/LLE	Fsp^3^
20	>8.6	24	<14	29/2.0	2.5/>6.1	0.23
21	>8.6	<5	<14	12/3.2	3.3/>5.3	0.24
22	7.1	<5	15	19/2.1	2.6/4.5	0.25
23	8.5	<5	<15	3.0/13	2.7/5.8	0.19
24	8.3	<5	45	17/1.2	3.8/4.5	0.15
25	8.1	<5	18	15/1.8	3.5/4.6	0.19
26	>8.6	76	32	0.20/17	1.9/>6.7	0.28
27	7.6	19	<15	21/2.6	2.4/5.2	0.26
28	6.7	<5	34	23/1.0	3.3/3.4	0.23
29	7.7	<5	76	6.4/2.9	3.4/4.3	0.22
30	7.5	<5	24	24/2.7	2.8/4.7	0.26
31	>8.6	57	116	11/4.1	2.7/>5.9	0.33
32	>8.6	67	76	5.1/3.1	3.1/>5.5	0.31
33	7.8	<5	75	25/1.6	2.9/4.9	0.29
34	>8.6	91	55	1.0/31	2.5/>6.1	0.35
35	>8.6	143	54	1.0/20	1.8/>6.8	0.30
36	7.1	<5	34	24/1.1	2.8/4.3	0.26

a SIK3 pIC_50_ values are reported as the geometric mean of *n* = 2 experiments.

b Kinetic solubility assessed in phosphate-buffered saline (*n* = 2).

c Metabolic stability determined in human liver microsomes (*n* = 2).

d Passive permeability measured using wild-type Madin-Darby canine kidney cells (*n* = 2).

Compounds **20–36** were tested in the SIK3 ADP-Glo assay at a concentration range of 2.5 nM – 50 μM. Several compounds were extremely potent SIK3 inhibitors, with IC_50_ values below the minimum concentration tested. In Table 1, these compounds are noted as having SIK3 pIC_50_ values of >8.6. Microsomal stability was generally good, with MDCK permeability being moderate-to-good for most compounds, although there were some notable exceptions such as compounds **23**, **26**, **34**, and **35**, which possessed low P_app_A-B values of 3.0, 0.2, 1.0, and 1.0 x 10^–6^ cm/s, respectively, and high efflux ratios (13, 17, 31, and 20, respectively). Kinetic solubility was low-to-moderate for most compounds, which was consistent with expectations based on their low sp^3^ character, with Fsp^3^ values ranging from 0.15–0.35.

We were also interested in investigating whether modification of the amide substituent would lead to improved solubility and permeability, with the aim of identifying compounds with balanced *in vitro* ADME properties that could be suitable for *in vivo* evaluation. Therefore, in parallel, we also conducted a second Suzuki array where the key intermediate **19** was replaced with compound **37**, which contained a difluoroazetidine moiety rather than a trifluoroethylamine (Figure 3). Out of the 24 possible target compounds, 13 were isolated with purity >90%, translating to a 54% reaction success rate for this plate. The structures of all isolated compounds are displayed in Figure 3, with SAR data presented in Table 2.

**Figure 3:**
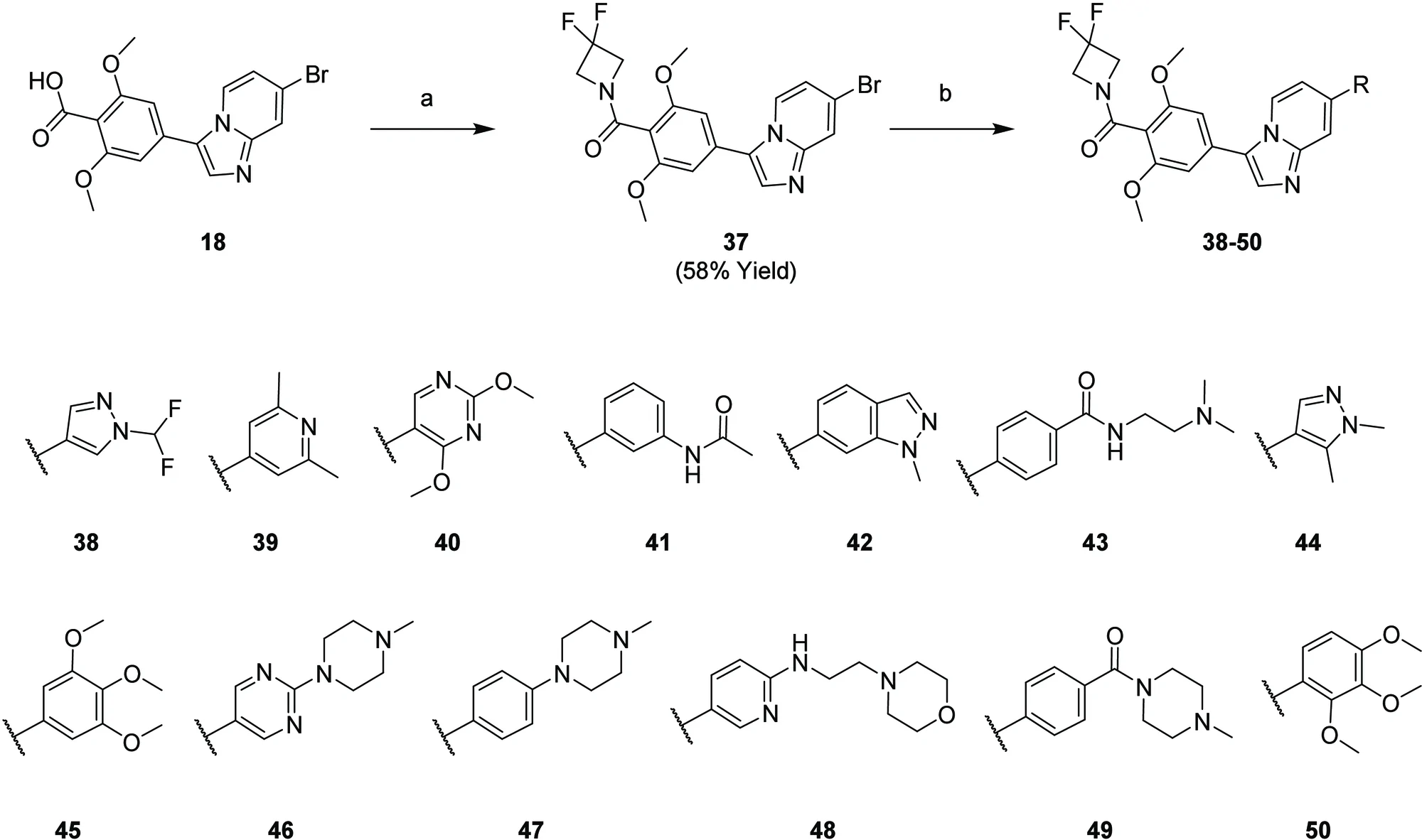
Structures of compounds successfully isolated from a second Suzuki coupling array. Reagents and conditions: (a) 3,3-difluoroazetidine hydrochloride, Et_3_N, HATU, DMF, r.t., 1 h; (b) aryl boronate, Pd­(dppf)­Cl_2_ (10 mol %), Cs_2_CO_3_, DMF/water (4:1), 90 °C, 23 h.

**Table 2:** *In Vitro* Properties Additional SIK Inhibitors

CMPD:	SIK3 pIC_50_ ^a^	Kin. Sol. (μM)^b^	HLM Cl_int_ (μL/min/mg)^c^	MDCK P_app_A-B (×10^–6^cm/s)/Efflux ratio^d^	cLogD/LLE	Fsp^3^
38	8.3	141	ND^e^	ND	1.5/6.8	0.26
39	7.9	28	<14	32/1.8	2.3/5.6	0.27
40	6.8	84	35	37/1.4	1.6/5.2	0.28
41	7.7	36	40	4.3/12	1.7/6.0	0.22
42	7.7	<5	72	34/0.9	2.5/5.2	0.22
43	8.5	116	25	0.4/13	0.9/7.6	0.30
44	6.9	150	21	25/2.3	1.4/5.5	0.29
45	7.0	36	162	21/2.2	1.8/5.2	0.29
46	7.7	108	ND	ND	1.7/7.0	0.36
47	>8.6	103	64	5.4/2.7	2.1/>6.5	0.33
48	8.4	116	189	1.3/21	1.5/6.9	0.37
49	>8.6	170	43	1.5/21	0.8/>7.8	0.32
50	6.7	<5	>277	29/1.3	1.7/6.0	0.29

a SIK3 pIC_50_ values are reported as the geometric mean of *n* = 2 experiments.

b Kinetic solubility assessed in phosphate-buffered saline (*n* = 2).

c Metabolic stability determined in human liver microsomes (*n* = 2).

d Passive permeability measured using wild-type Madin-Darby canine kidney cells (*n* = 2).

e ND = Not determined.

In general, compounds **38–50** possessed slightly reduced SIK3 inhibitory activity, although several compounds were still identified with SIK3 pIC_50_ values of >8. Additionally, the reduced lipophilicity of these compounds translated to generally improved lipophilic ligand efficiency (LLE). Furthermore, these compounds possessed improved *in vitro* ADME profiles relative to those displayed in Figure 2, with generally moderate-to-good solubility, likely associated with an increase in Fsp,^3^ moderate-to-good passive permeability, and low-to-moderate intrinsic clearance (See for matched pair analysis).

With a significant amount of SAR data generated, a selection of the prepared compounds were profiled further, using a radiometric protein kinase assay to confirm SIK3 inhibitory activity via an orthogonal method to ADP-Glo, and investigate selectivity with respect to SIK1 and SIK2 inhibition (Table 3). The nonselective kinase inhibitor, staurosporine, was included in these experiments as a positive control. To enable comparison of the selectivity of each compound, measured IC_50_ values were converted into K_i_,app values according to the Cheng-Prusoff equation, in order to account for variations in substrate affinity.^37^ All of the compounds were confirmed as being potent SIK3 inhibitors with p*K*_i_,app values >8.0. Additionally, as noted in Table 3, compound **39** displayed moderate selectivity for SIK3 over the other isoforms, and became the focus of more detailed investigations.

**Table 3:** SIK Enzymatic Inhibitory Potency of Selected Compounds

	31	34	39	48	49	Staurosporine
SIK1 p*K*_i_,app^a^	7.5	8.3	7.2	7.6	8.3	9.3
SIK2 p*K*_i_,app^a^	7.7	8.4	7.0	7.9	8.0	9.3
SIK3 p*K*_i_,app^a^	8.4	8.7	8.1	8.2	8.3	9.0
SIK1/SIK3 Fold Selectivity	9.1	2.4	8.7	3.6	1.1	0.5
SIK2/SIK3 Fold Selectivity	4.9	2.0	12	2.2	2.3	0.5

a IC_50_ values against each kinase were determined using a radiometric protein kinase assay, and the geometric mean was calculated from *n* = 2 replicates, then converted into K_i_,app values according to the Cheng-Prusoff equation.^37^

First, computational studies were conducted to investigate the structural foundation for SIK3 inhibition, using the cocrystal structure of **GLPG Cmpd-219** (**5**) bound to SIK3 (PDB: 8R4O) as the basis for docking studies.^1^ Docked poses of compounds **31**, **34**, **39**, **48**, and **49** were generated, with the pose generated for compound **39** displayed in Figure 4 (see for additional structures). Compound **39** forms a hydrogen bond through the imidazo­[1,2-*a*]­pyridine nitrogen atom with the backbone NH of Ala145 within the hinge region, as well as a hydrogen bond through the amide carbonyl with Lys95. These interactions are consistent with previously reported cocrystal structures.^1,15^

**Figure 4:**
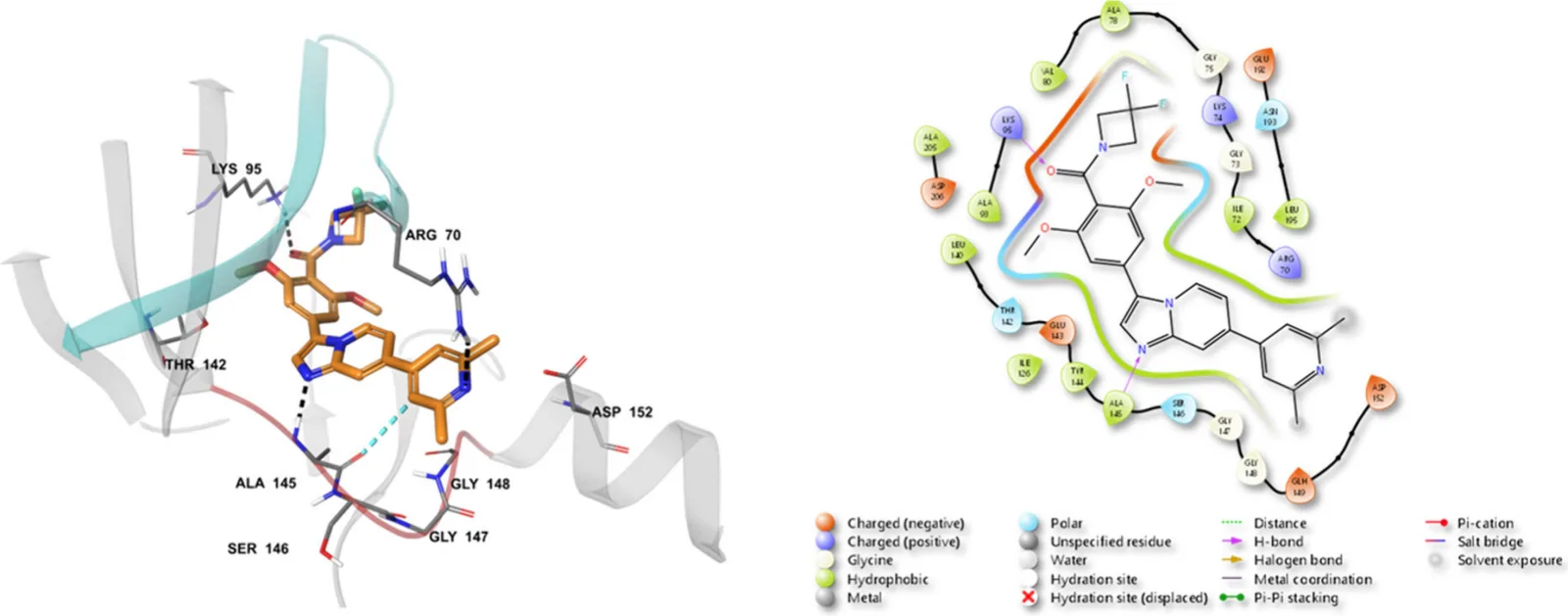
Docked structure of compound **39** bound to SIK3 (studies conducted using PDB: 8R4O).^1^ Black dashed lines indicate hydrogen bonds; blue dashed lines indicate hydrogen bonds to aromatic C–H units.

To rationalize the selectivity of compound **39**, structural alignment studies were conducted using the docked pose of **39** in SIK3, and the AlphaFold structures of SIK1 and SIK2 (Figure 5).^34–36^ One residue that is not conserved across the SIK isoforms is Ser146 in SIK3 (Lys107 in SIK1 and Lys100 in SIK2). In our alignment studies, we observed that the carbonyl oxygen within these lysine residues is oriented toward the methyl group on the pyridine ring in **39**. In SIK1, the distance between these atoms is ∼ 2.7 Å, and a steric clash is observed, and in SIK2, the distance between these atoms is ∼ 1.8 Å, with a more significant steric interaction indicated (Figure 5). In SIK3, the carbonyl oxygen of Ser146 is oriented away from the pyridyl methyl group of **39** (distance ∼ 3.8 Å) and no equivalent steric clash is observed. A rationale for the difference in orientation of the carbonyl group in these lysine residues in SIK1 and SIK2 relative to the carbonyl group in the serine residue in SIK3 can be developed by analyzing nearby amino acid residues. In the AlphaFold structures of SIK1 and SIK2, there is an interaction between Asp158/Asp151, respectively, and Asn108/Asn101, respectively, which alters the positioning of Lys107/Lys100, respectively. In SIK3, this asparagine residue is replaced by a glycine, and therefore the positioning of Ser146 is different. Furthermore, in the AlphaFold structure of SIK2, Lys100 forms a salt bridge interaction with Glu45, which further shifts the positioning of Lys100, orienting the carbonyl toward compound **39** (Figure 5). Finally, in the model of SIK1 and SIK2, an additional steric clash is observed between the pyridine ring of **39** and Gly109/Gly102, respectively, whereas in SIK3, no such steric interaction is observed between **39** and Gly148. This is likely because in SIK1 and SIK2 the adjacent residue to these glycine units is an asparagine, whereas in SIK3 the adjacent residue is another glycine and, as such, this region is more flexible and is likely able to better accommodate the bulky 2,6-dimethylpyridine moiety (Figure 5). In summary, computational studies using the crystal structure of SIK3 and AlphaFold models of SIK1 and SIK2 indicated that compound **39** inhibits SIK3 more potently than the other isoforms since the bulky 2,6-dimethylpyridine group leads to less favorable steric interactions with residues present in SIK1 and 2, which are not conserved in SIK3.

**Figure 5:**
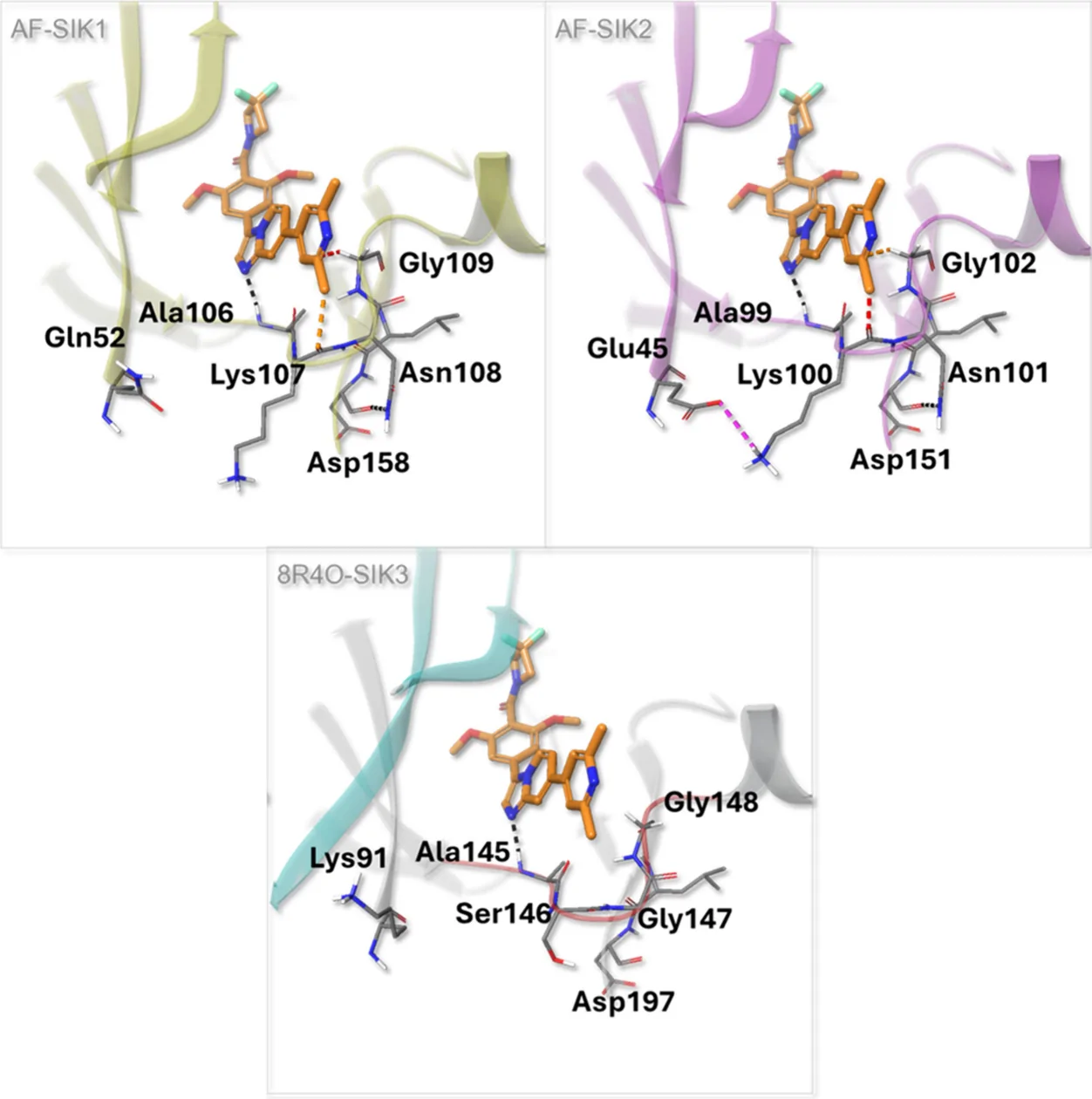
Rationale for the selectivity of compound **39**. The AlphaFold structures of SIK1 and SIK2 were superposed onto the structure of SIK3 (PDB: 8R4O, chain E) and the docked structure of compound **39**. Black dashed lines indicate hydrogen bonds; orange dashed lines indicate steric clashes; and red dashed lines indicate significant steric clashes.

To confirm the utility of compound **39** as a high-quality chemical tool, studies were conducted to investigate its wider kinase selectivity, and cellular target engagement (Figure 6). First, the effect of this compound on the activity of 140 kinases was assessed at a concentration of 100 nM. In summary, **39** showed good selectivity for the salt-inducible kinases over the wider kinome. SIK3 was confirmed to be the kinase most inhibited by **39**, and an S-score (50) of 0.043 was measured, indicating that, at a concentration of 100 nM, **39** reduces the activity of <5% of tested kinases by >50% (Figure 6a). Subsequent biochemical assays confirmed that compound **39** inhibits RIPK2 (pIC_50_ = 7.2), but not Aurora B, ERK5, or IRAK1 (pIC_50_ < 4.5), thus demonstrating that **39** possesses excellent kinase selectivity (). SIK1/2/3 inhibitory potency was also measured in HEK-293 cells using a NanoBRET assay (Figure 6b). In this assay, the cellular SIK3 and SIK2 pIC_50_ values of **39** were determined to be 8.1 and 7.1, respectively; furthermore, no significant SIK1 inhibition was observed up to a concentration of 10 μM, indicating that **39** potently inhibits SIK3 in a cellular context, with modest selectivity over SIK2 (8-fold) and excellent selectivity over SIK1.

**Figure 6:**
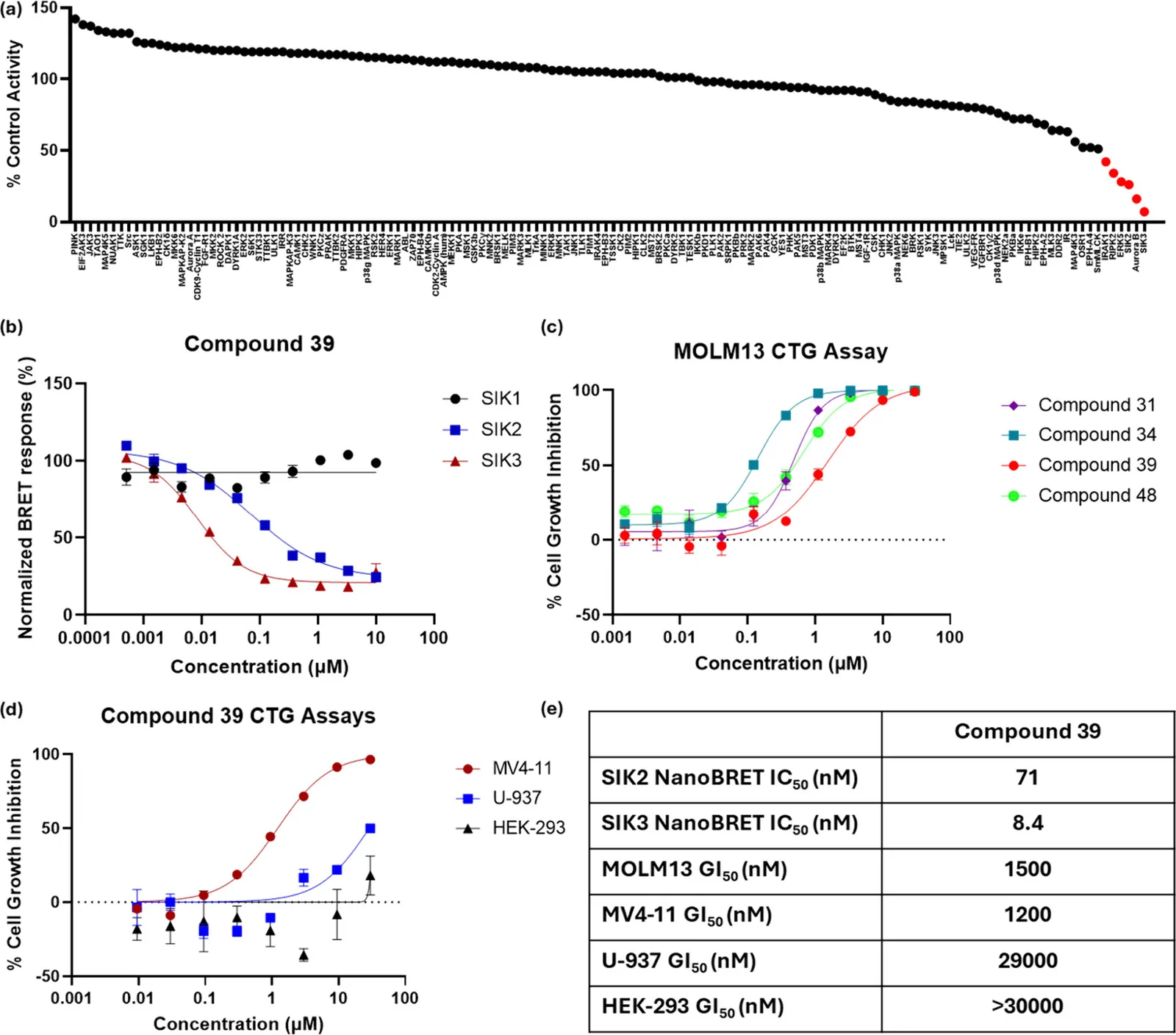
Further assessment of SIK inhibitors. (a) The effect of **39** on the activity of a general panel of 140 kinases was assessed. Kinases showing >50% inhibition are highlighted in red. (b) SIK1/2/3 IC_50_ curves for **39** determined using a NanoBRET assay in HEK-293 cells. Data plotted is the mean ± SEM of *n* = 2 biologically independent replicates. (c) Cell viability assay results for indicated SIK inhibitors in MOLM13 cells. Cells were treated with the compound at a range of concentrations, for a period of 4 days, then cell viability was assessed using a CTG assay. Data plotted is the mean ± SEM of *n* = 2 biologically independent replicates. (d) Cell viability assays for compound **39** in additional cell lines. Cells were treated with the compound at a range of concentrations, for a period of 3 days, then cell viability was assessed using a CTG assay. Data plotted is the mean ± SEM of *n* = 2 biologically independent replicates. (e) Summarized cellular assay data for compound **39**.

Subsequently, the *in vitro* phenotypic effects of **39**, as well as compounds **31**, **34**, and **48**, were investigated using a MEF2C-expressing cellular model of acute myeloid leukemia. Tarumoto et al. demonstrated, using genetic methods, that SIK2 and SIK3 have a partially redundant relationship in MEF2C overexpressing AML. In their studies, none of the tested MEF2C-expressing AML cell lines were affected by targeting SIK2 alone, however, the cytotoxic effect of SIK3 knockdown was enhanced by simultaneous knockdown of SIK2.^22^ Similarly, the SIK2/3 inhibitors **YKL-05-099** (**8**) and **Cmpd-7s** (**9**) were shown to be potent cytotoxic agents in MEF2C-expressing AML cell lines such as MOLM13.^25,26^ We sought to reproduce these findings using our chemical tools, by conducting CellTiter-Glo (CTG) cell viability assays in MOLM13 cells. All the compounds were shown to be cytotoxic in this cell line, with GI_50_ values ranging from 145 to 1520 nM, confirming that SIK inhibition induces a cytotoxic effect in MOLM13 cells (Figure 6c). Interestingly, **39**, which was a moderately isoform-selective SIK3 inhibitor, was found to be the least effective antiproliferative agent. This indicated that selectively targeting SIK3 yields a less-potent antiproliferative effect than targeting a combination of SIK isoforms in MEF2C-expressing AML disease cell models and is consistent with the studies conducted by Tarumoto et al.^22^ Compound **39** was then evaluated in CTG assays in additional cell lines. Specifically, MV4-11 and U-937 were employed, representing MEF2C-high and MEF2C-low AML cell lines, respectively, and HEK-293 cells were used as a noncancerous control cell line. In summary, compound **39** induced cytotoxicity in MV4-11 cells (GI_50_ = 1.2 μM), and no significant effect was observed in U-937 and HEK-293 cells (Figure 6d). Collectively, these findings indicate that compound **39** inhibits cellular SIK activity, selectively inducing cytotoxicity in MEF2C-high AML cell lines (MOLM13 and MV4-11) while sparing MEF2C-low AML cell lines (U-937) as well as noncancerous cells. Finally, some studies have suggested that SIK inhibition induces cytotoxicity in ovarian cancer cell lines.^27,28^ To investigate this effect, CTG assays were conducted in SK-OV3 cells using compounds **34** and **39**. In these assays, neither compound induced cytotoxicity at concentrations up to 30 μM, indicating that SIK inhibition does not induce cytotoxicity in ovarian cancer cell models (). Collectively, these studies demonstrate the applicability of compound **39** as a chemical tool to study the role of SIK2/3 in cellular disease models.

In summary, the salt-inducible kinases represent high value therapeutic targets, and SIK inhibition has been highlighted as a potential strategy for therapeutic intervention in diseases including ulcerative colitis, acute myeloid leukemia, and ovarian cancer.^16,25–28^ In this report, we described our endeavors aimed at identifying novel SIK inhibitors, using parallel chemistry to facilitate the generation of an appreciable body of SAR data. This resulted in the identification of novel high-quality pan-SIK inhibitors **31**, **34**, **48**, and **49**, as well as compound **39**, which demonstrated a biased selectivity profile toward SIK3. This compound demonstrated good selectivity across a general panel of 140 kinases, and was cell active, with virtually no drop-off between biochemical and cellular SIK3 inhibitory potency. Furthermore, *in vitro* ADME data generated for this compound indicated that it would be a good candidate for *in vivo* pharmacokinetic studies. To demonstrate the applicability of the compounds described, their phenotypic effect was assessed in MOLM13 cells, resulting in the identification of potent antiproliferative agents, and compound **39** was evaluated in additional cellular models of acute myeloid leukemia, demonstrating its applicability as a chemical tool. We believe that the compounds described will be valuable for target validation studies and would serve as effective starting points for a lead optimization campaign.

## Supplementary Material





## Notes

Molecular formula strings of final compounds (CSV)
Additional figures and tables, experimental procedures, and analytical spectra for key intermediates and final compounds (PDF)

## Abbreviations

ADME: absorption, distribution, metabolism, and excretion
AML: acute myeloid leukemia
AMPK: adenosine monophosphate-activated protein kinase
cAMP: cyclic adenosine monophosphate
ATP: adenosine triphosphate
CPME: cyclopentyl methyl ether
CRTC: cAMP-regulated transcriptional coactivators
CTG: CellTiter-Glo
DMF: *N*,*N*-dimethylformamide
dppf: bis­(diphenylphosphino)­ferrocene
ERK5: Extracellular signal-Regulated Kinase 5
GI_50_: half maximal growth inhibitory concentration
HATU: hexafluorophosphate azabenzotriazole tetramethyl uronium
HDAC: histone deacetylase
HLM: human liver microsome
HPLC: high-performance liquid chromatography
IBD: inflammatory bowel disease
IC_50_: half maximal inhibitory concentration
IL: interleukin
IRAK1: Interleukin-1 receptor-associated kinase 1
LCMS: liquid chromatography–mass spectrometry
LKB1: liver kinase B1
LLE: lipophilic ligand efficiency
MDCK: Madin-Darby canine kidney cells
MEF2C: myocyte enhancer factor-2C
NF-κB: Nuclear Factor kappa-light-chain-enhancer of activated B cells
NIS: *N*-iodosuccinimide
PKA: protein kinase A
r.t.: room temperature
SAR: structure–activity relationship
SEM: Standard Error of the Mean
SFC: supercritical fluid chromatography
SIK: salt-inducible kinase
THF: tetrahydrofuran
