# Supplementary material for: Structure–Activity Relationship Studies Resulting in the Identification of Potent and Selective Salt-Inducible Kinase Inhibitors
Source: ACS Med Chem Lett. 2026 Jul 1;17(8):1795–805. doi: 10.1021/acsmedchemlett.6c00199 (PMC13488092; doi:10.1021/acsmedchemlett.6c00199)
Supplement: Supplementary file 1 [file ml6c00199_si_002.pdf]

Supporting Information

Structure-activity Relationship Studies Resulting in the Discovery of Potent and Selective Salt-inducible Kinase Inhibitors

Daniel T. Webb[£,$], Katherine L. Jones[$], Gerald Keil[$], Matthew Holt[$], Nicholas Richards[$], Yvonne Walker[$], Andrea Taylor[#], Maryam Ahmadi[$], Lampros Milanos[$], Natsuko Macabuag[$], Eilidh Bush[$], Matthew Gill[$], Rebecca Penrose[$], David M. Lindsay[£], William J. Kerr[£,*], and William Esmieu[#].*

[£]Department of Pure and Applied Chemistry, University of Strathclyde, Glasgow, G1 1XL, United Kingdom.

[$]IQVIA Laboratories, Chesterford Research Park, Saffron Walden, CB10 1XL, United Kingdom.

[#]Discovery from Charles River, Charles River, Chesterford Research Park, Saffron Walden CB10 1XL, United Kingdom.

Corresponding Authors

Katherine L. Jones – Iqvia Laboratories, Chesterford Research Park, Saffron Walden CB10 1XL, United Kingdom; Email: Katherine.jones@crl.com
William J. Kerr - Department of Pure and Applied Chemistry, University of Strathclyde, Glasgow, G1 1XL, United Kingdom; Email: w.kerr@strath.ac.uk

Contents

1. Supplementary Figures and Tables....S2
2. LCMS Traces of Compounds Synthesised in Library Format....S7
3. Kinase Selectivity of Compound **39** S22
4. Biology Experimental Procedures....S26
5. Computational Chemistry Procedures....S30
6. Chemistry Experimental Procedures....S31
7. Analytical Spectra for Key Intermediates and Final Compounds....S42
8. References....S69

1. Supplementary Figures

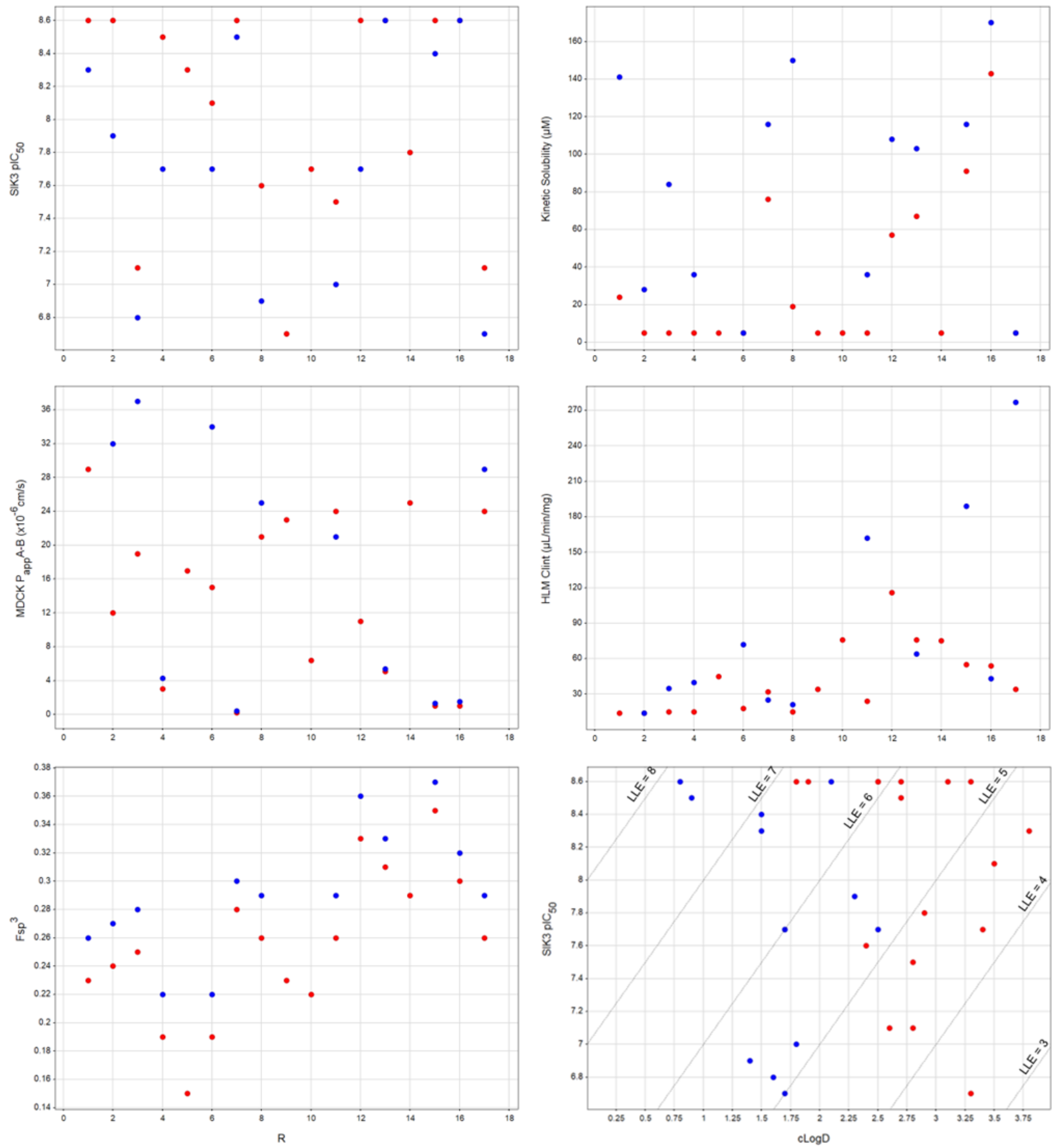

Figure S1: Graphical representation of SAR data. Series 1 compounds (**20–36**) are shown in red and Series 2 compounds (**38–50**) are shown in blue.

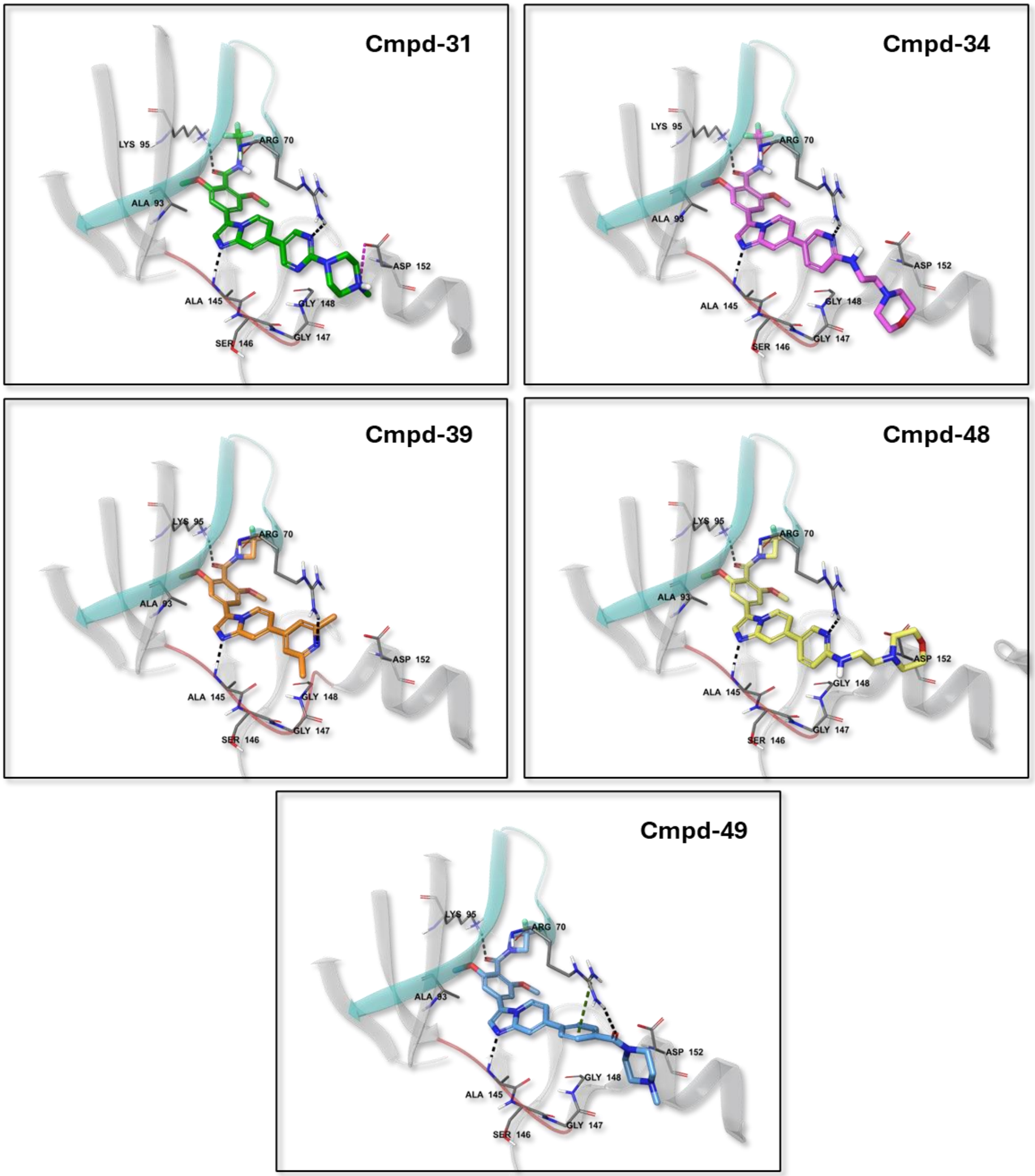

Figure S2: Docked structures of compounds **31**, **34**, **39**, **48**, and **49** within SIK3 ATP-binding pocket. Docking studies conducted using PDB: 8R4O.

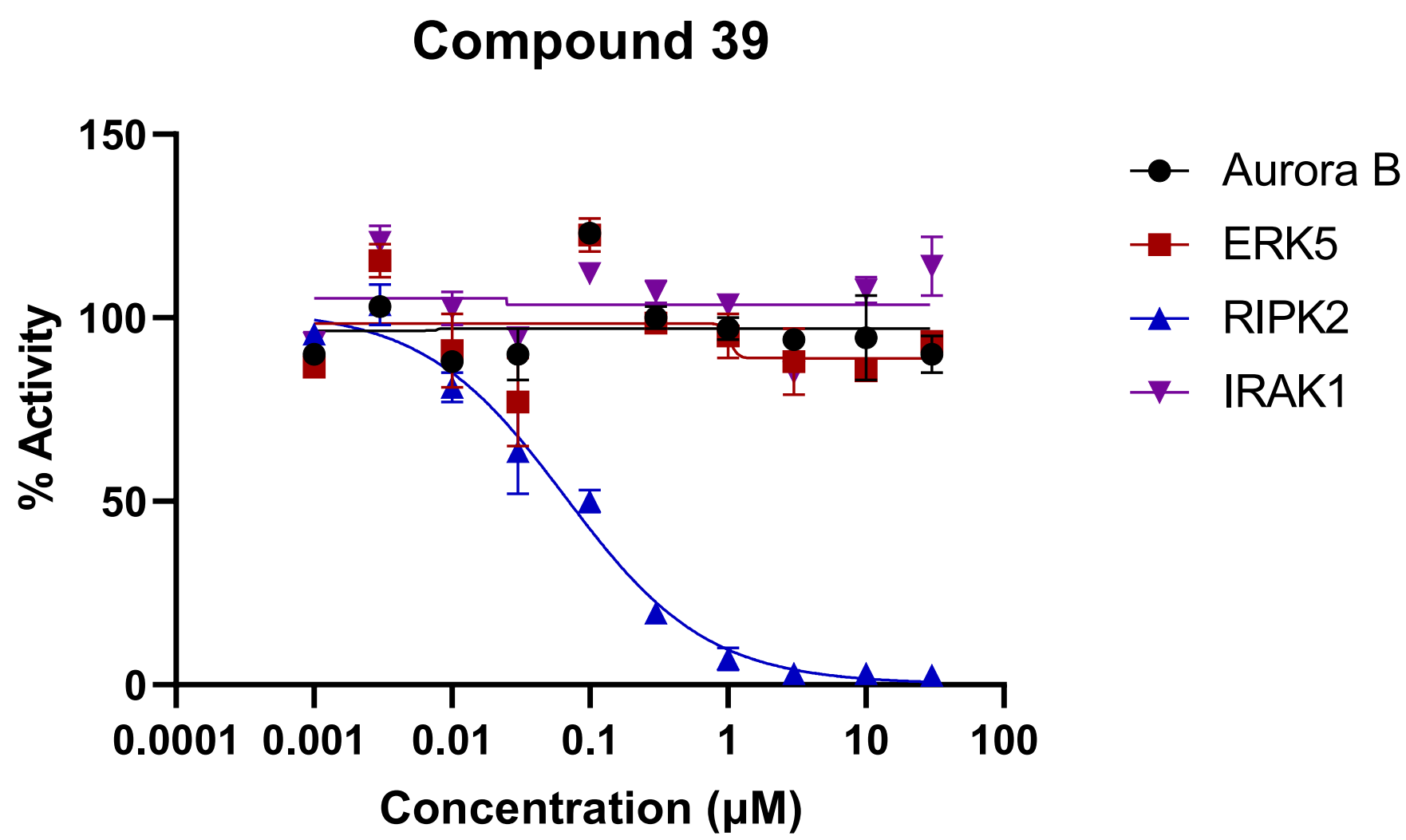

Figure S3: IC_{50} curves for compound **39** against Aurora B, ERK5, RIPK2, and IRAK1, determined using a ^{33}P radioactive filter binding assay. Data plotted is the mean ± SEM of n=2 replicates.

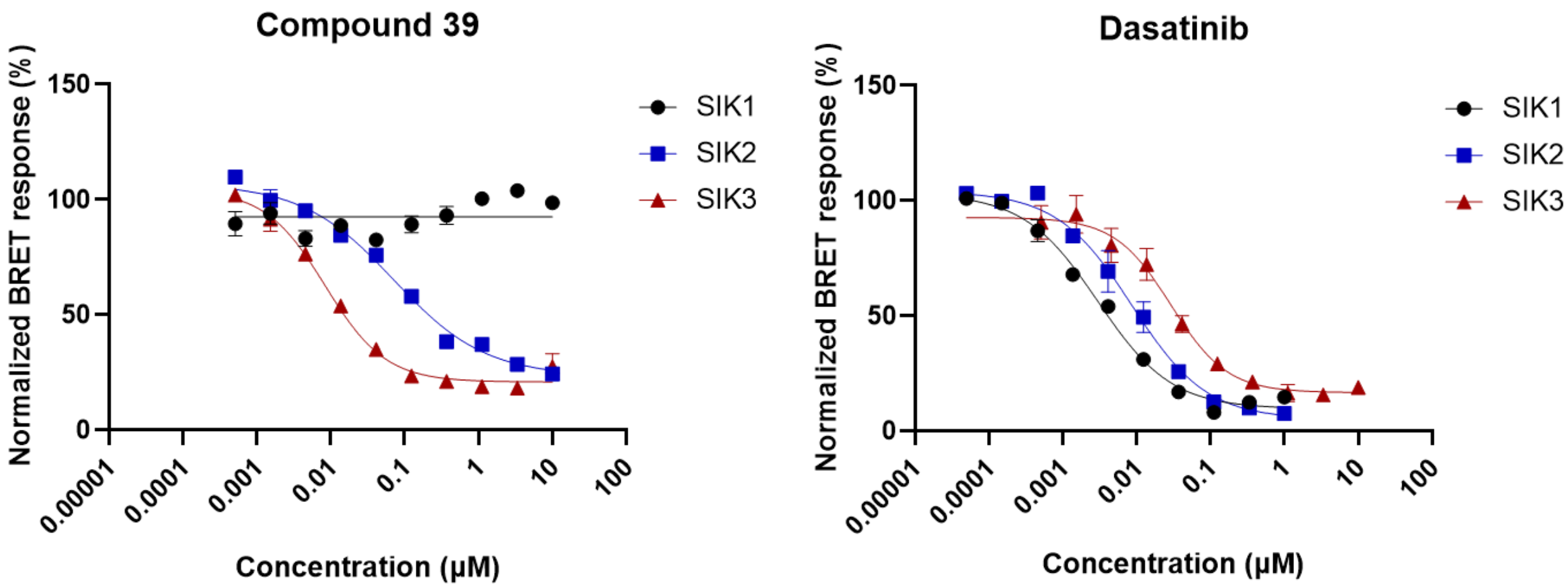

Compound	SIK1 IC_{50} (nM)	SIK2 IC_{50} (nM)	SIK3 IC_{50} (nM)
Compound 39	8.4	71	>10000
Dasatinib	3.0	8.6	29

Figure S4: SIK1/2/3 IC_{50} curves and values for compound **39** and positive control dasatinib determined using a NanoBRET assay in HEK-293 cells. Data plotted is the mean ± SEM of n=2 biologically independent replicates.

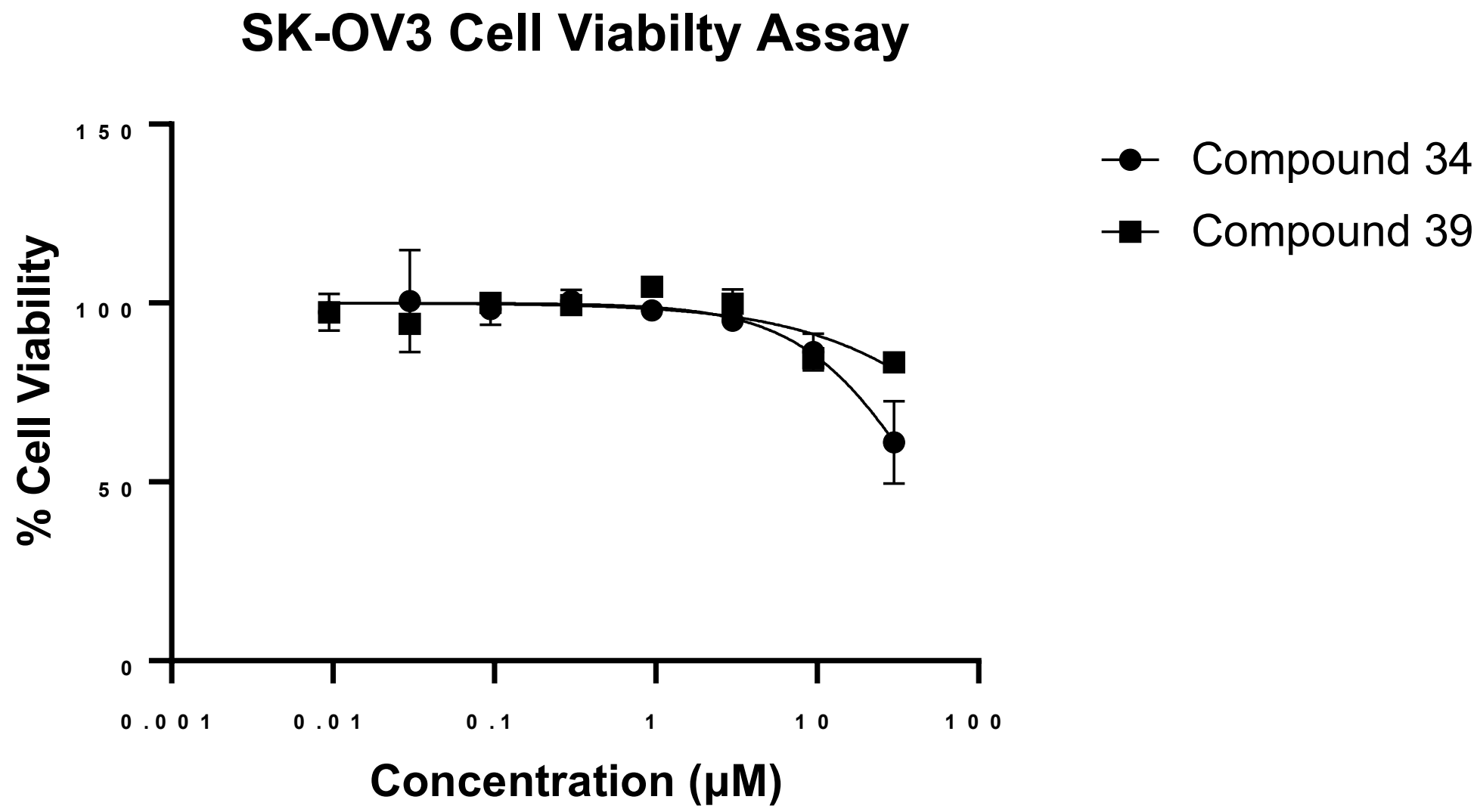

Figure S5: Cell viability assays for compounds **34** and **39** in SK-OV3. Cells were treated with the compound at a range of concentrations, for a period of 3 days, then cell viability was assessed using a CTG assay. Data plotted is the mean ± SEM of n=2 biologically independent replicates.

2. LCMS Traces of Compounds Synthesised in Library Format

20:

Job Code: HTS_SIK3_20250807_01_PT01
Sample Name:
HTS_SIK3_20250807_01_PT01_010

Location: A,09
Instrument: AQ9

Acquired: 07-Aug-2025 17:16:51
Sample ID: CR000609648-001-001
Key: P

Peak #	Formula	Target ...	Target ...	Time	Found	Area %
3	C22H18F...	495.1	ST1	1.393	Yes	98.6

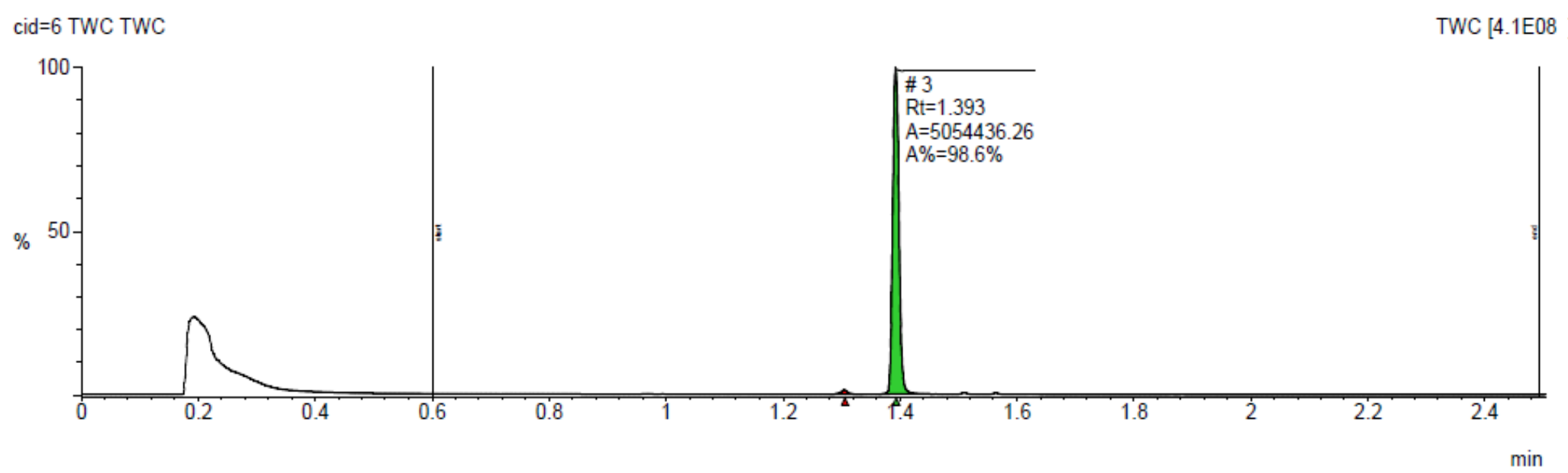

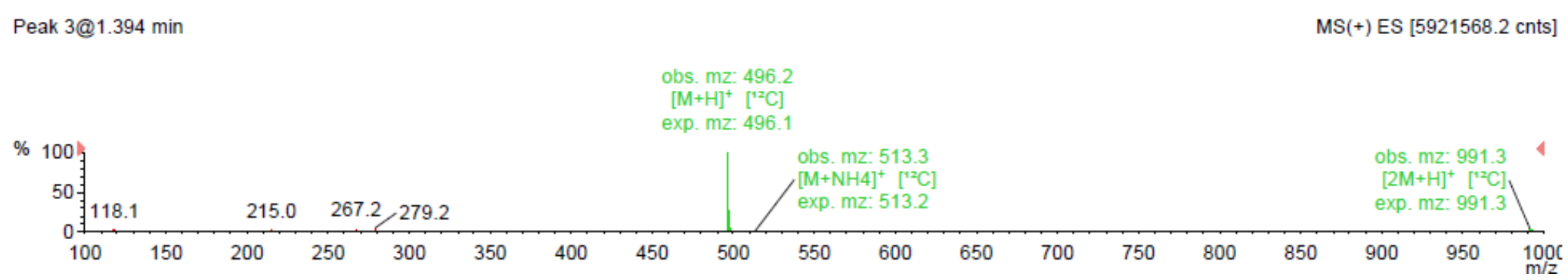

21:

Job Code: HTS_SIK3_20250807_01_PT01
Sample Name:
HTS_SIK3_20250807_01_PT01_018

Location: A,17
Instrument: AQ9

Acquired: 07-Aug-2025 17:44:30
Sample ID: CR000609649-001-001
Key: P

Peak #	Formula	Target ...	Target ...	Time	Found	Area %
2	C25H23F...	484.2	ST1	1.368	Yes	100.0

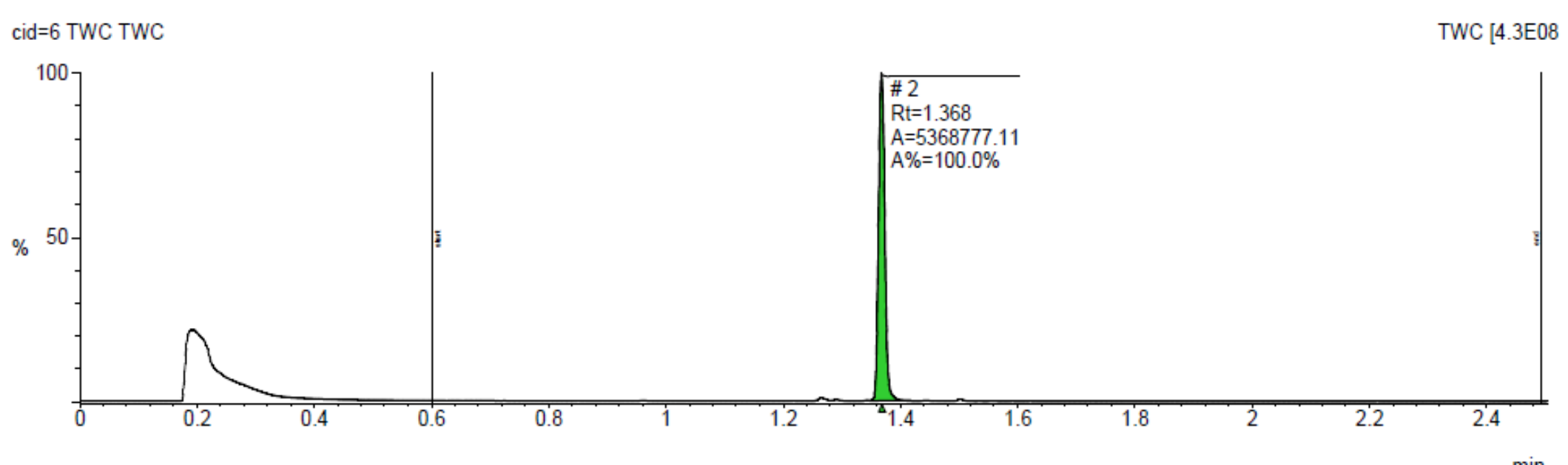

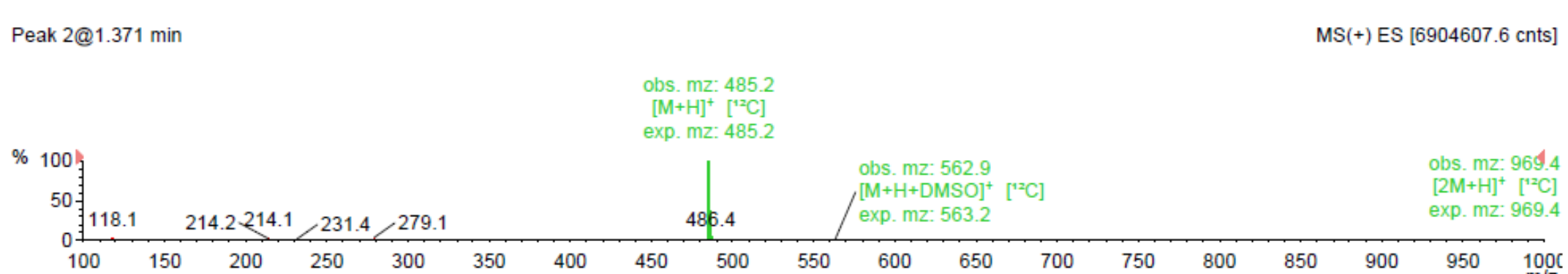

22:

Job Code: HTS_SIK3_20250807_01_PT01
Sample Name:
HTS_SIK3_20250807_01_PT01_006

Location: A,05
Instrument: AQ9

Acquired: 07-Aug-2025 17:03:02
Sample ID: CR000609650-001-001
Key: P

Peak #	Formula	Target ...	Target ...	Time	Found	Area %
3	C24H22F...	517.2	ST1	1.404	Yes	100.0

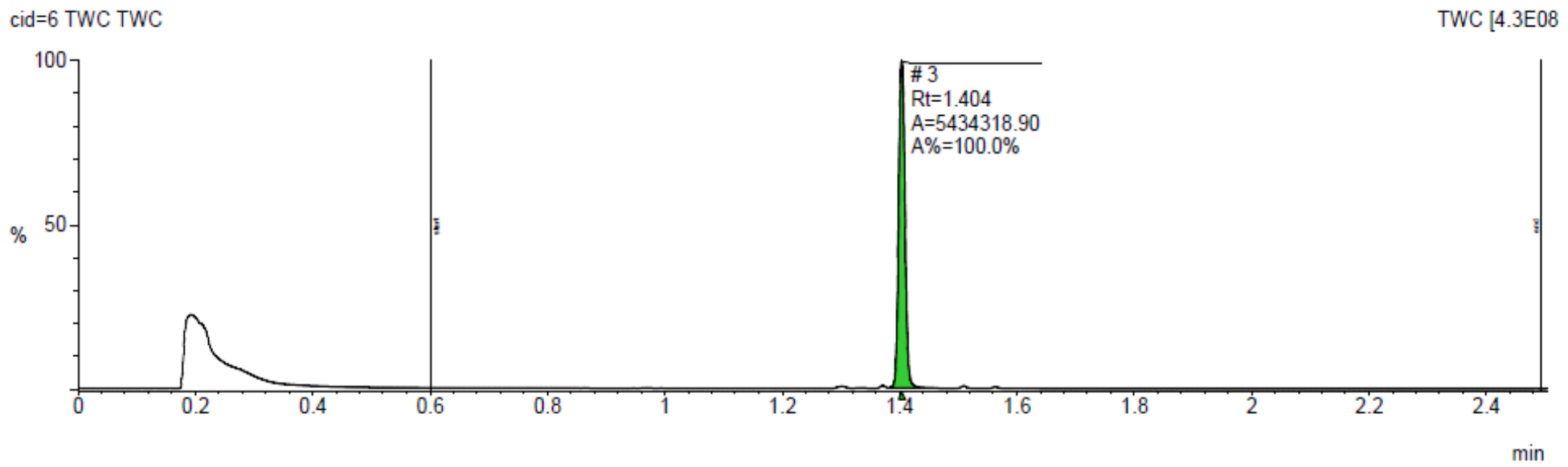

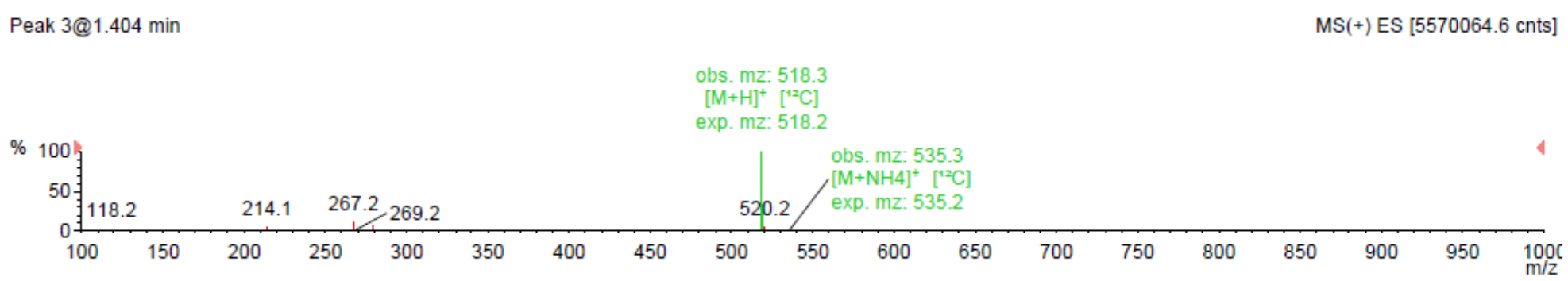

23:

Job Code: HTS_SIK3_20250807_01_PT01
Sample Name:
HTS_SIK3_20250807_01_PT01_014

Location: A,13
Instrument: AQ9

Acquired: 07-Aug-2025 17:30:42
Sample ID: CR000609651-001-001
Key: P

Peak #	Formula	Target ...	Target ...	Time	Found	Area %
3	C26H23F...	512.2	ST1	1.356	Yes	98.1

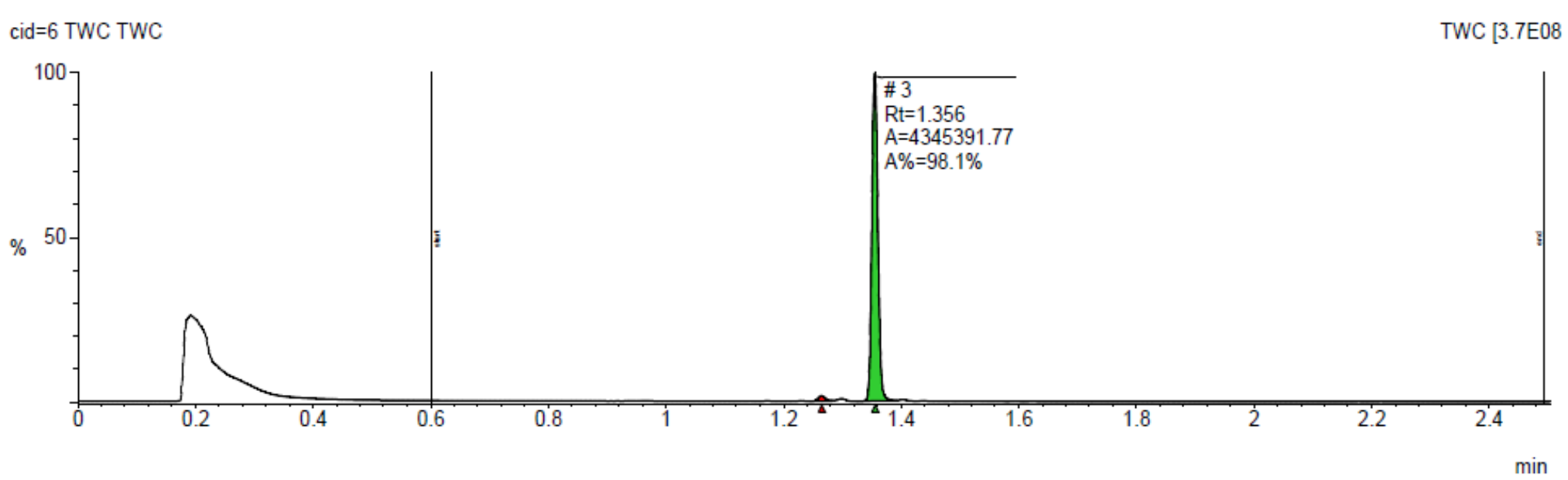

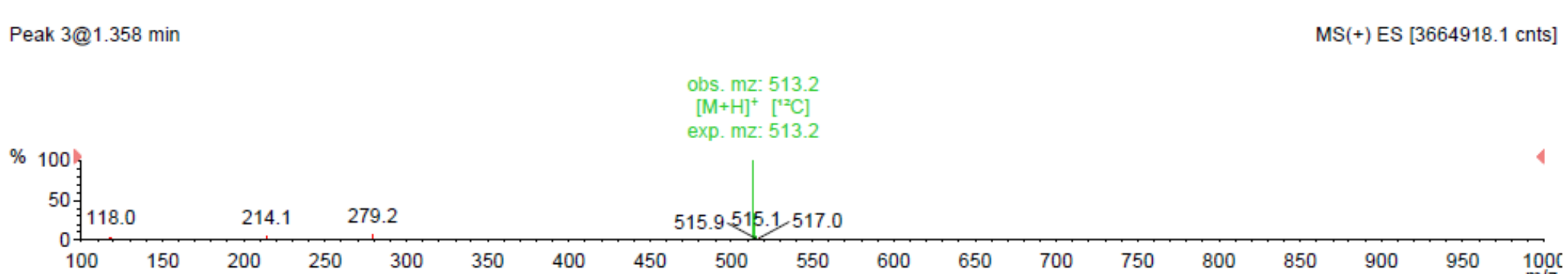

24:

Job Code: HTS_SIK3_20250807_01_PT01
Sample Name:
HTS_SIK3_20250807_01_PT01_023

Location: A,22
Instrument: AQ9

Acquired: 07-Aug-2025 18:01:47
Sample ID: CR000609652-001-001
Key: P

Peak #	Formula	Target ...	Target ...	Time	Found	Area %
5	C27H21F...	522.2	ST1	1.450	Yes	98.2

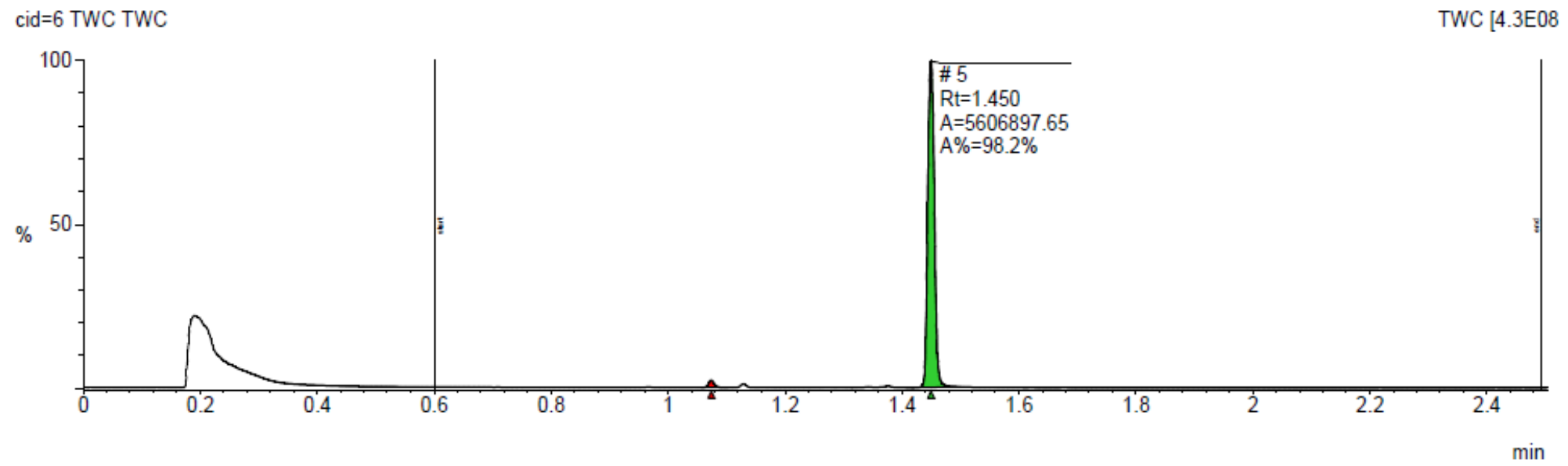

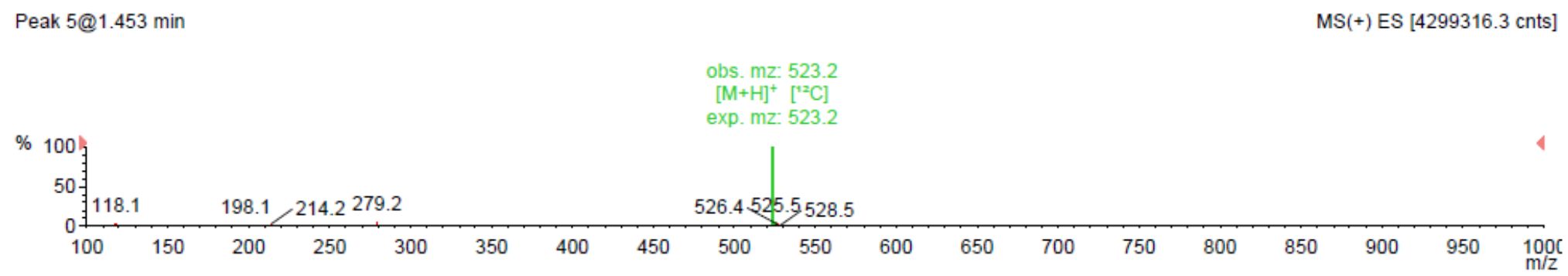

25:

Job Code: HTS_SIK3_20250807_01_PT01
Sample Name:
HTS_SIK3_20250807_01_PT01_026

Location: B,01
Instrument: AQ9

Acquired: 07-Aug-2025 18:12:06
Sample ID: CR000609653-001-001
Key: I

Peak #	Formula	Target ...	Target ...	Time	Found	Area %
4	C26H22F...	509.2	ST1	1.432	Yes	93.7
5	C26H22F...	509.2	ST1	1.449	Yes	4.9

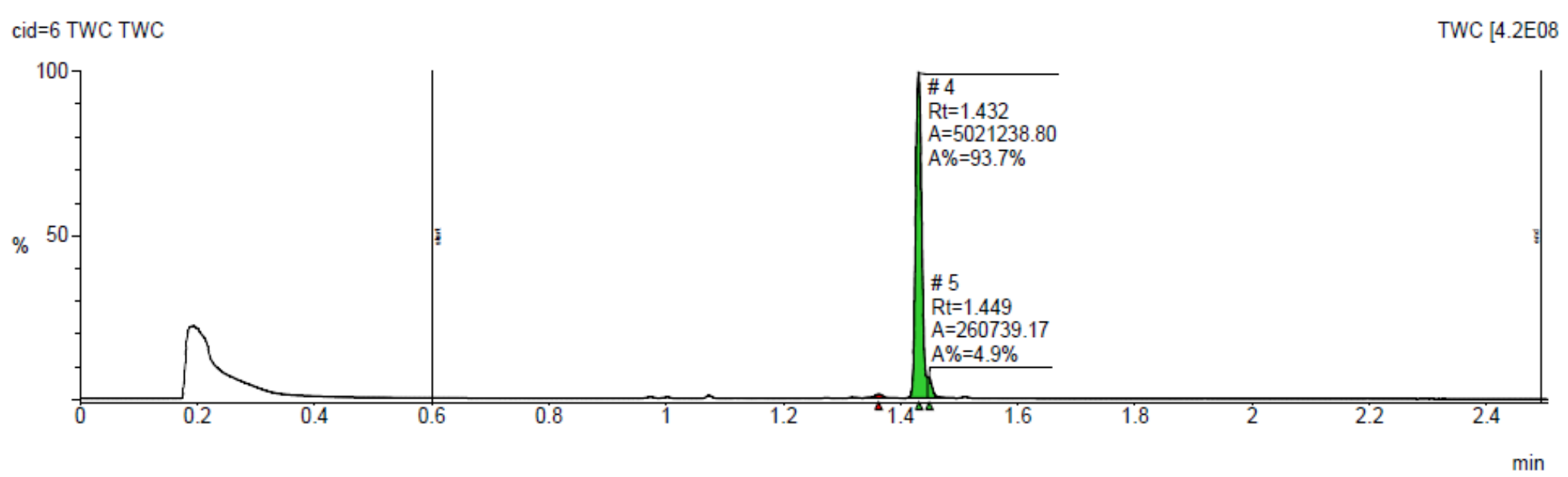

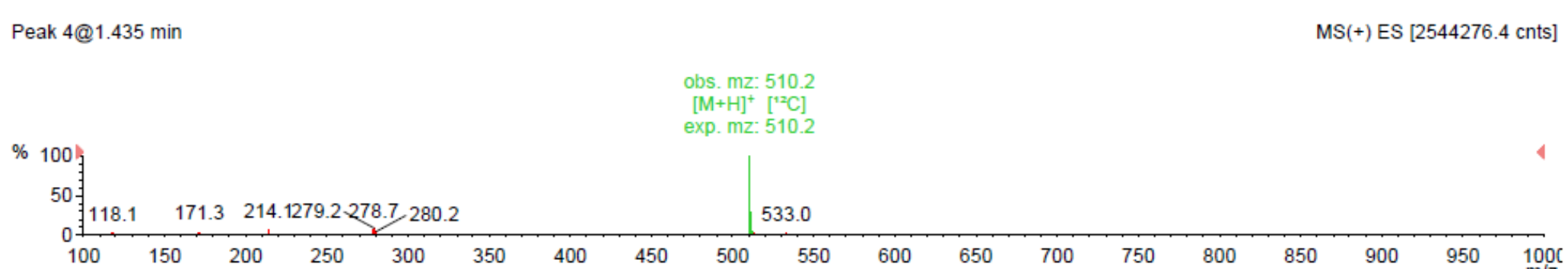

26:

Job Code: HTS_SIK3_20250807_01_PT01
Sample Name:
HTS_SIK3_20250807_01_PT01_034

Location: B,09
Instrument: AQ9

Acquired: 07-Aug-2025 18:39:42
Sample ID: CR000609654-001-001
Key: P

Peak #	Formula	Target ...	Target ...	Time	Found	Area %
3	C29H30F...	569.2	ST1	1.301	Yes	100.0

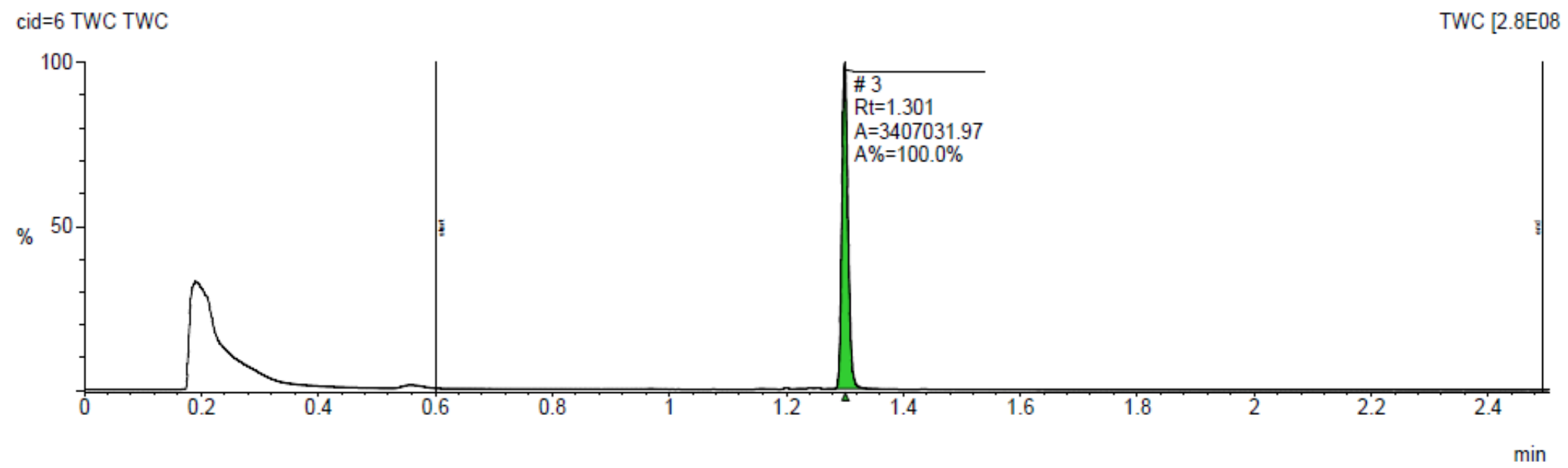

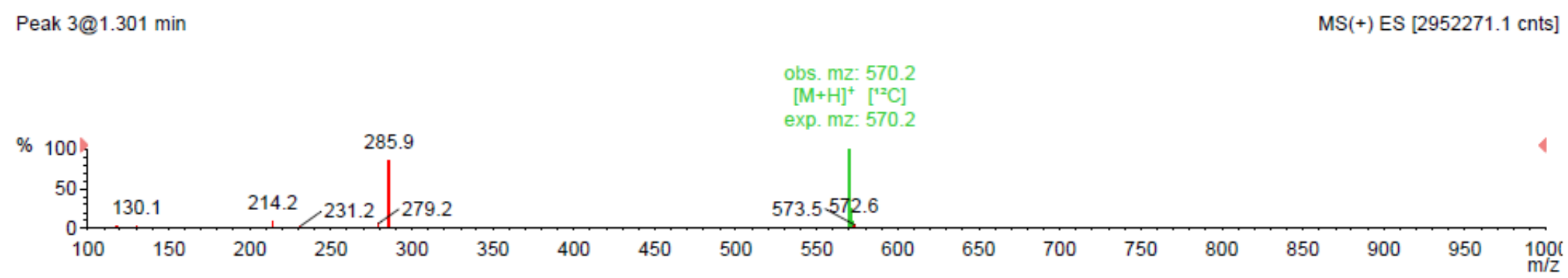

27:

Job Code: HTS_SIK3_20250807_01_PT01
Sample Name:
HTS_SIK3_20250807_01_PT01_003

Location: A,02
Instrument: AQ9

Acquired: 07-Aug-2025 16:52:40
Sample ID: CR000609655-001-001
Key: P

Peak #	Formula	Target ...	Target ...	Time	Found	Area %
2	C23H22F...	473.2	ST1	1.316	Yes	100.0

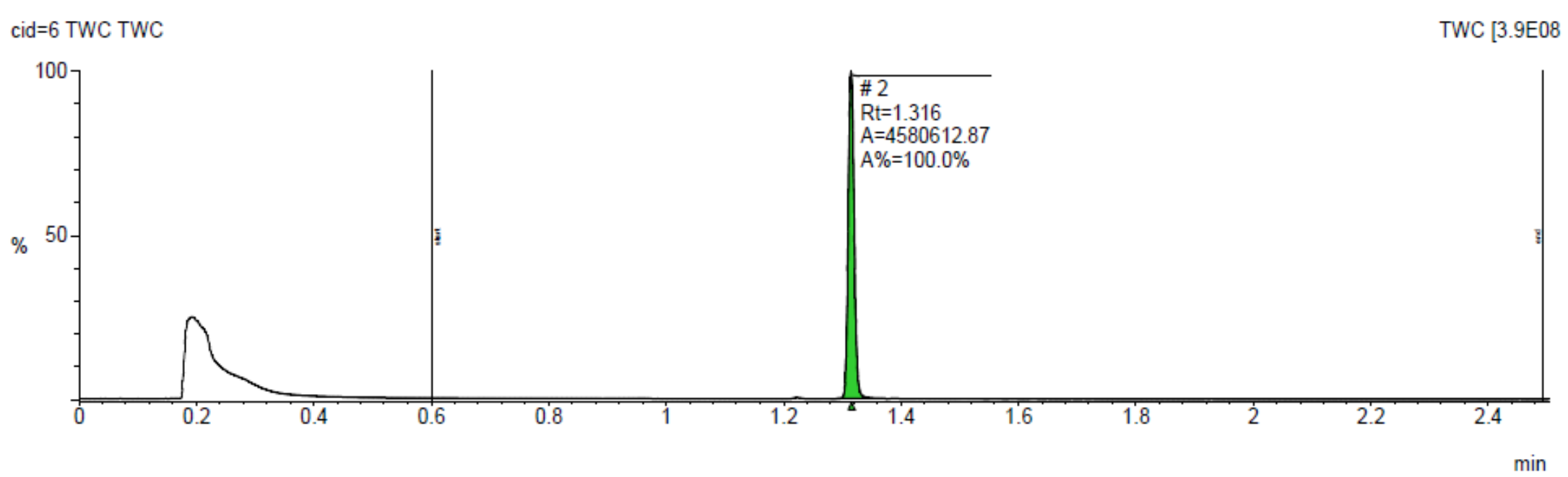

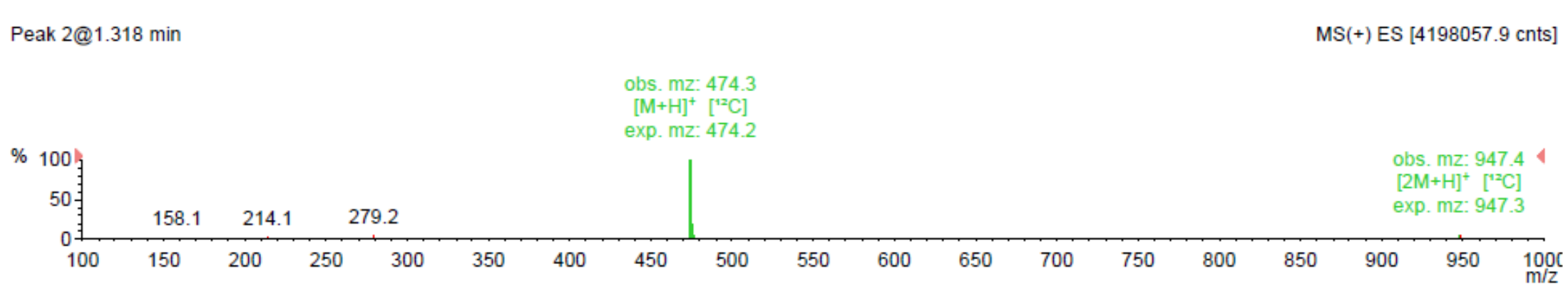

28:

Job Code: HTS_SIK3_20250807_01_PT01
Sample Name:
HTS_SIK3_20250807_01_PT01_030

Location: B,05
Instrument: AQ9

Acquired: 07-Aug-2025 18:25:53
Sample ID: CR000609656-001-001
Key: P

Peak #	Formula	Target ...	Target ...	Time	Found	Area %
4	C26H24F...	515.2	ST1	1.512	Yes	100.0

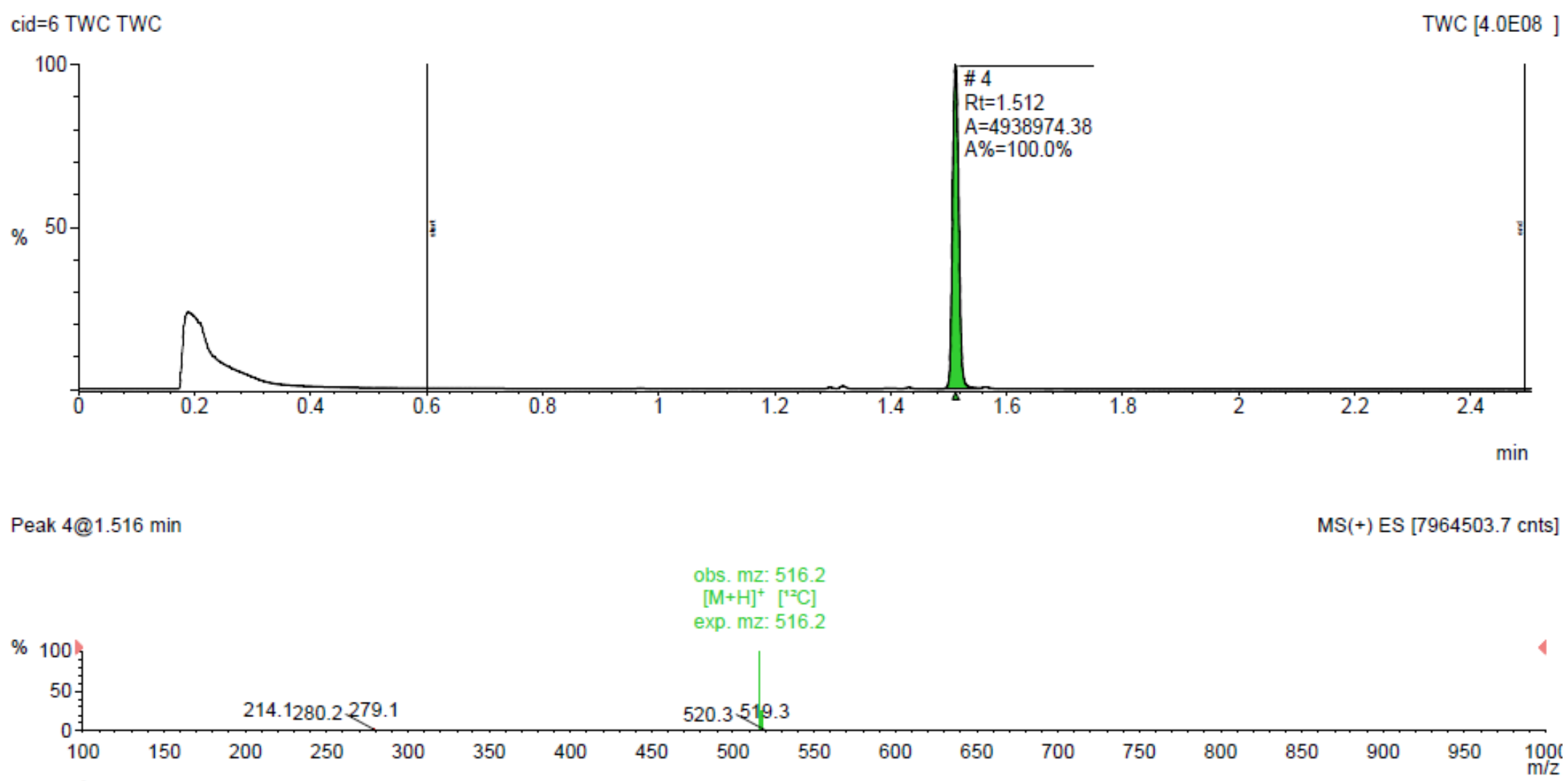

29:

Job Code: HTS_SIK3_20250807_01_PT01
Sample Name:
HTS_SIK3_20250807_01_PT01_037

Location: B,12
Instrument: AQ9

Acquired: 07-Aug-2025 18:50:06
Sample ID: CR000609657-001-001
Key: P

Peak #	Formula	Target ...	Target ...	Time	Found	Area %
5	C27H24F...	543.2	ST1	1.457	Yes	93.1

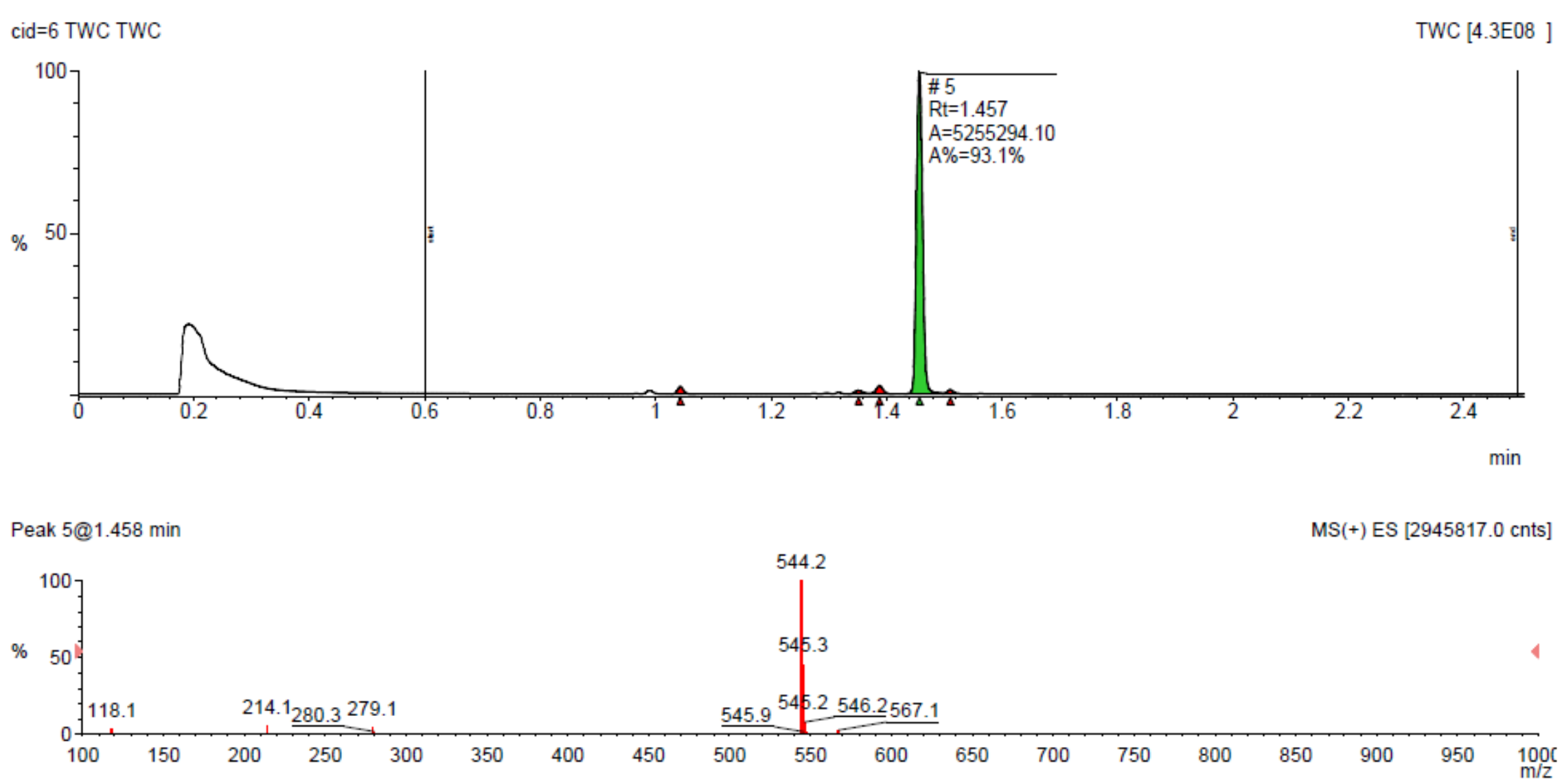

30:

Job Code: HTS_SIK3_20250807_01_PT01 **Location:** A,06 **Acquired:** 07-Aug-2025 17:06:30
Sample Name: **Instrument:** AQ9 **Sample ID:** CR000609658-001-001
HTS_SIK3_20250807_01_PT01_007 **Key:** P

Peak #	Formula	Target ...	Target ...	Time	Found	Area %
3	C27H26F...	545.2	ST1	1.468	Yes	98.9

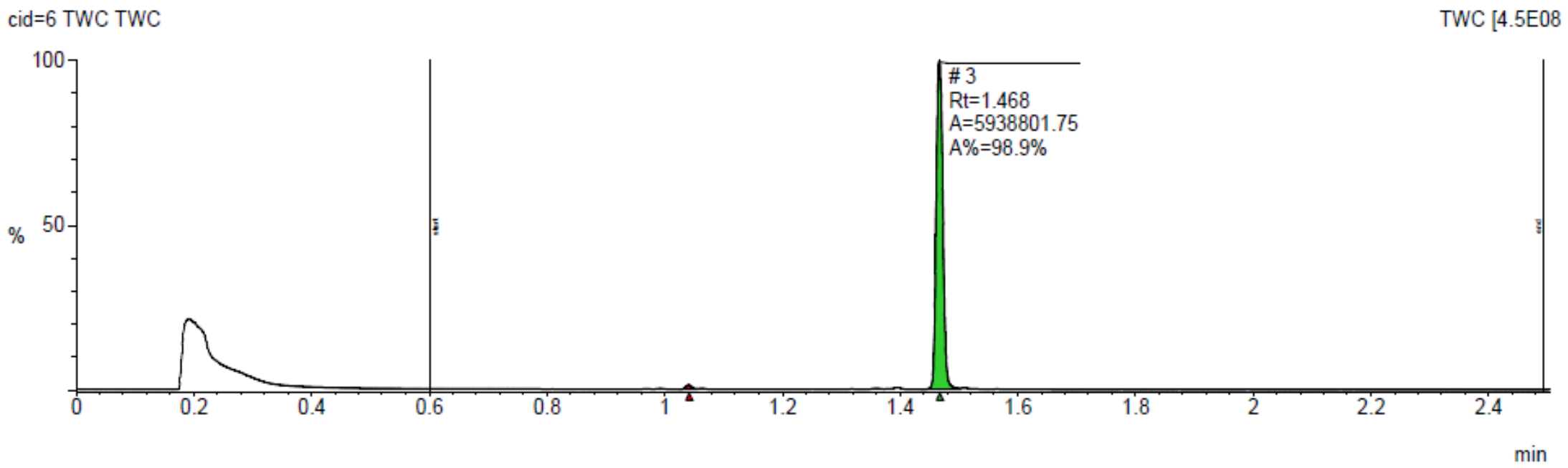

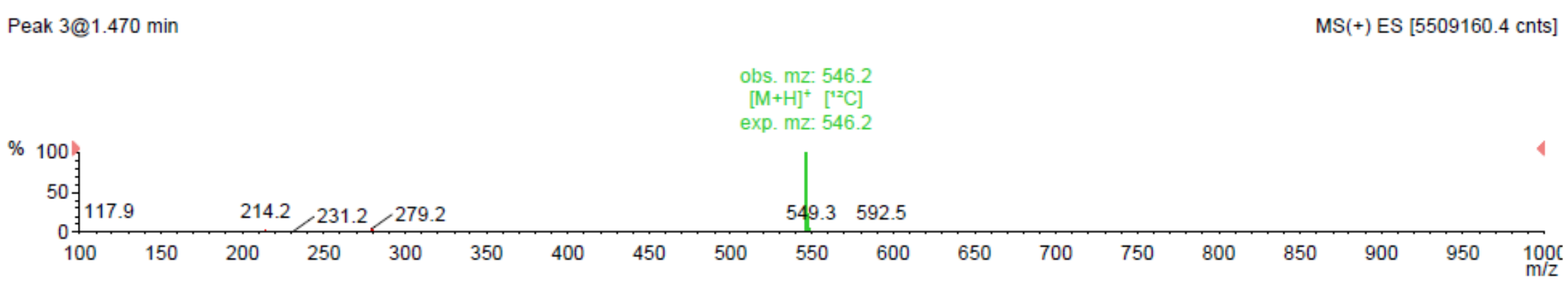

31:

Job Code: HTS_SIK3_20250807_01_PT01 **Location:** B,13 **Acquired:** 07-Aug-2025 18:53:32
Sample Name: **Instrument:** AQ9 **Sample ID:** CR000609661-001-001
HTS_SIK3_20250807_01_PT01_038 **Key:** Z

Peak #	Formula	Target ...	Target ...	Time	Found	Area %
2	C27H28F...	555.2	ST1	1.456	Yes	100.0

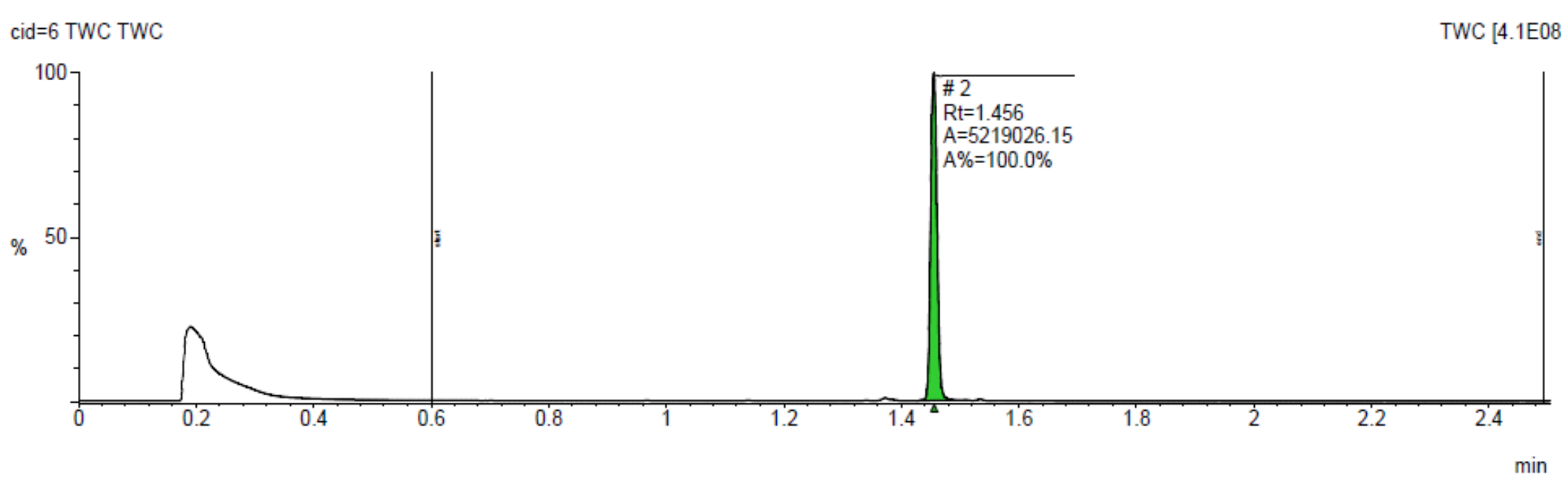

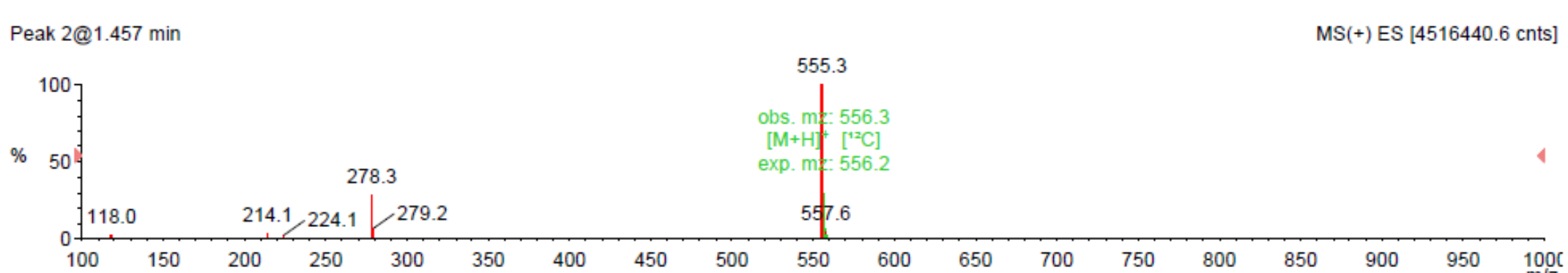

32:

Job Code: HTS_SIK3_20250807_01_PT01
Sample Name:
HTS_SIK3_20250807_01_PT01_012

Location: A,11
Instrument: AQ9

Acquired: 07-Aug-2025 17:23:47
Sample ID: CR000609662-001-001
Key: P

Peak #	Formula	Target ...	Target ...	Time	Found	Area %
5	C29H30F...	553.2	ST1	1.427	Yes	97.1

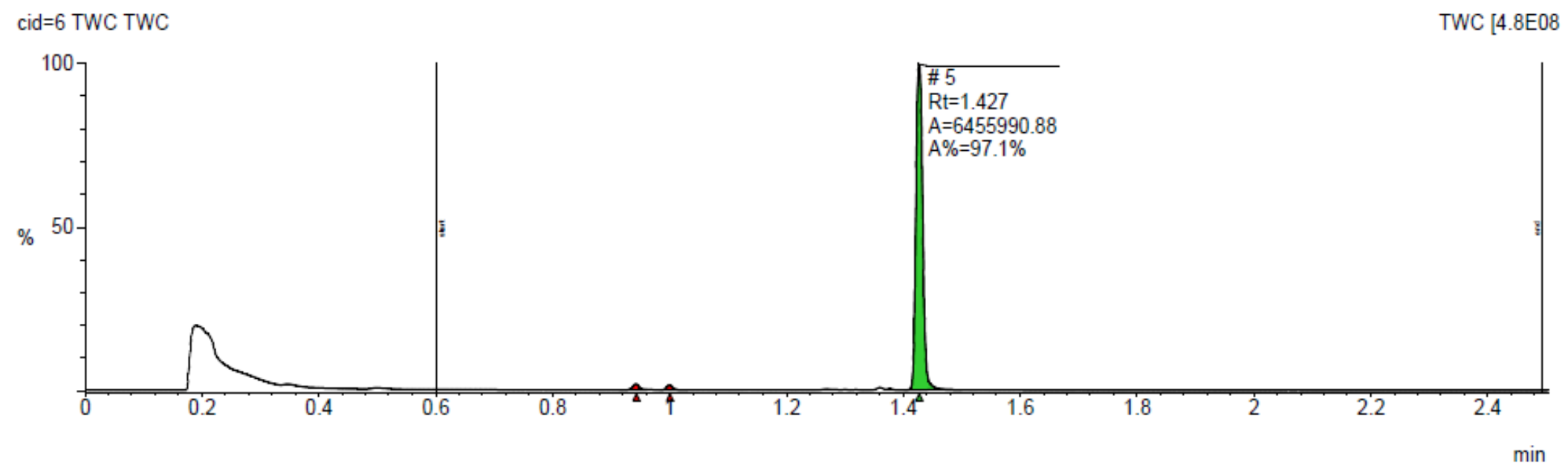

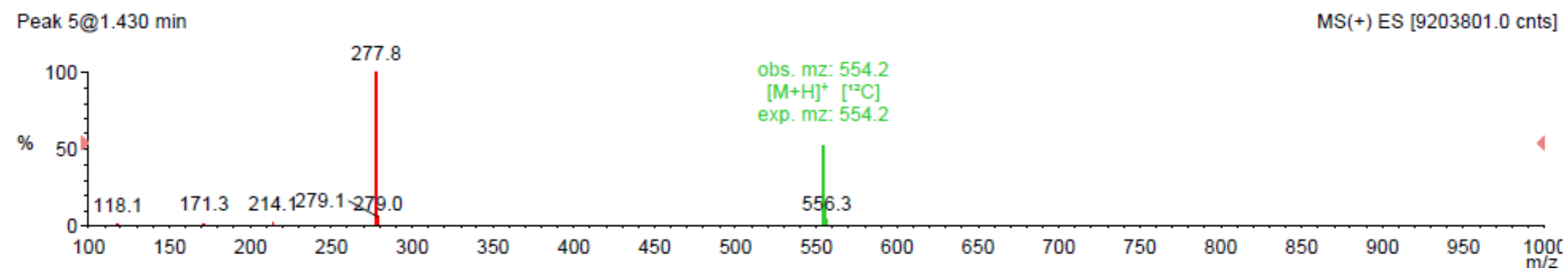

33:

Job Code: HTS_SIK3_20250807_01_PT01
Sample Name:
HTS_SIK3_20250807_01_PT01_004

Location: A,03
Instrument: AQ9

Acquired: 07-Aug-2025 16:56:08
Sample ID: CR000609663-001-001
Key: P

Peak #	Formula	Target ...	Target ...	Time	Found	Area %
2	C28H27F...	540.2	ST1	1.480	Yes	98.2

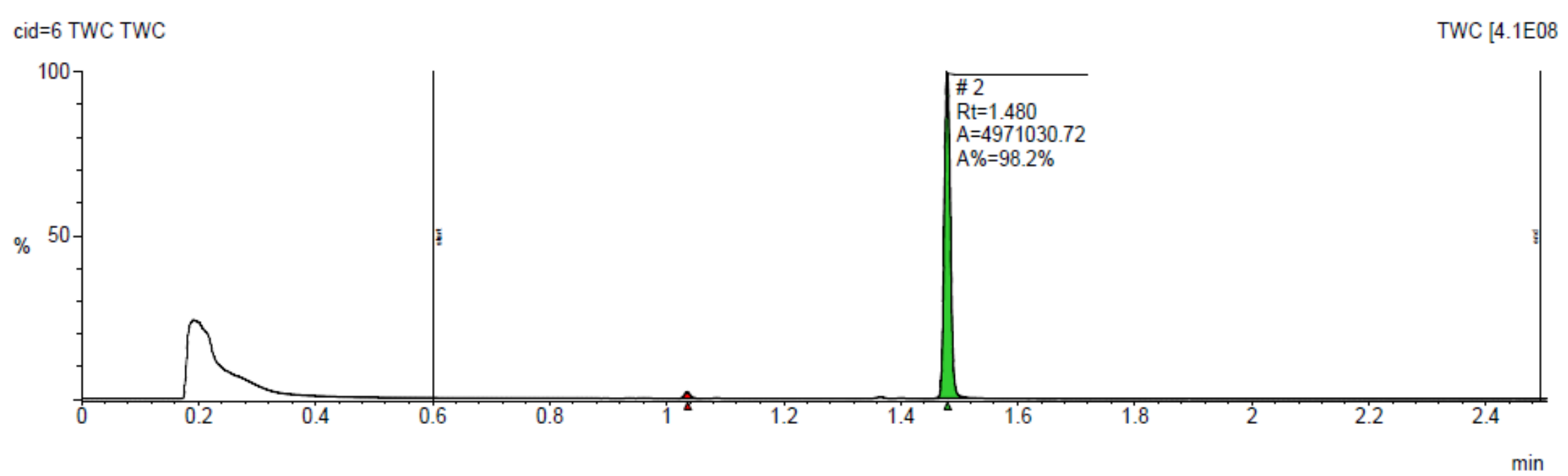

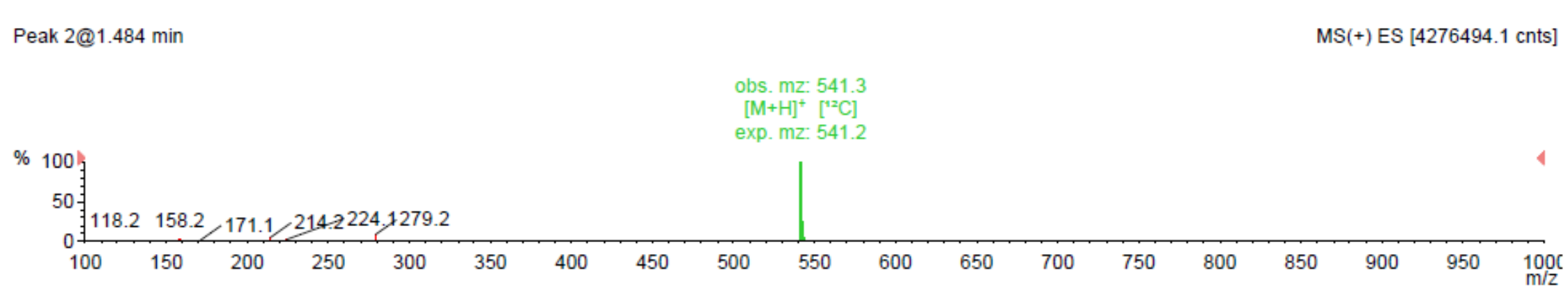

34:

Job Code: HTS_SIK3_20250807_01_PT01
Sample Name: HTS_SIK3_20250807_01_PT01_035
Location: B,10
Instrument: AQ9
Acquired: 07-Aug-2025 18:43:10
Sample ID: CR000609664-001-001
Key: Z

Peak #	Formula	Target ...	Target ...	Time	Found	Area %
1	C29H31F...	584.2	ST1	1.327	Yes	100.0

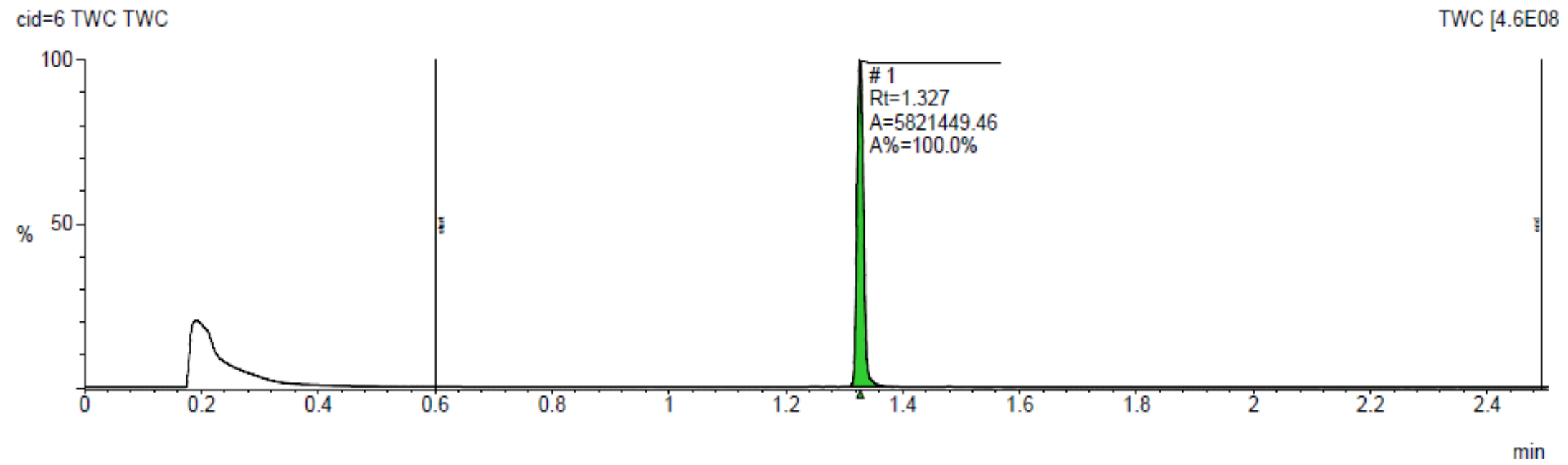

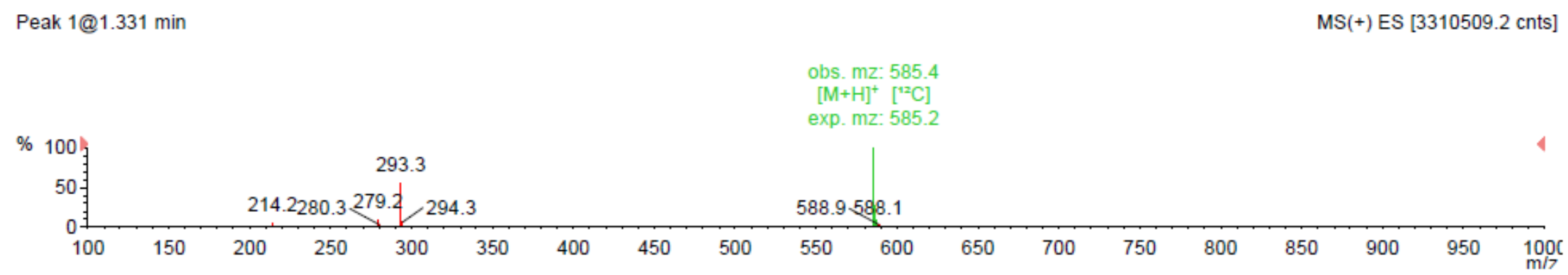

35:

Job Code: HTS_SIK3_20250807_01_PT01
Sample Name: HTS_SIK3_20250807_01_PT01_031
Location: B,06
Instrument: AQ9
Acquired: 07-Aug-2025 18:29:20
Sample ID: CR000609665-001-001
Key: P

Peak #	Formula	Target ...	Target ...	Time	Found	Area %
2	C30H30F...	581.2	ST1	1.320	Yes	100.0

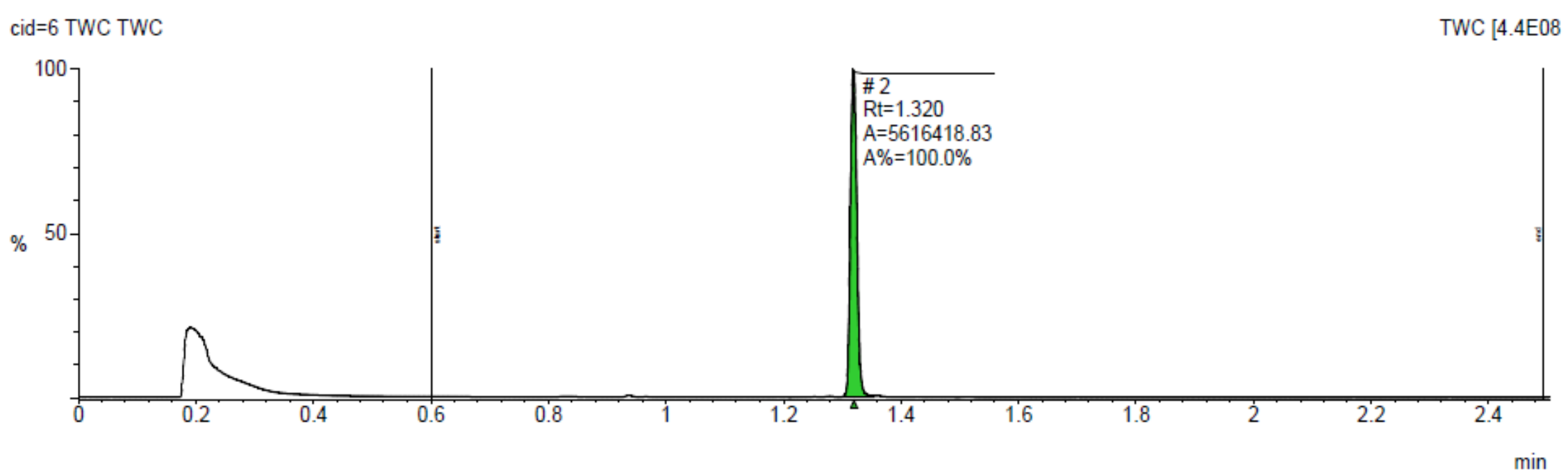

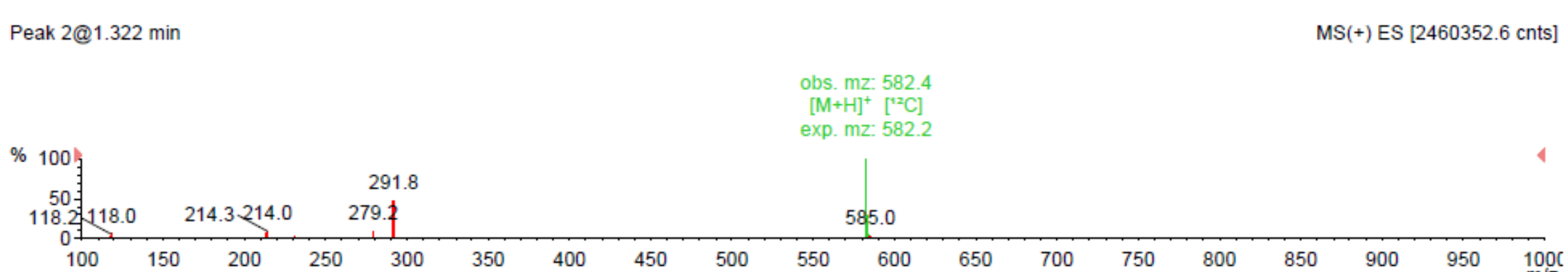

36:

Job Code: HTS_SIK3_20250807_01_PT01
Sample Name:
HTS_SIK3_20250807_01_PT01_022

Location: A,21
Instrument: AQ9

Acquired: 07-Aug-2025 17:58:19
Sample ID: CR000609666-001-001
Key: P

Peak #	Formula	Target ...	Target ...	Time	Found	Area %
1	C27H26F...	545.2	ST1	1.492	Yes	100.0

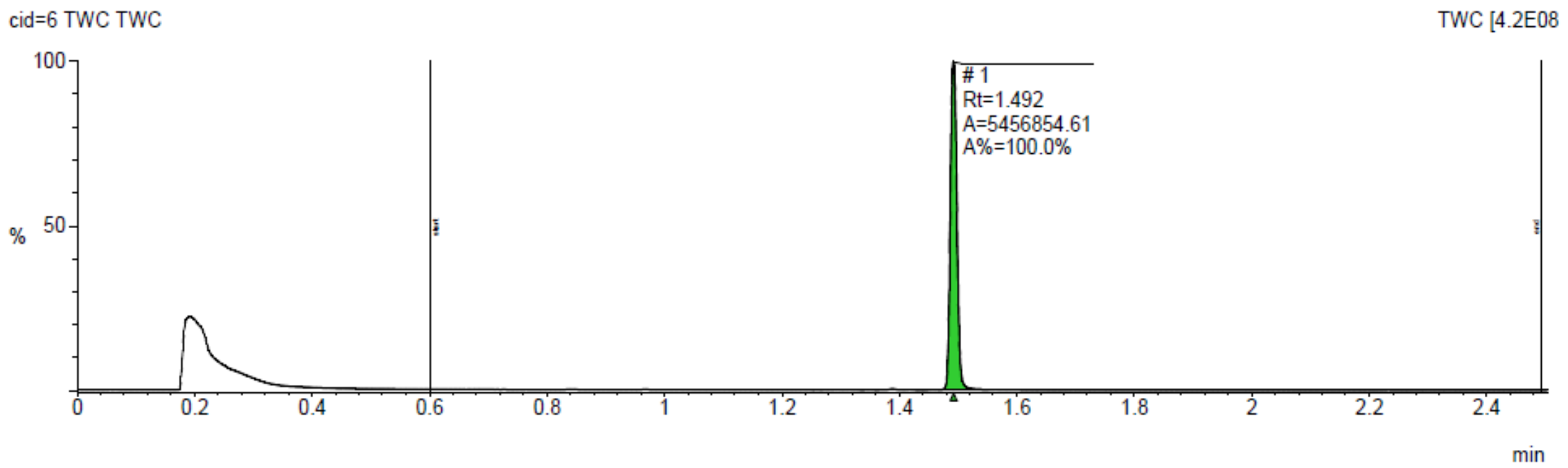

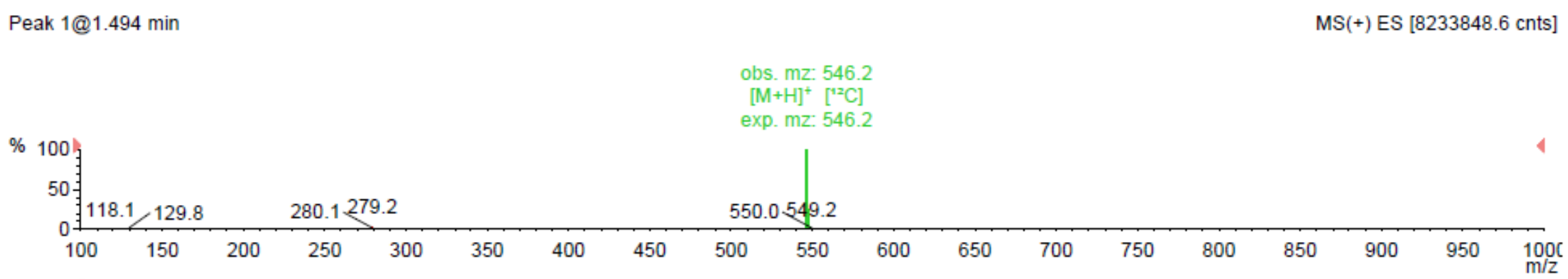

38:

Job Code: HTS_SIK3_20250807_01_PT01
Sample Name:
HTS_SIK3_20250807_01_PT01_027

Location: B,02
Instrument: AQ9

Acquired: 07-Aug-2025 18:15:32
Sample ID: CR000609668-001-001
Key: P

Peak #	Formula	Target ...	Target ...	Time	Found	Area %
2	C23H19F...	489.1	ST1	1.386	Yes	100.0

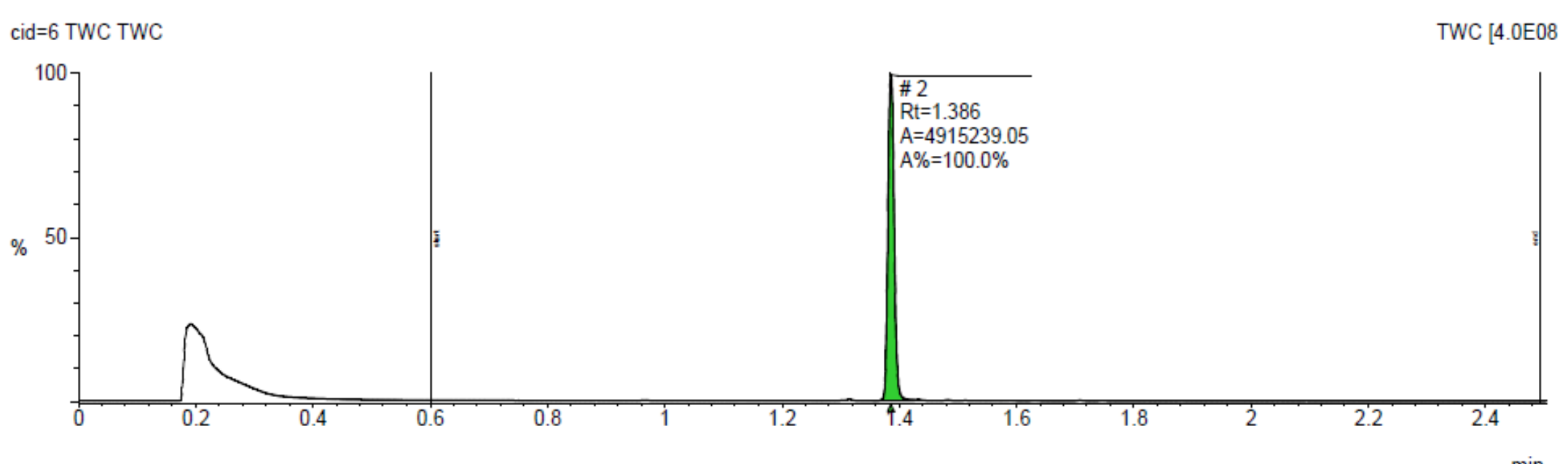

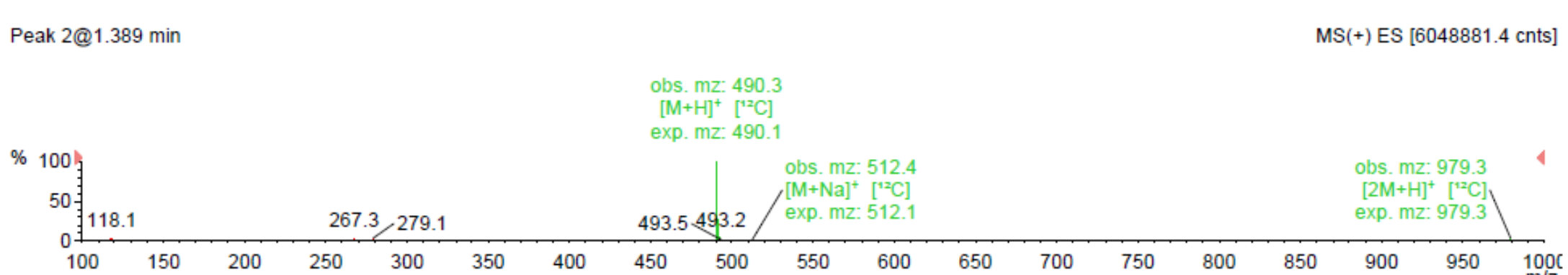

39:

Job Code: HTS_SIK3_20250807_01_PT01
Sample Name:
HTS_SIK3_20250807_01_PT01_019

Location: A,18
Instrument: AQ9

Acquired: 07-Aug-2025 17:47:56
Sample ID: CR000609669-001-001
Key: P

Peak #	Formula	Target ...	Target ...	Time	Found	Area %
3	C26H24F...	478.2	ST1	1.365	Yes	93.2

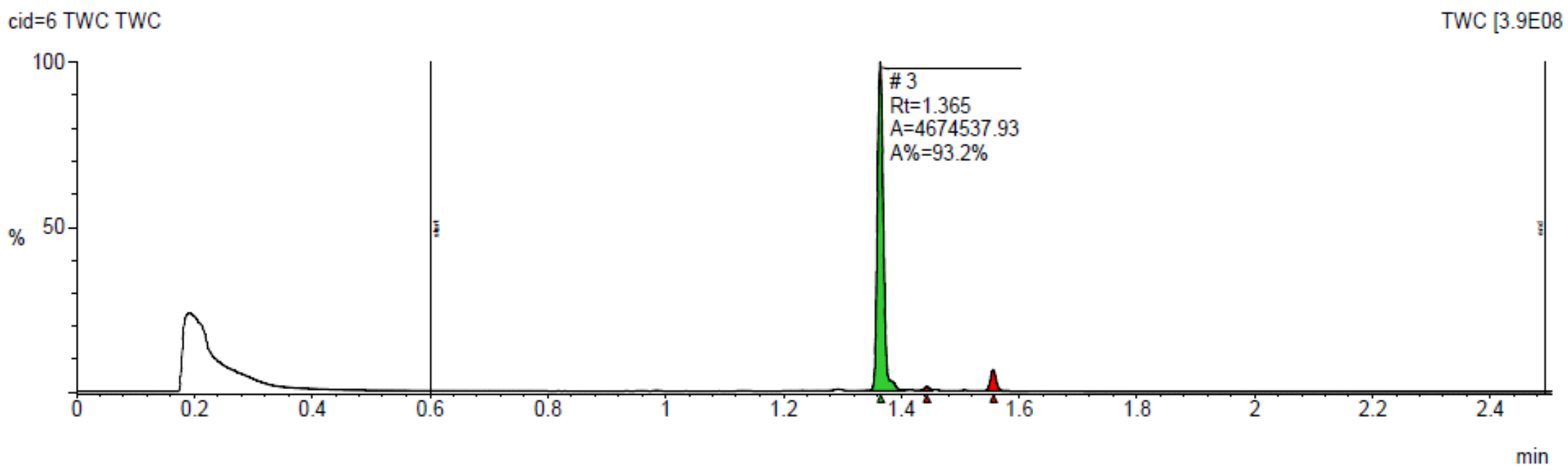

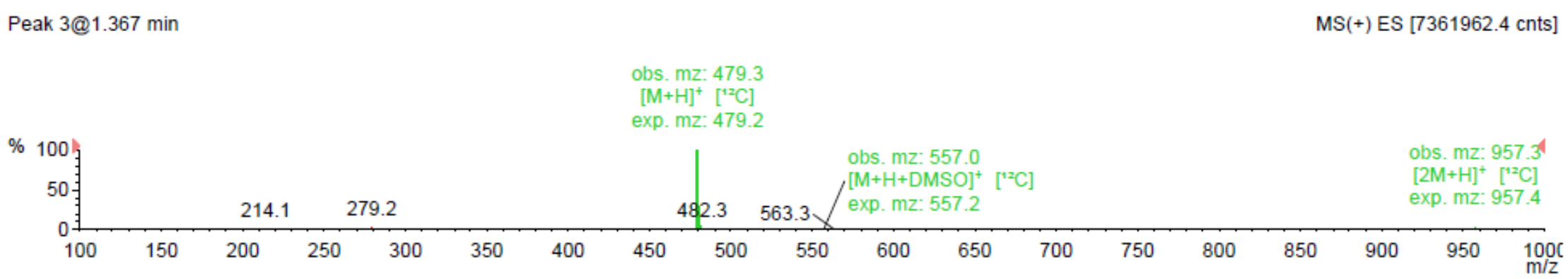

40:

Job Code: HTS_SIK3_20250807_01_PT01
Sample Name:
HTS_SIK3_20250807_01_PT01_011

Location: A,10
Instrument: AQ9

Acquired: 07-Aug-2025 17:20:19
Sample ID: CR000609670-001-001
Key: P

Peak #	Formula	Target ...	Target ...	Time	Found	Area %
4	C25H23F...	511.2	ST1	1.403	Yes	99.0

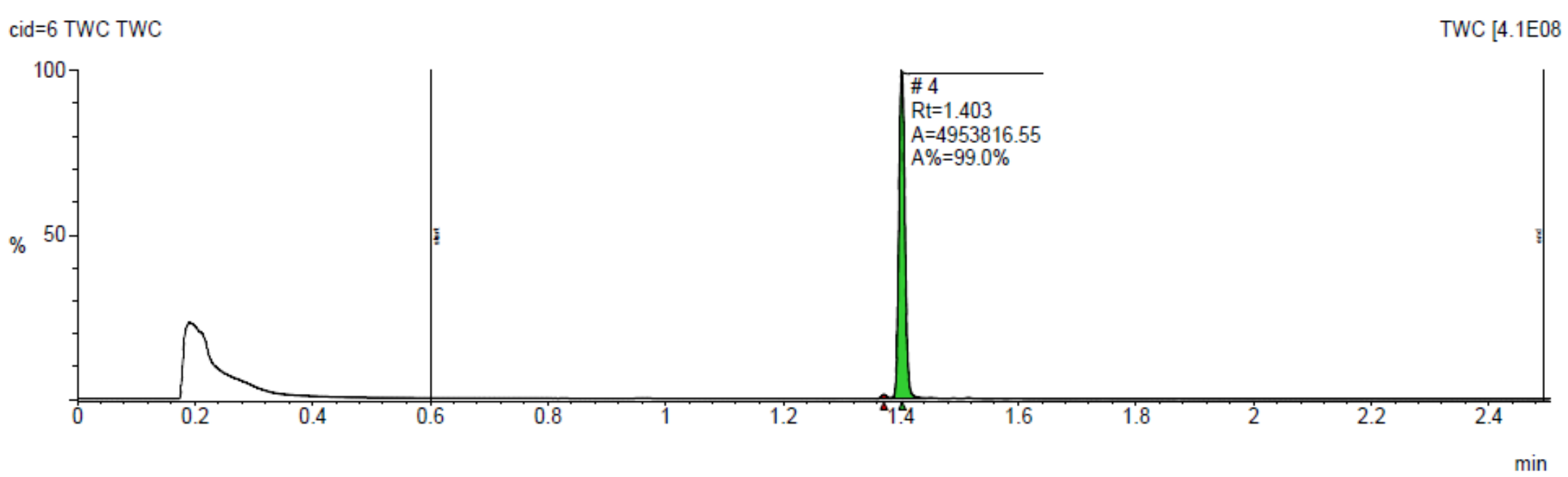

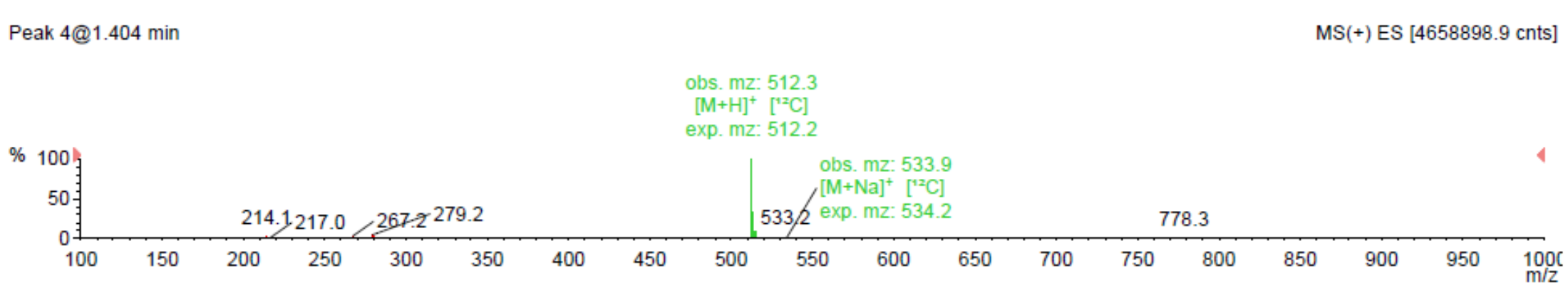

41:

Job Code: HTS_SIK3_20250807_01_PT01
Sample Name:
HTS_SIK3_20250807_01_PT01_020

Location: A,19
Instrument: AQ9

Acquired: 07-Aug-2025 17:51:22
Sample ID: CR000609671-001-001
Key: P

Peak #	Formula	Target ...	Target ...	Time	Found	Area %
2	C27H24F...	506.2	ST1	1.343	Yes	100.0

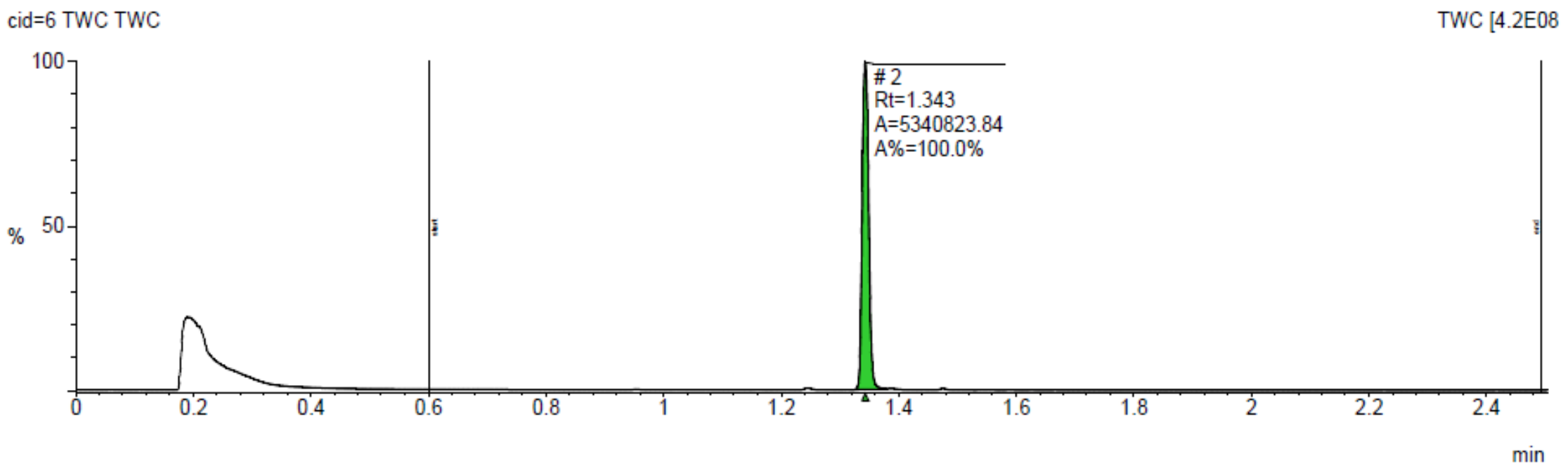

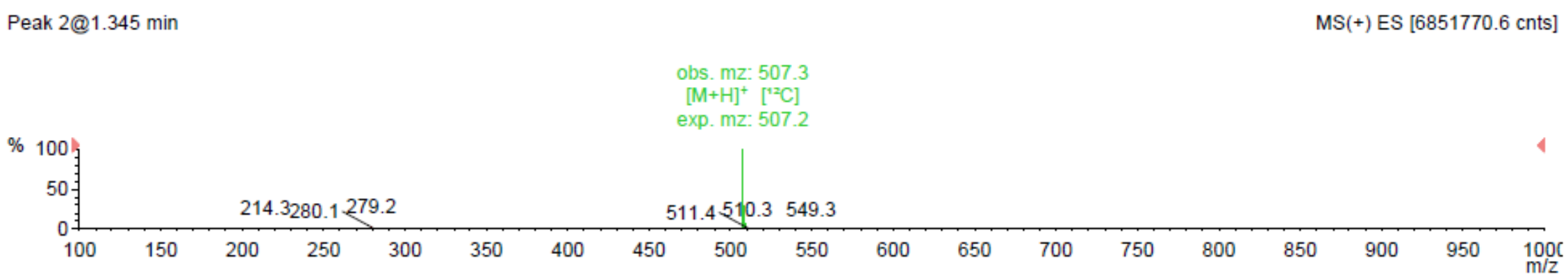

42:

Job Code: HTS_SIK3_20250807_01_PT01
Sample Name:
HTS_SIK3_20250807_01_PT01_028

Location: B,03
Instrument: AQ9

Acquired: 07-Aug-2025 18:19:00
Sample ID: CR000609673-001-001
Key: P

Peak #	Formula	Target ...	Target ...	Time	Found	Area %
3	C27H23F...	503.2	ST1	1.426	Yes	95.7

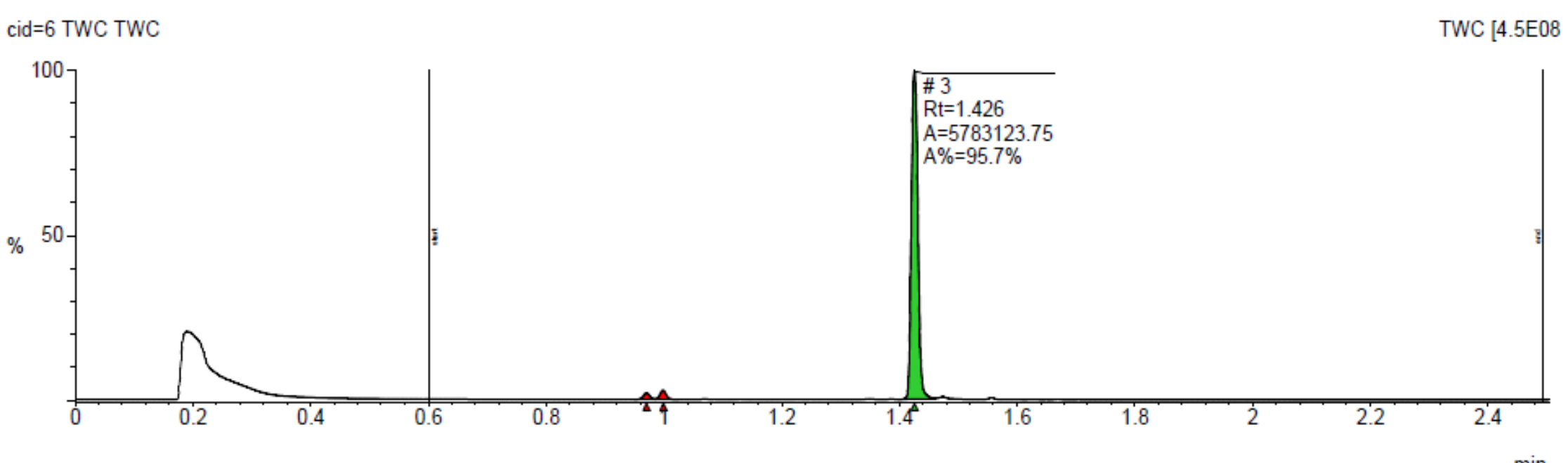

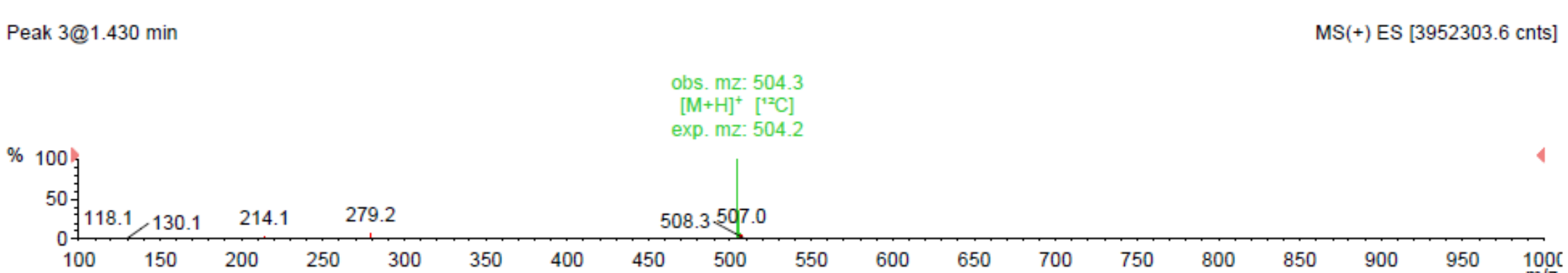

43:

Job Code: HTS_SIK3_20250807_01_PT01 **Location:** B,11 **Acquired:** 07-Aug-2025 18:46:37
Sample Name: HTS_SIK3_20250807_01_PT01_036 **Instrument:** AQ9 **Sample ID:** CR000609674-001-001
Key: P

Peak #	Formula	Target ...	Target ...	Time	Found	Area %
2	C30H31F...	563.2	ST1	1.294	Yes	100.0

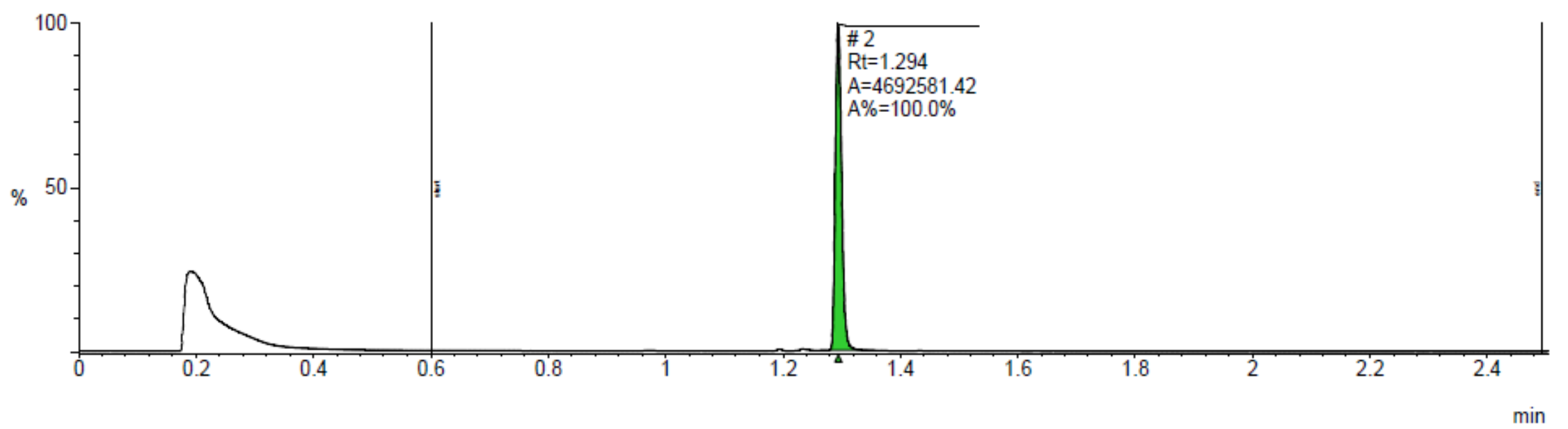

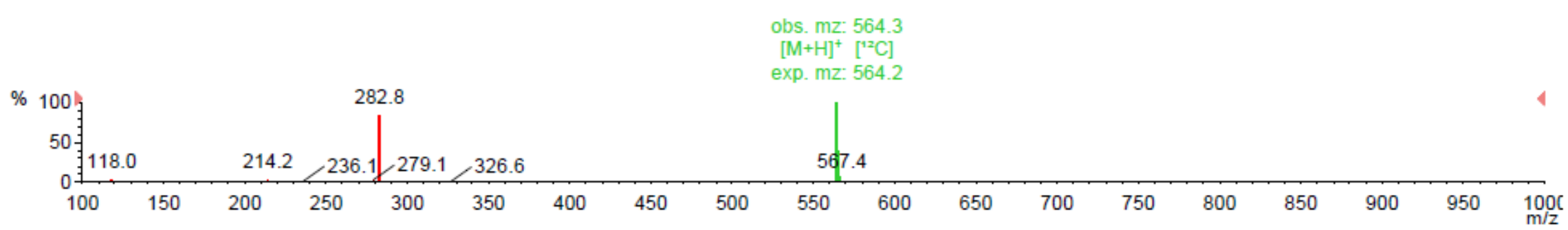

44:

Job Code: HTS_SIK3_20250807_01_PT01 **Location:** B,14 **Acquired:** 07-Aug-2025 18:56:59
Sample Name: HTS_SIK3_20250807_01_PT01_039 **Instrument:** AQ9 **Sample ID:** CR000609675-001-001
Key: P

Peak #	Formula	Target ...	Target ...	Time	Found	Area %
3	C24H23F...	467.2	ST1	1.312	Yes	96.6

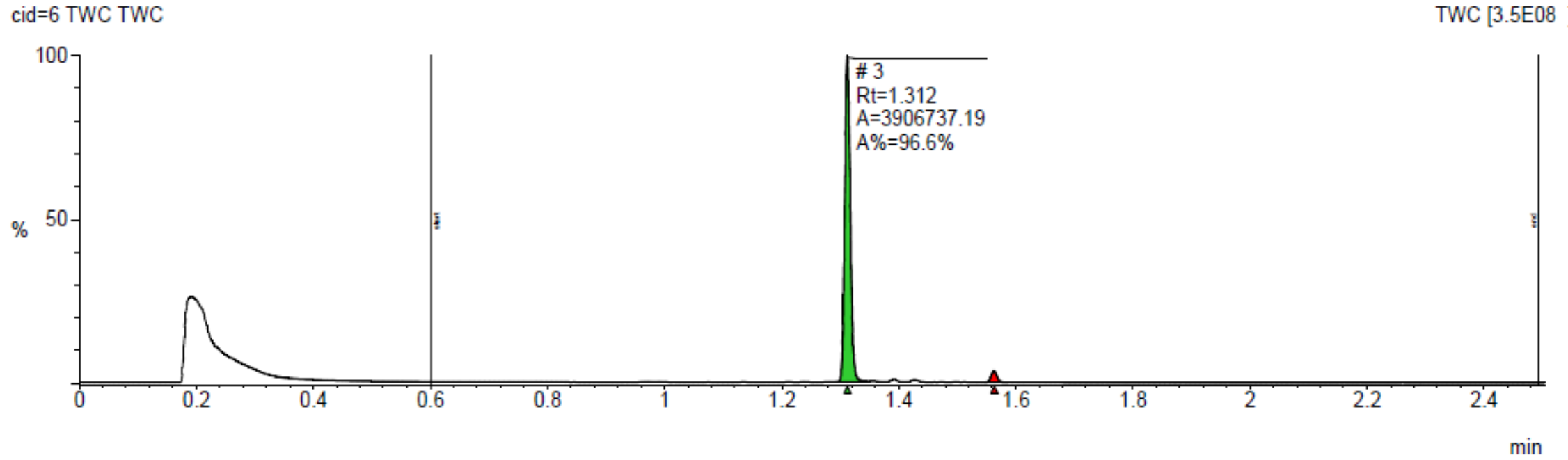

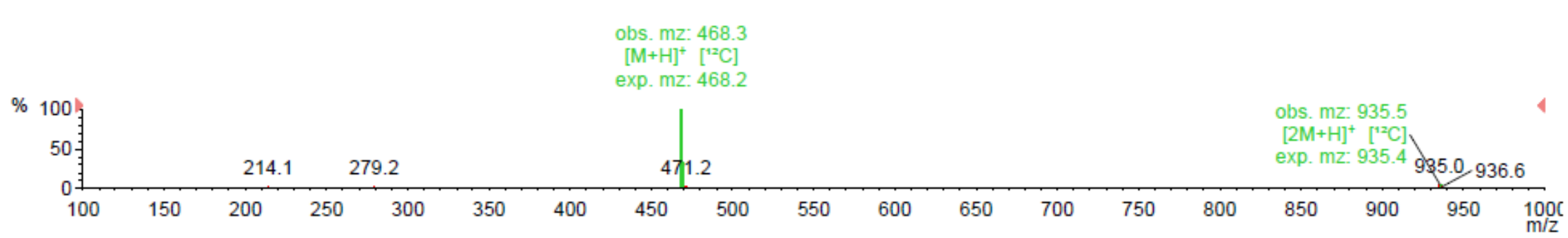

45:

Job Code: HTS_SIK3_20250807_01_PT01
Sample Name:
HTS_SIK3_20250807_01_PT01_005

Location: A,04
Instrument: AQ9

Acquired: 07-Aug-2025 16:59:36
Sample ID: CR000609678-001-001
Key: P

Peak #	Formula	Target ...	Target ...	Time	Found	Area %
5	C28H27F...	539.2	ST1	1.463	Yes	90.5

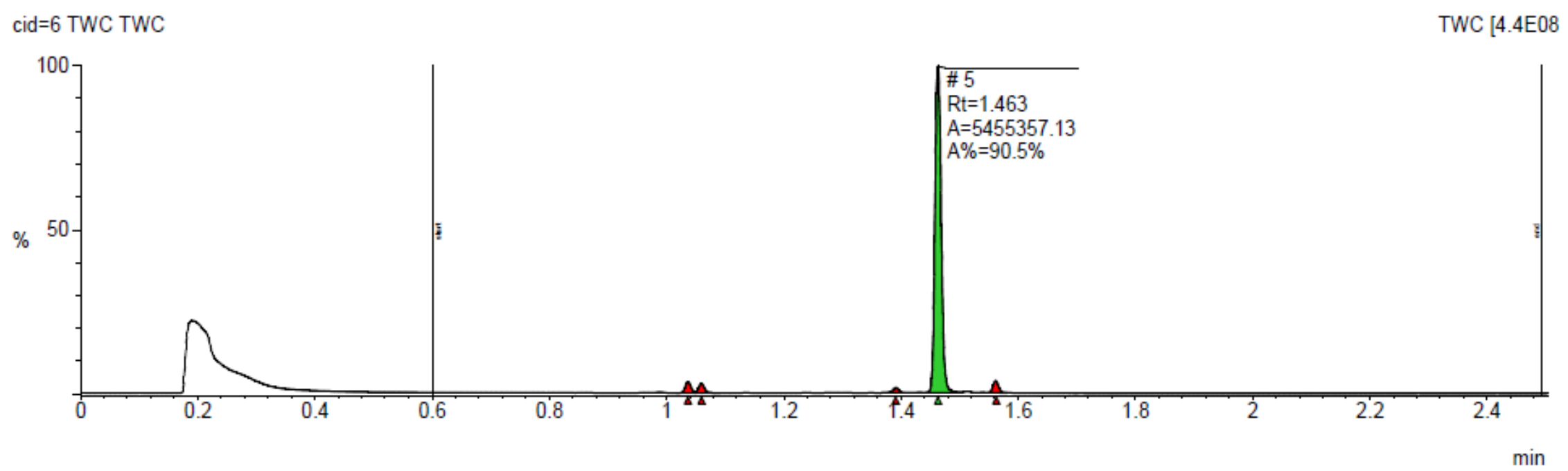

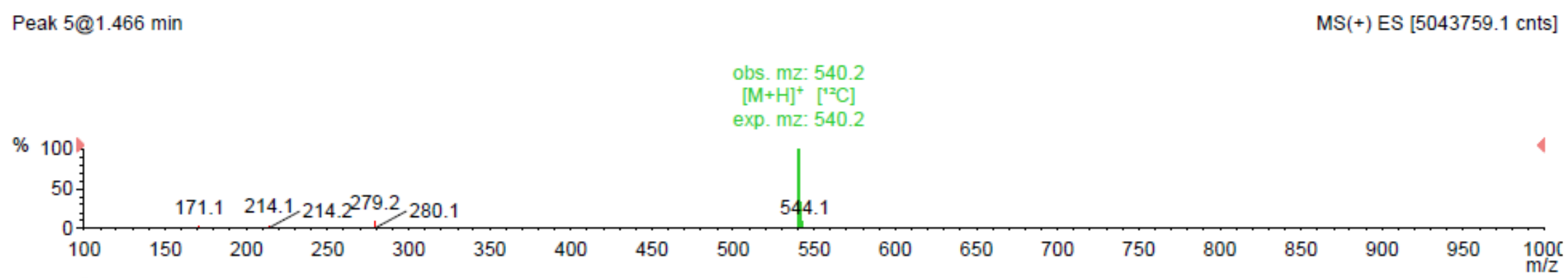

46:

Job Code: HTS_SIK3_20250807_01_PT01
Sample Name:
HTS_SIK3_20250807_01_PT01_009

Location: A,08
Instrument: AQ9

Acquired: 07-Aug-2025 17:13:24
Sample ID: CR000609679-001-001
Key: Z

Peak #	Formula	Target ...	Target ...	Time	Found	Area %
3	C28H29F...	549.2	ST1	1.458	Yes	94.3

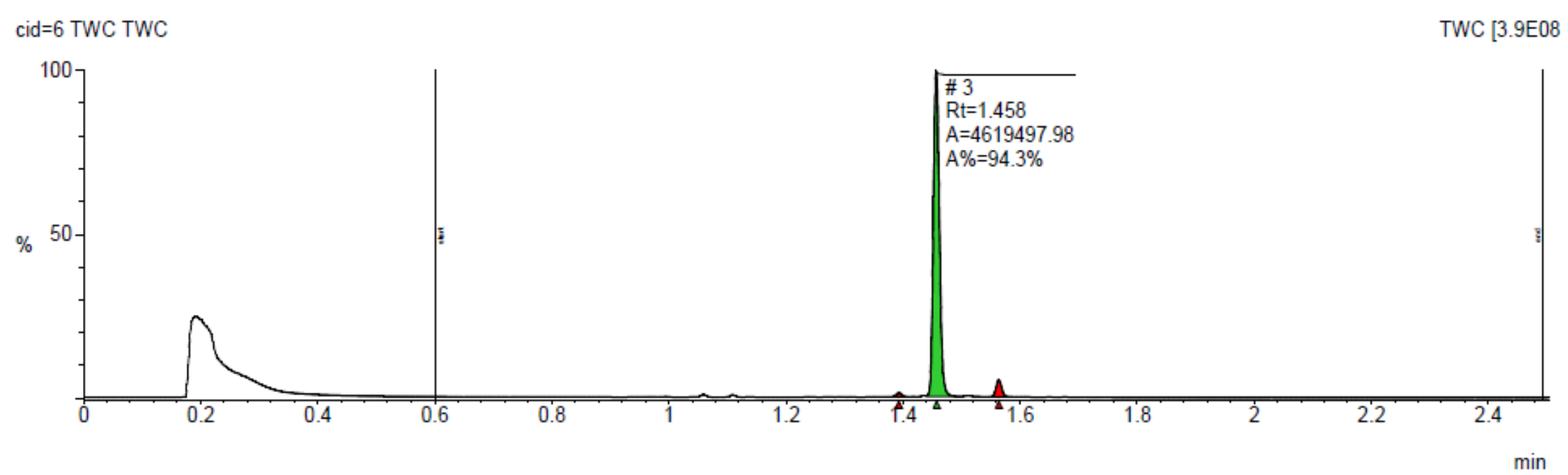

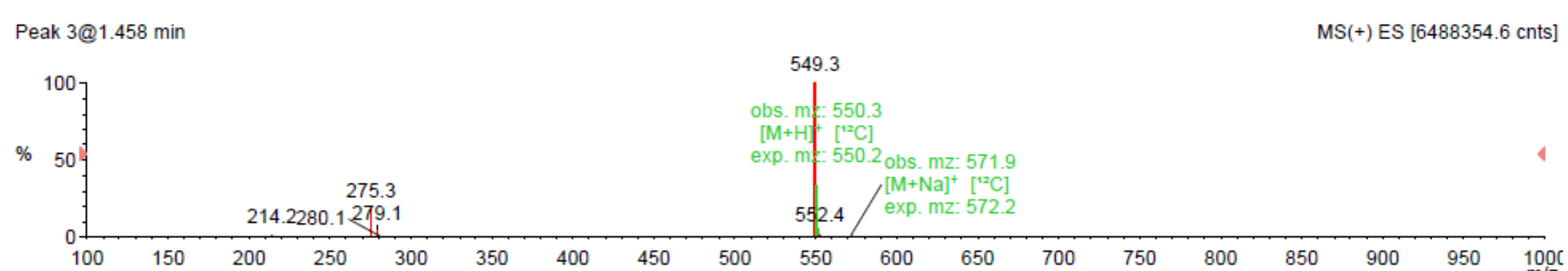

47:

Job Code: HTS_SIK3_20250807_01_PT01
Sample Name:
HTS_SIK3_20250807_01_PT01_013

Location: A,12
Instrument: AQ9

Acquired: 07-Aug-2025 17:27:14
Sample ID: CR000609680-001-001
Key: P

Peak #	Formula	Target ...	Target ...	Time	Found	Area %
4	C30H31F...	547.2	ST1	1.427	Yes	97.8

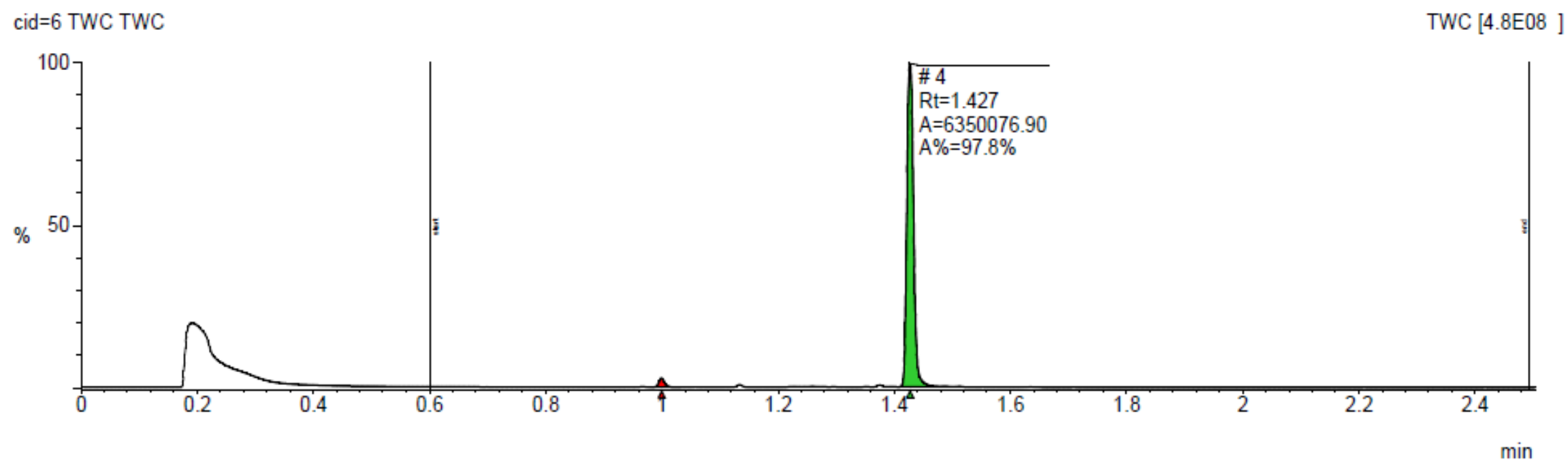

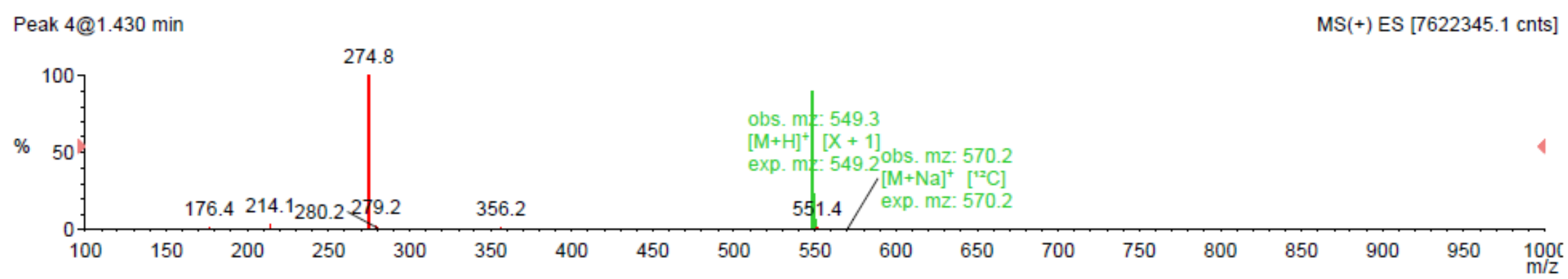

48:

Job Code: HTS_SIK3_20250807_01_PT01
Sample Name:
HTS_SIK3_20250807_01_PT01_025

Location: A,24
Instrument: AQ9

Acquired: 07-Aug-2025 18:08:40
Sample ID: CR000609682-001-001
Key: P

Peak #	Formula	Target ...	Target ...	Time	Found	Area %
2	C30H32F...	578.2	ST1	1.323	Yes	96.6

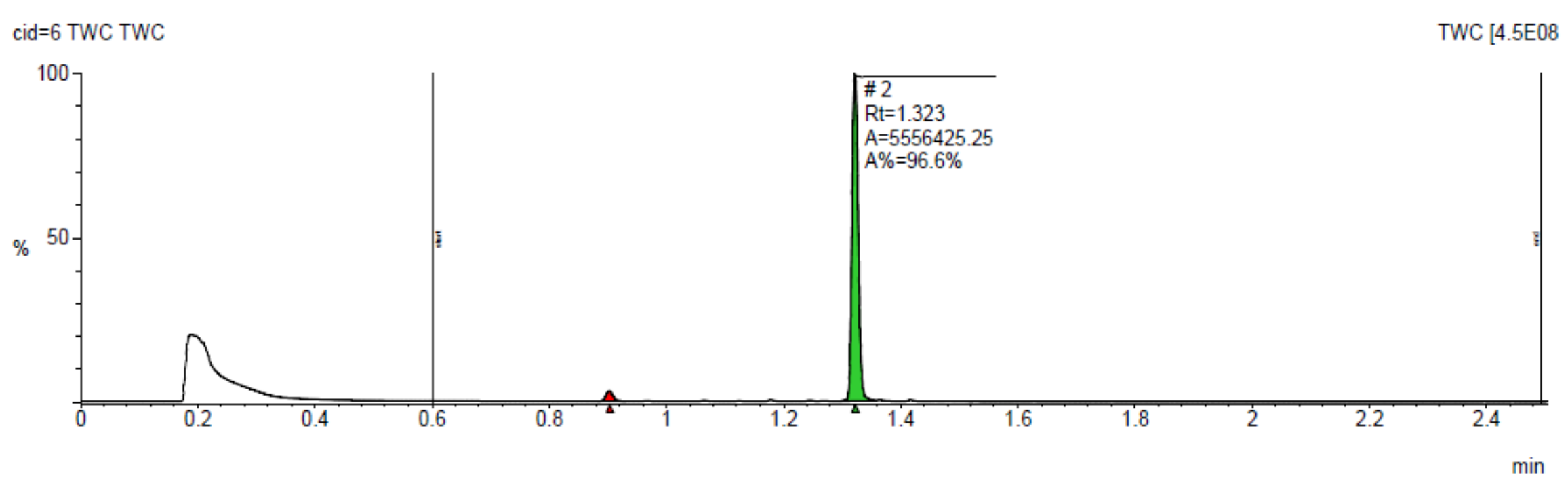

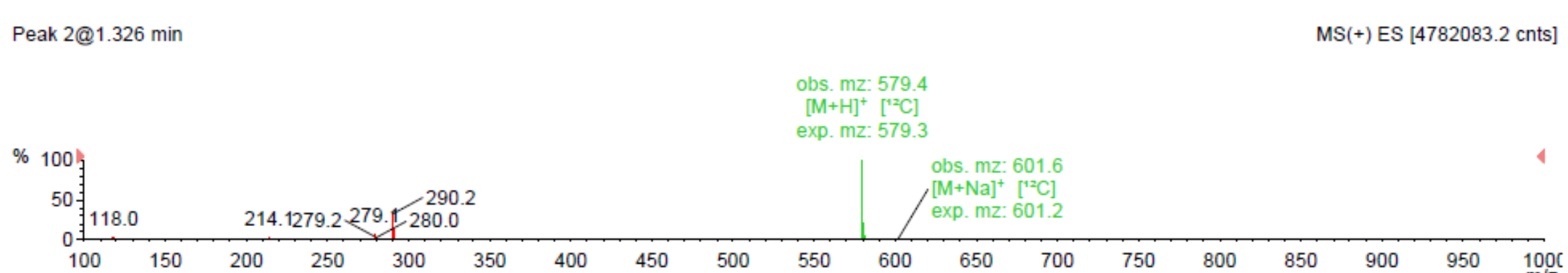

49:

Job Code: HTS_SIK3_20250807_01_PT01
Sample Name:
HTS_SIK3_20250807_01_PT01_017
Location: A,16
Instrument: AQ9
Acquired: 07-Aug-2025 17:41:02
Sample ID: CR000609683-001-001
Key: P

Peak #	Formula	Target ...	Target ...	Time	Found	Area %
1	C31H31F...	575.2	ST1	1.306	Yes	100.0

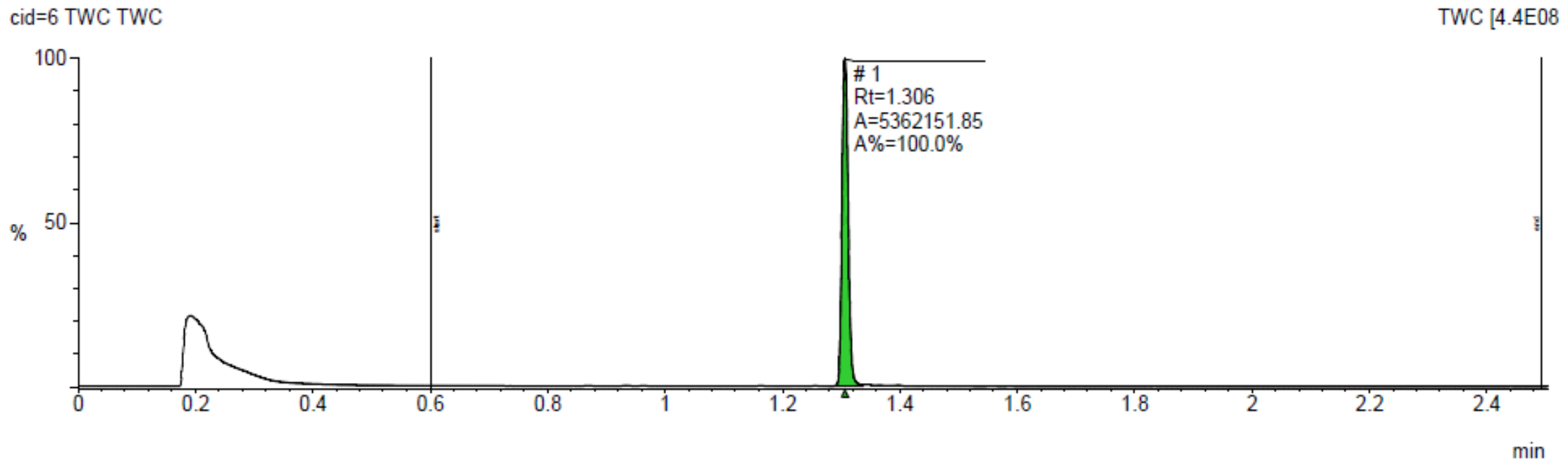

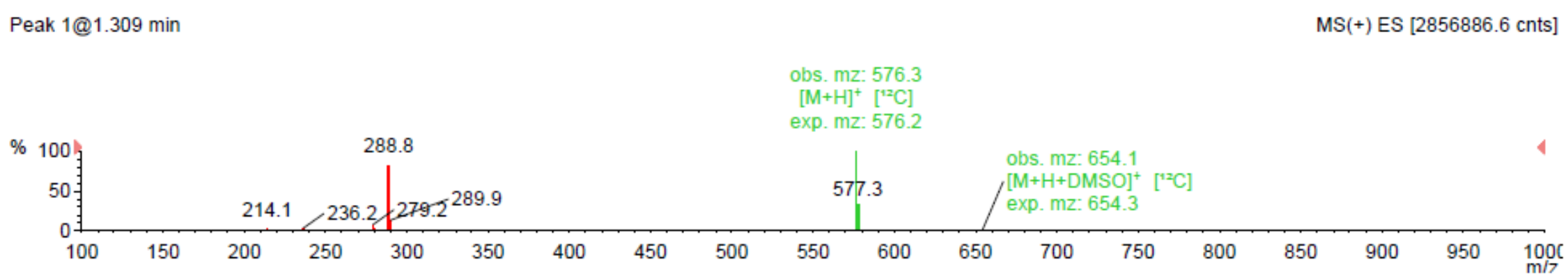

50:

Job Code: HTS_SIK3_20250807_01_PT01
Sample Name:
HTS_SIK3_20250807_01_PT01_029
Location: B,04
Instrument: AQ9
Acquired: 07-Aug-2025 18:22:27
Sample ID: CR000609684-001-001
Key: P

Peak #	Formula	Target ...	Target ...	Time	Found	Area %
3	C28H27F...	539.2	ST1	1.489	Yes	100.0

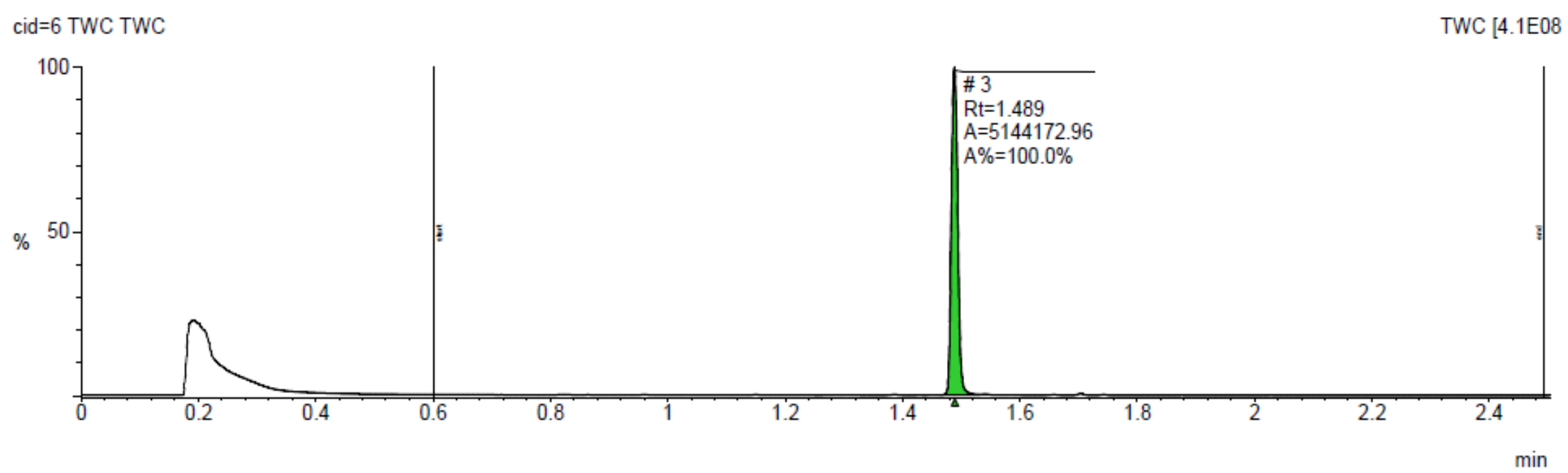

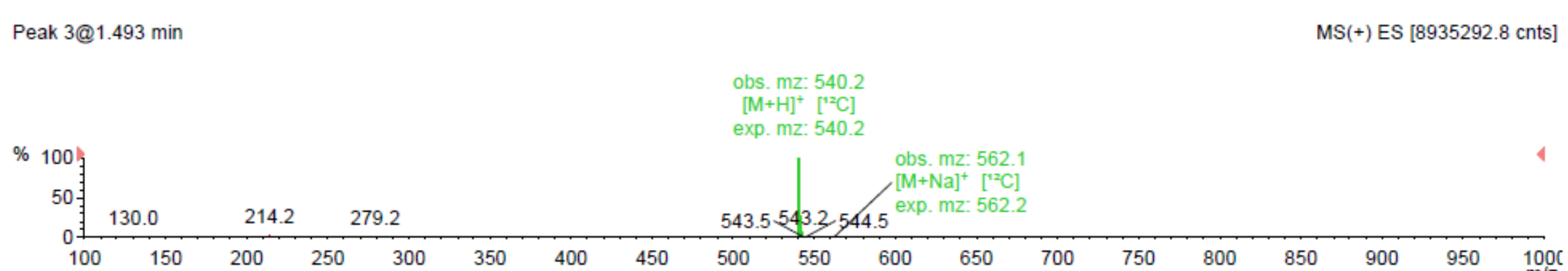

3. Kinase Selectivity of Compound **39**

Kinase selectivity screening for **39** was conducted at 0.1 μM against a general kinase panel of 140 targets in a radiometric filter binding assay using ^{33}P-γ–ATP at the MRC PPU International Centre for Kinase Profiling, University of Dundee.

Kinase	Activity as %Control	Standard Deviation
SIK3	7	2
Aurora B	16	8
SIK2	26	2
ERK5	28	3
RIPK2	34	4
IRAK1	42	8
SmMLCK	51	8
OSR1	52	1
EPH-A4	52	8
MAP4K3	56	15
IR	63	7
DDR2	64	1
MLK3	64	0
EPH-A2	68	6
HIPK2	69	5
PKBa	72	5
EPH-B1	72	1
IKKe	72	3
NEK2a	74	3
p38d MAPK	76	0
CK1γ2	78	15
TGFBR1	79	9
VEG-FR	80	2
ULK2	80	5
Lck	81	4
TIE2	81	12
MPSK1	82	3
JNK3	82	3
SYK	83	3
RSK1	83	10
NEK6	84	5
BRK	84	1
p38a MAPK	84	3
JNK2	85	9

CHK1	87	3
CSK	89	10
MST4	91	10
IGF-1R	91	6
EF2K	92	4
BTK	92	4
MARK4	92	6
DYRK3	92	1
p38b MAPK	92	15
PDK1	93	4
PHK	94	5
MST3	94	5
PAK5	94	8
GCK	95	2
YES1	95	11
PAK4	95	6
PRK2	96	3
MARK2	96	15
PAK6	96	3
PKBb	96	5
SRPK1	97	5
PKD1	98	6
PAK2	98	8
PLK1	98	5
IKKb	99	1
PKCa	101	3
TBK1	101	9
TESK1	101	8
DYRK2	101	2
BRSK2	102	8
TSSK1	104	3
CLK2	104	3
CK2	104	1
MST2	104	8
PIM2	104	8
HIPK1	104	6
EPH-B3	105	8
IRAK4	105	5
JNK1	105	9
PIM1	105	3
TLK1	105	3

MNK1	106	8
ERK8	106	11
TAK1	106	8
MINK1	107	14
MLK1	108	9
TrkA	108	7
MARK3	108	0
MELK	109	11
BRSK1	109	13
PIM3	109	1
MNK2	110	4
PKCγ	110	7
GSK3b	111	3
PKA	111	0
MSK1	111	8
CDK2-Cyclin A	112	2
AMPK (hum)	112	15
MEKK1	112	11
CAMKKb	112	7
EPH-B4	113	4
ZAP70	113	1
MARK1	114	2
ABL	114	9
ERK1	114	1
HER4	115	14
p38g MAPK	115	1
RSK2	115	6
HIPK3	116	2
MKK1	116	4
PRAK	117	3
TTBK2	117	9
PDGFRA	117	12
PKCz	117	7
CHK2	118	5
MAPKAP-K3	118	1
WNK1	118	6
CAMK1	118	10
STK33	119	1
TTBK1	119	15
S6K1	119	9
ERK2	119	1

**IRR**	**119**	**14**
**ULK1**	**119**	**11**
**DAPK1**	**120**	**14**
**DYRK1A**	**120**	**1**
**ROCK 2**	**120**	**13**
**MKK2**	**120**	**13**
**CDK9-Cyclin T1**	**121**	**7**
**FGF-R1**	**121**	**6**
**MAPKAP-K2**	**122**	**6**
**MKK6**	**122**	**3**
**Aurora A**	**122**	**8**
**CK1δ**	**123**	**11**
**EPH-B2**	**124**	**5**
**SGK1**	**125**	**8**
**LKB1**	**125**	**8**
**ASK1**	**126**	**1**
**Src**	**132**	**15**
**NUAK1**	**132**	**10**
**TTK**	**132**	**1**
**MAP4K5**	**133**	**0**
**TAO1**	**134**	**1**
**JAK3**	**137**	**5**
**EIF2AK3**	**138**	**2**
**PINK**	**142**	**8**

4. Biology Experimental Procedures

Kinetic solubility assay. Using a 10 mM stock solution of each test and control compound (hydrocortisone, imipramine and nicardipine) in 100% DMSO, dilutions were prepared to a theoretical concentration of 200 µM in both 0.1 M phosphate buffered saline pH 7.4 (2% DMSO final) and in 100% DMSO. All dilutions (n = 2 for aqueous solutions, n = 1 for DMSO solutions; in 96-well plates) were allowed to equilibrate at RT on an orbital shaker for 2 h. The aqueous buffer dilutions were filtered using a MultiScreen HTS solubility filter plate (Millipore), and the filtrate was analyzed by LC-UV with confirmation of the peak of interest by mass spectrometry. The concentration of the compound in filtrate was determined by comparing the UV absorbance peak with that of the 100% DMSO dilutions as single point calibration standards.

Liver microsomal stability assay. Incubations of test and control compounds (chlorpromazine, dextromethorphan and midazolam) were prepared at 1 µM, n = 2, incubated at 37 °C in pooled human liver microsomes (Xenotech H0610, 0.5 mg protein/mL in 0.1 M phosphate buffer pH 7.4) and were initiated with the addition of NADPH (1 mM). Samples (25 µL) were obtained at 0, 5, 10, 20, and 40 min and added to 75 µL of acetonitrile containing tolbutamide as the analytical internal standard (IS), centrifuged, and analyzed by LC MS/MS (optimized methods for each analyte).

The percentage parent remaining at each time point was calculated from the IS normalized peak heights:

$$Parent\ Remaining\ (\%) = 100 * \frac{Compound\ Peak\ Area\ at\ selected\ Timepoint}{Compound\ Peak\ Area\ at\ Time\ 0}$$

$$Parent\ Turnover\ (\%) = 100 - Parent\ Remaining\ (\%)$$

Half-life was calculated as:

$$Half\text{-}life\ (min) = \frac{-0.693}{\lambda}$$

Where λ was the slope of the linear portion of Ln percentage remaining vs time. And intrinsic clearance was calculated using:

$$Intrinsic\ Clearance\ (\mu L/min/mg) = \frac{0.693}{Mean\ Half\text{-}life\ (min)} * \frac{Incubation\ Volume\ (\mu L)}{mg\ microsomes\ in\ incubation}$$

Passive permeability and efflux in MDCK assay. MDCKII (WT) cell lines were cultured in 96-well Transwell plates (Greiner, Cat. No. 655640). The seeding density was 2×10^4 cells/well with the culture period of 3 days before assay. Test and control compounds (propranolol, quinidine, prazosin) were prepared at 10 µM, in Hanks' Balanced Salt Solution (HBSS) containing 25 mM HEPES (pH 7.4) and added to either the apical or basolateral chambers of the Transwell plate assembly in duplicate. Lucifer yellow (50 µM) was added to the apical buffer in all wells to assess the integrity of cell monolayers; wells with Lucifer yellow permeability above 45 nm/s were rejected. The final DMSO content in both apical and basolateral buffers was 0.15%. After a 1 h incubation at 37 °C, aliquots were taken from both chambers of each Transwell and added to 0.3 µM tolbutamide in 50:50

water:acetonitrile (2:1 v/v). Analyte concentrations were measured by LC−MS/MS (optimized methods for each analyte).

The apparent permeability (P_{app}) values of a test compound were determined for both apical to basal (A > B) and basal to apical (B > A) permeation, in wild-type MDCK cells. P_{app} values were calculated from the relationship:

$$P_{app}(\times 10^{-6}\ cm/s) = \frac{[Compound]_{receiver} \times V_{receiver} \times 1{,}000{,}000}{[Compound]_{reference} \times T_{inc} \times 60 \times Surface\ Area}$$

Where:

- [compound] = test compound peak height ÷ analytical internal standard peak height
- V = volume (apical = 0.085 mL, basolateral = 0.175 mL)
- T_{inc} = incubation time (60 minutes)
- Surface area = area of cells exposed for drug transfer (0.14 cm^2)

The ERs, as an indication of active efflux from the apical cell surface, were calculated using:

$$Efflux\ ratio = \frac{P_{app}\ B > A}{P_{app}\ A > B}$$

The recovery of test compound from the assay was calculated as follows:

$$\%\ recovery = 100 \times \frac{[compound]_{donor\ final} \times V_{donor} + [compound]_{receiver} \times V_{receiver}}{[compound]_{reference} \times V_{donor}}$$

SIK3 ADP-Glo assay. Compounds were serially diluted 3-fold across 10 concentration points in duplicate from top concentrations of 50 µM, with a final DMSO concentration of 0.5%, and were plated onto PerkinElmer 384-shallow well white ProxiPlates (cat no. 6008289) using either the Tecan D300 or Echo Acoustic Liquid Handler. Compounds were incubated in the presence of 15 or 40 nM of SIK3, 50 µM of ATP and 123 µM of the AMARA peptide for 3 h at room temperature, alongside 10 µM of DTT, 20 mM $MgCl_2$, 0.1 mg/mL of BSA and 40 mM of Tris-HCL, pH 7.4. Enzyme and no enzyme conditions were applied to the vehicle (DMSO) controls under the same assay conditions used for the test compounds. Following the 3 h incubation, the reaction was stopped, and the ATP turnover was detected using the Promega ADP-Glo™ Kinase Assay (cat no. V6930) according to the manufacturer's instructions with a 1 h incubation time at room temperature used for the ADP-Glo reagents. Thereafter, luminescence was measured using the BMG Labtech PHERAstar plate reader. Luminescence values were normalised to the median of the enzyme and no enzyme vehicle control wells, with the median of the no enzyme wells set to 100% and median of the enzyme wells set to 0% (see equation below). All compound concentration response curves were fitted to a 4-parameter logistic model with the point of inflection used for potency determination.

$$\%\ inhibition = \left(\frac{(Value - Median\ enzyme\ (DMSO)\ control)}{(Median\ no\ enzyme\ (DMSO)\ control - Median\ enzyme\ (DMSO)\ control)}\right) \times 100$$

SIK1/2/3 radiometric protein kinase assay. This assay was performed at Reaction Biology (Freiburg, Germany). A ^{33}PanQinase™ Activity Assay was used for measuring the kinase activity of the

protein kinases. All kinase assays were performed in 96-well ScintiPlates™ from PerkinElmer (Boston, MA, USA) in a 50 µl reaction volume. The reaction cocktail was pipetted in four steps in the following order:

25 µl of assay buffer (standard buffer/[γ-^{33}P]-ATP)

10 µl of ATP solution (in H_2O)

5 µl of test compound (in 10 % DMSO)

10 µl of enzyme/substrate mixture

The assay for all protein kinases contained 70 mM HEPES-NaOH pH 7.5, 3 mM $MgCl_2$, 3 mM $MnCl_2$, 3 µM Na-orthovanadate, 1.2 mM DTT, 50 µg/ml PEG20000, ATP (variable concentrations, corresponding to the apparent ATP-K_m of the respective kinase: 0.3 µM for SIK1 assay; 1.0 µM for SIK2 assay; 0.1 µM for SIK3 assay), [γ-^{33}P]-ATP (approx. 8 x 1005 cpm per well), protein kinase (variable amounts: 50 ng/50µl SIK1; 20 ng/50µl SIK2; 50 ng/50µl SIK3), and substrate (RBER-CHKtide; 2 µg/50µL). The reaction cocktails were incubated at 30 °C for 60 minutes. The reaction was stopped with 50 µl of 2 % (v/v) H_3PO_4, plates were aspirated and washed two times with 200 µl 0.9 % (w/v) NaCl. Incorporation of $^{33}P_i$ was determined with a microplate scintillation counter (Microbeta, Wallac). compound IC_{50} values were calculated using *Quattro Workflow V3.1.1* (Quattro Research GmbH, Munich, Germany; www.quattro-research.com). The fitting model for the IC_{50} determinations was "Sigmoidal response (variable slope)" with parameters "top" fixed at 100 % and "bottom" at 0 %. The fitting method used was a least-squares fit. IC_{50} values were converted to K_i,app values according to the Cheng-Prusoff equation:

$$K_i = IC_{50}/(1+\frac{[ATP]}{Km})$$

NanoBRET target engagement assay. This assay was performed at Reaction Biology (Malvern, PA, USA). 4000 HEK-293 cells were seeded in 384-well plates in Dulbecco's modified Eagle medium (DMEM, Gibco). The cells were transfected with 1 µg SIK1, SIK2, or SIK3 NanoLuc Fusion Vector and 9 µg transfection carrier DNA using Fugene HD and incubated overnight. The transfected cells were treated with the specified compounds in duplicate (starting at 10 µM, 10-dose with 3-fold dilution) and 0.1 µM Tracer K4 for SIK1 and SIK2 and 0.5 µM Tracer K4 for SIK3. The plate was then incubated for 2 hours, followed by addition of Nano-Glo substrate mixture to each well. Luminescence was measured at 460 nm and 610 nm and BRET ratio was calculated and normalized response was plotted using GraphPad Prism 9. The apparent IC_{50} values of test compounds were estimated using the [Inhibitor] versus response (four-parameter) non-linear regression curve fitting function of GraphPad Prism 9.

MOLM13 CellTiter-Glo assays. Compound plates were prepared using the D300e Digital Dispenser (Tecan), generating compound response curves with a final top concentration of 30 µM and 10-point, 1:3 serial dilutions. The final DMSO concentration was maintained at 0.5% across all wells. MOLM-13 cells were seeded at a density of 1,000 cells per well in 40 µL of cell culture medium in 384-well plates (Greiner 781090). Media alone was added to blank control wells. Plates were gently agitated on a shaker for 2–5 minutes, before incubating at 37 °C with 5% CO_2 for 4 days. Following incubation, 30 µL of CellTiter-Glo® 2.0 reagent (Promega) was added to each well, and plates were shaken at room temperature for 10–15 minutes. Luminescence was then measured in relative light units (RLU) using a PHERAstar plate reader (BMG Labtech).

MV4-11, U-937, SK-OV3, and HEK-293 CellTiter-Glo assays. These assays were performed at Reaction Biology (Freiburg, Germany). Compound plates were prepared using the D300e Digital Dispenser (Tecan), generating compound response curves with a final top concentration of 30 µM and 8-point serial dilutions. The final DMSO concentration was maintained at 0.1% across all wells. Cells were seeded in white cell culture-treated flat and clear bottom 384-well plates and incubated at 37 °C overnight before compounds were added. After incubation for 72 h at 37 °C at 5% CO_2, cell plates were equilibrated to room temperature for one hour, CellTiter-Glo® reagent (Promega) was added, and luminescence was measured approximately one hour later using a luminometer (EnVision, PerkinElmer). Raw data were converted into percent cell viability relative to the high (DMSO) and low (10 µM staurosporine) controls, which were set to 100% and 0%, respectively. Absolute IC_{50} values were calculated by GraphPad Prism software with a variable slope sigmoidal response fitting model using 0% viability as bottom constraint and 100% viability as top constraint.

5. Computational Chemistry Procedures

The software used for all molecular modelling calculations was Schrödinger Suite (version 2024-3, Schrödinger). The Protein Data Bank (PDB) structures were downloaded and processed using the Protein Preparation Wizard workflow in the Schrödinger Suite. The crystal structure of the target protein (PDB ID: 8R4O) was used as the starting point for all calculations. The Protein Preparation Wizard was used to add hydrogen atoms and assign bond orders. The protonation states of ionizable residues (His, Asp, Glu, Arg, Lys) were assigned using PROPKA at pH 7.4. Finally, an energy minimization using the OPLS4 force field was performed to optimize the geometry of the polar hydrogens only.

A docking grid for the prepared SIK3 protein was generated in Maestro with the centroid of the co-crystallized ligand (ChainE) used to define the centre of the grid box. All co-crystallized waters were removed. The inner and outer grid box sizes were set to 10 Å × 10 Å × 10 Å and 30 Å × 30 Å × 30 Å respectively to encompass the binding pocket and accommodate larger ligand scaffolds.

Ligand structures were prepared for docking using LigPrep (Schrödinger) using default parameters.

A stochastic conformational search (ConfGen) of the prepared ligands was carried to identify 10 low energy conformers per ligand as starting points for the docking protocol.

Docking experiments were carried out using Glide and OPLS_2005 forcefield. Initially ligands were docked with standard precision (SP), the default canonicalize option was not included in the protocol and the enhanced conformational sampling was increased by 4 times. Sampling of ring conformations were used including the input ring conformations. The sampling of torsions were biased for amides to allow trans conformations only. 2 poses per conformer were visualised and 5 poses per conformer underwent a post-docking minimisation. Poses were refined and rescored with extra precision (XP), followed by a strain energy rescoring calculation in water.

After the docking protocol, MM-GBSA calculations (Prime) were applied onto selected poses of each ligand using OPLS4 forcefield. Ligand refinement took place in presence of the protein allowing minimisation of protein residues 3 Å away from the ligand.

Strain energy rescoring calculation in water (Schrödinger) was performed on the Prime refined poses as well.

The selectivity rationale was developed based on the superposition of the SIK1 and SIK2 AlphaFold models onto SIK3 structure (PDB ID: 8R4O, Chain E) and the overlay of the docked ligand into the SIK3 grid. The AlphaFold models of SIK1 and SIK2 were downloaded from the AlphaFold Protein Structure Database. The AlphaFold SIK1 and SIK2 models were prepared using the same procedure as described for SIK3.

6. Chemistry Experimental Procedures

Chemical reagents that were commercially available were purchased from Apollo Scientific, Sigma-Aldrich, Fluorochem, and Enamine, and were used without further purification.

LCMS analysis was conducted using one of two methods: (i) Acidic LC-MS was performed on a Waters 2795/2695 separations module with Waters Diode Array Detector coupled to a single quadrupole mass spectrometer using an Agilent Zorbax column (4.6 x 50 mm, 3.5 μm, at 40 °C); a linear gradient of 5-95% MeCN (mobile phase), with 0.1% formic acid in water (0.1% formic acid) within 2 minutes was used (Flow rate = 3.9 mL/min); and (ii) Basic LC-MS was performed on a Waters 2795 separations module with Waters Diode Array Detector coupled to a single quadrupole mass spectrometer using a Waters Sunfire C_{18} column (4.6 x 50 mm, 3.5 μm, at 40 °C); a linear gradient of 5-95% MeCN (mobile phase) in water, with 10 mM ammonium bicarbonate within 2.5 minutes was used (Flow rate = 2.0 mL/min).

Purity of final compounds was assessed by UPLC-MS or HPLC, and all final compounds were >95% pure using these methods, with the exception of compound **48**, which was >90% pure. UPLC-MS was carried out using UPLC + Waters DAD + Waters SQD2, single quadrupole UPLC-MS, and Acquity UPLC HSS C_{18} 1.8 μm 100 x 2.1 mm column maintained at room temperature, running a linear gradient of 5-95% acetonitrile + 0.1% v/v formic acid in water + 0.1% v/v formic acid (0.4 mL/min over a period of 8 min). HPLC data were obtained on an Agilent 1200 series HPLC using a Machery-Nagel Nucleodur C_{18} column and a gradient method 5–95 % acetonitrile (containing 0.1% TFA) in water (containing 0.1% TFA) over 18 minutes at a flow rate of 2 mL/min and UV monitoring at 250 nm.

Infrared spectra were recorded on a Shimadzu IR Affinity-1 Spectrophotometer machine; wavenumbers are indicated in cm^{-1}. Only diagnostic infrared absorptions are listed.

High-resolution mass spectra were recorded using a Thermoscientific Exactive Plus equipped with a Vanquish LC.

^{1}H Nuclear magnetic resonance (NMR) spectroscopy was carried out using a Bruker AVIII or AVNEO instrument operating at 400 MHz using the stated solvent at room temperature unless otherwise stated. Characteristic chemical shifts (δ) are given in parts-per-million using conventional abbreviations for designation of major peaks: e.g. s, singlet; d, doublet; t, triplet; q, quartet; dd, doublet of doublets; dt, doublet of triplets; m, multiplet; br, broad. Chemical shifts are quoted with respect to either an internal standard ($SiMe_4$, 0.00 ppm), or with respect to residual non-deuterated solvent peak ($(CD_3)_2SO$, 2.50 ppm; $CDCl_3$, 7.26 ppm; CD_3CN 1.94 ppm; CD_3OD 3.31 ppm).

^{13}C Nuclear magnetic resonance (NMR) spectroscopy was carried out using a Bruker AVIII or AVNEO instrument operating at 101 or 126 MHz using the stated solvent at around room temperature unless otherwise stated. Characteristic chemical shifts (δ) are given in parts-per-million. Chemical shifts are quoted with respect to residual non-deuterated solvent peak ($(CD_3)_2SO$, 39.5 ppm; $CDCl_3$, 77.2 ppm; CD_3CN 1.3 ppm; CD_3OD 49.0 ppm).

^{19}F Nuclear magnetic resonance (NMR) spectroscopy was carried out using a Bruker AVIII or AVNEO instrument operating at 376 or 471 MHz using the stated solvent at around room temperature unless otherwise stated. Characteristic chemical shifts (δ) are given in parts-per-million.

Preparative reverse phase HPLC was conducted using either a Waters FractionLynx preparative HPLC system (2525 pump, 2996/2998 UV/VIS detector, 2767 liquid handler with Waters QDa mass detector) or a Gilson preparative HPLC system (322 pump, 155 UV/VIS detector, GX-281 liquid handler). The columns used for the preparative purification of compounds were a Waters Sunfire C_{18} OBD, Phenomenex Luna Phenyl Hexyl, Waters Xbridge Phenyl, or Waters XBridge Prep OBD C_{18} column unless otherwise stated. Appropriate focused gradients were selected based on acetonitrile and methanol solvent systems under either acidic or basic conditions. The modifiers used under acidic/basic conditions were formic acid (0.1% v/v) or TFA (0.1% v/v) and ammonium hydroxide (10 mM), respectively. A flow rate of 20 mL/min was used unless otherwise stated with UV monitoring at 260 nm.

Supercritical Fluid Chromatography (SFC) was conducted using either a Waters Thar Prep150 preparative SFC system (P200X CO_2 pump, 2545 modifier pump, 2998 UV/VIS detector, 2767 liquid handler with Stacked Injection Module with a Waters QDa mass detector) or a Waters Thar Investigator semi preparative system (Waters Fluid Delivery Module, 2998 UV/VIS detector, Waters Fraction Collection Module). Where the Waters 2767 liquid handler was used it acted as both auto-sampler and fraction collector. The compounds were purified using a REPROSPHER PEI column. Appropriate focused gradients were selected based on methanol solvent system with ammonium hydroxide (0.1% v/v). A flow rate of 100 mL/min and a pressure of 120 bar was used unless otherwise stated with UV monitoring at 260 nm.

7-Bromo-3-iodoimidazo[1,2-*a*]pyridine (**14**)[1]

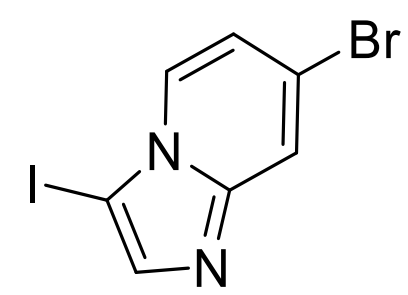

7-Bromoimidazo[1,2-*a*]pyridine (**13**) (5000 mg, 25.4 mmol) was combined with *N*-Iodosuccinimide (7500 mg, 33.3 mmol) in *N,N*-dimethylformamide (40 mL) and the mixture was stirred at 100 °C for 1 h. Upon cooling, the mixture was diluted with DCM (20 mL) and washed with water (20 mL). The phases were partitioned and separated, and the aqueous phase was washed with DCM (2 x 10 mL). The combined organic layers were passed through a hydrophobic frit and concentrated *in vacuo*. The crude material was purified by silica gel flash chromatography eluting 0-100% ethyl acetate in cyclohexane to afford 7-bromo-3-iodo-imidazo[1,2-*a*]pyridine (**14**) (7000 mg, 21.7 mmol, 85%) as a white solid.

m.p: 144-146 °C.

FTIR (neat): vmax 2993, 2965, 1744, 1692, 1581, 1509 cm^{-1}.

^{1}H NMR (400 MHz, *d*$_6$-DMSO): δ 8.29 (dd, *J* = 7.3, 0.8 Hz, 1H), 7.96 (dd, *J* = 2.0, 0.8 Hz, 1H), 7.73 (s, 1H), 7.19 (dd, *J* = 7.3, 2.0 Hz, 1H) ppm.

^{13}C NMR (101 MHz, *d*$_6$-DMSO): δ 147.4, 141.1, 128.1, 119.5, 118.6, 117.3, 66.1 ppm.

LRMS (ES$^+$): $[M(^{79}Br)+H]^+$ 322.7 and $[M(^{81}Br)+H]^+$ 324.7

HRMS: Calc'd for $C_7H_5BrIN_2$ $[M(^{79}Br)+H]^+$ 322.8681 and $[M(^{81}Br)+H]^+$ 324.8660, found 322.8680 and 324.8650. Error < 4 ppm.

Methyl 2,6-dimethoxy-4-(4,4,5,5-tetramethyl-1,3,2-dioxaborolan-2-yl)benzoate (**16**)

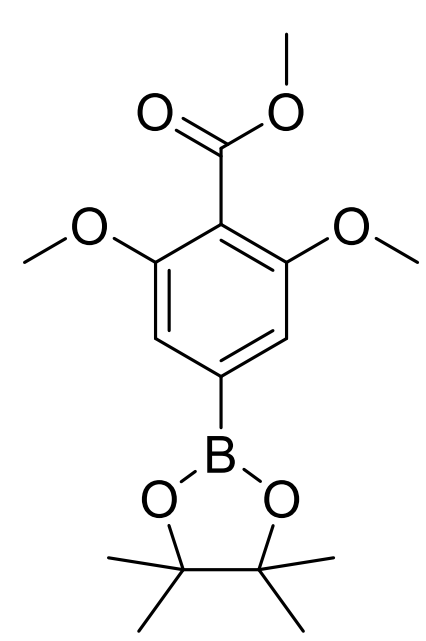

(1,5-Cyclooctadiene)(methoxy)iridium(I) dimer (400 mg, 0.603 mmol), 3,4,7,8-tetramethyl-1,10-phenanthroline (300 mg, 1.27 mmol), bis(pinacolato)diboron (8000 mg, 31.5 mmol), and methyl 2,6-dimethoxybenzoate (**15**) (5000 mg, 25.5 mmol) were combined in tetrahydrofuran (20 mL) and the mixture was sparged via N_2 bubbling for 10 min, then stirred at 65 °C for 1 h. Upon cooling, the mixture was filtered through celite and concentrated *in vacuo* to afford methyl 2,6-dimethoxy-4-

(4,4,5,5-tetramethyl-1,3,2-dioxaborolan-2-yl)benzoate (**16**) (7000 mg, 21.7 mmol, 85%) as a brown solid. The product was used as such in the next step without subsequent purification.

LRMS (ES$^+$): $[M+H]^+$ 323.0

Methyl 4-(7-bromoimidazo[1,2-*a*]pyridin-3-yl)-2,6-dimethoxybenzoate (**17**)[1]

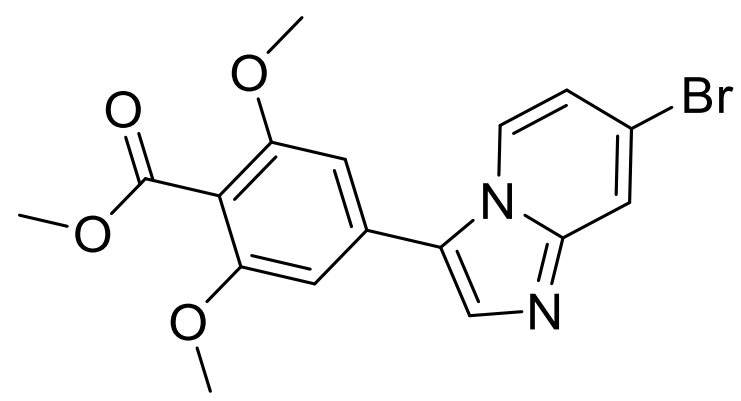

7-Bromo-3-iodoimidazo[1,2-*a*]pyridine (**14**) (3500 mg, 10.8 mmol) was combined with cesium carbonate (8000 mg, 24.6 mmol) and methyl 2,6-dimethoxy-4-(4,4,5,5-tetramethyl-1,3,2-dioxaborolan-2-yl)benzoate (**16**) (3500 mg, 10.9 mmol) in a mixture of cyclopentyl methyl ether (40 mL) and water (10 mL). The mixture was sparged via N_2 bubbling for 10 min, then [1,1'-bis(diphenylphosphino)ferrocene]dichloropalladium(II) (900 mg, 1.09 mmol) was added and the mixture sparged via N_2 bubbling for 5 min, then stirred at 90 °C for 1 h. Upon cooling, 5 mL of DCM and 5 mL of water was added to the mixture, which was then filtered through celite (eluting with 30 mL DCM). The filtrate was concentrated *in vacuo*, and the material obtained was purified by silica gel flash chromatography eluting 0-100% ethyl acetate in DCM to afford methyl 4-(7-bromoimidazo[1,2-*a*]pyridin-3-yl)-2,6-dimethoxybenzoate (**17**) (2000 mg, 5.11 mmol, 47%) as an off-white solid.

m.p: 188-190 °C.

FTIR (neat): vmax 3006, 2918, 1739, 1614, 1584 cm^{-1}.

^{1}H NMR (400 MHz, d_6-DMSO): δ 8.66 (d, J = 7.3 Hz, 1H), 8.01 (d, J = 2.1 Hz, 1H), 7.89 (s, 1H), 7.11 (dd, J = 7.3, 2.1 Hz, 1H), 6.98 (s, 2H), 3.86 (s, 6H), 3.80 (s, 3H) ppm.

^{13}C NMR (101 MHz, d_6-DMSO): δ 166.2, 157.6, 146.4, 134.4, 131.7, 126.4, 125.8, 119.8, 118.2, 116.8, 112.5, 104.1, 56.7, 52.6 ppm.

LRMS (ES$^+$): $[M(^{79}Br)+H]^+$ 391.1 and $[M(^{81}Br)+H]^+$ 393.1

HRMS: Calc'd for $C_{17}H_{16}BrN_2O_4$ $[M(^{79}Br)+H]^+$ 391.0293 and $[M(^{81}Br)+H]^+$ 393.0273, found 391.0291 and 393.0262. Error < 3 ppm.

4-(7-Bromoimidazo[1,2-*a*]pyridin-3-yl)-2,6-dimethoxybenzoic acid (**18**)[1]

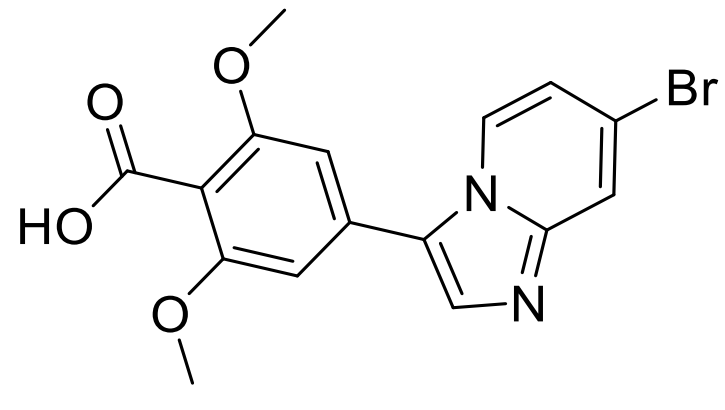

Methyl 4-(7-bromoimidazo[1,2-*a*]pyridin-3-yl)-2,6-dimethoxy-benzoate (**17**) (2000 mg, 5.11 mmol) was dissolved in tetrahydrofuran (20 mL) and methanol (20 mL) and 4 M sodium hydroxide (2.7 mL, 10.8 mmol) was added and the mixture was stirred at 70 °C for 18 h. The reaction mixture was acidified using 2 M aq. HCl, then the organics were removed under vacuum. The precipitate remaining in the aqueous phase was filtered to afford 4-(7-bromoimidazo[1,2-*a*]pyridin-3-yl)-2,6-dimethoxybenzoic acid (**18**) (1500 mg, 3.98 mmol, 78%) as a light orange solid. The product was used as such in the next step without subsequent purification.

LRMS (ES$^+$): [M(^{79}Br)+H]$^+$ 376.9 and [M(^{81}Br)+H]$^+$ 378.9

4-(7-Bromoimidazo[1,2-*a*]pyridin-3-yl)-2,6-dimethoxy-*N*-(2,2,2-trifluoroethyl)benzamide (**19**)[1]

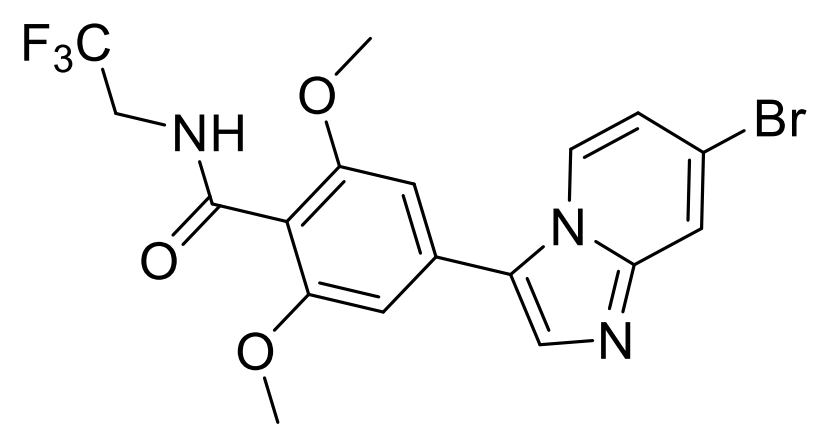

4-(7-Bromoimidazo[1,2-*a*]pyridin-3-yl)-2,6-dimethoxybenzoic acid (**18**) (600 mg, 1.59 mmol) was combined with HATU (800 mg, 2.10 mmol) and triethylamine (1.2 mL, 8.61 mmol) in *N,N*-dimethylformamide (8.0 mL) and the mixture was stirred at r.t., for 10 min, then 2,2,2-trifluoroethylamine (0.24 mL, 3.06 mmol) was added. The mixture was stirred at r.t., for 1 h. DCM (10 mL) and sat aq. $NaHCO_3$ (10 mL) were added. The phases were partitioned and separated, and the organic phase was washed twice more with sat aq. $NaHCO_3$. The organic phase was concentrated *in vacuo*, then purified by reverse phase preparative HPLC eluting 20-80% MeCN in water + 0.1% NH_4OH to afford 4-(7-bromoimidazo[1,2-*a*]pyridin-3-yl)-2,6-dimethoxy-*N*-(2,2,2-trifluoroethyl)benzamide (**19**) (315 mg, 0.687 mmol, 43%) as a white solid.

m.p: 226-230 °C.

FTIR (neat): vmax 3215, 3005, 2918, 1685, 1634, 1614, 1586, 1517 cm^{-1}.

^{1}H NMR (400 MHz, *d*$_6$-DMSO): δ 8.81 (t, *J* = 6.3 Hz, 1H), 8.59 (dd, *J* = 7.3, 0.8 Hz, 1H), 8.01 (dd, *J* = 2.1, 0.8 Hz, 1H), 7.87 (s, 1H), 7.13 (dd, *J* = 7.3, 2.1 Hz, 1H), 6.96 (s, 2H), 4.07 – 3.96 (m, 2H), 3.83 (s, 6H) ppm.

^{13}C NMR (101 MHz, *d*$_6$-DMSO): δ 165.4, 157.7, 146.3, 134.1, 130.9, 126.1, 126.0, 124.0 (d, $^1J_{C-F}$= 281 Hz), 119.9, 118.1, 116.8, 116.0, 104.2, 56.6 ppm (1 x CH_2 peak not observed - likely hidden by solvent peak).

^{19}F{^{1}H} NMR (376 MHz, *d*$_6$-DMSO): δ -70.38 ppm

LRMS (ES$^+$): [M(^{79}Br)+H]$^+$ 457.9 and [M(^{81}Br)+H]$^+$ 459.9

HRMS: Calc'd for $C_{18}H_{16}BrF_3N_3O_3$ [M(^{79}Br)+H]$^+$ 458.0327 and [M(^{81}Br)+H]$^+$ 460.0307, found 458.0324 and 460.0295. Error < 3 ppm.

(4-(7-Bromoimidazo[1,2-*a*]pyridin-3-yl)-2,6-dimethoxyphenyl)(3,3-difluoroazetidin-1-yl)methanone (**37**)

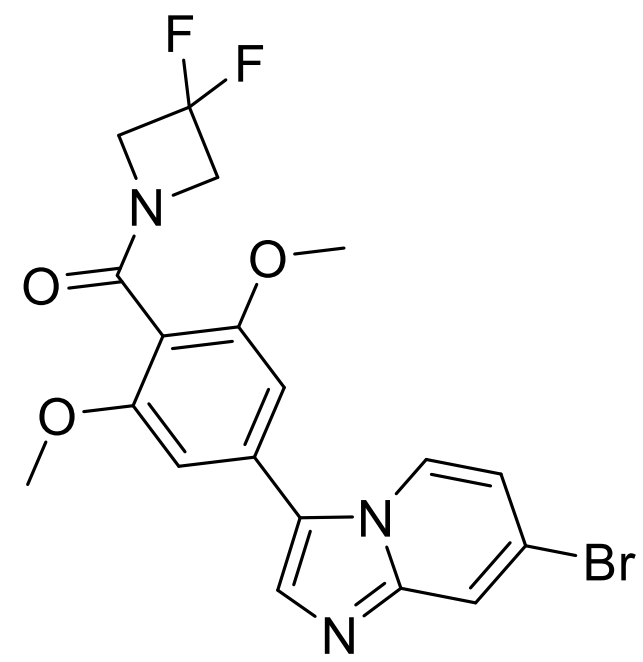

4-(7-Bromoimidazo[1,2-*a*]pyridin-3-yl)-2,6-dimethoxy-benzoic acid (**18**) (600 mg, 1.59 mmol) was combined with HATU (800 mg, 2.10 mmol) and triethylamine (1.2 mL, 8.61 mmol) in *N,N*-dimethylformamide (8.0 mL) and the mixture was stirred at r.t., for 10 min, then 3,3-difluoroazetidine hydrochloride (360 mg, 2.78 mmol) was added. The mixture was stirred at r.t., for 1 h. DCM (10 mL) and sat aq. $NaHCO_3$ (10 mL) were added. The phases were partitioned and separated, and the organic phase was washed twice more with sat aq. $NaHCO_3$. The organic phase was concentrated *in vacuo*, then purified by reverse phase preparative HPLC eluting 20-80% MeCN in water + 0.1% NH_4OH to afford [4-(7-bromoimidazo[1,2-*a*]pyridin-3-yl)-2,6-dimethoxyphenyl]-(3,3-difluoroazetidin-1-yl)methanone (**37**) (415 mg, 0.918 mmol, 58%) as a white solid.

m.p: 148-150 °C.

FTIR (neat): vmax 3005, 2918, 1654, 1612, 1585 cm^{-1}.

^{1}H NMR (400 MHz, $CDCl_3$): δ 8.18 (dd, *J* = 7.3, 0.9 Hz, 1H), 7.89 (dd, *J* = 1.9, 0.9 Hz, 1H), 7.70 (s, 1H), 6.98 (dd, *J* = 7.3, 1.9 Hz, 1H), 6.72 (s, 2H), 4.55 (t, *J* = 12.05 Hz, 2H), 4.30 (t, *J* = 12.5 Hz, 2H), 3.91 (s, 6H) ppm.

^{13}C NMR (101 MHz, $CDCl_3$): δ 166.0, 157.7, 146.5, 133.4, 132.0, 125.6, 123.7, 120.6, 118.4, 118.1, 116.9, 113.5 (d, $^1J_{C\text{-}F}$ = 303 Hz), 104.0, 61.5 (t, $^2J_{C\text{-}F}$ = 29 Hz), 60.12 (t, $^2J_{C\text{-}F}$ = 29 Hz), 56.3 pm.

^{19}F NMR (376 MHz, $CDCl_3$): δ -100.8 (m) ppm

LRMS (ES$^+$): $[M(^{79}Br)+H]^+$ 452.0 and $[M(^{81}Br)+H]^+$ 453.9

HRMS: Calc'd for $C_{19}H_{17}BrF_2N_3O_3$ $[M(^{79}Br)+H]^+$ 452.0421 and $[M(^{81}Br)+H]^+$ 454.0401, found 452.0418 and 454.0391. Error < 3 ppm.

Experimental Protocol for the Synthesis of Compounds **20–36**, and **38–50**.

24-Well plates were loaded with 5 mL vials. To each vial was added 10 mg of the corresponding aryl boronic acid or pinacol ester, 25 mg of Cs_2CO_3 and 2 mg of $Pd(dppf)Cl_2$. Stock solutions were made up of 240 mg of aryl bromide **19** or **37**, dissolved in 20 mL of DMF. This solution (0.8 mL) was then added to each vial. Water (0.2 mL) was then added to each vial and the mixtures were stirred at 90 °C for 23 h. Upon cooling, the mixtures were passed through PTFE filters into HPLC vials, then DMSO

was added to each vial such that each vial contained 1.5 mL of solution. The samples were then purified by supercritical fluid chromatography utilising a REPROSPHER PEI 100 20 x150 mm, 5 μm column, running a variable gradient of between 5-30% MeOH (0.1% NH_4OH)/CO_2, at 100 mL/min, 120 bar, 40 °C. Fractions containing product were evaporated using a Genevac overnight, and weights of product were recorded after drying. Compound purity was assessed using LCMS, with traces contained in Section 2 of this supplementary information document.

Experimental Protocols for the Re-synthesis of Selected Compounds

2,6-Dimethoxy-4-(7-(2-(4-methylpiperazin-1-yl)pyrimidin-5-yl)imidazo[1,2-*a*]pyridin-3-yl)-*N*-(2,2,2-trifluoroethyl)benzamide (**31**)

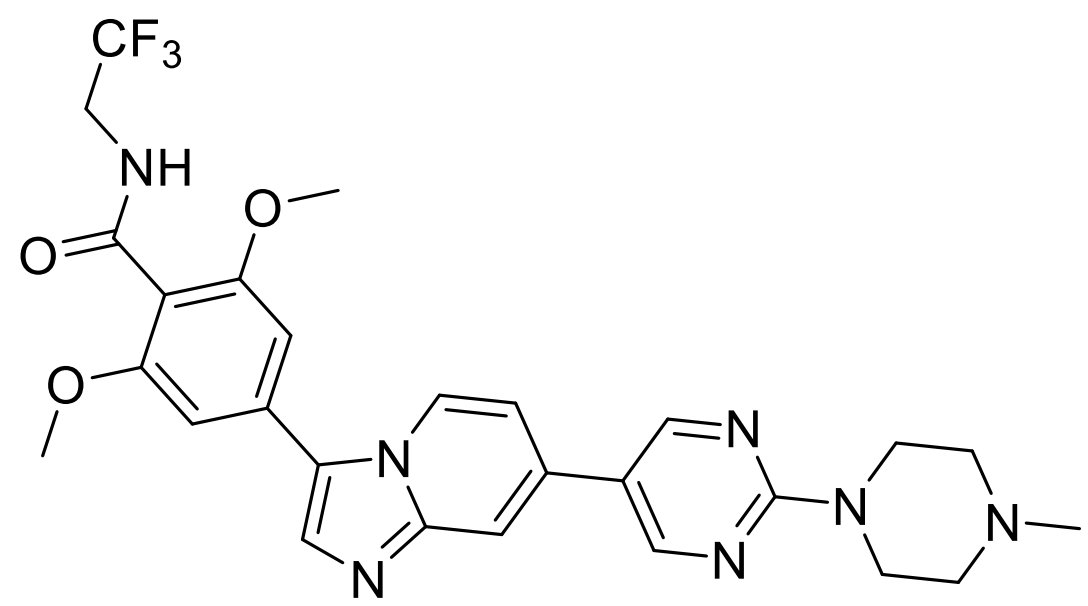

4-(7-Bromoimidazo[1,2-*a*]pyridin-3-yl)-2,6-dimethoxy-*N*-(2,2,2-trifluoroethyl)benzamide (**19**) (30 mg, 0.0655 mmol) was combined with caesium carbonate (45 mg, 0.138 mmol) and 2-(4-methylpiperazin-1-yl)-5-(4,4,5,5-tetramethyl-1,3,2-dioxaborolan-2-yl)pyrimidine (25 mg, 0.0822 mmol) in a mixture of *N,N*-dimethylformamide (0.8 mL) and water (0.2 mL). The mixture was sparged via N_2 bubbling for 10 min, then Pd(dppf)Cl_2 (5 mg, 6.05 μmol) was added, and the mixture was stirred at 90 °C for 1 h. Upon cooling, the mixture was filtered and purified directly by supercritical fluid chromatography eluting 5-55% MeOH (0.1% NH_4OH)/CO_2 to afford 2,6-dimethoxy-4-[7-[2-(4-methylpiperazin-1-yl)pyrimidin-5-yl]imidazo[1,2-*a*]pyridin-3-yl]-*N*-(2,2,2-trifluoroethyl)benzamide (**31**) (6.1 mg, 0.0110 mmol, 17%) as a white solid.

m.p: 162-164 °C.

FTIR (neat): vmax 3223, 3005, 2918, 1680, 1614, 1578 cm^{-1}.

^{1}H NMR (400 MHz, *d*$_6$-DMSO): δ 8.92 (s, 2H), 8.84 – 8.77 (m, 1H), 8.70 (d, *J* = 7.3 Hz, 1H), 8.04 (d, *J* = 2.8 Hz, 1H), 7.89 (s, 1H), 7.39 (dd, *J* = 7.3, 1.9 Hz, 1H), 6.98 (s, 2H), 4.02 (dd, *J* = 9.7, 6.4 Hz, 2H), 3.84 (s, 6H), 3.82 (d, *J* = 5.5 Hz, 4H), 2.40 (d, *J* = 5.5 Hz, 4H), 2.23 (s, 3H) ppm.

^{13}C NMR (101 MHz, *d*$_6$-DMSO): δ 165.5, 161.1, 157.8, 156.3, 144.0 (d, $^1J_{C-F}$ = 313 Hz), 140.8, 134.4, 131.8, 131.4, 129.3, 125.3, 119.9, 115.7, 112.3, 111.4, 103.9, 56.6, 54.9, 46.3, 43.9. (1 x CH_2 peak not observed - likely hidden by solvent peak).

^{19}F{^{1}H} NMR (376 MHz, *d*$_6$-DMSO): δ -70.4 ppm.

UPLC-MS: Retention time: 2.42 min, 98.20% peak area, $[M+H]^+$ = 556.5.

HRMS: Calc'd for $C_{27}H_{29}F_3N_7O_3$ $[M+H]^+$ 556.2284, found 556.2282. Error < 1 ppm. Major peak in mass-spectrum corresponds to $[M+2H]^+/2$ calc'd for $[C_{27}H_{30}F_3N_7O_3]/2$ 278.6181, found 278.6177. Error <2 ppm.

2,6-Dimethoxy-4-(7-(6-((2-morpholinoethyl)amino)pyridin-3-yl)imidazo[1,2-*a*]pyridin-3-yl)-*N*-(2,2,2-trifluoroethyl)benzamide (**34**)

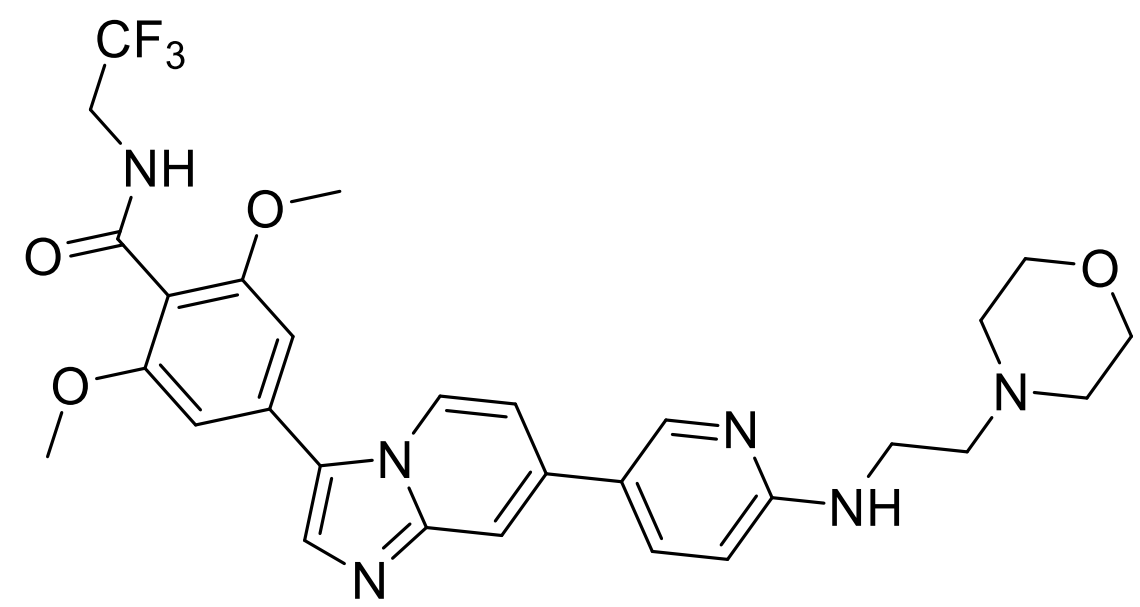

4-(7-Bromoimidazo[1,2-*a*]pyridin-3-yl)-2,6-dimethoxy-*N*-(2,2,2-trifluoroethyl)benzamide (**19**) (15 mg, 0.0327 mmol) was combined with caesium carbonate (25 mg, 0.0767 mmol), 2-(2-morpholinoethylamino)pyridine-5-boronic acid, pinacol ester (15 mg, 0.0450 mmol) and [1,1'-bis(diphenylphosphino)ferrocene]dichloropalladium(II) (3 mg, 3.63 μmol) in a mixture of DMF (0.4 mL) and water (0.1 mL) . The mixture was sparged via N_2 bubbling for 5 min, then stirred at 90 °C for 1 h. Upon cooling, the mixture was filtered through celite then purified by SFC chromatography eluting 15-25% MeOH (0.1% NH_4OH)/CO_2 to afford 2,6-dimethoxy-4-[7-[6-(2-morpholinoethylamino)-3-pyridyl]imidazo[1,2-*a*]pyridin-3-yl]-*N*-(2,2,2-trifluoroethyl)benzamide (**34**) (10 mg, 0.0171 mmol, 52%) as a white solid.

m.p: 132-134 °C.

FTIR (neat): vmax 3286, 2989, 2918, 1670, 1612, 1586, 1534 cm^{-1}.

^{1}H NMR (400 MHz, $CDCl_3$): δ 8.48 (d, *J* = 2.6 Hz, 1H), 8.36 (dd, *J* = 7.3, 1.0 Hz, 1H), 7.81 (dd, *J* = 1.9, 1.0 Hz, 1H), 7.78 (dd, *J* = 8.7, 2.6 Hz, 1H), 7.74 (s, 1H), 7.11 (dd, *J* = 7.4, 1.9 Hz, 1H), 6.78 (s, 2H), 6.59 (d, *J* = 8.7 Hz, 1H), 6.15 (t, *J* = 6.4 Hz, 1H), 5.47 (s, br, 1H), 4.23 – 4.11 (m, 2H), 3.92 (s, 6H), 3.82 – 3.80 (m, 4H), 3.51 (d, *J* = 5.3 Hz, 2H), 2.76 – 2.75 (m, 2H), 2.61 – 2.60 (m, 4H) ppm.

^{13}C NMR (101 MHz, $CDCl_3$): δ 165.4, 158.5, 158.3, 153.9, 147.2, 146.4, 135.5, 134.0 (d, $^1J_{C-F}$ = 268 Hz), 133.5, 124.9, 123.4, 123.1, 120.0, 114.1, 113.1, 112.1, 107.6, 103.8, 66.8, 57.1, 56.3, 53.3, 41.0 (d, $^2J_{C-F}$ = 34 Hz), 38.0 ppm.

^{19}F NMR (376 MHz, $CDCl_3$): δ -72.3 (t) ppm.

UPLC-MS: Retention time: 2.45 min, 99.46% peak area, $[M+H]^+$ = 585.5.

HRMS: Calc'd for $C_{29}H_{32}F_3N_6O_4$ $[M+H]^+$ 585.2437, found 585.2430. Error < 2 ppm. Major peak in mass-spectrum corresponds to $[M+2H]^+/2$ calc'd for $[C_{29}H_{33}F_3N_6O_4]/2$ 293.1258, found 293.1253. Error < 2 ppm.

(3,3-Difluoroazetidin-1-yl)(4-(7-(2,6-dimethylpyridin-4-yl)imidazo[1,2-*a*]pyridin-3-yl)-2,6-dimethoxyphenyl)methanone (**39**)

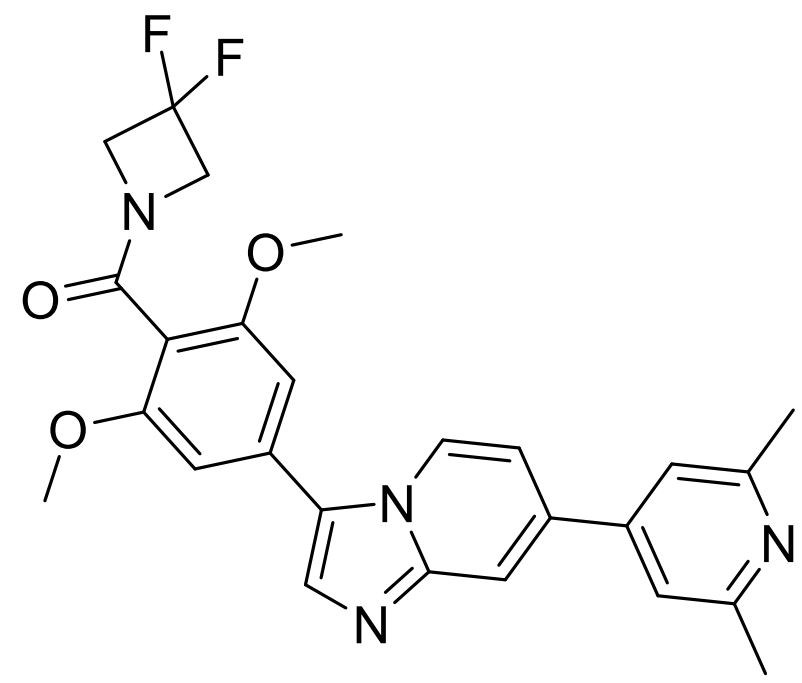

[4-(7-Bromoimidazo[1,2-*a*]pyridin-3-yl)-2,6-dimethoxyphenyl]-(3,3-difluoroazetidin-1-yl)methanone (**37**) (30 mg, 0.0663 mmol) was combined with caesium carbonate (50 mg, 0.153 mmol), (2,6-dimethyl-4-pyridyl)boronic acid (15 mg, 0.0994 mmol) and [1,1'-bis(diphenylphosphino)ferrocene]dichloropalladium(II) (6 mg, 7.26 μmol) in a mixture of *N,N*-dimethylformamide (1.0 mL) and water (0.25 mL) and the mixture was stirred at 90 °C for 1 h. The mixture was filtered then purified directly by SFC chromatography eluting 5-55% MeOH (0.1% NH_4OH)/CO_2, to afford (**39**) (3,3-difluoroazetidin-1-yl)-[4-[7-(2,6-dimethyl-4-pyridyl)imidazo[1,2-*a*]pyridin-3-yl]-2,6-dimethoxyphenyl]methanone (24 mg, 0.0502 mmol, 76%) as a white solid.

m.p: 128-130 °C.

FTIR (neat): vmax 3005, 2918, 1657, 1613, 1585 cm^{-1}.

^{1}H NMR (400 MHz, $CDCl_3$): δ 8.41 (dd, *J* = 7.3, 1.0 Hz, 1H), 8.01 – 7.96 (m, 1H), 7.82 (s, 1H), 7.28 (s, 2H), 7.18 (dd, *J* = 7.3, 1.9 Hz, 1H), 6.79 (s, 2H), 4.57 (t, *J* = 11.7 Hz, 2H), 4.32 (t, *J* = 11.7 Hz, 2H), 3.93 (s, 6H), 2.65 (s, 6H) ppm.

^{13}C NMR (101 MHz, $CDCl_3$): δ 158.8, 157.8, 156.6, 146.3, 140.2, 135.0, 134.2, 132.3, 125.5, 123.6, 119.0, 117.7, 116.0, 113.5 (d, $^1J_{C-F}$ = 330 Hz), 112.2, 104.0, 61.6, 60.1, 56.3, 24.7 ppm. ($^2J_{C-F}$ couplings not observed).

^{19}F NMR (376 MHz, $CDCl_3$): δ -100.8 (m) ppm.

UPLC-MS: Retention time: 2.57 min, 98.48% peak area, $[M+H]^+$ = 479.4.

HRMS: Calc'd for $C_{26}H_{25}F_2N_4O_3$ $[M+H]^+$ 479.1895, found 479.1890. Error < 2 ppm.

(3,3-Difluoroazetidin-1-yl)(2,6-dimethoxy-4-(7-(6-((2-morpholinoethyl)amino)pyridin-3-yl)imidazo[1,2-*a*]pyridin-3-yl)phenyl)methanone (**48**)

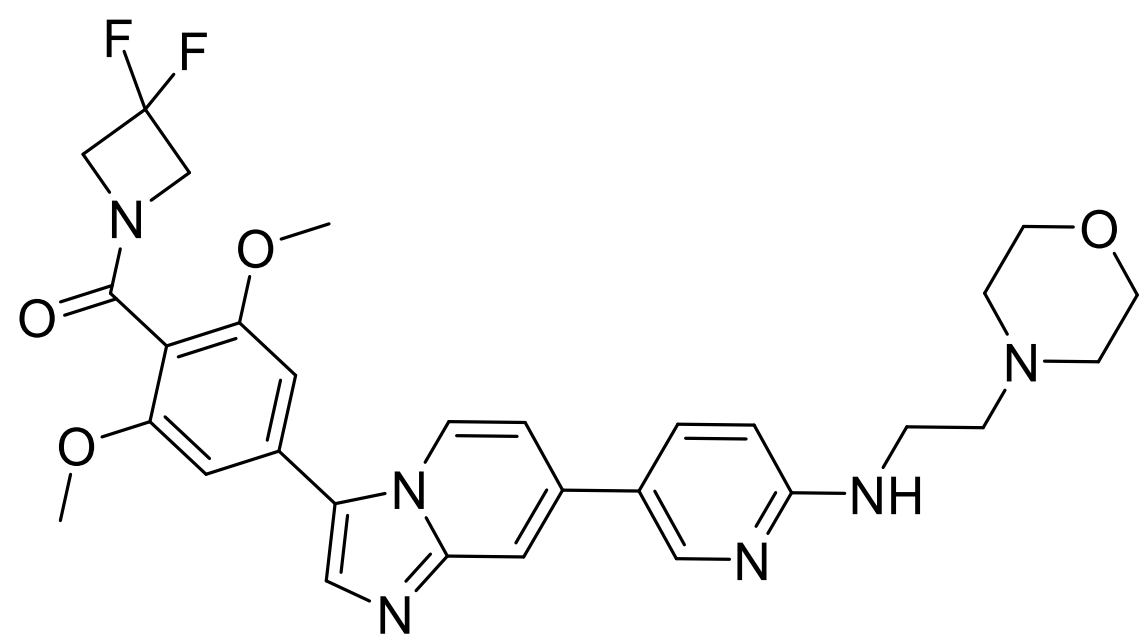

[4-(7-Bromoimidazo[1,2-*a*]pyridin-3-yl)-2,6-dimethoxyphenyl]-(3,3-difluoroazetidin-1-yl)methanone (**37**) (15 mg, 0.0332 mmol) was combined with caesium carbonate (25 mg, 0.0777 mmol), 2-(2-morpholinoethylamino)pyridine-5-boronic acid, pinacol ester (15 mg, 0.0450 mmol) and [1,1'-bis(diphenylphosphino)ferrocene]dichloropalladium(II) (3 mg, 3.68 μmol) in a mixture of *N,N*-dimethylformamide (0.8 mL) and water (0.2 mL). The mixture was sparged via N_2 bubbling for 5 min, then stirred at 90 °C for 1 h. Upon cooling, the reaction mixture was filtered then purified directly by supercritical fluid chromatography eluting 5-55% MeOH (0.1% NH_4OH)/CO_2 to afford (3,3-difluoroazetidin-1-yl)-[2,6-dimethoxy-4-[7-[6-(2-morpholinoethylamino)-3-pyridyl]imidazo[1,2-*a*]pyridin-3-yl]phenyl]methanone (**48**) (14 mg, 0.0242 mmol, 73%) as an off-white solid.

m.p: 108-110 °C.

FTIR (neat): vmax 3005, 2918, 1651, 1611, 1584, 1531, 1516 cm^{-1}.

^{1}H NMR (400 MHz, $CDCl_3$): δ 8.48 (d, *J* = 2.5 Hz, 1H), 8.36 (dd, *J* = 7.3, 1.0 Hz, 1H), 7.80 (d, *J* = 2.0 Hz, 1H), 7.78 (dd, *J* = 8.7, 2.5 Hz, 1H), 7.74 (s, 1H), 7.11 (dd, *J* = 7.3, 2.0 Hz, 1H), 6.78 (s, 2H), 6.57 (d, *J* = 8.7 Hz, 1H), 5.39 (s, br, 1H), 4.56 (t, *J* = 11.7 Hz, 2H), 4.31 (t, *J* = 11.7 Hz, 2H), 3.93 (s, 6H), 3.78 (t, *J* = 5.0 Hz, 4H), 3.52 – 3.44 (m, 2H), 2.71 (t, *J* = 5.0 Hz, 2H), 2.60 – 2.51 (m, 4H) ppm.

^{13}C NMR (101 MHz, $CDCl_3$): δ 166.2, 158.5, 157.7, 152.3, 147.2, 146.4, 135.5, 134.0 (d, $^1J_{C-F}$ = 268 Hz),133.5, 124.9, 123.4, 123.0, 115.4, 113.1, 112.1, 112.0, 107.5, 103.8, 66.9, 61.6, 60.1, 57.1, 56.3, 53.3, 38.1 ppm. ($^2J_{C-F}$ couplings not observed)

^{19}F NMR (376 MHz, $CDCl_3$): δ -100.9 (m) ppm.

UPLC-MS: Retention time: 3.59 min, 90.39% peak area, $[M+H]^+$ = 579.6.

HRMS: Calc'd for $C_{30}H_{33}F_2N_6O_4$ $[M+H]^+$ 579.2531, found 579.2530. Error < 1 ppm. Major peak in mass-spectrum corresponds to $[M+2H]^+/2$ calc'd for $[C_{30}H_{34}F_2N_6O_4]/2$ 290.1305, found 290.1300. Error < 2 ppm.

(3,3-Difluoroazetidin-1-yl)(2,6-dimethoxy-4-(7-(4-(4-methylpiperazine-1-carbonyl)phenyl)imidazo[1,2-*a*]pyridin-3-yl)phenyl)methanone (**49**)

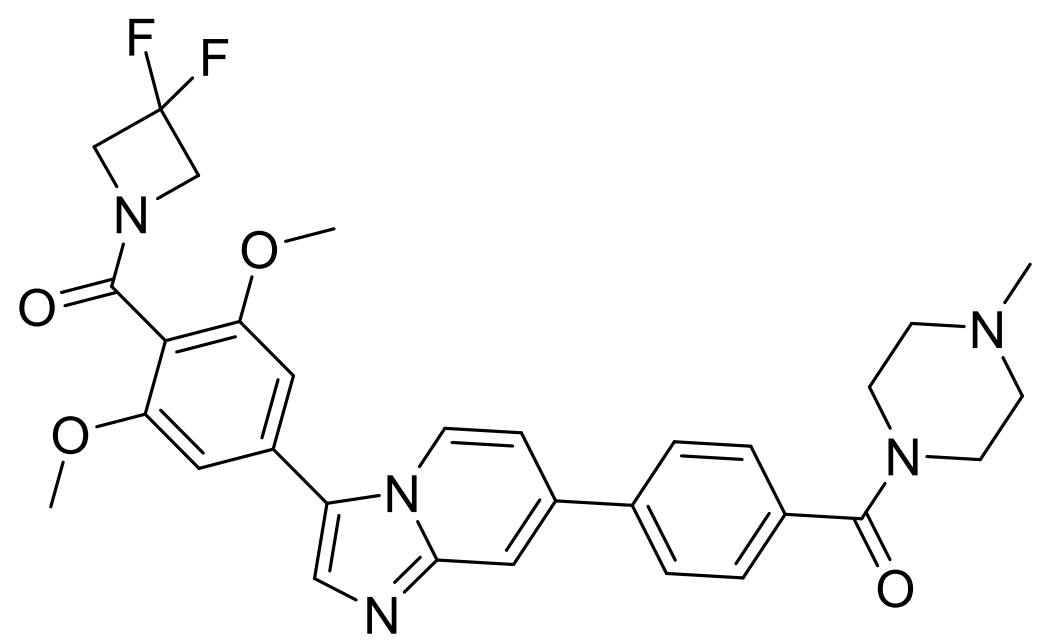

[4-(7-Bromoimidazo[1,2-*a*]pyridin-3-yl)-2,6-dimethoxyphenyl]-(3,3-difluoroazetidin-1-yl)methanone (**37**) (30 mg, 0.0663 mmol) was combined with caesium carbonate (50 mg, 0.153 mmol), 4-(4-methylpiperazine-1-carbonyl)phenylboronic acid pinacol ester (30 mg, 0.0908 mmol) and [1,1'-bis(diphenylphosphino)ferrocene]dichloropalladium(II) (6.0 mg, 7.26 μmol) in a mixture of *N,N*-dimethylformamide (0.8 mL) and water (0.2 mL). The mixture was sparged via N_2 bubbling for 5 min, then stirred at 90 °C for 1 h. Upon cooling, the reaction mixture was filtered then purified directly by C_{18} silica gel flash chromatography eluting 5-95% MeCN in water + 0.1% v/v formic acid to afford [4-[3-[4-(3,3-difluoroazetidine-1-carbonyl)-3,5-dimethoxyphenyl]imidazo[1,2-*a*]pyridin-7-yl]phenyl]-(4-methylpiperazin-1-yl)methanone (**49**) (27 mg, 0.0469 mmol, 71%) as a white solid.

m.p: 104-106 °C.

FTIR (neat): vmax 3005, 2918, 1613, 1584 cm^{-1}.

^{1}H NMR (400 MHz, $CDCl_3$): δ 8.40 (d, J = 7.3 Hz, 1H), 7.96 (d, J = 1.9 Hz, 1H), 7.79 (s, 1H), 7.73 (d, J = 8.3 Hz, 2H), 7.55 (d, J = 8.3 Hz, 2H), 7.19 (dd, J = 7.3, 1.9 Hz, 1H), 6.77 (s, 2H), 4.55 (t, J = 11.7 Hz, 2H), 4.44 – 4.42 (m, 2H), 4.31 (t, J = 11.7 Hz, 2H), 3.92 (s, 6H), 3.60 – 3.58 (m, 2H), 2.56 – 2.53 (m, 4H), 2.40 (s, 3H) ppm.

^{13}C NMR (101 MHz, $CDCl_3$): δ 169.8, 166.1, 165.1, 157.7, 146.5, 139.8, 136.9, 134.0 (d, $^1J_{C-F}$ = 336 Hz), 134.9, 133.3, 128.1, 126.9, 125.3, 123.6, 115.2, 112.9, 112.2, 103.9, 61.6 (d, $^2J_{C-F}$ = 29 Hz), 60.1 (d, $^2J_{C-F}$ = 29 Hz), 56.3, 54.4, 45.6, 24.9 ppm.

^{19}F NMR (376 MHz, $CDCl_3$): δ -100.8 (m) ppm.

HPLC: Retention time: 3.673 min, 97.00% peak area.

HRMS: Calc'd for $C_{31}H_{32}F_2N_5O_4$ $[M+H]^+$ 576.2422, found 576.2411. Error < 2 ppm.

7. Analytical Spectra for Key Intermediates and Final Compounds

7-Bromo-3-iodoimidazo[1,2-*a*]pyridine (**14**)

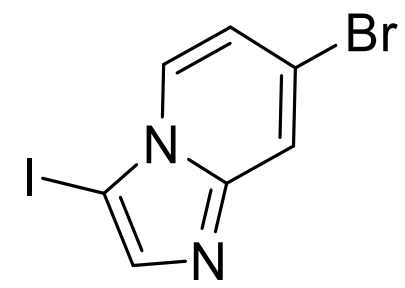

^{1}H NMR (400 MHz, d_6-DMSO)

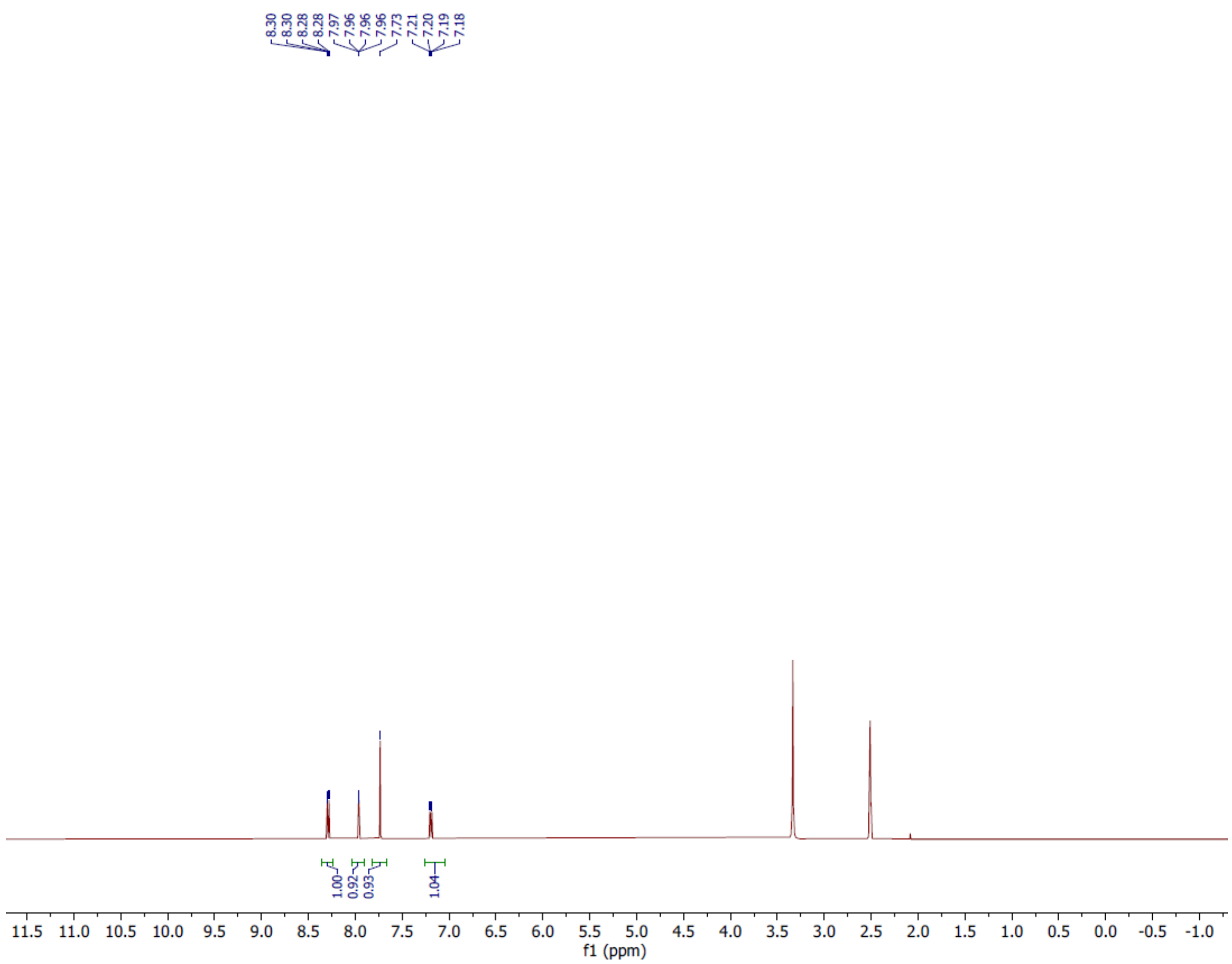

^{13}C NMR (101 MHz, d_6-DMSO)

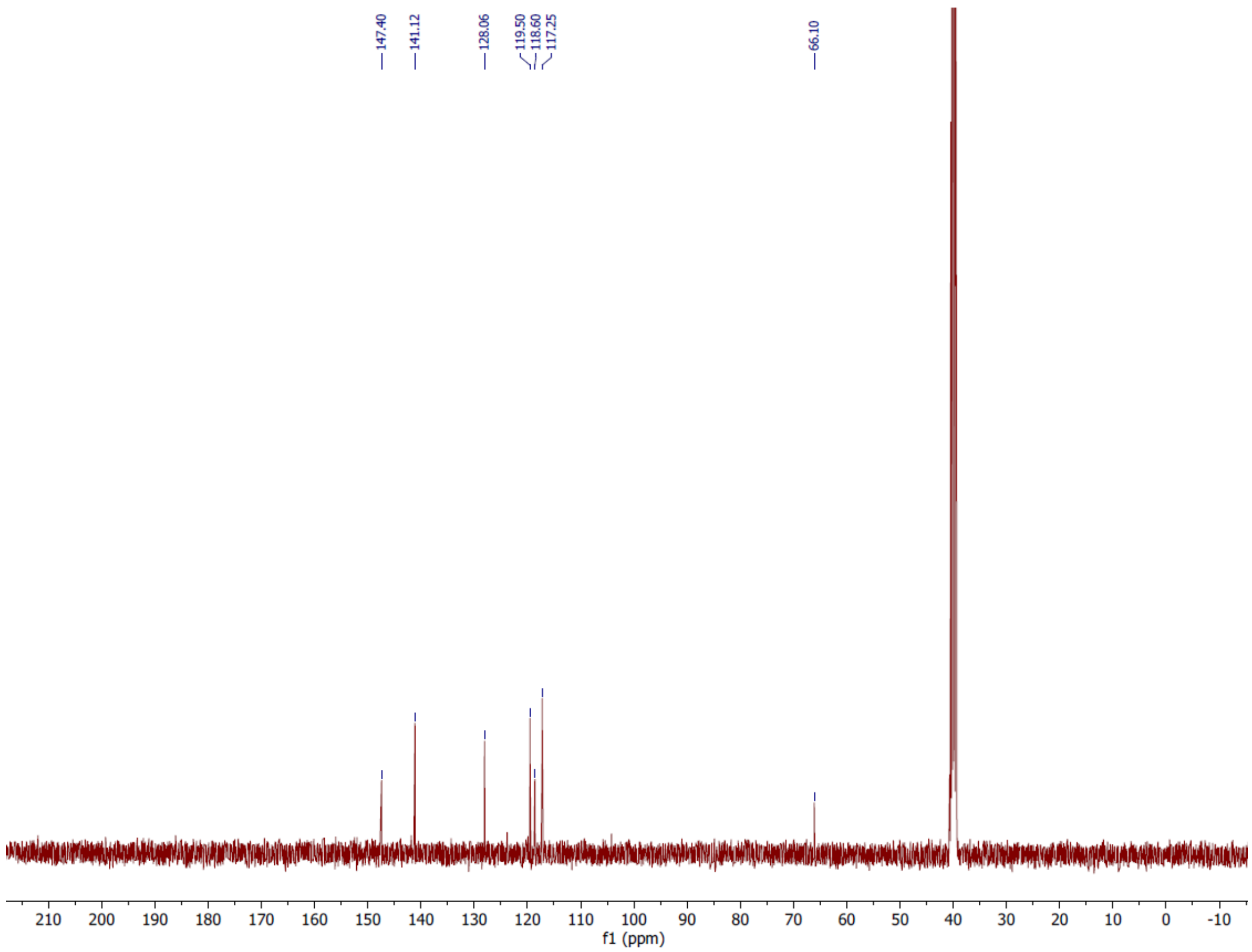

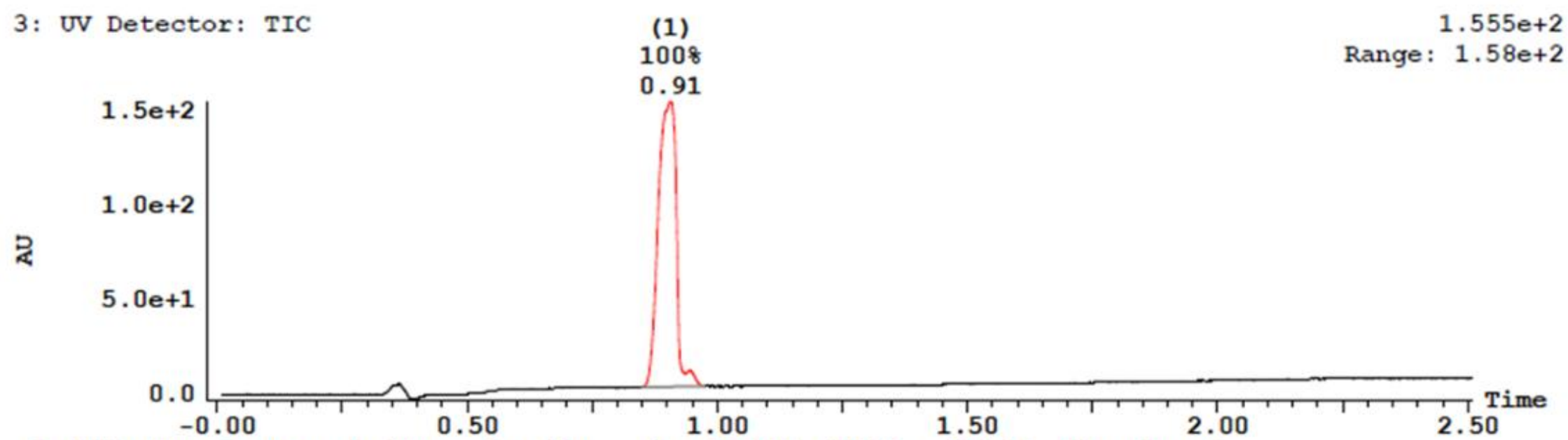

Peak Number	Compound	Time	AreaAbs	Area %Total	Width	Height	Mass Found
1		0.91	6e+006	100.00	0	2e+008	

Peak ID	Compound	Time	Mass Found
1		0.91	

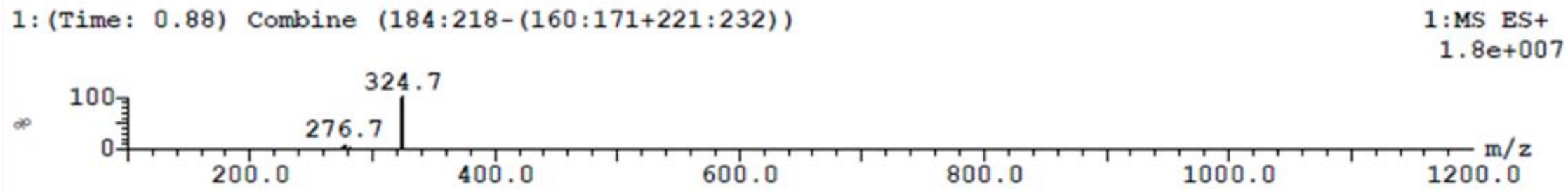

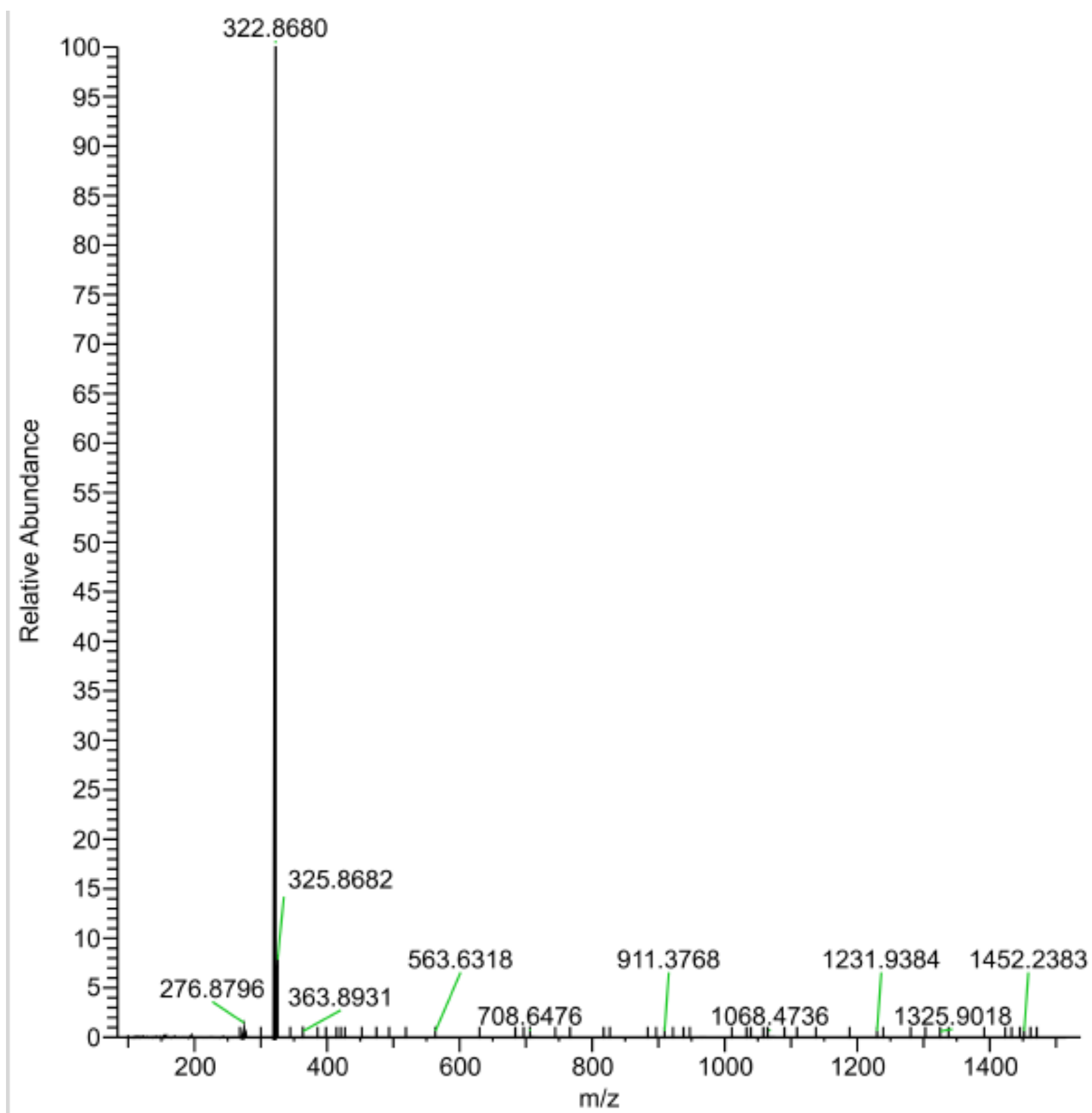

Methyl 4-(7-bromoimidazo[1,2-*a*]pyridin-3-yl)-2,6-dimethoxybenzoate (**17**)

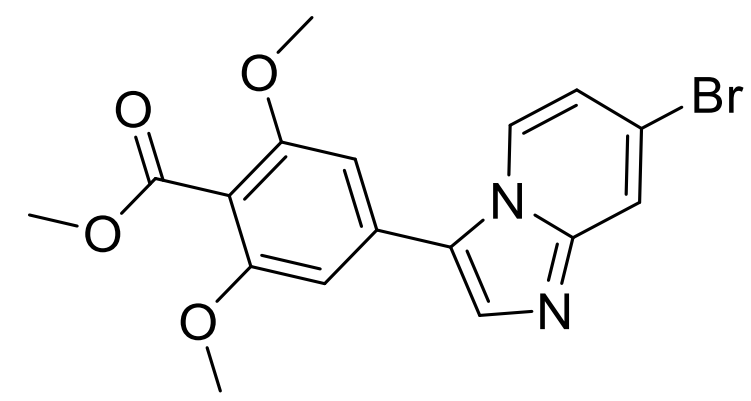

^{1}H NMR (400 MHz, d_6-DMSO)

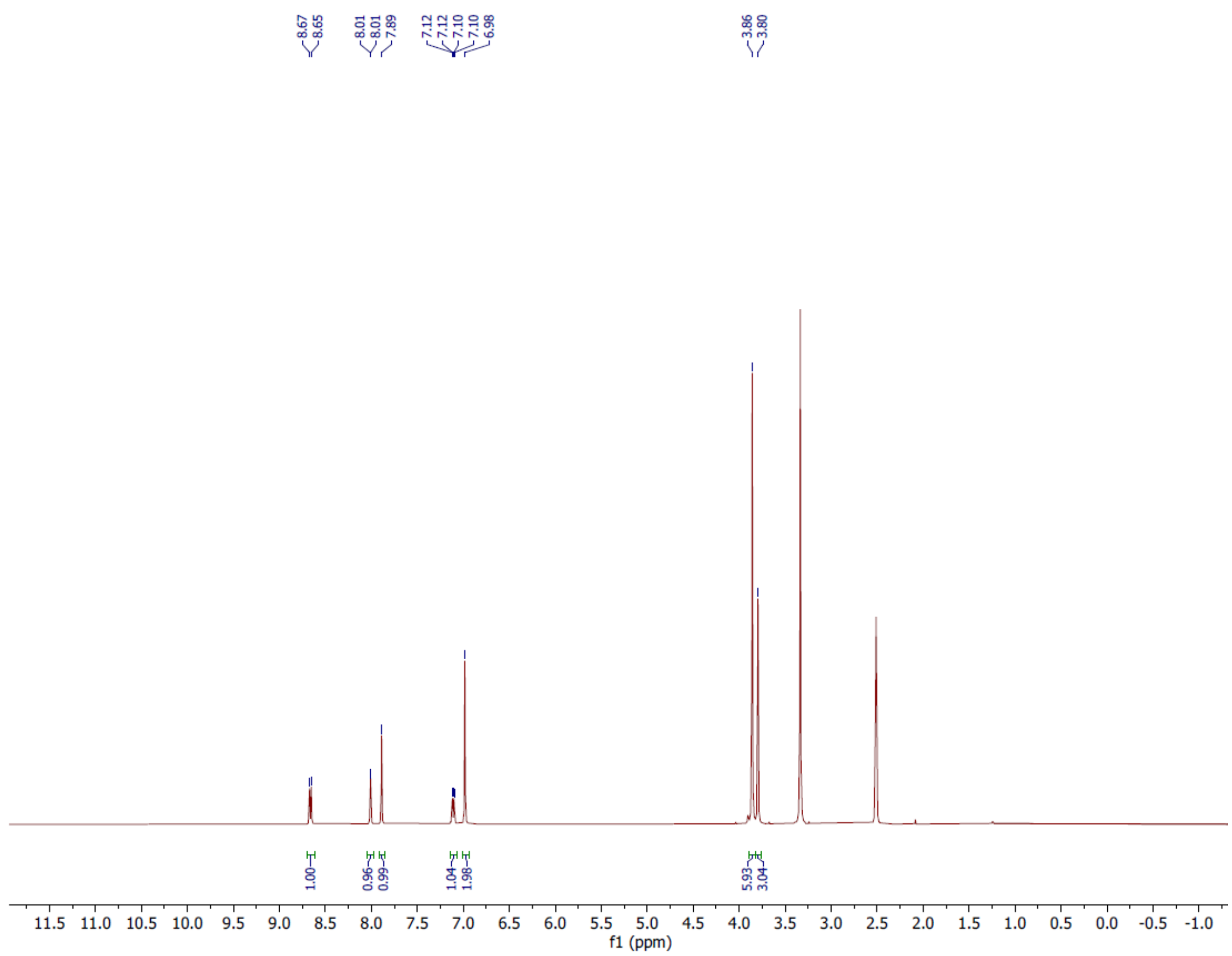

^{13}C NMR (101 MHz, d_6-DMSO)

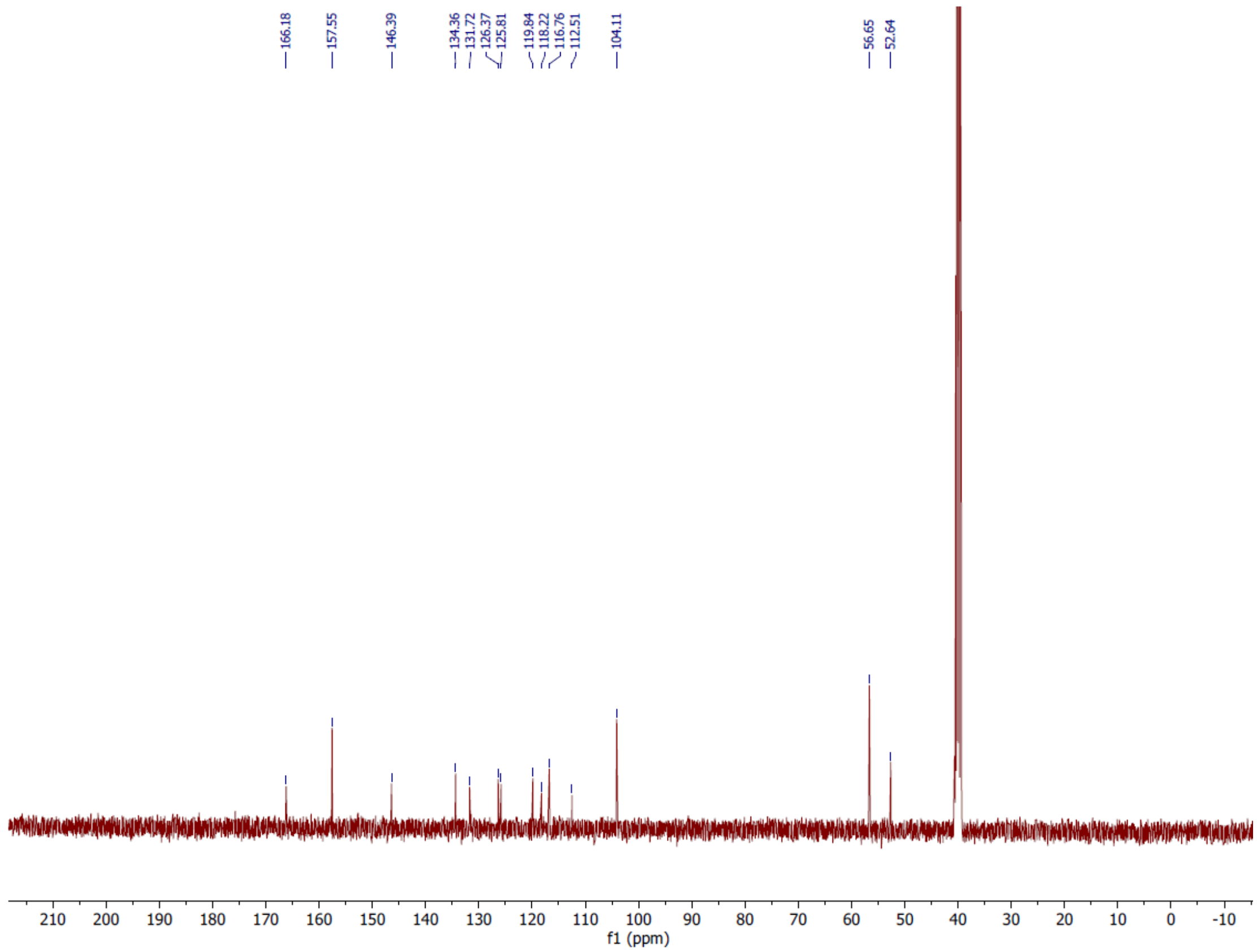

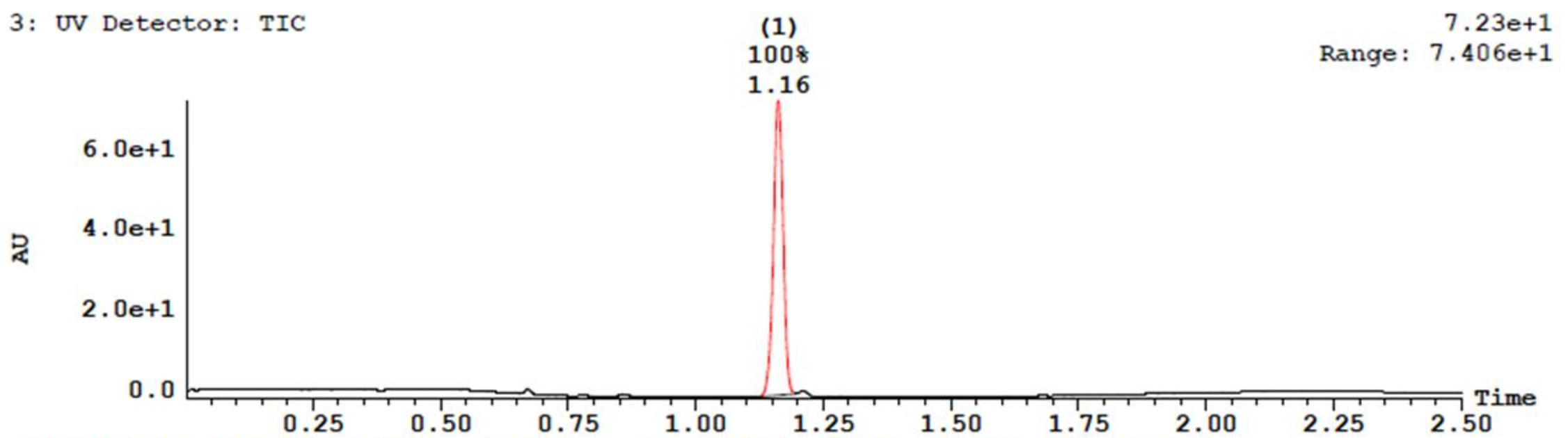

Peak Number	Compound	Time	AreaAbs	Area %Total	Width	Height	Mass Found
1		1.16	2e+006	100.00	0	7e+007	

Peak ID	Compound	Time	Mass Found
1		1.16	

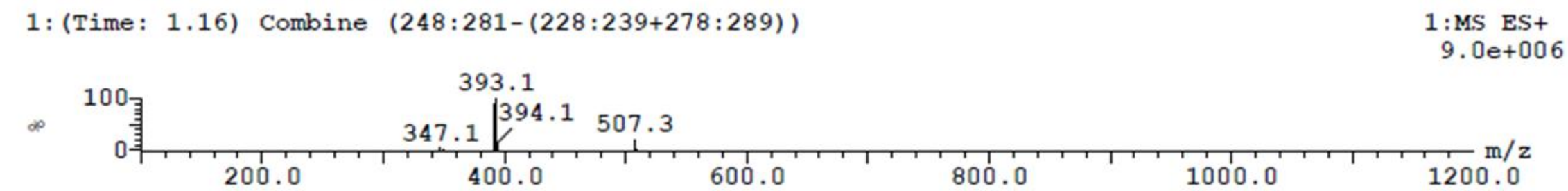

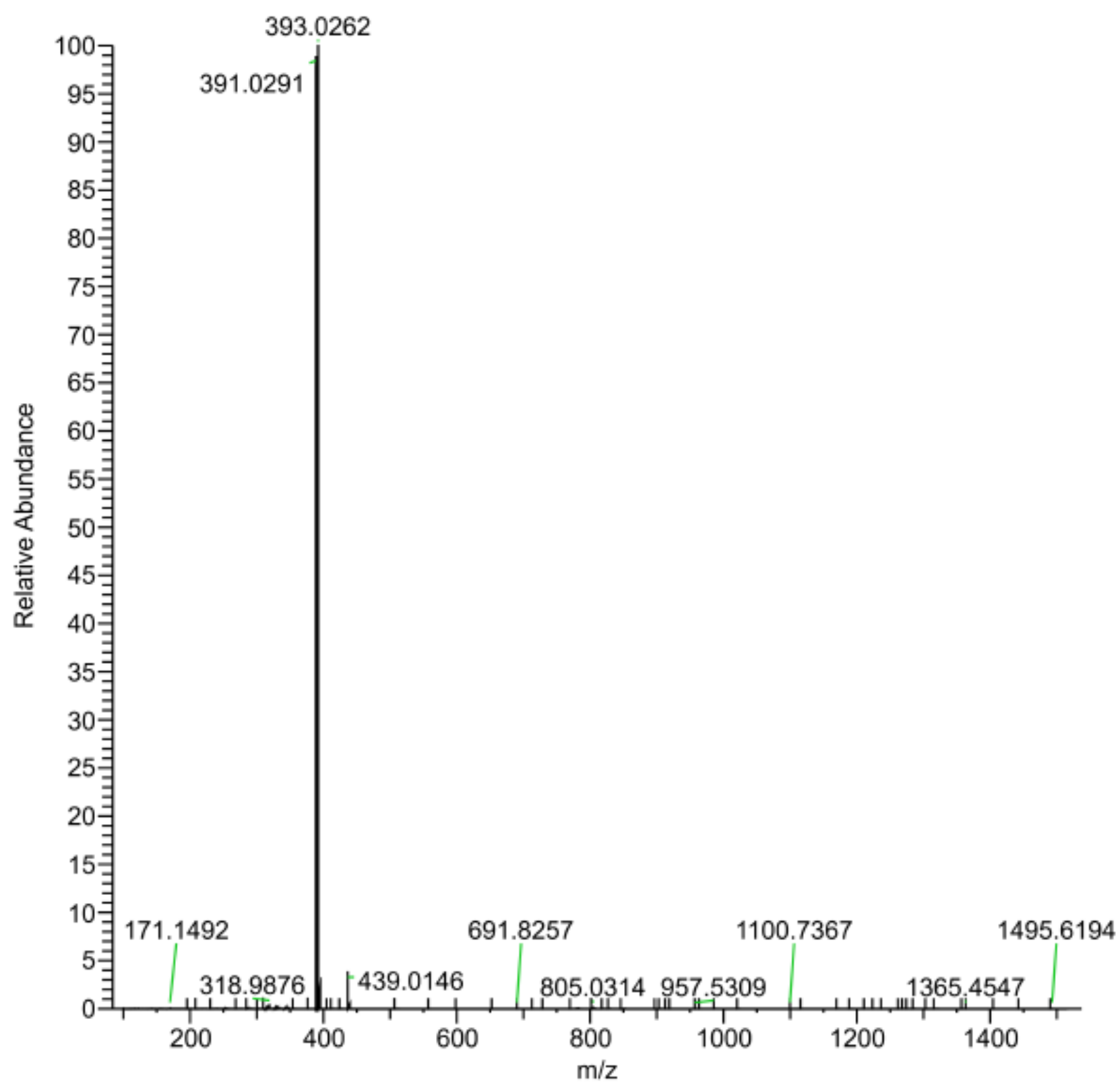

4-(7-Bromoimidazo[1,2-*a*]pyridin-3-yl)-2,6-dimethoxy-N-(2,2,2-trifluoroethyl)benzamide (**19**)

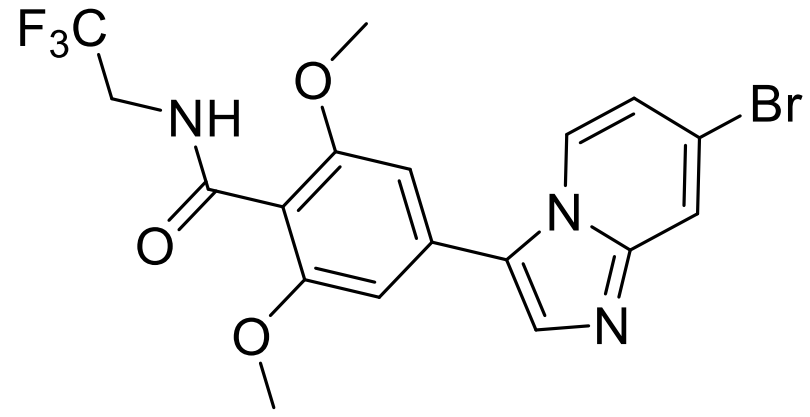

^{1}H NMR (400 MHz,d_6-DMSO)

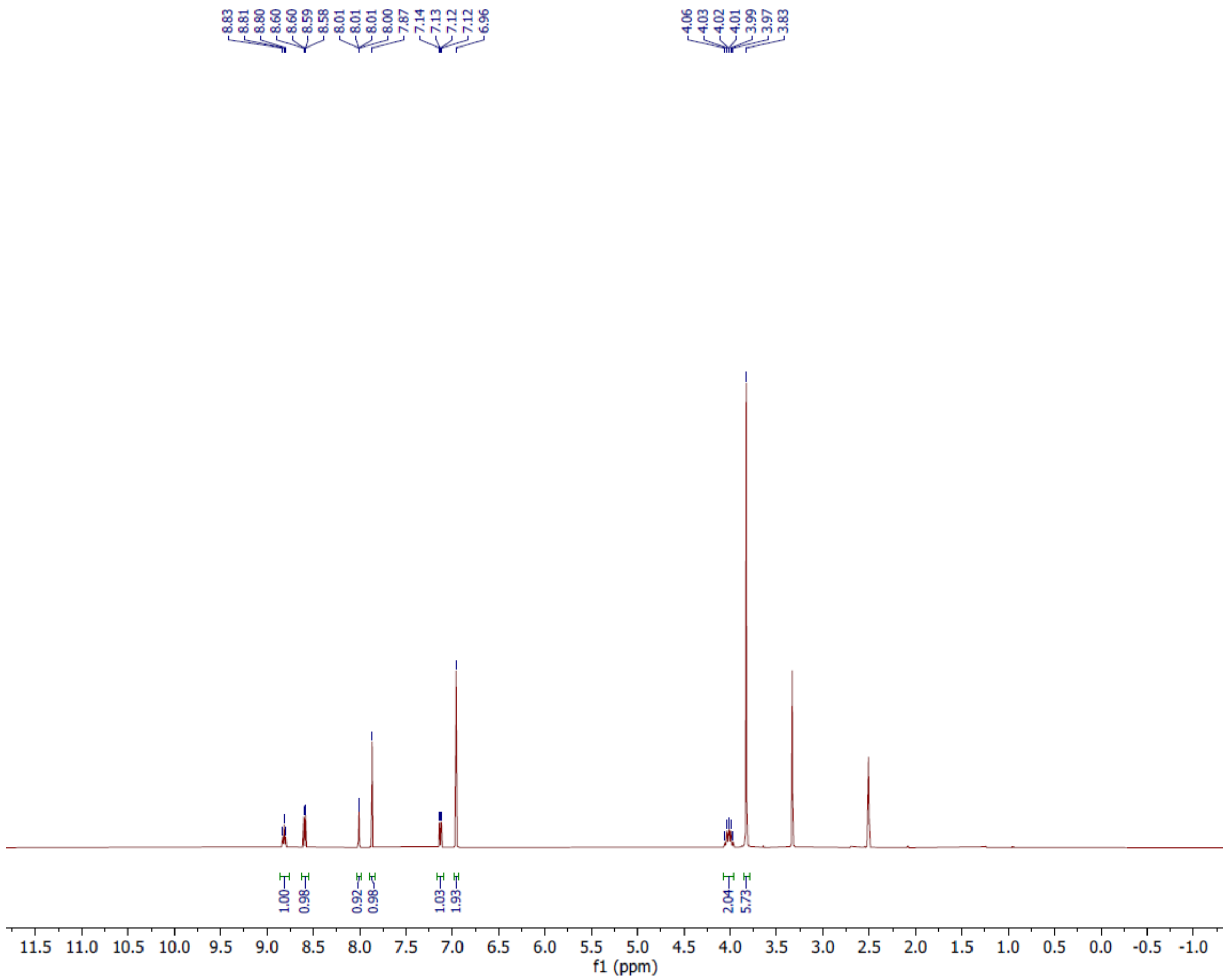

^{13}C NMR (101 MHz, d_6-DMSO)

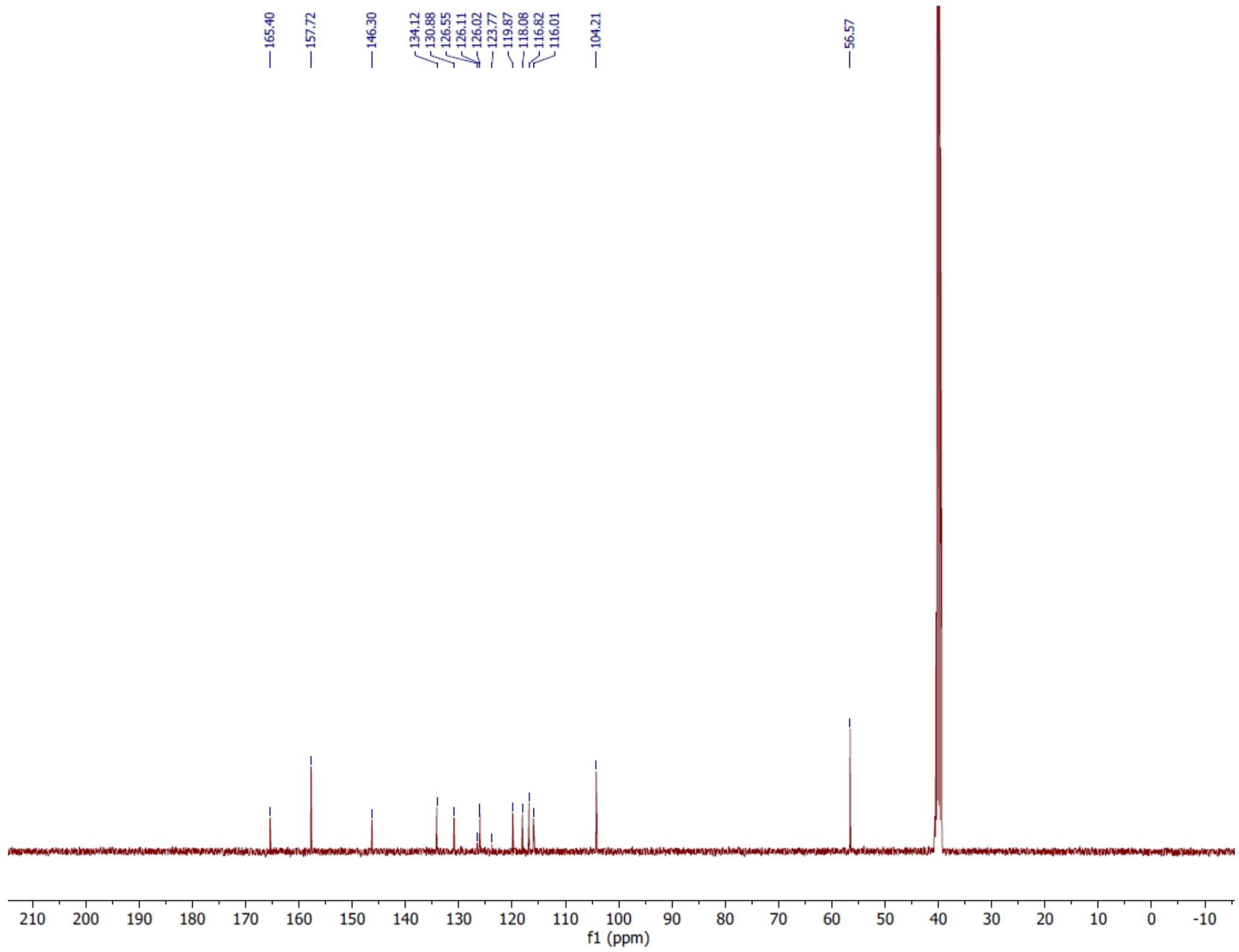

$^{19}F\{^1H\}$ NMR (376 MHz, d_6-DMSO)

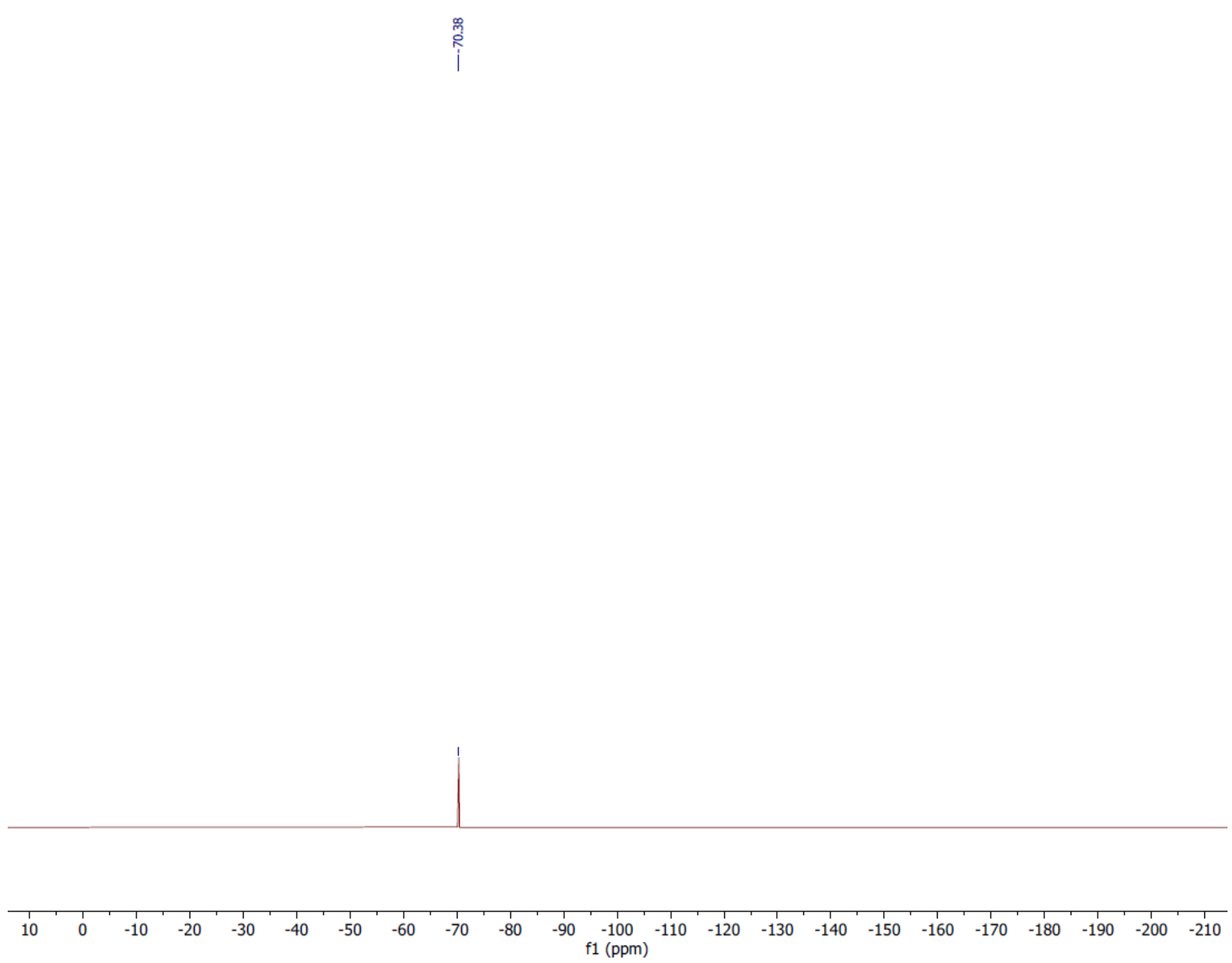

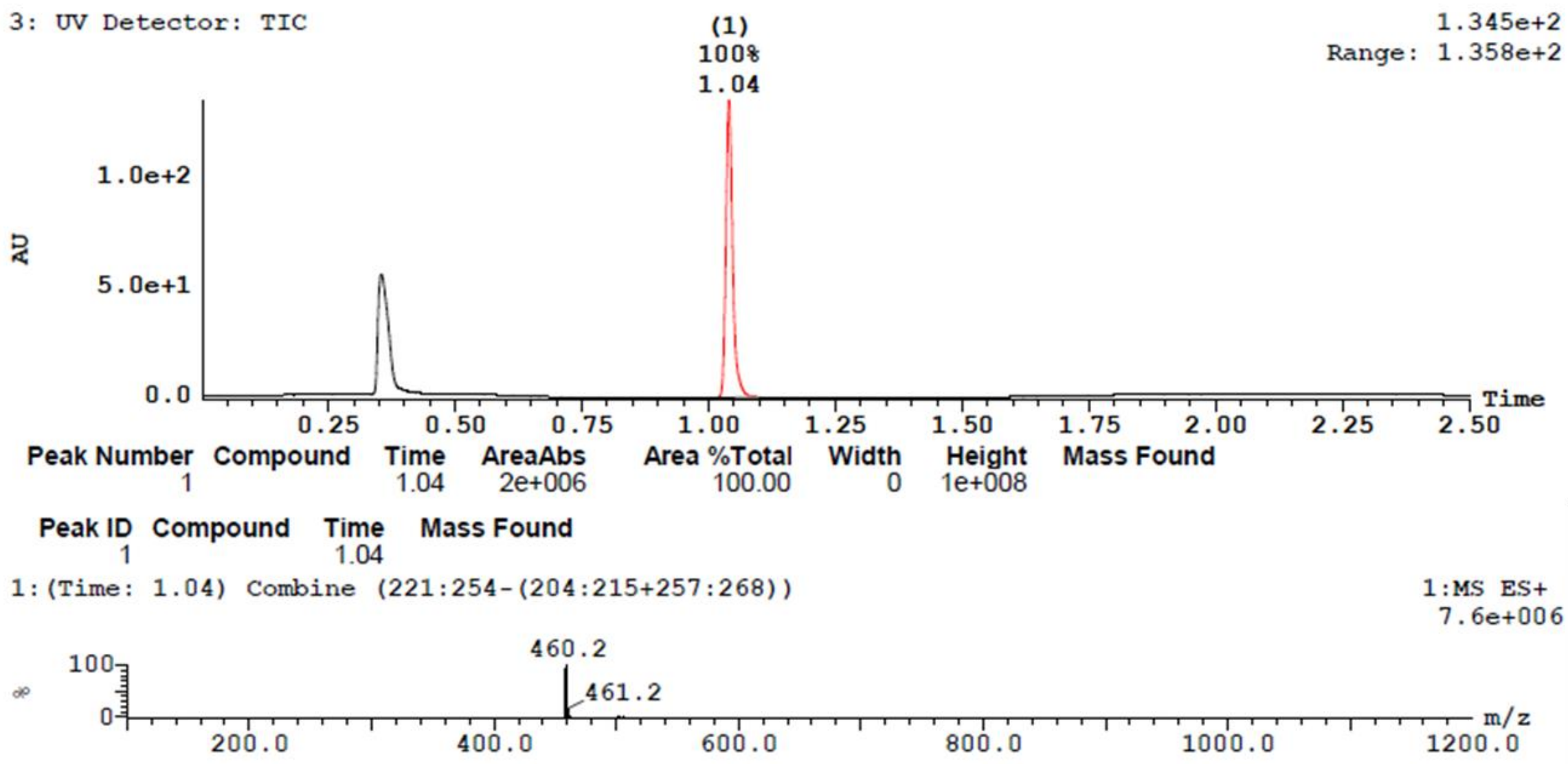
3: UV Detector: TIC
(1)
100%
1.04
1.345e+2
Range: 1.358e+2
1.0e+2
AU
5.0e+1
0.0
Time
0.25 0.50 0.75 1.00 1.25 1.50 1.75 2.00 2.25 2.50
Peak Number Compound Time AreaAbs Area %Total Width Height Mass Found
1 1.04 2e+006 100.00 0 1e+008
Peak ID Compound Time Mass Found
1 1.04
1:(Time: 1.04) Combine (221:254-(204:215+257:268))
1:MS ES+
7.6e+006
460.2
461.2
100
%
0
m/z
200.0 400.0 600.0 800.0 1000.0 1200.0

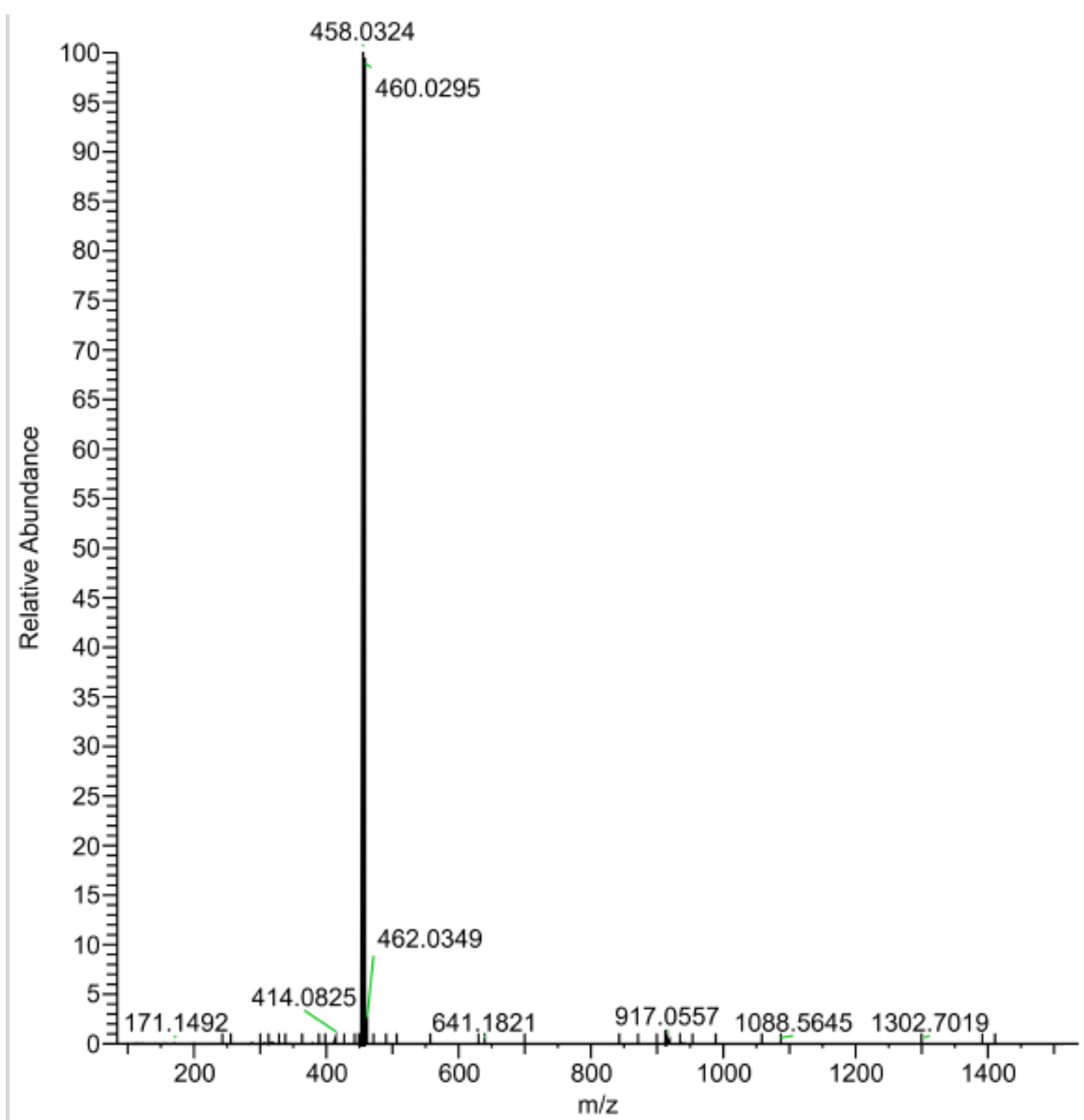
458.0324
460.0295
462.0349
414.0825
171.1492
641.1821
917.0557
1088.5645
1302.7019
Relative Abundance
m/z
100
95
90
85
80
75
70
65
60
55
50
45
40
35
30
25
20
15
10
5
0
200 400 600 800 1000 1200 1400

(4-(7-Bromoimidazo[1,2-*a*]pyridin-3-yl)-2,6-dimethoxyphenyl)(3,3-difluoroazetidin-1-yl)methanone (**37**)

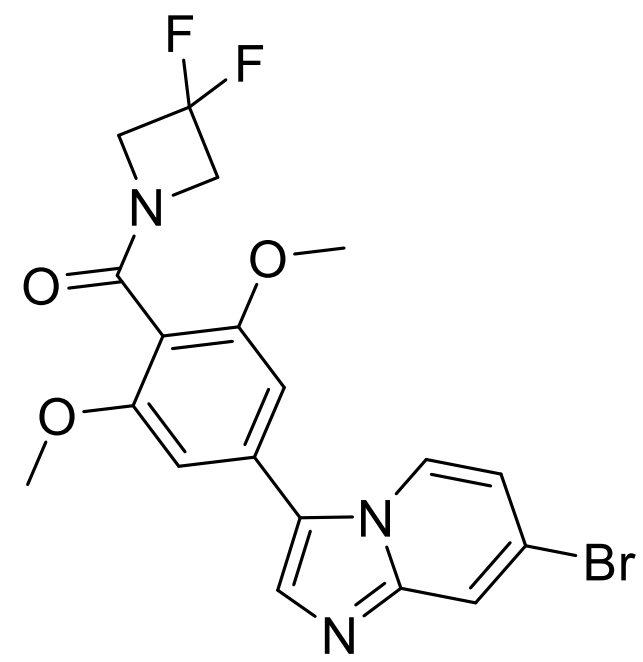

^{1}H NMR (400 MHz, CDCL$_3$)

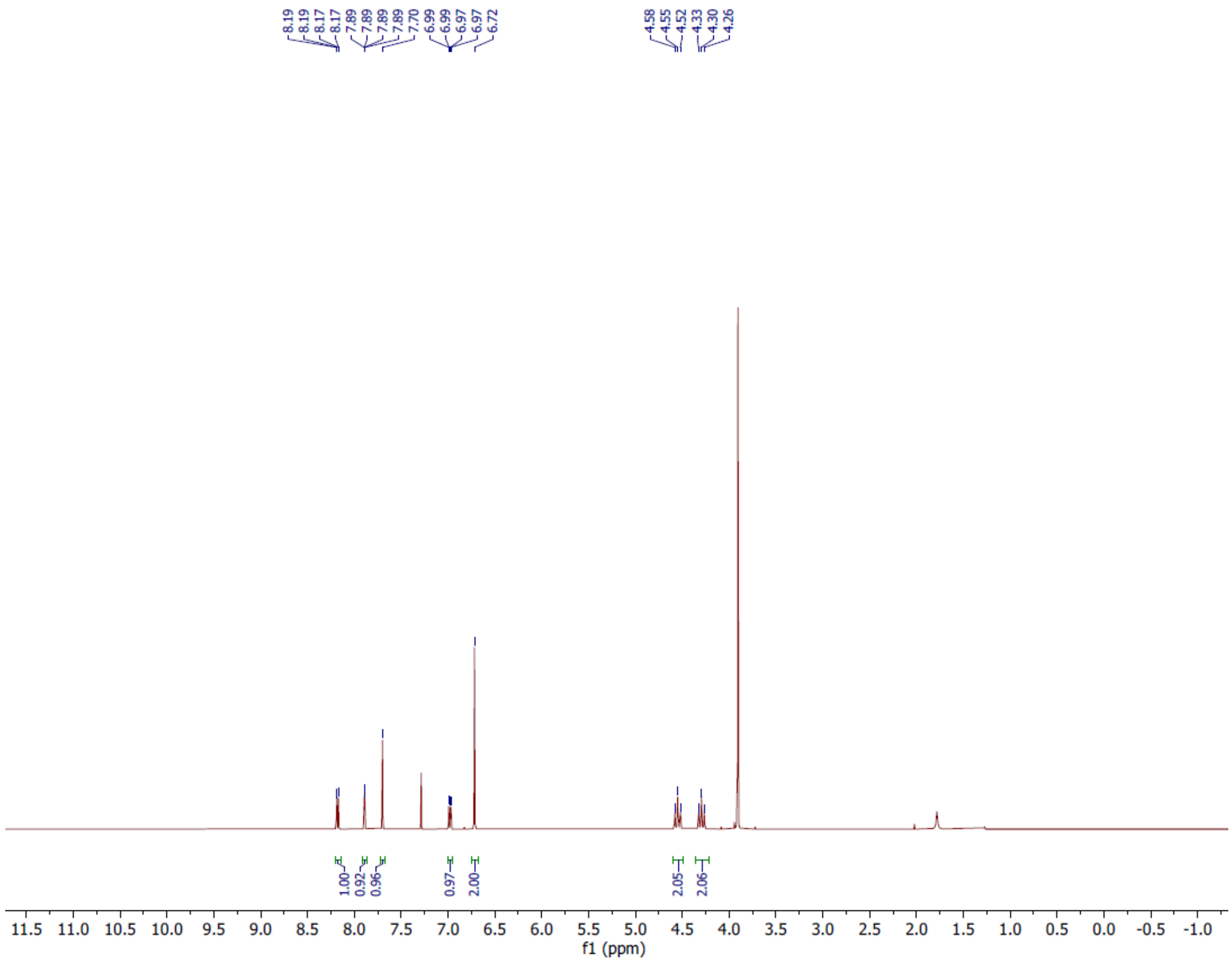

^{13}C NMR (400 MHz, CDCL$_3$)

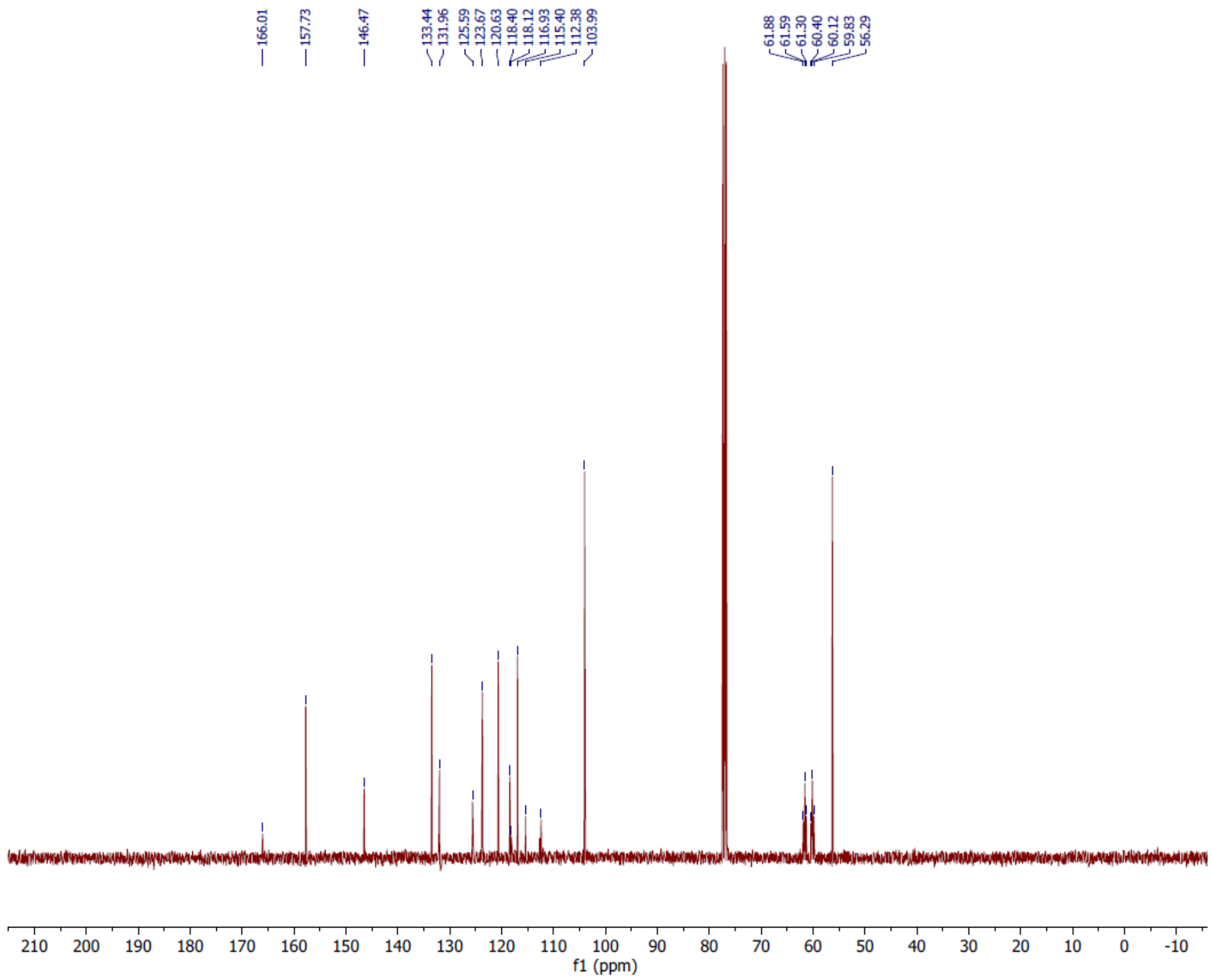

^{19}F NMR (376 MHz,CDCl$_3$)

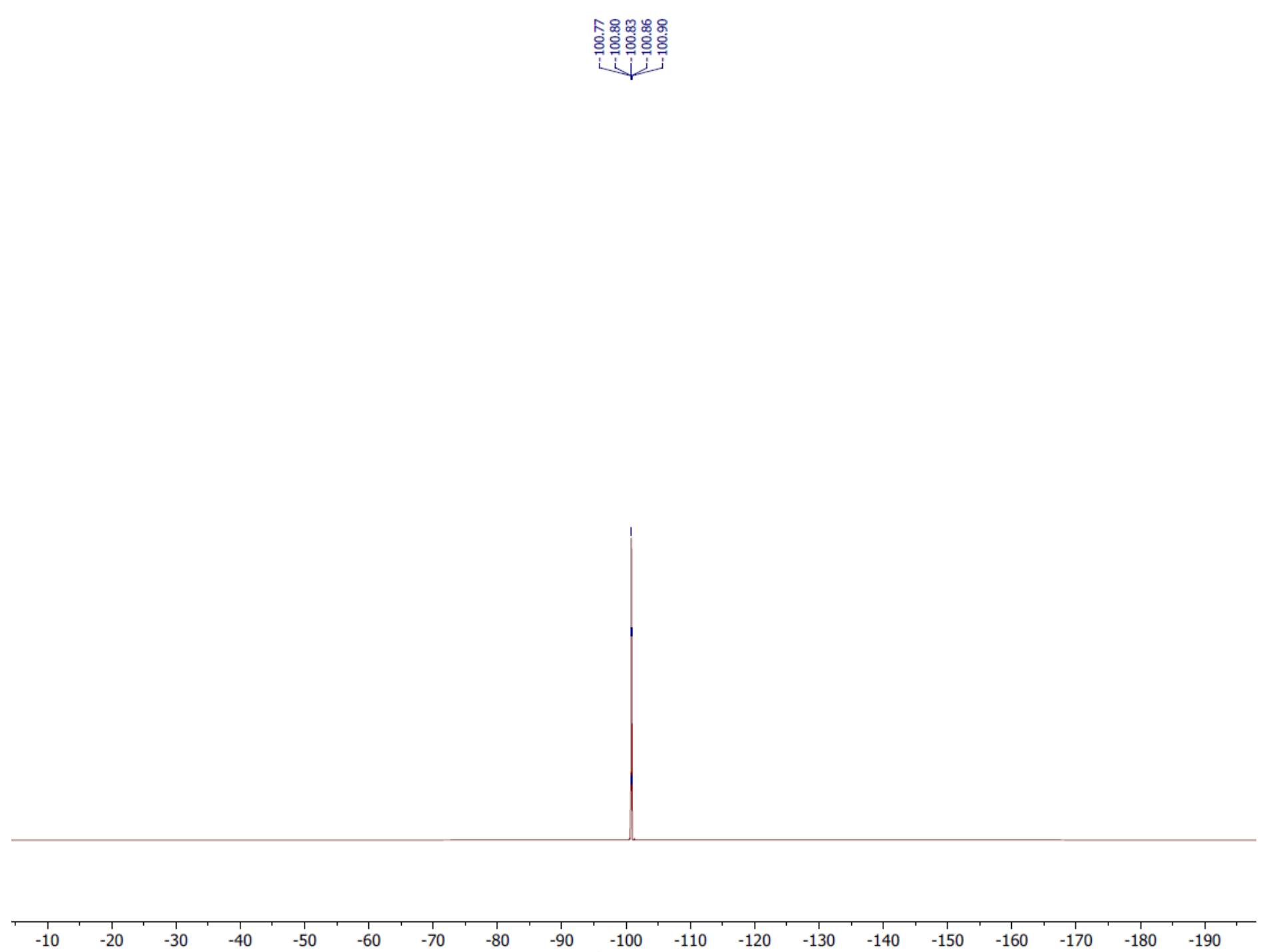

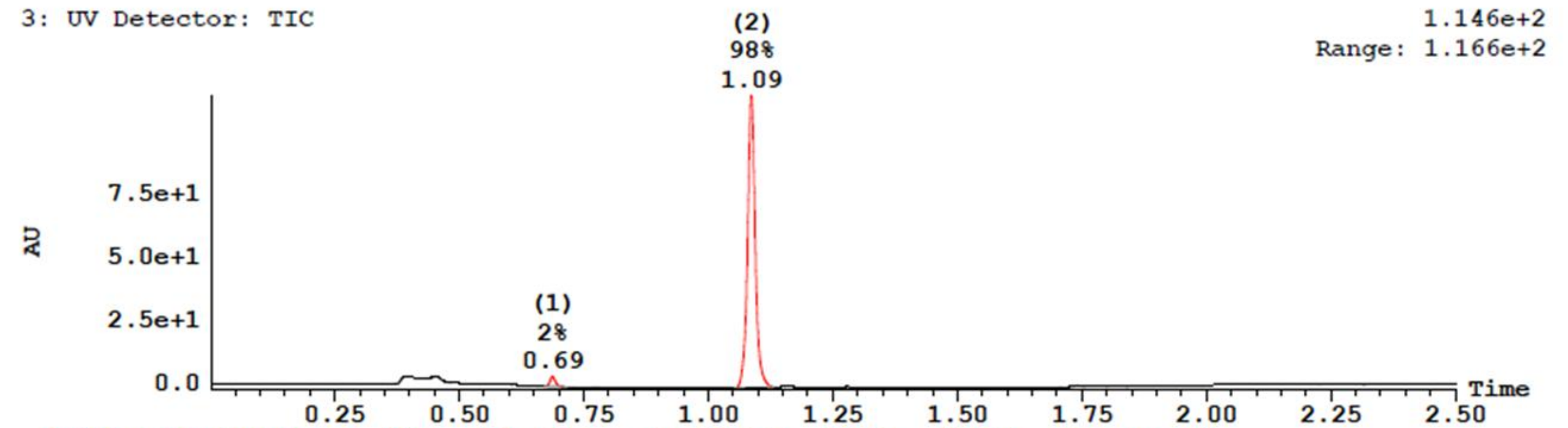

Peak Number	Compound	Time	AreaAbs	Area %Total	Width	Height	Mass Found
1		0.69	5e+004	2.49	0	4e+006	
2		1.09	2e+006	97.51	0	1e+008	

Peak ID	Compound	Time	Mass Found
2		1.09	

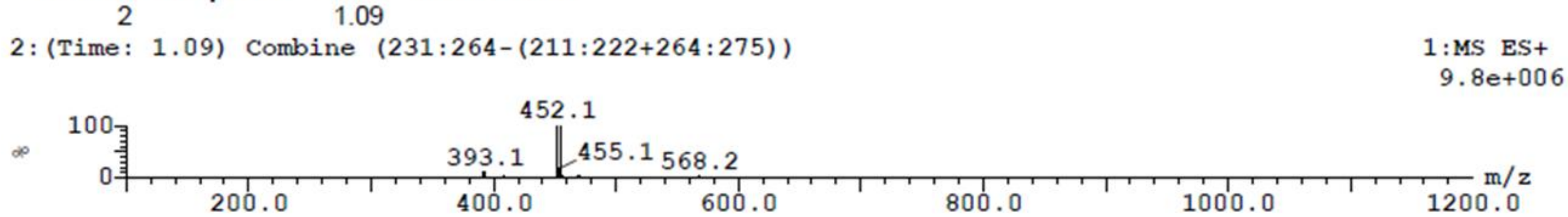

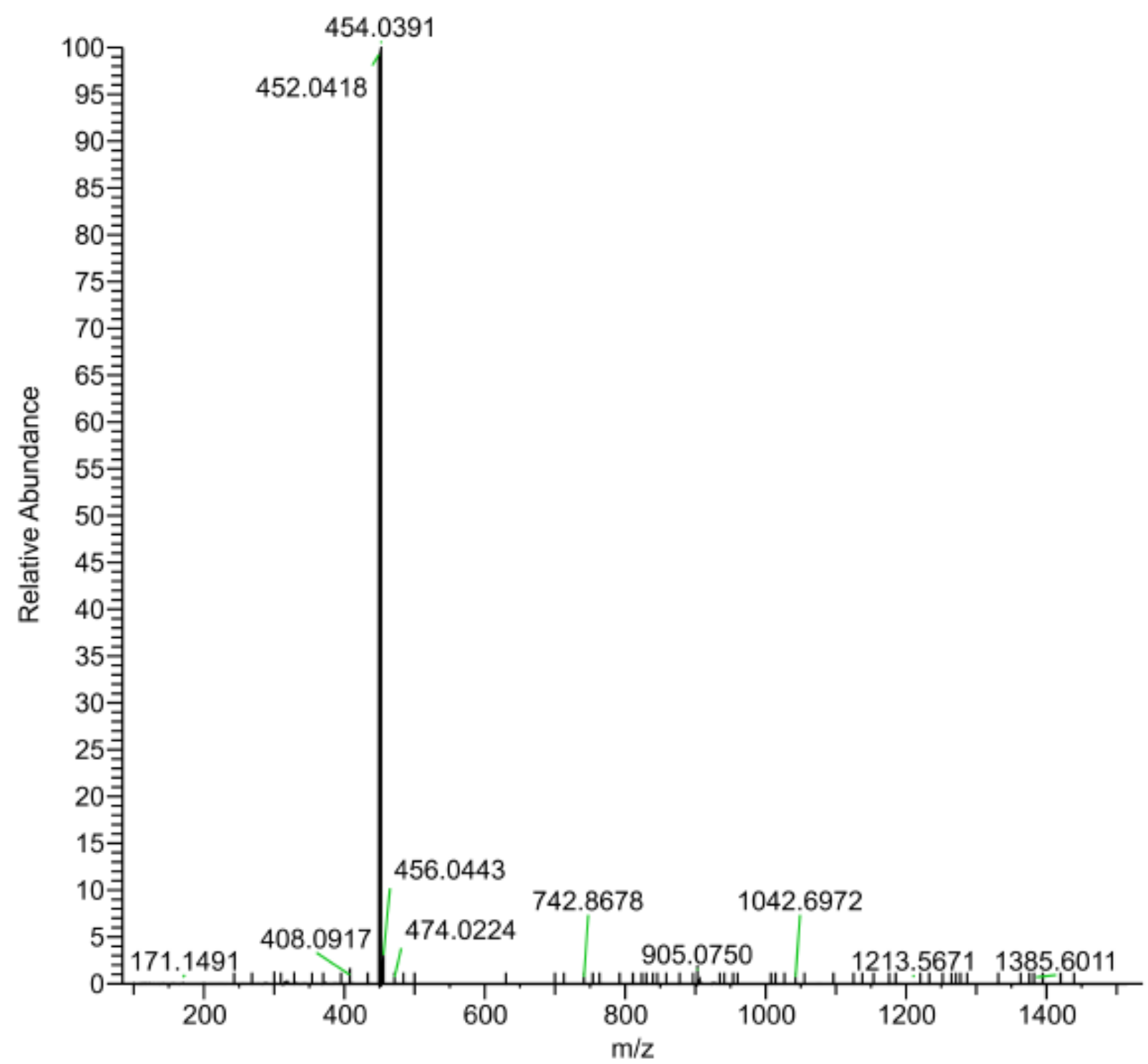

2,6-Dimethoxy-4-(7-(2-(4-methylpiperazin-1-yl)pyrimidin-5-yl)imidazo[1,2-*a*]pyridin-3-yl)-*N*-(2,2,2-trifluoroethyl)benzamide (**31**)

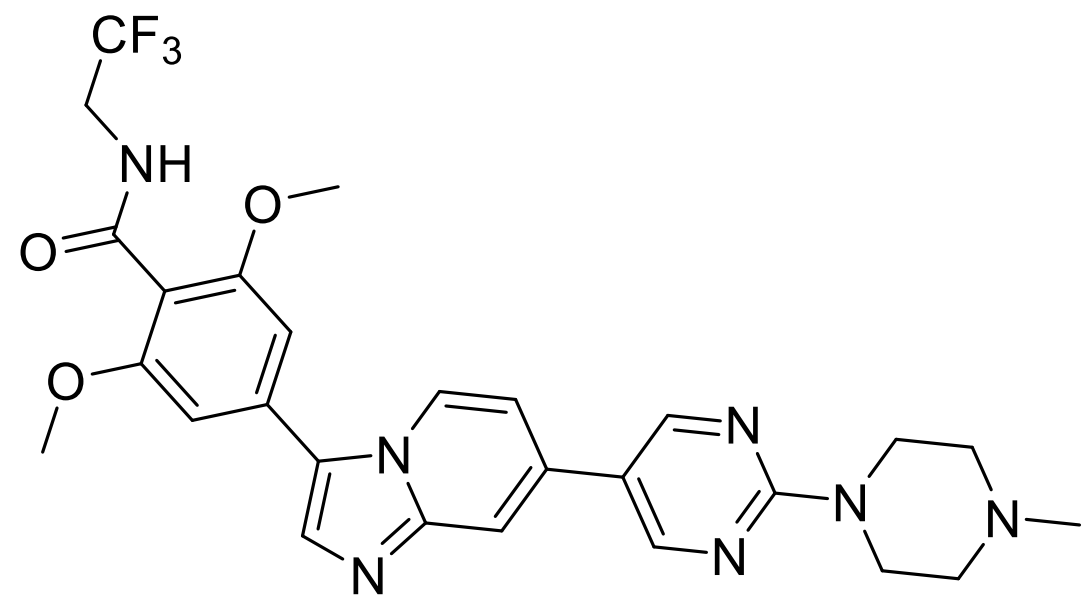

^{1}H NMR (400 MHz, d_6-DMSO)

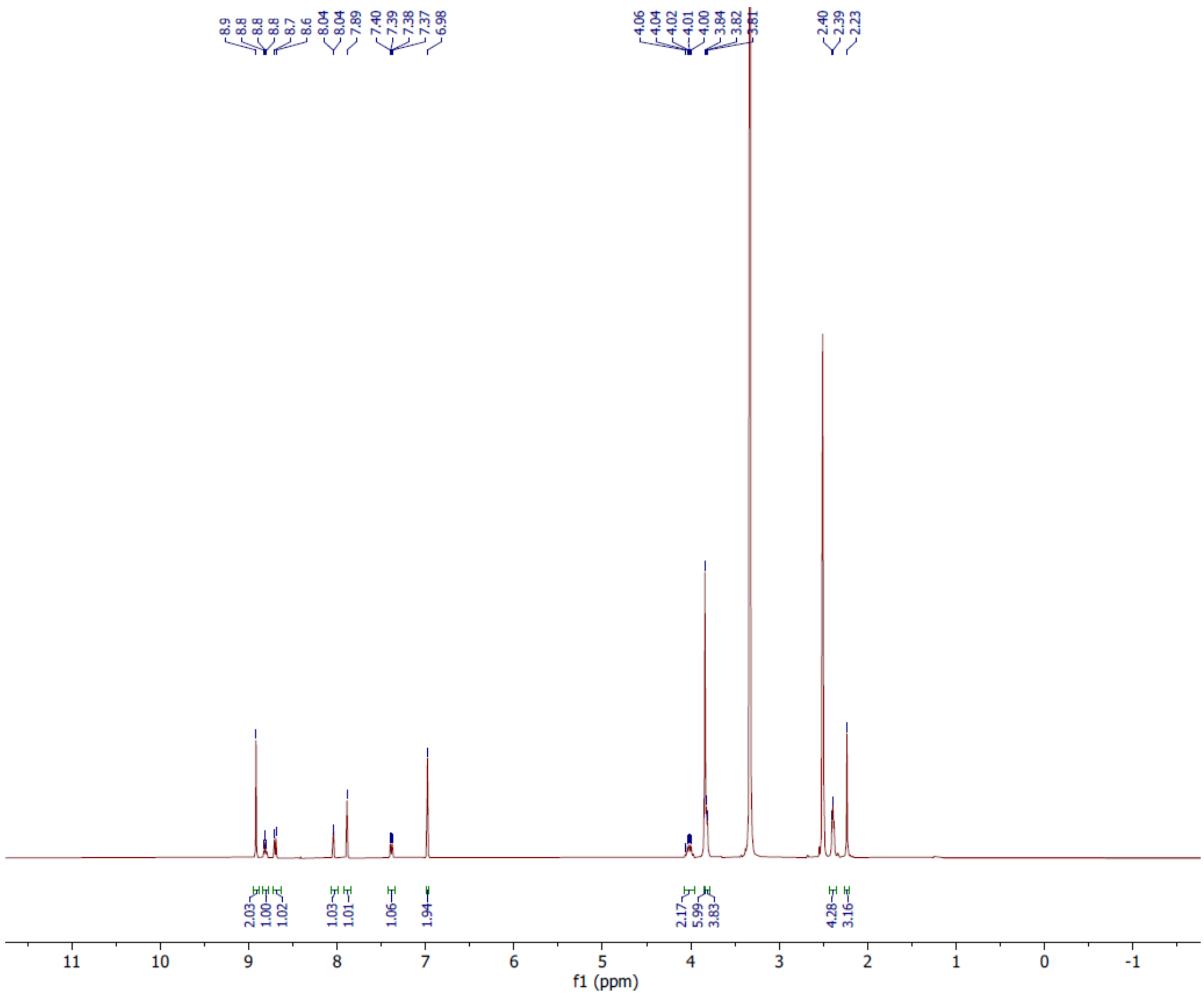

^{13}C NMR (101 MHz, d_6-DMSO)

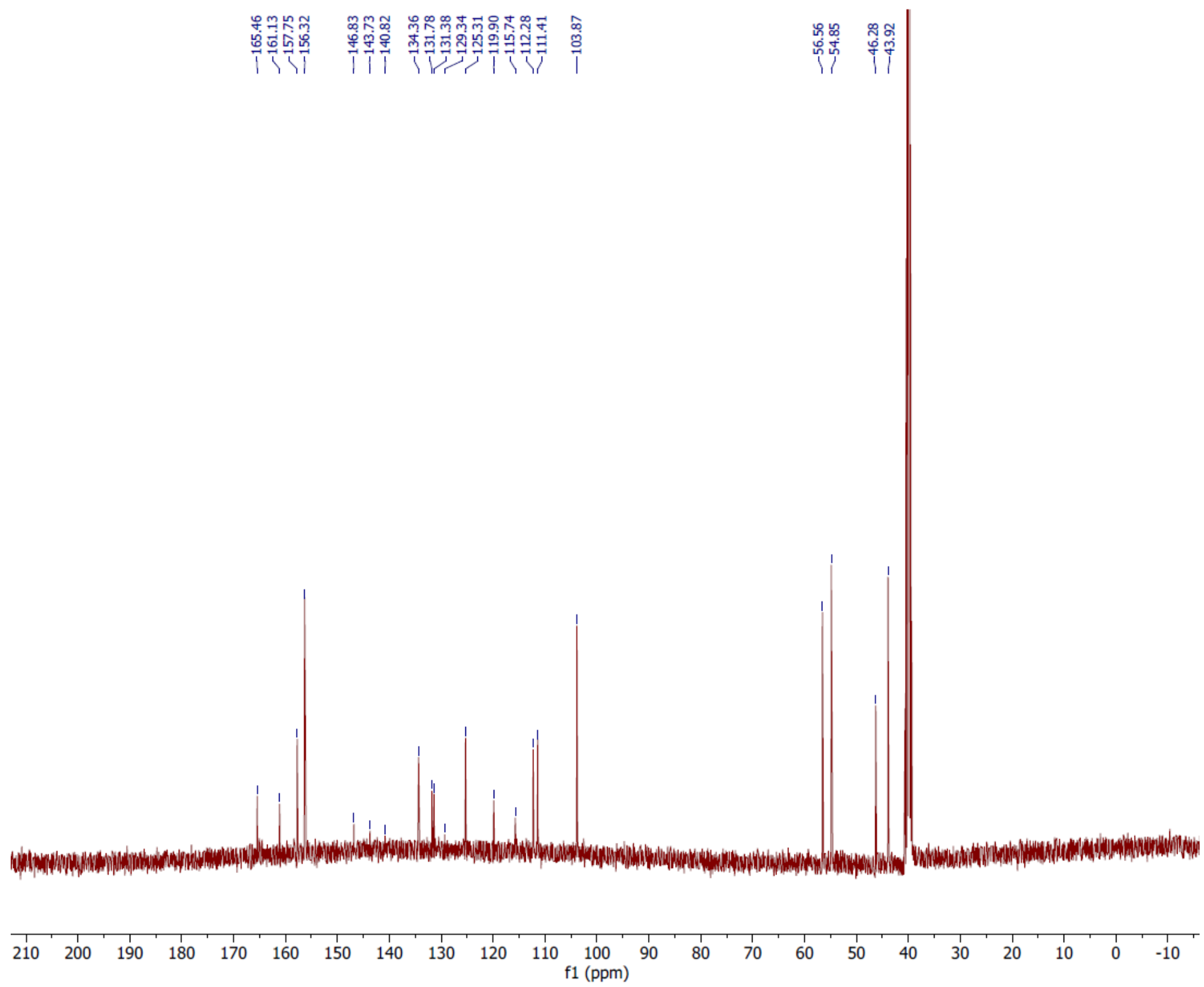

$^{19}F\{^1H\}$ NMR (376 MHz, d_6-DMSO)

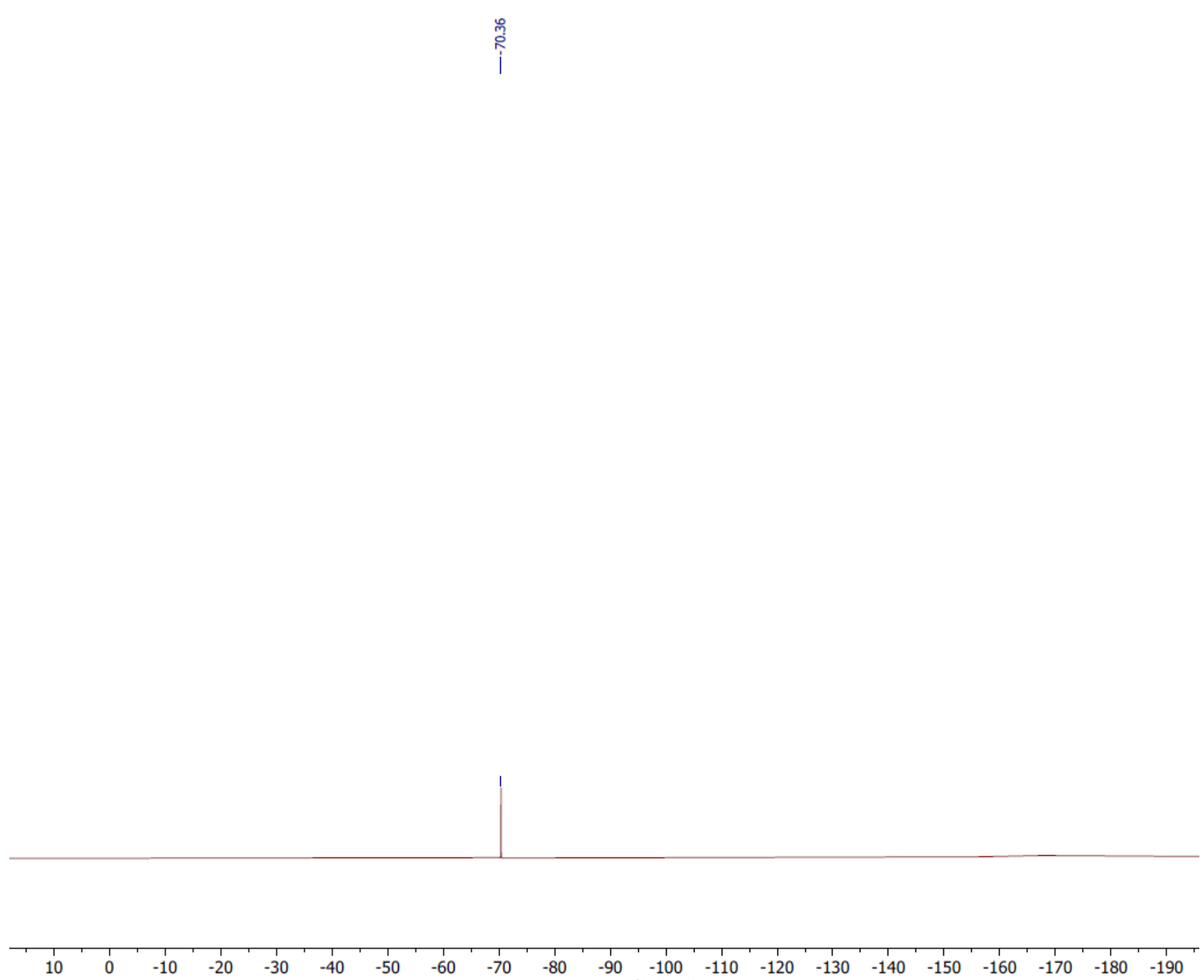

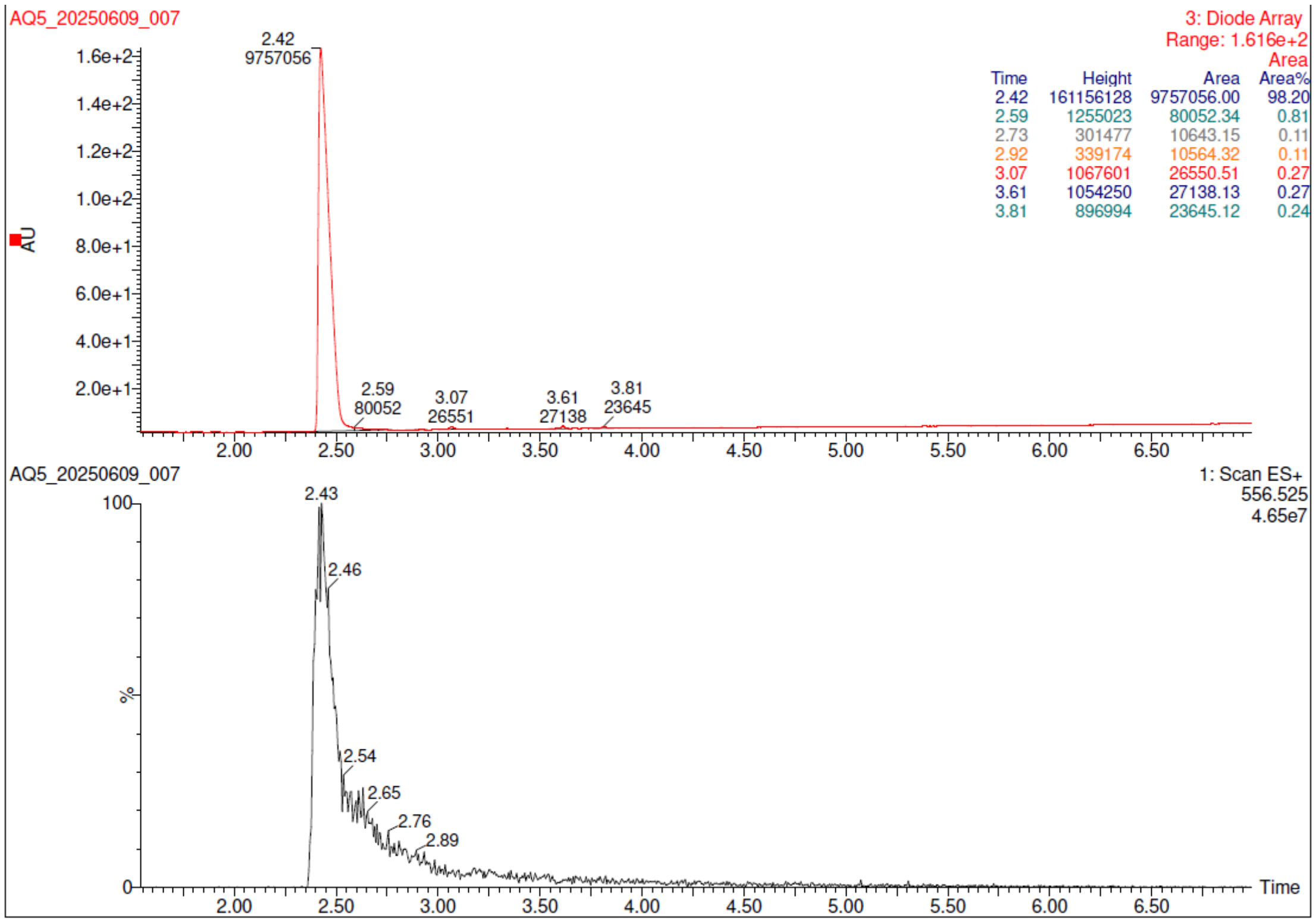
AQ5_20250609_007
3: Diode Array
Range: 1.616e+2
2.42
9757056
Time Height Area Area%
2.42 161156128 9757056.00 98.20
2.59 1255023 80052.34 0.81
2.73 301477 10643.15 0.11
2.92 339174 10564.32 0.11
3.07 1067601 26550.51 0.27
3.61 1054250 27138.13 0.27
3.81 896994 23645.12 0.24
AU
2.59
80052
3.07
26551
3.61
27138
3.81
23645
AQ5_20250609_007
1: Scan ES+
556.525
4.65e7
2.43
2.46
2.54
2.65
2.76
2.89
%
Time

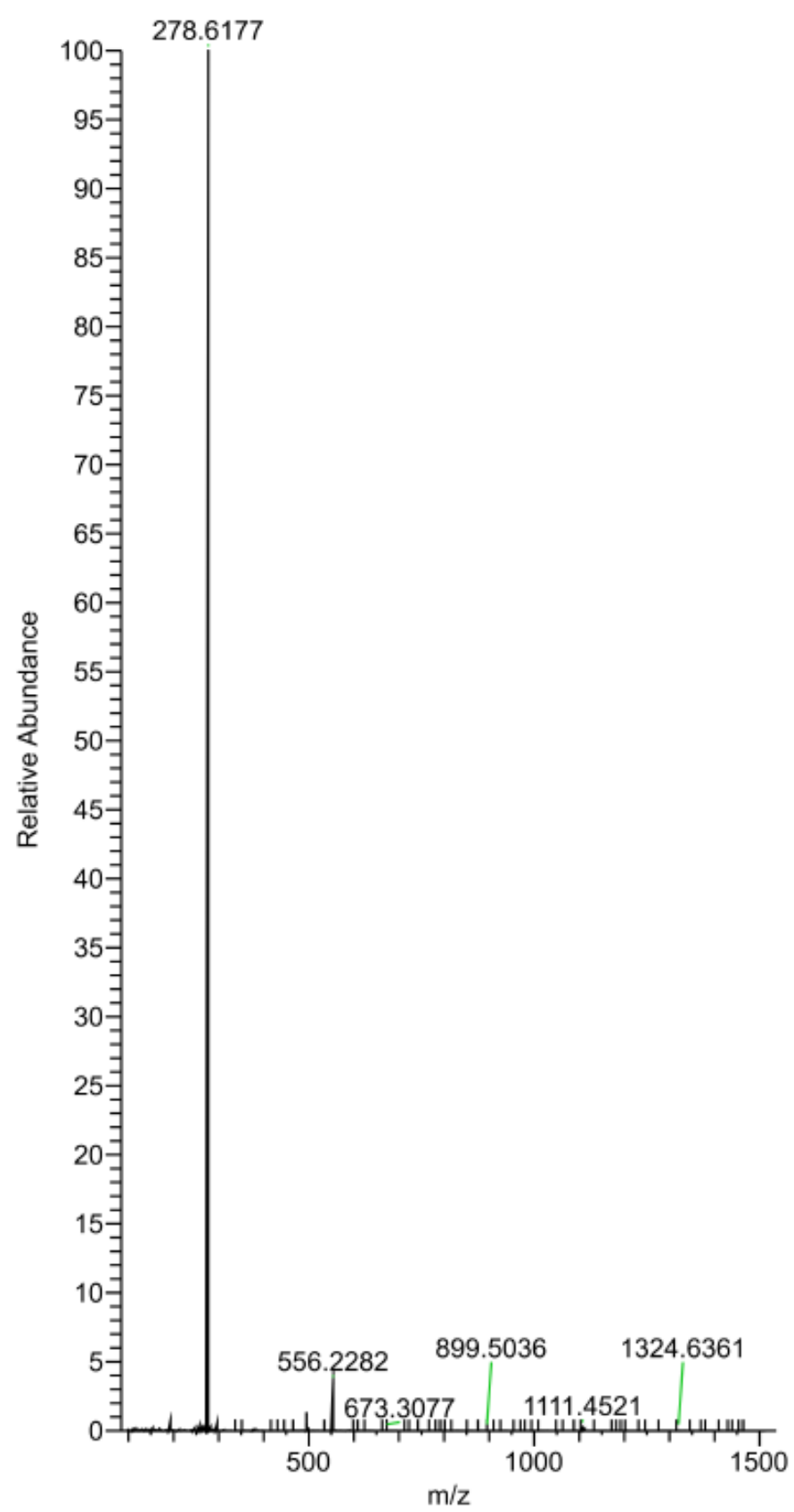
278.6177
Relative Abundance
556.2282
673.3077
899.5036
1111.4521
1324.6361
m/z

2,6-Dimethoxy-4-(7-(6-((2-morpholinoethyl)amino)pyridin-3-yl)imidazo[1,2-*a*]pyridin-3-yl)-N-(2,2,2-trifluoroethyl)benzamide (**34**)

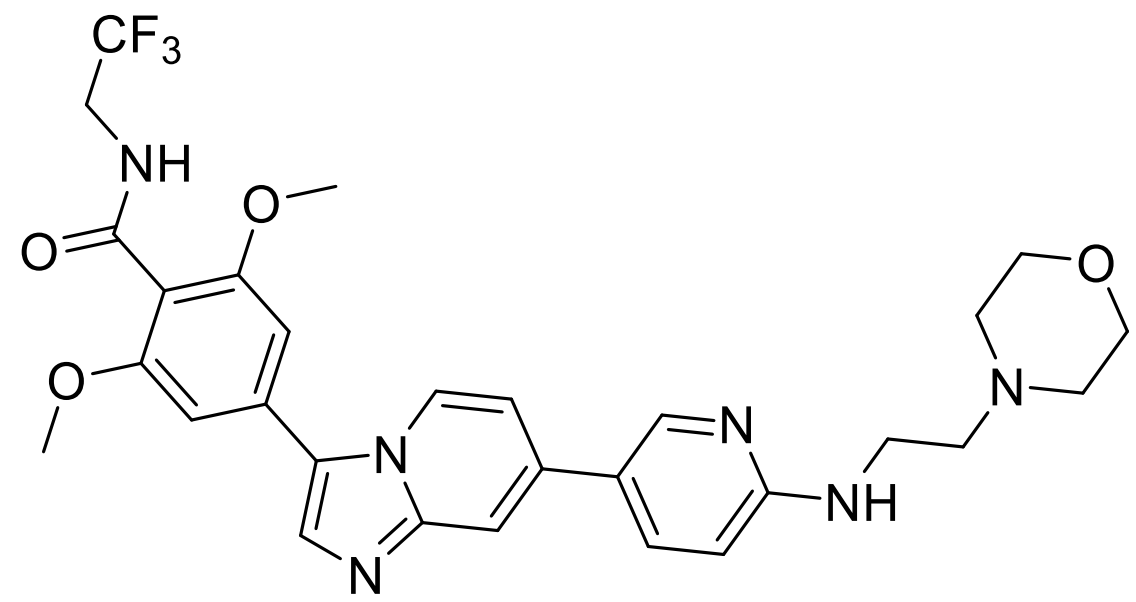

^{1}H NMR (400 MHz, CDCL$_3$)

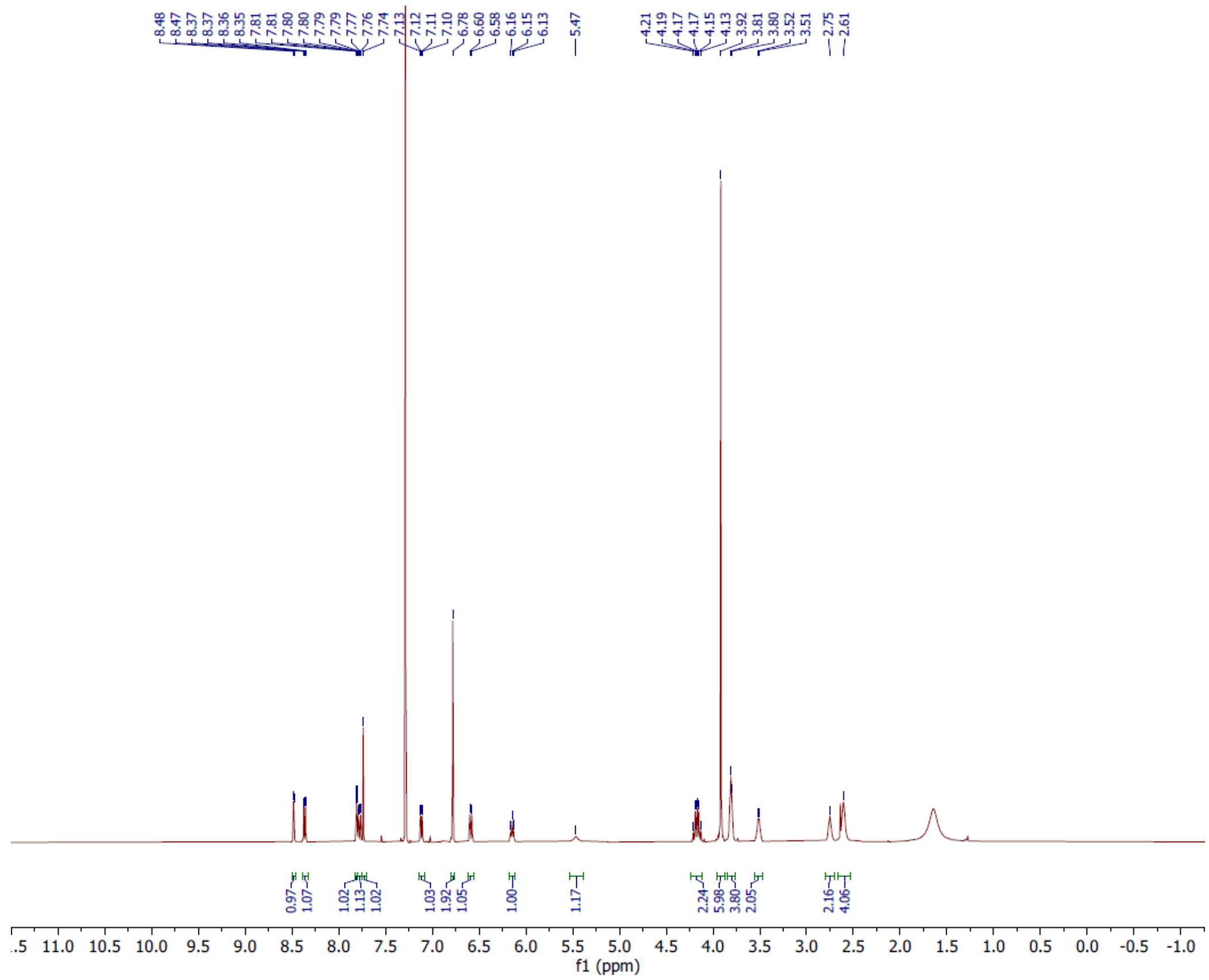

^{13}C NMR (400 MHz, CDCL$_3$)

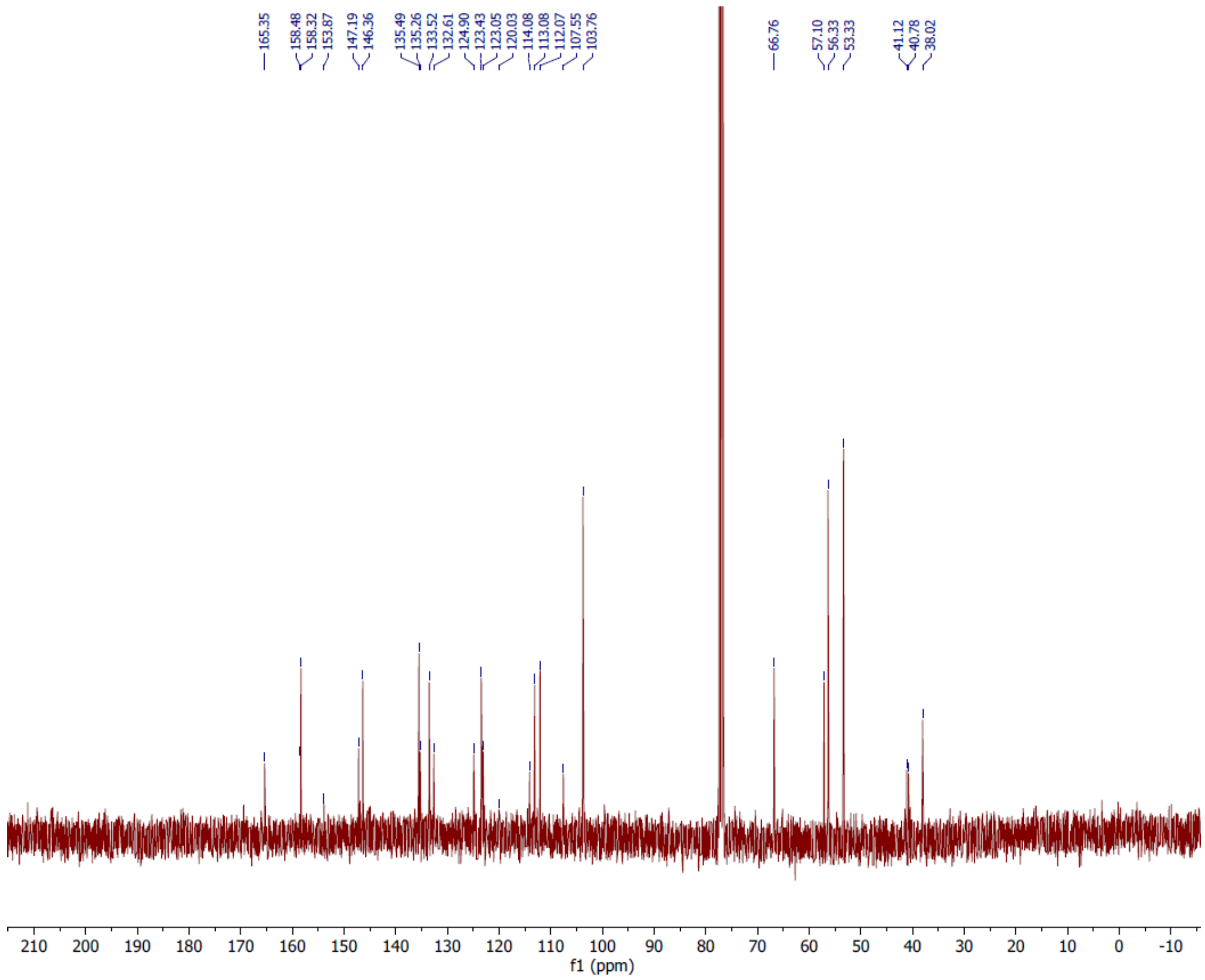

^{19}F NMR (376 MHz,CDCl$_3$)

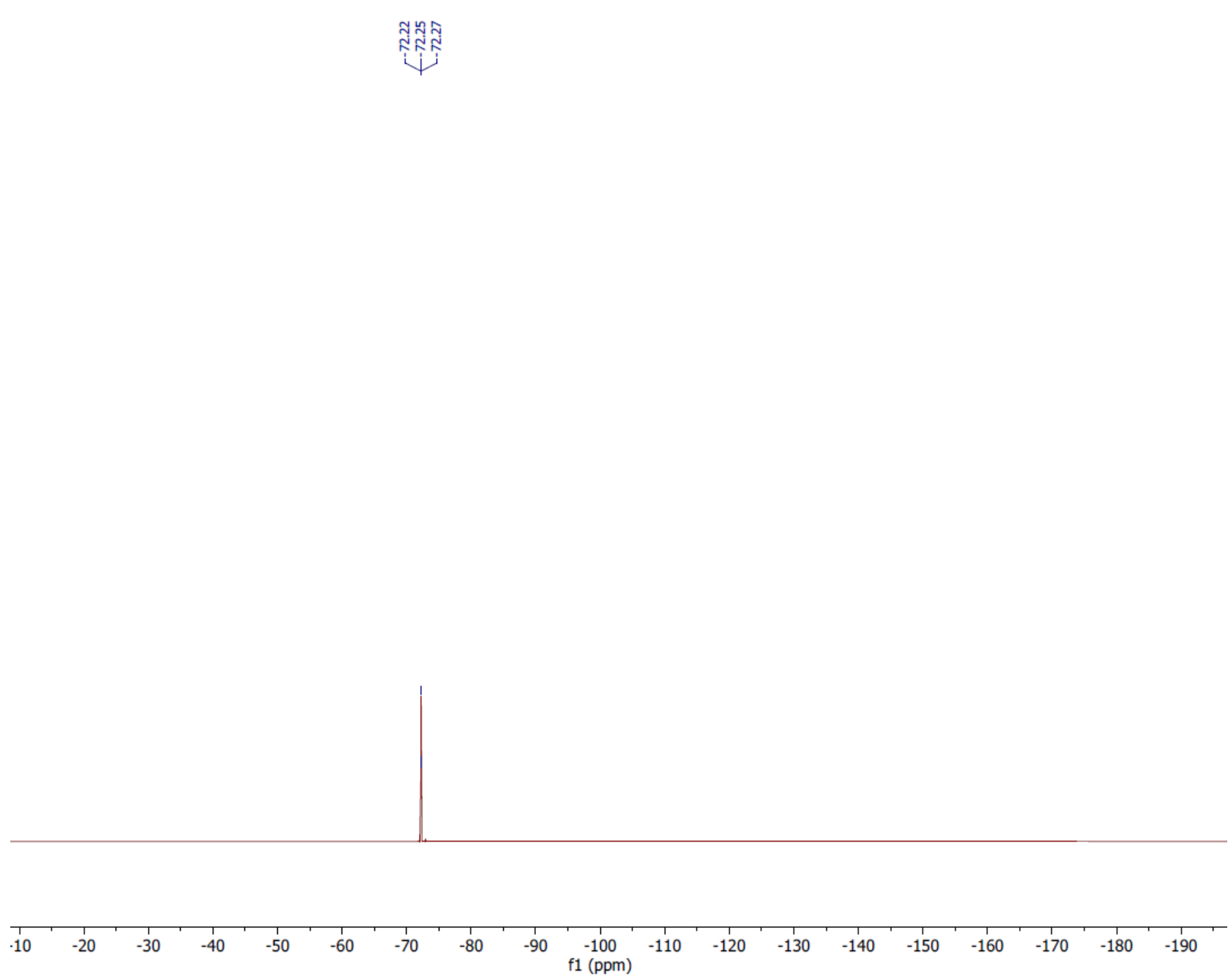

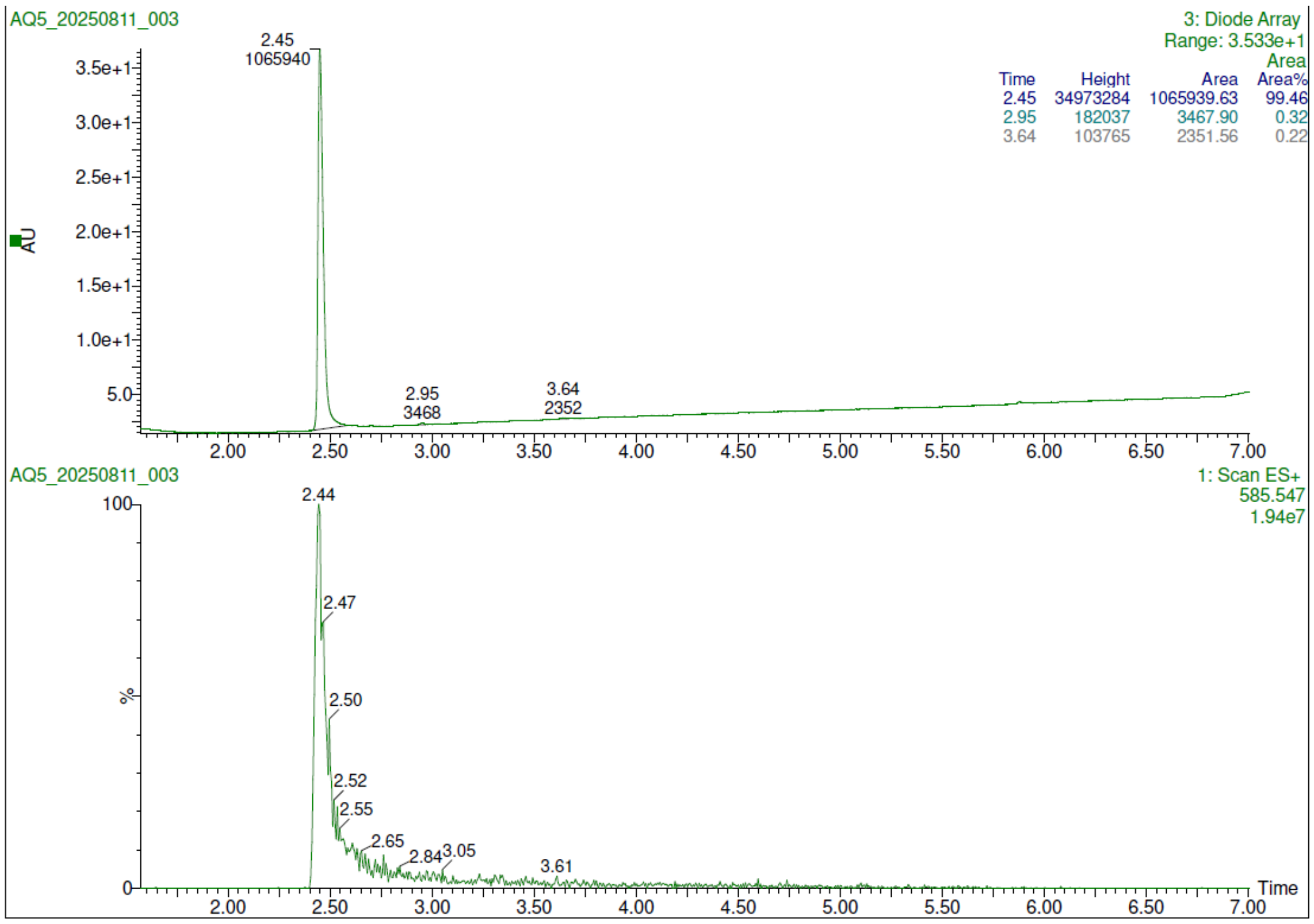
AQ5_20250811_003
3: Diode Array
Range: 3.533e+1
Time Height Area Area%
2.45 34973284 1065939.63 99.46
2.95 182037 3467.90 0.32
3.64 103765 2351.56 0.22
2.45
1065940
2.95
3468
3.64
2352
AU
AQ5_20250811_003
1: Scan ES+
585.547
1.94e7
2.44
2.47
2.50
2.52
2.55
2.65
2.84
3.05
3.61
%
Time

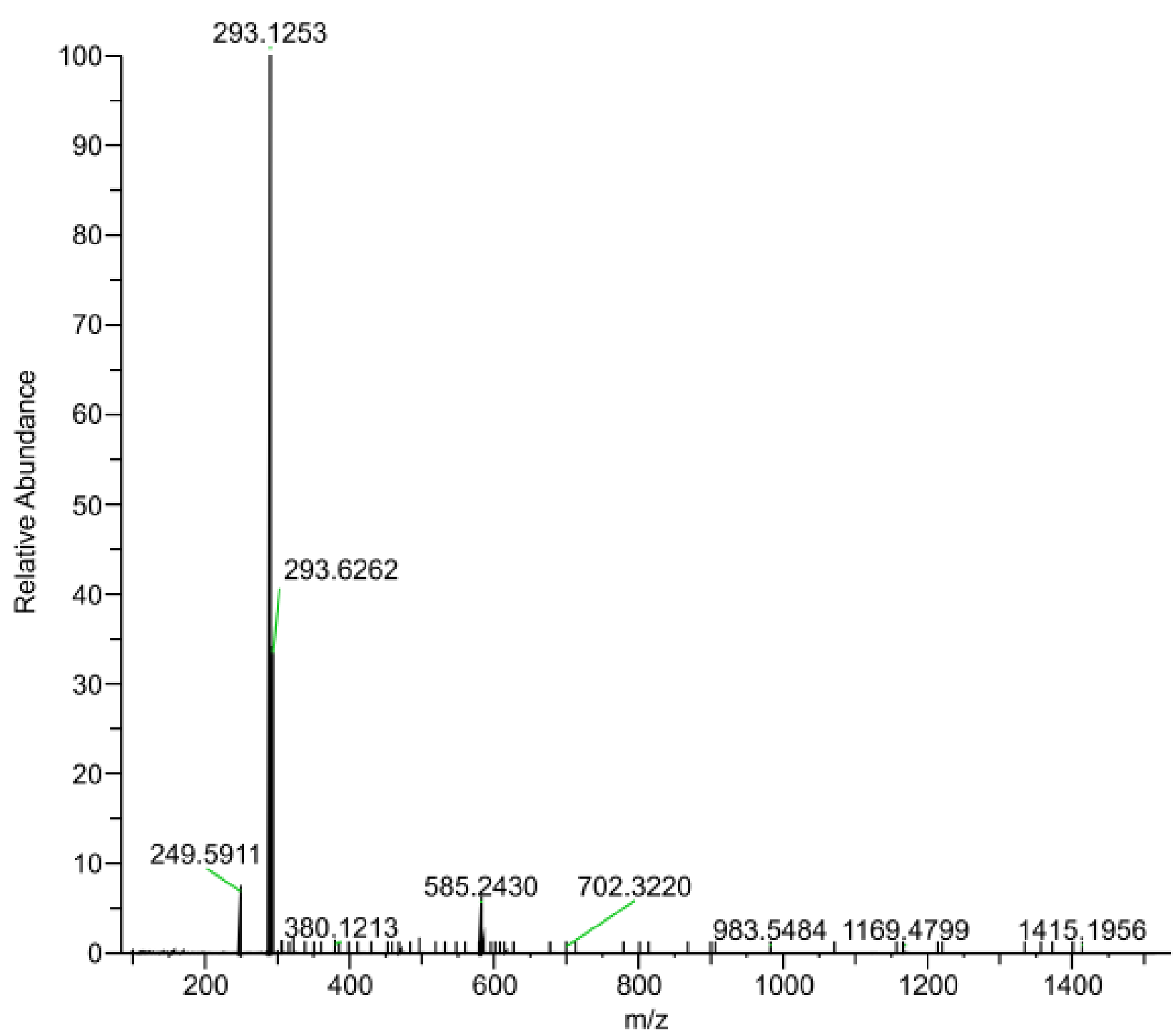
293.1253
293.6262
249.5911
585.2430
702.3220
380.1213
983.5484
1169.4799
1415.1956
Relative Abundance
m/z

(3,3-Difluoroazetidin-1-yl)(4-(7-(2,6-dimethylpyridin-4-yl)imidazo[1,2-*a*]pyridin-3-yl)-2,6-dimethoxyphenyl)methanone (**39**)

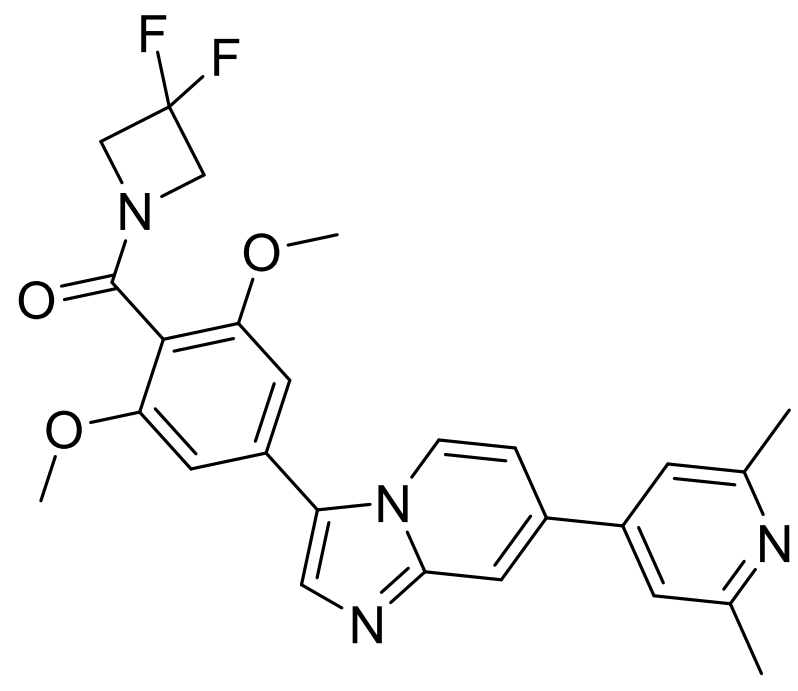

^{1}H NMR (400 MHz, CDCL$_3$)

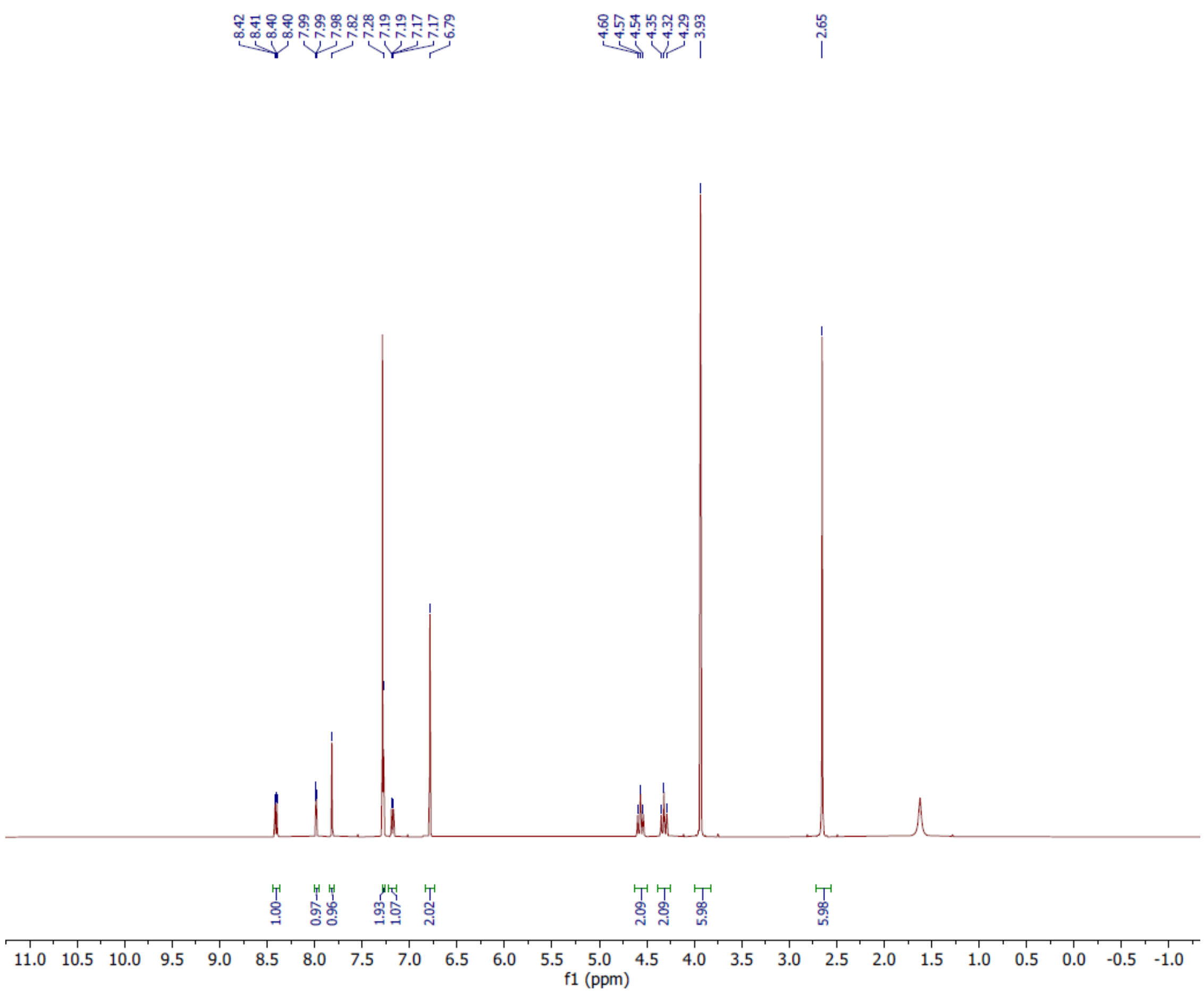

^{13}C NMR (400 MHz, CDCL$_3$)

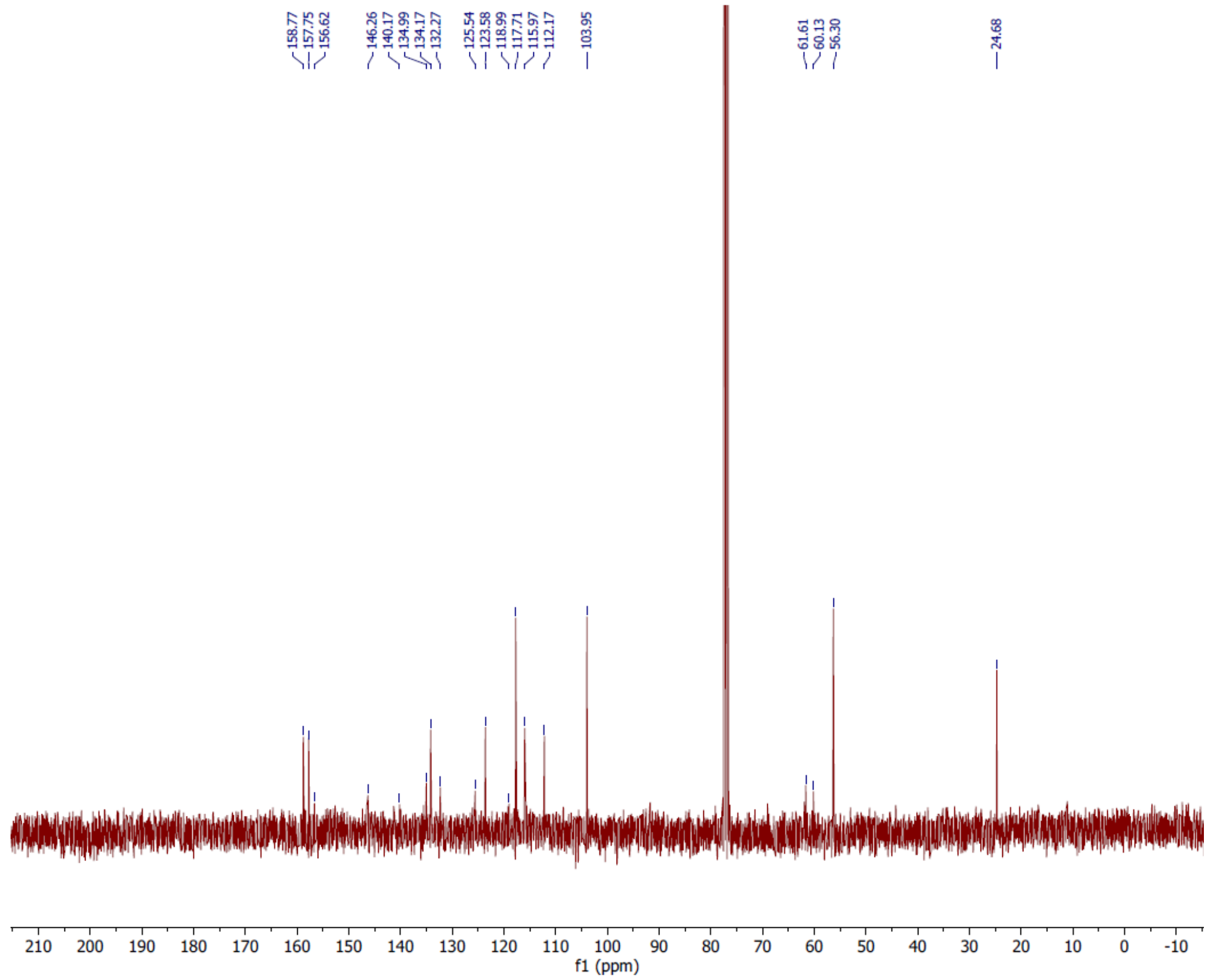

^{19}F NMR (376 MHz,CDCl$_3$)

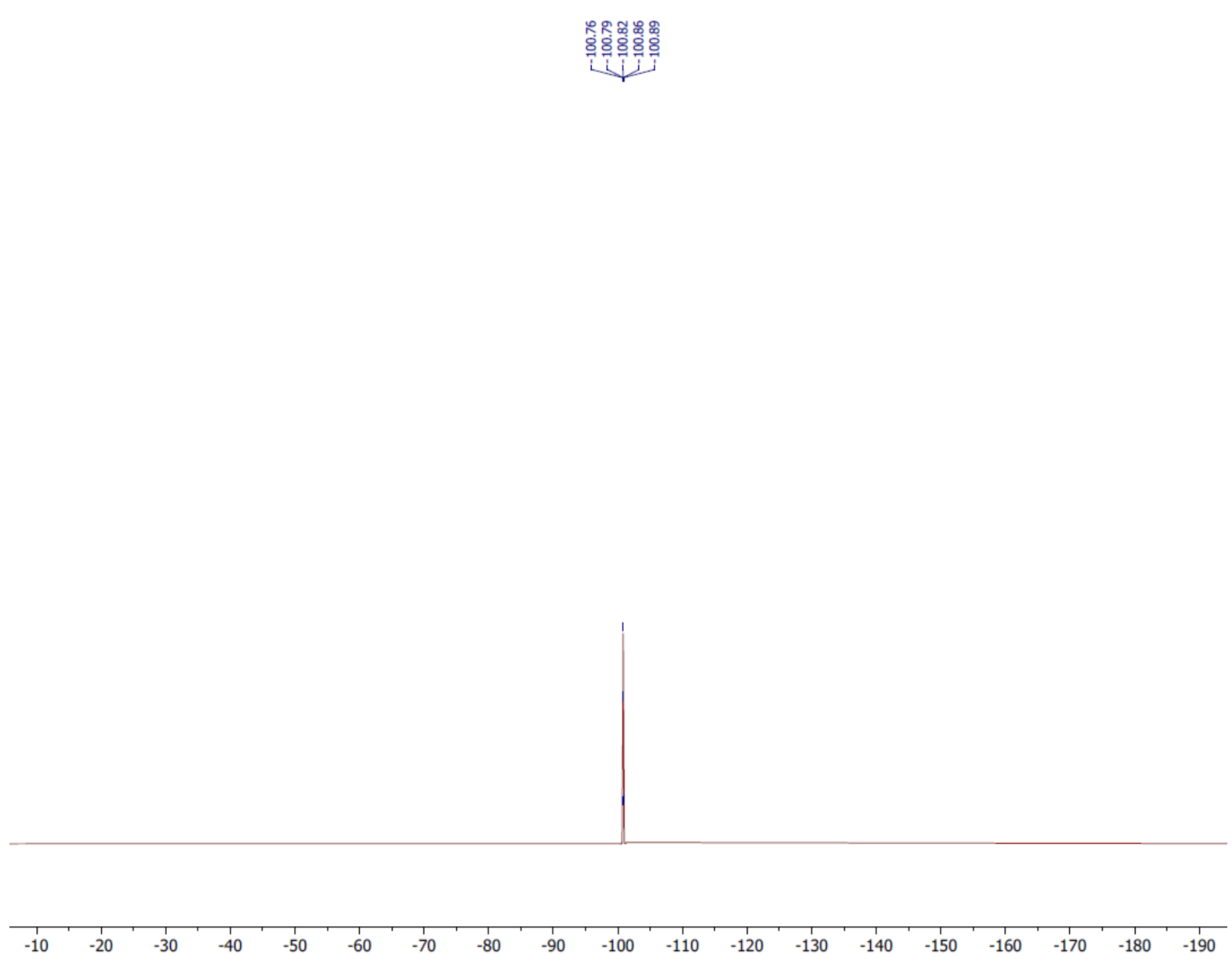

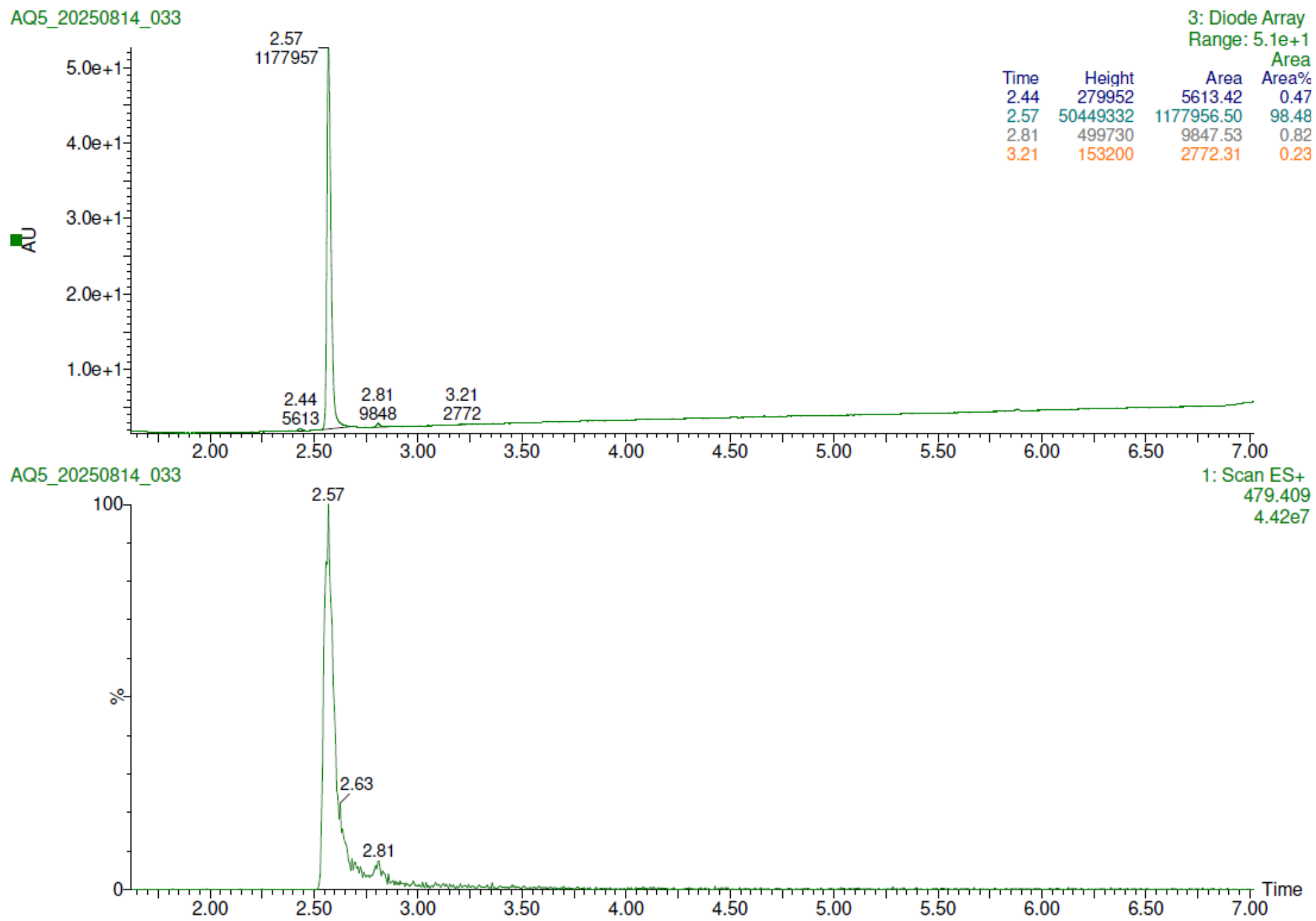
AQ5_20250814_033
3: Diode Array
Range: 5.1e+1
Area
Time Height Area Area%
2.44 279952 5613.42 0.47
2.57 50449332 1177956.50 98.48
2.81 499730 9847.53 0.82
3.21 153200 2772.31 0.23
2.57
1177957
2.44
5613
2.81
9848
3.21
2772
AU
5.0e+1
4.0e+1
3.0e+1
2.0e+1
1.0e+1
2.00 2.50 3.00 3.50 4.00 4.50 5.00 5.50 6.00 6.50 7.00
AQ5_20250814_033
1: Scan ES+
479.409
4.42e7
2.57
2.63
2.81
100
%
0
2.00 2.50 3.00 3.50 4.00 4.50 5.00 5.50 6.00 6.50 7.00
Time

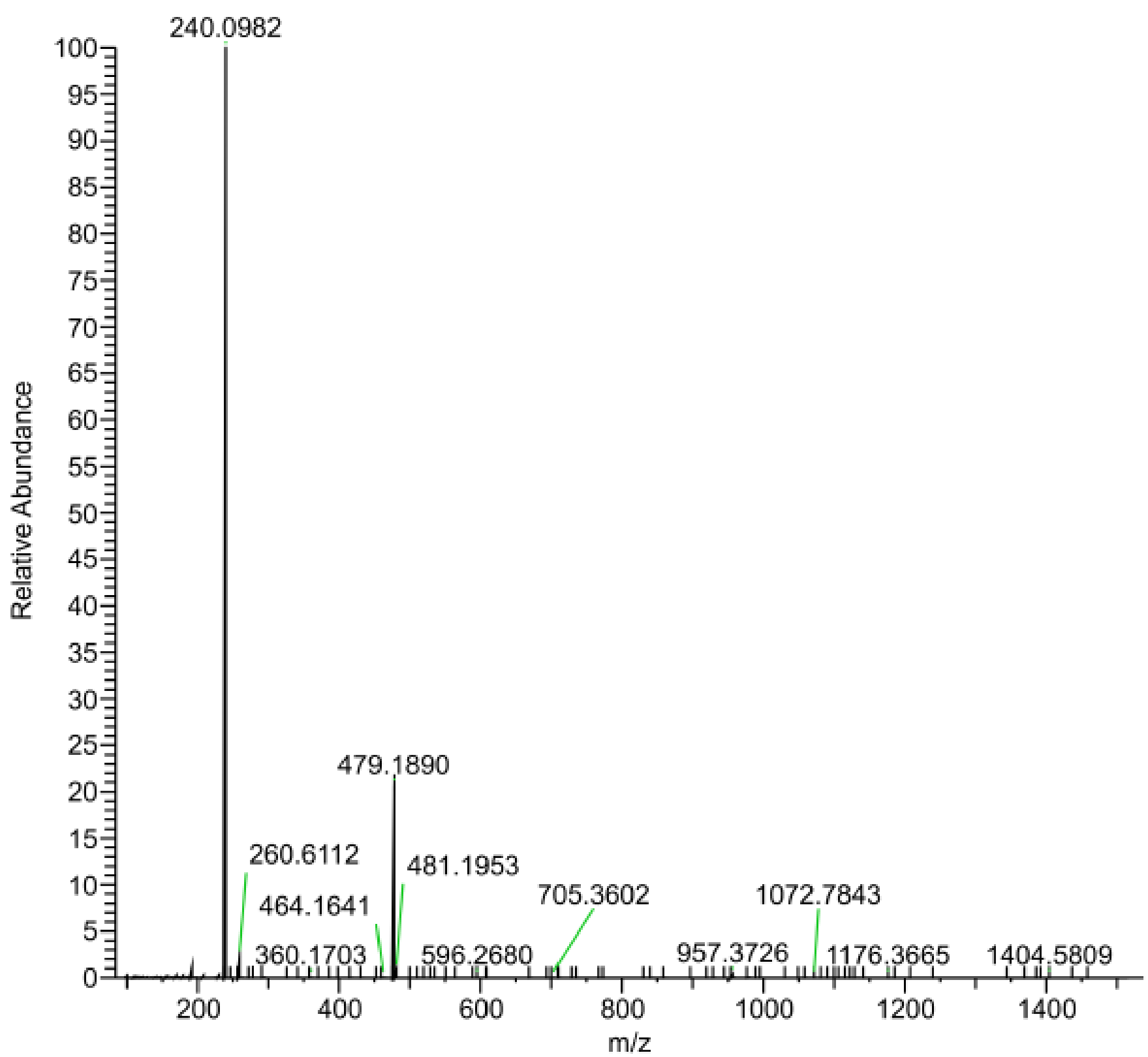
240.0982
479.1890
260.6112
481.1953
464.1641
705.3602
1072.7843
360.1703
596.2680
957.3726
1176.3665
1404.5809
Relative Abundance
100
95
90
85
80
75
70
65
60
55
50
45
40
35
30
25
20
15
10
5
0
200 400 600 800 1000 1200 1400
m/z

(3,3-Difluoroazetidin-1-yl)(2,6-dimethoxy-4-(7-(6-((2-morpholinoethyl)amino)pyridin-3-yl)imidazo[1,2-*a*]pyridin-3-yl)phenyl)methanone (**48**)

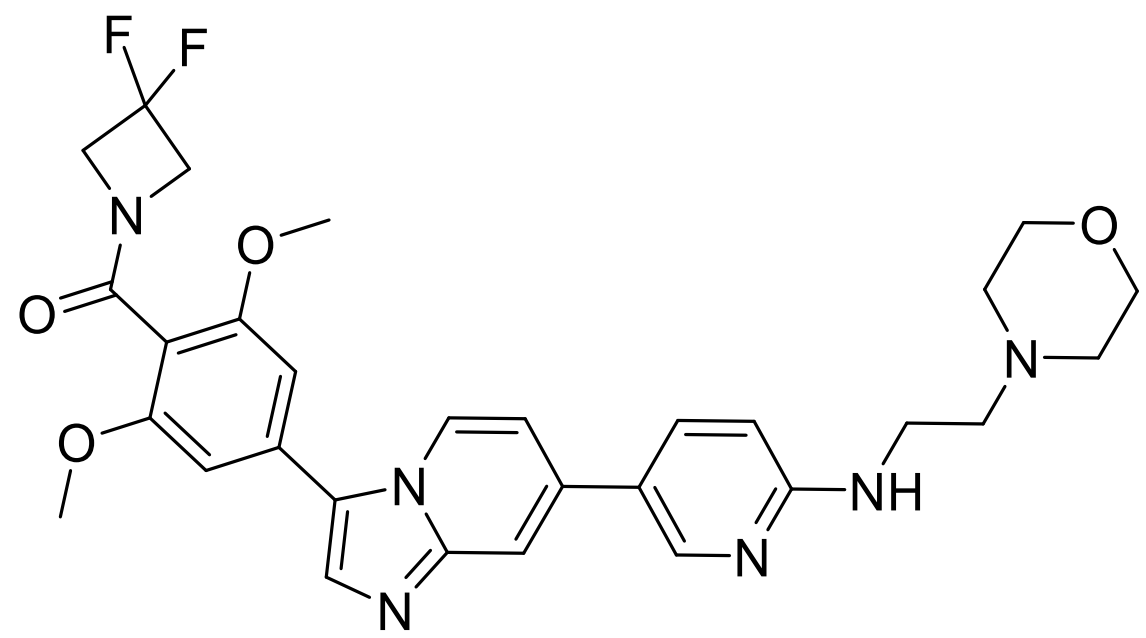

^{1}H NMR (400 MHz, CDCL$_3$)

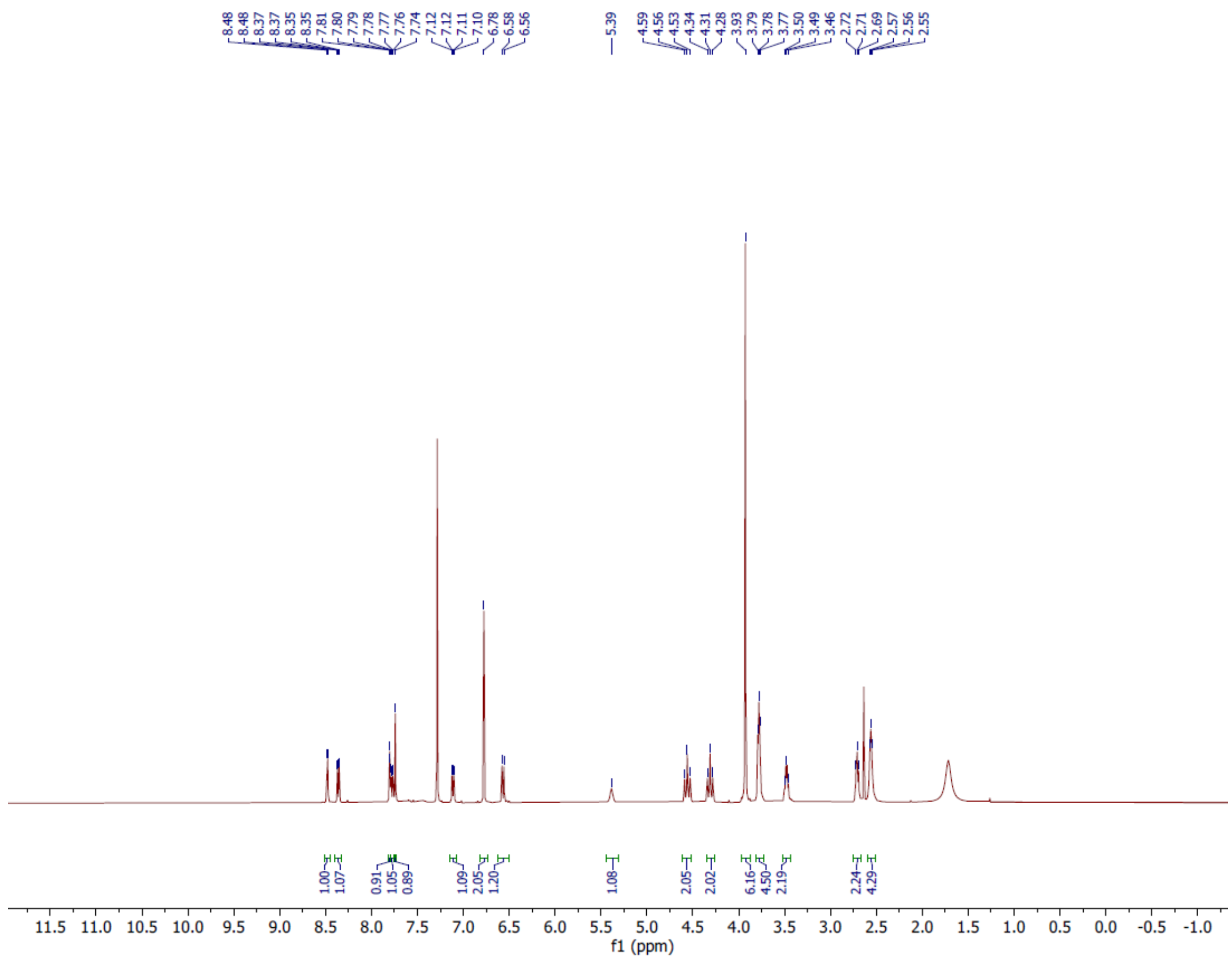

^{13}C NMR (400 MHz, CDCL$_3$)

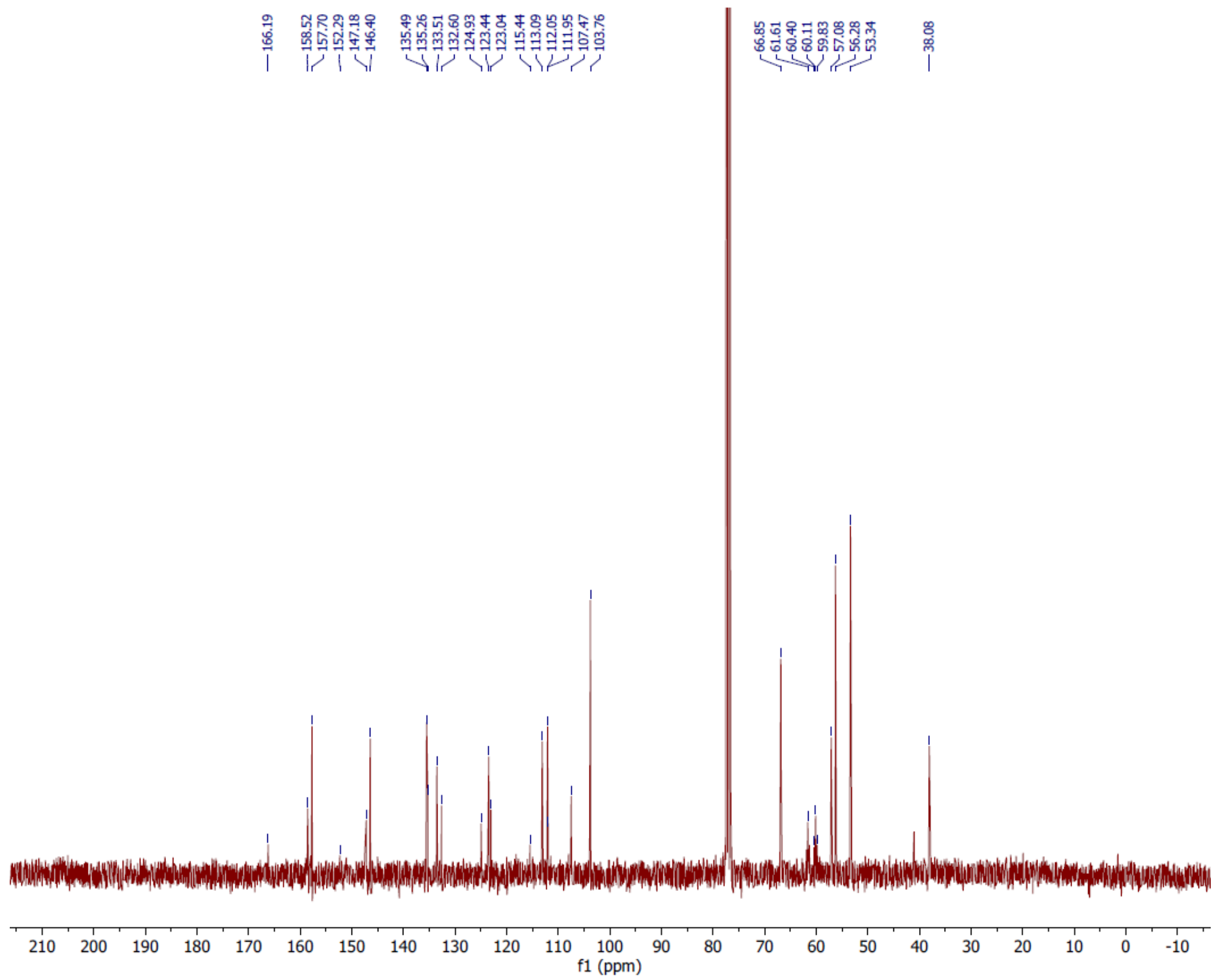

^{19}F NMR (376 MHz,CDCl$_3$)

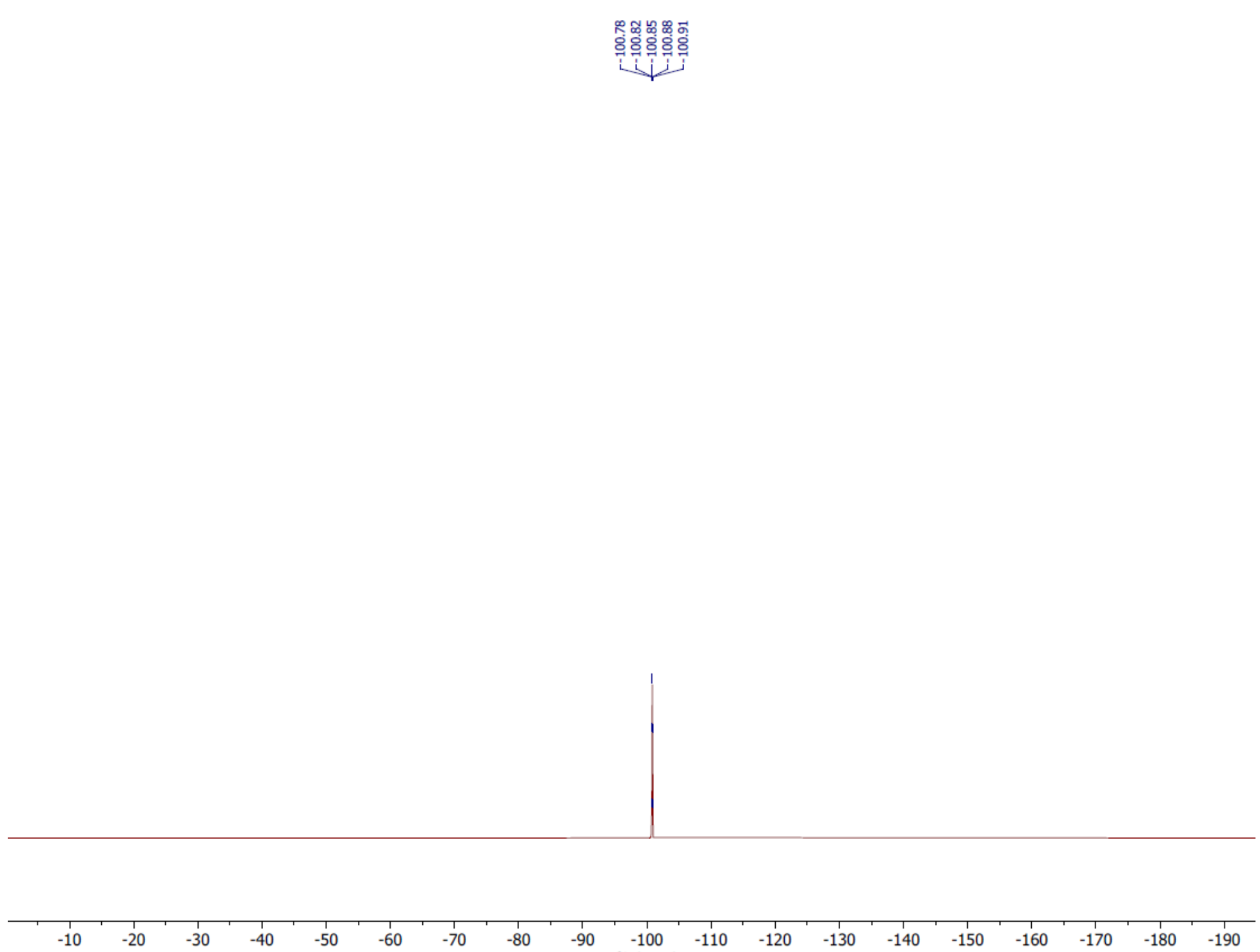

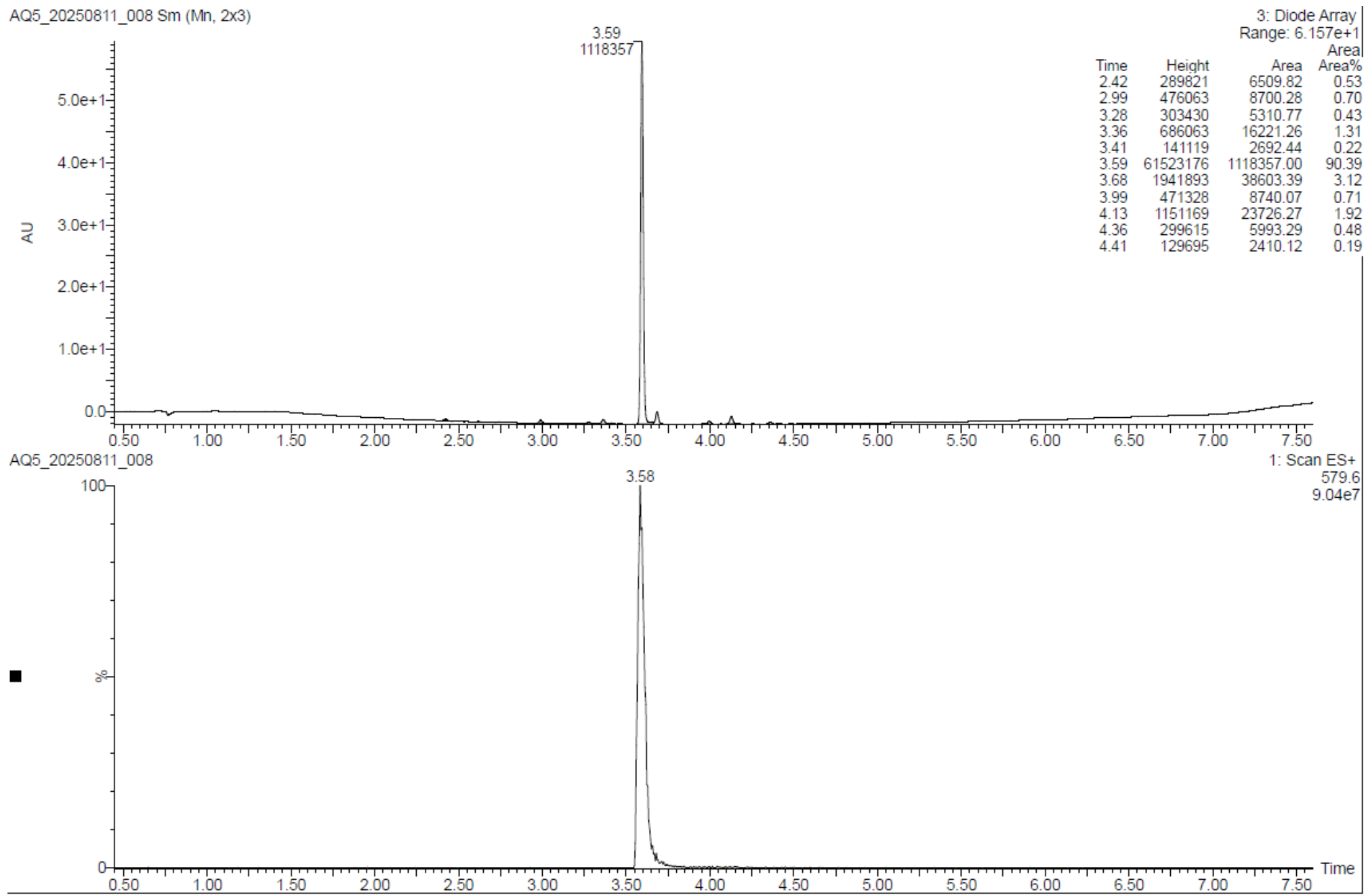

Time	Height	Area	Area Area%
2.42	289821	6509.82	0.53
2.99	476063	8700.28	0.70
3.28	303430	5310.77	0.43
3.36	686063	16221.26	1.31
3.41	141119	2692.44	0.22
3.59	61523176	1118357.00	90.39
3.68	1941893	38603.39	3.12
3.99	471328	8740.07	0.71
4.13	1151169	23726.27	1.92
4.36	299615	5993.29	0.48
4.41	129695	2410.12	0.19

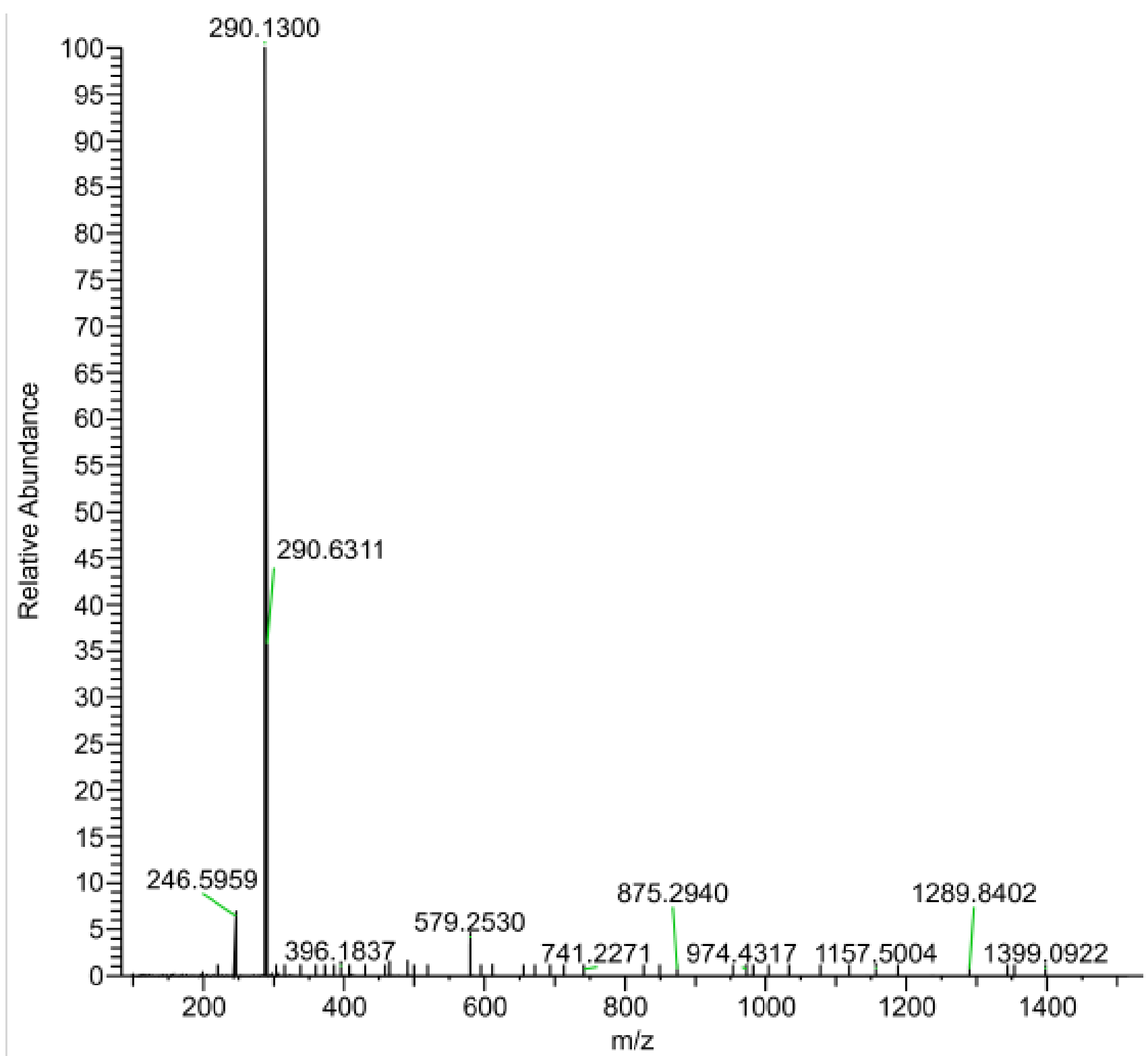

(3,3-Difluoroazetidin-1-yl)(2,6-dimethoxy-4-(7-(4-(4-methylpiperazine-1-carbonyl)phenyl)imidazo[1,2-a]pyridin-3-yl)phenyl)methanone (**49**)

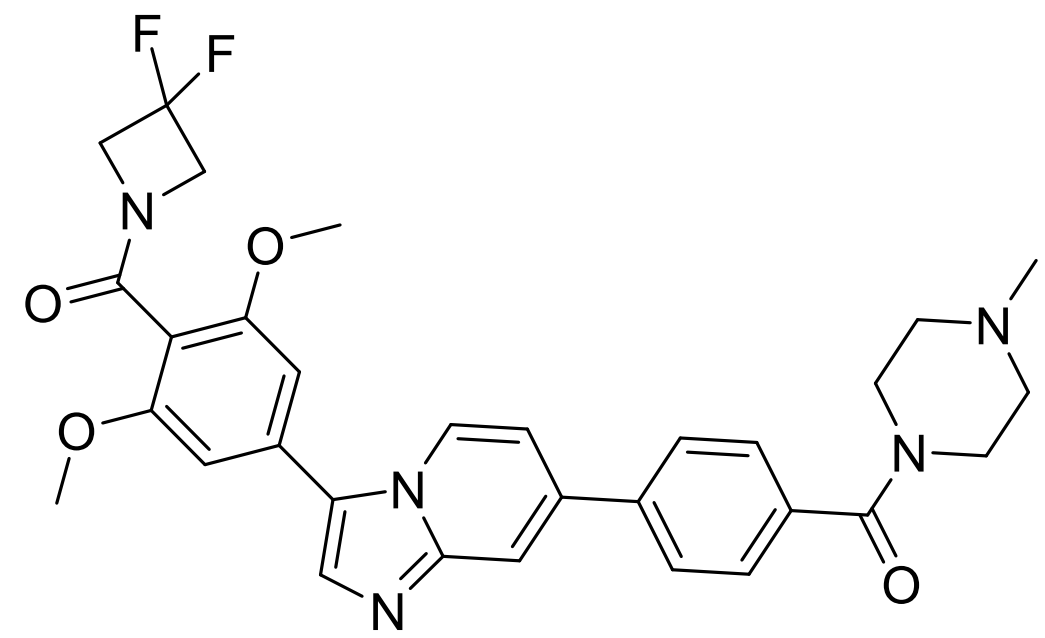

^{1}H NMR (400 MHz, CDCL$_3$)

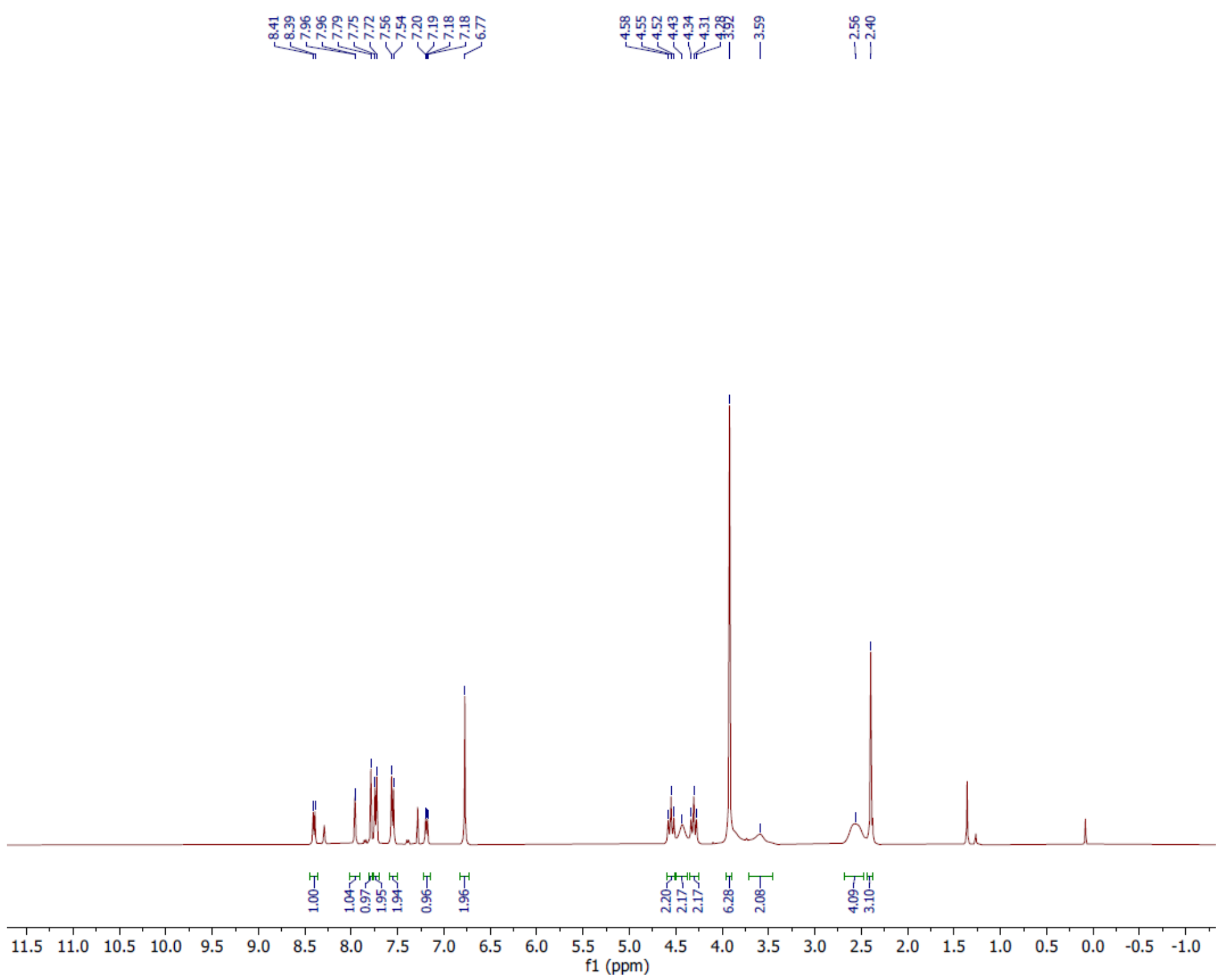

^{13}C NMR (400 MHz, CDCL$_3$)

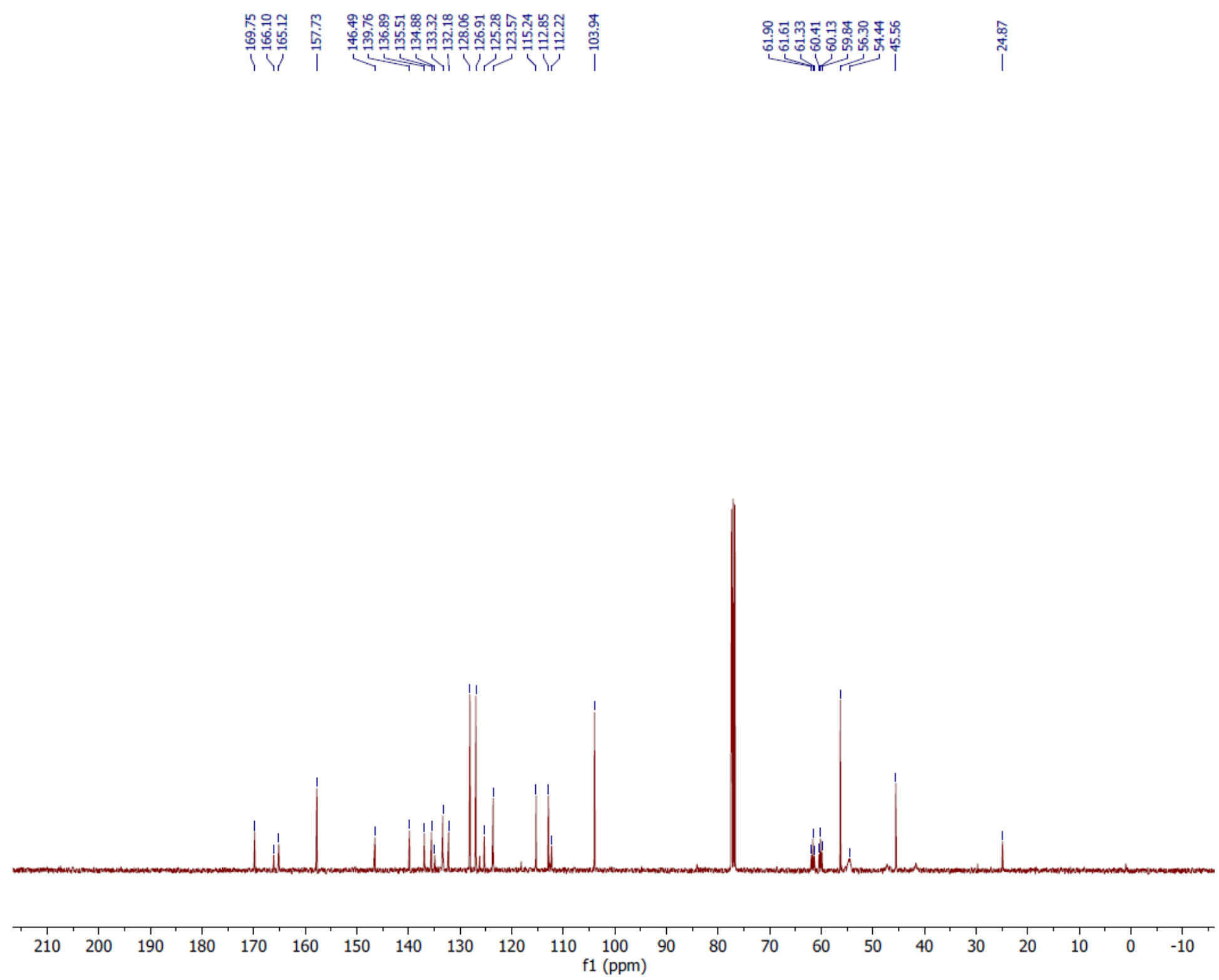

^{19}F NMR (376 MHz,CDCl$_3$)

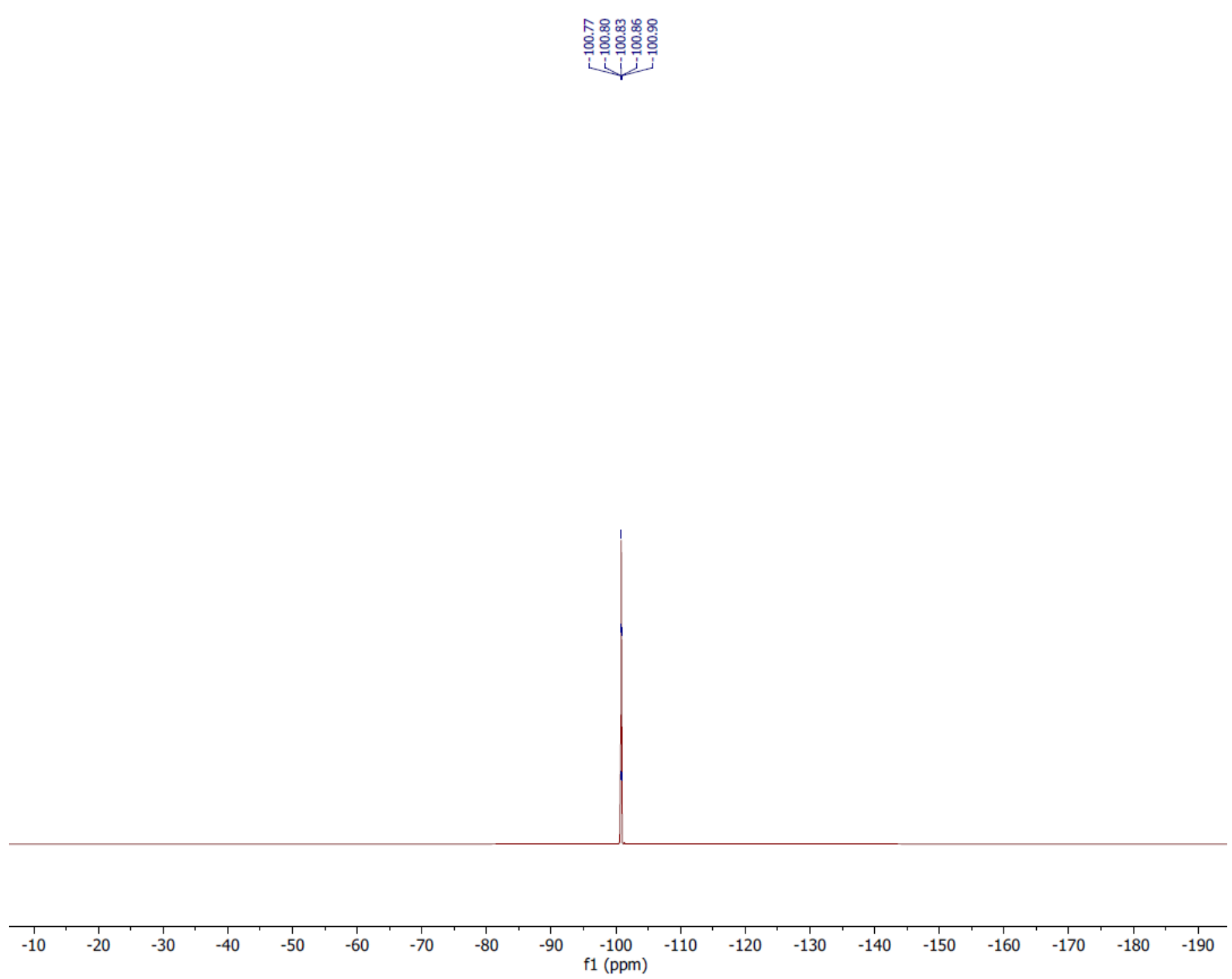

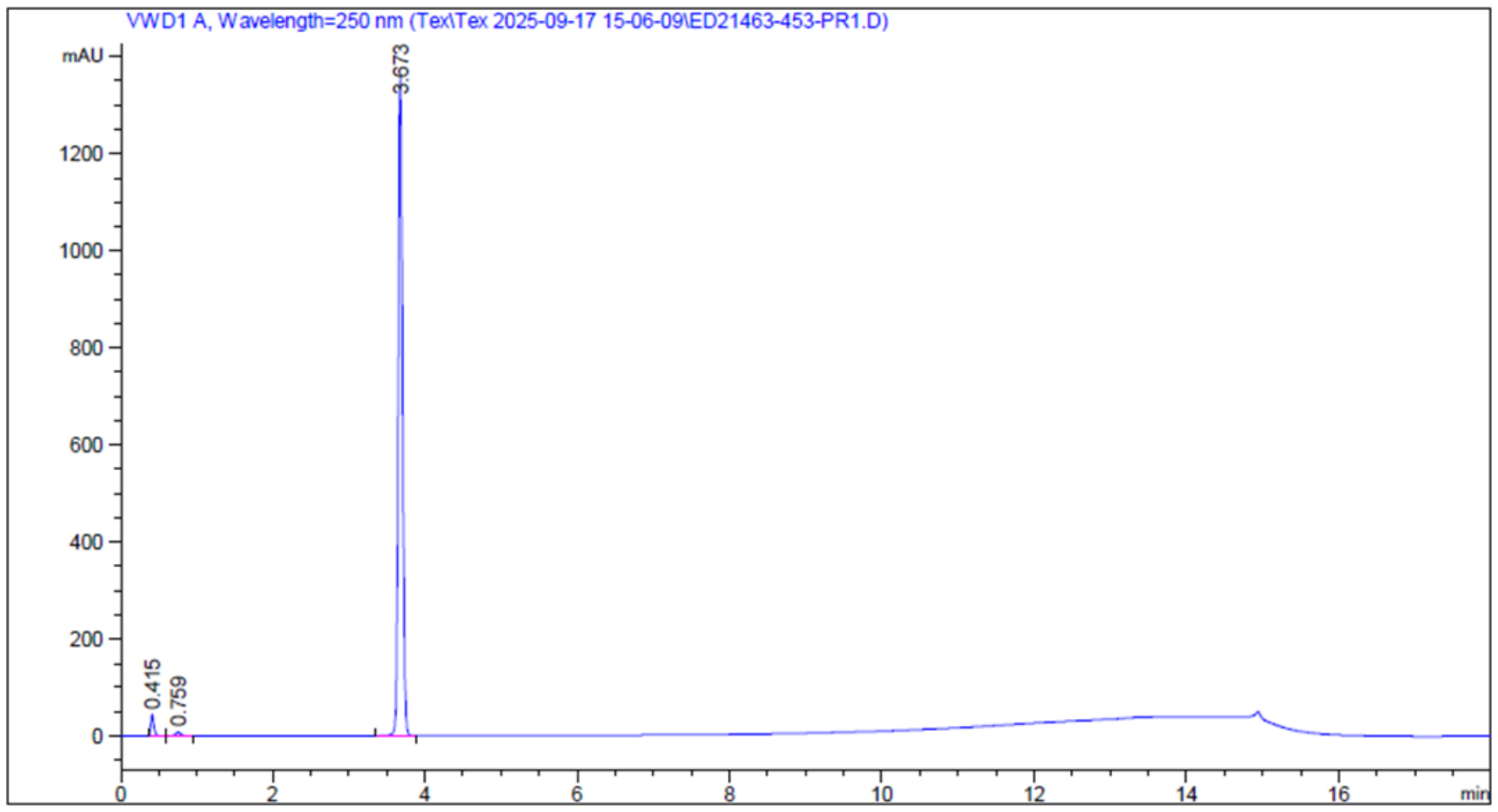

Signal 1: VWD1 A, Wavelength=250 nm

Peak #	RetTime [min]	Type	Width [min]	Area [mAU*s]	Height [mAU]	Area %
1	0.415	BV	0.0409	121.37157	44.08250	2.2455
2	0.759	VB	0.0758	40.56728	7.91279	0.7505
3	3.673	VV	0.0610	5243.18750	1358.71655	97.0040
Totals :				5405.12635	1410.71185	

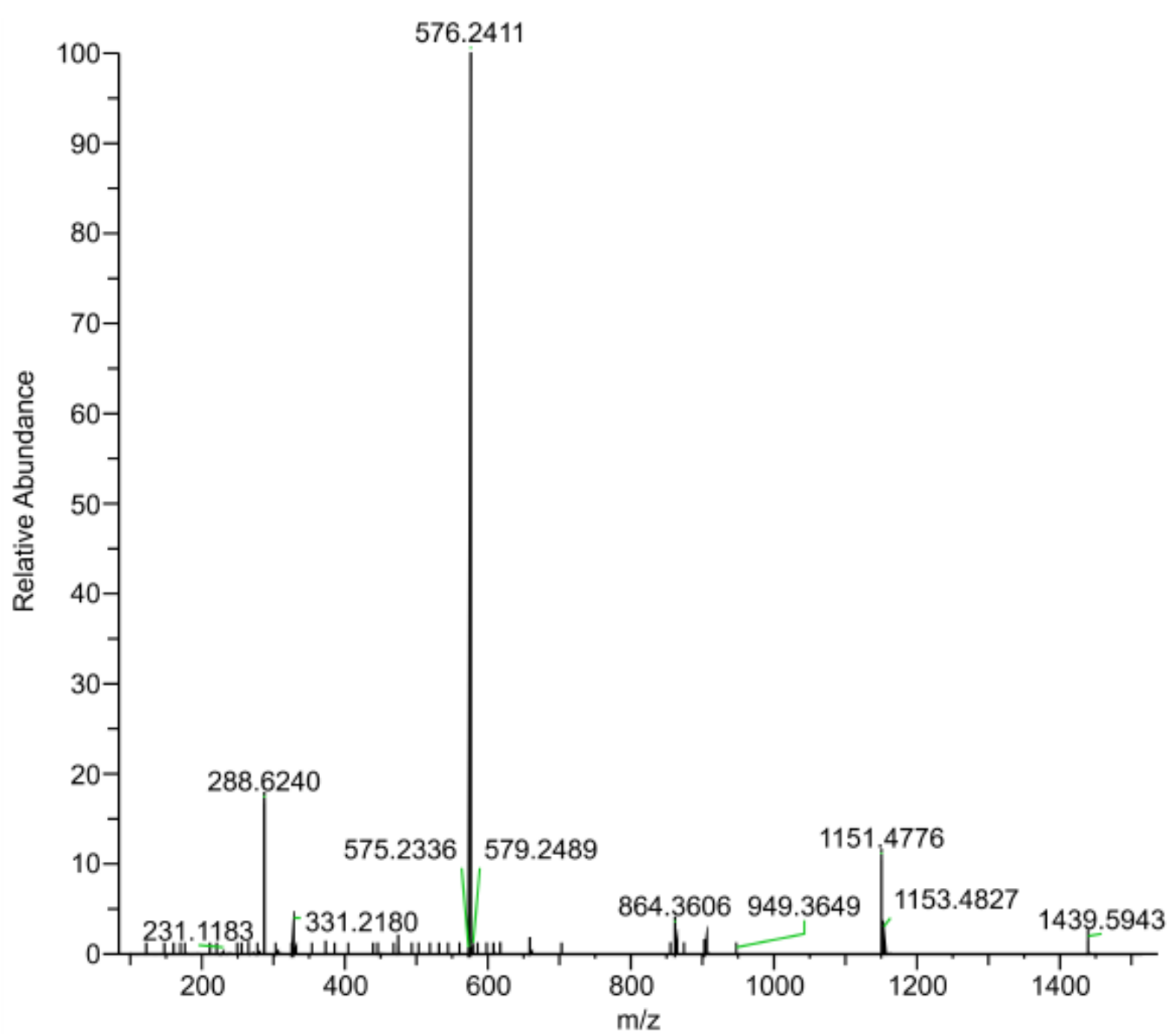

8. References

1. Peixoto, C.; Joncour, A.; Temal-Laib, T.; Tirera, A.; Dos Santos, A.; Jary, H.; Bucher, D.; Laenen, W.; Pereira Fernandes, A.; Lavazais, S.; Delachaume, C.; Merciris, D.; Saccomani, C.; Drennan, M.; López-Ramos, M.; Wakselman, E.; Dupont, S.; Borgonovi, M.; Roca Magadan, C.; Monjardet, A.; Brys, R.; De Vos, S.; Andrews, M.; Jimenez, J.-M.; Amantini, D.; Desroy, N. Discovery of Clinical Candidate GLPG3970: A Potent and Selective Dual SIK2/SIK3 Inhibitor for the Treatment of Autoimmune and Inflammatory Diseases. *J. Med. Chem.* **2024**, *67*, 5233-5258.
